# Effects of Fertility Behaviors on Depression Among the Elderly: Empirical Evidence From China

**DOI:** 10.3389/fpubh.2020.570832

**Published:** 2021-01-26

**Authors:** Zhen Hu, Yuanyang Wu, Hualei Yang, Lin Xie, Anqi Zhang, Xueyu Lin, Yafeng Nie, Xiaoyu Zhang

**Affiliations:** ^1^School of Economics and Management, Northwest Agricultural and Forestry University, Xianyang, China; ^2^School of Public Administration, Zhongnan University of Economics and Law, Wuhan, China; ^3^Institution of Population and Labor Economics, The Chinese Academy of Social Science, Beijing, China; ^4^School of Public Economy and Management, Shanghai University of Finance and Economics, Shanghai, China

**Keywords:** fertility behavior, the elderly, depression, mental health, childbirth

## Abstract

**Background:** Increased population aging is associated with increased incidence of depression among the elderly. Existing studies have shown that ill-advised fertility behaviors during their youth also affect the health of the elderly. However, insufficient attention has been paid to depression among elderly in China. This paper focuses on how fertility behaviors affect senile depression among parents by examining the heterogeneity of such effects and tests the applicability of existing theoretical findings in a Chinese sample.

**Methods:** The effects of fertility behaviors on depression among the elderly were investigated using the China Health and Retirement Longitudinal Study (CHARLS), a nationally representative dataset. The effects of early-age fertility behaviors on the degree of depression among the elderly were investigated using ordinary least squares and ordered probit models that adjusted for demographic and socioeconomic factors.

**Results:** (1) The age of first childbirth, childbearing period, and number of births were significantly and positively correlated with the degree of depression among the elderly (particularly rural persons aged 50–70 and older womens). (2) Elderly persons with sons had no better mental health status than those without sons, thus indicating the inapplicability of the traditional concept of “more sons are equal to more happiness” to the actual mental health situation of the elderly in China today.

**Conclusion:** Overall, multiple, late, and boy-oriented childbearing and overly long childbearing periods had negative effects on mental health among Chinese elderly persons. This study tested the applicability of existing theoretical inferences and empirical conclusions in China, thereby further expanding the current literature regarding the effects of fertility behaviors on depression among the elderly.

## Introduction

Increased population aging is associated with increased incidence of depression among the elderly (1). Studies have shown that ill-advised fertility behaviors at a young age also affect the health of the elderly, particularly their mental health (2–4). This raises the question of whether there exists a relationship between fertility behaviors (e.g., number of births, age of first childbirth, childbearing period, and gender structure^1^) and the degree depression among the elderly.

Regarding the effects of fertility behaviors on the mental health of parents, it is known that fertility generally increases the rate of depression among mothers. However, there is no current consensus on how this affects fathers (5–8). However, in general, mothers experience more stress than fathers (5). This is because many mothers are required to work while also raising children; studies have shown that raising children increases negative emotions (5, 6). While this finding has since been corroborated, it has simultaneously been shown that childbearing can actually increase happiness for fathers (7, 9). In contrast, a study that found motherhood did not increase positive emotions for women also found that fatherhood did not significantly increase life satisfaction for men (8). Roeters et al. (10) further argued that parenthood has no effect on happiness for men, but has negative effects on happiness for women. Further, prenatal lifestyles exert differential effects on happiness between men and women. Specifically, men with active lifestyles who become fathers tend to find that their happiness decreases, while women with substantial amounts of leisure time or long working hours who become mothers experience positive effects on their happiness, albeit in relatively small measure.

Regarding the effects of the childbearing age and period on parental mental health, most studies have found that extremely early childbearing ages and long childbearing periods increase the degree of depression for women. For instance, adolescent mothers aged 15–17 are at least twice as likely to experience depression compared with adult mothers (11). Further, women who bear their first children at the age of 30 have the lowest degree of depression (12). Premature childbearing age, excessive childbearing, and giving birth to relatively heavy babies also increase disease risk for women, while becoming a father and raising children affects the physical health of men (13). In addition, fathers tend to face higher disease risk if their first children are born at their early age and/or when the mother bears children of abnormal weights. In contrast, men may experience physical health benefits if they do not become fathers until 25 years of age or later. Engelman et al. (14) studied the effects of the birth gender structure on health, thus finding that parental physical functioning was correlated with the number of raised daughters as opposed to the number of raised sons; in particular, these effects were more significant among older fathers when compared with older mothers.

Childbearing usually reduces happiness through the lack of home care and social support, imbalances between career development and home care, and reductions in personal leisure and consumption. Further, women who have difficulties with childcare are more likely to become depressed, while wives who leave childcare to other family members are less depressed, and husbands are significantly less depressed than wives (15). Among African–American women, research has shown that increased numbers of children and gravidity significantly aggravate depressive symptoms, while those without childbearing experience have fewer depressive symptoms; this is because social support moderates the positive correlation between the number of births and depression (16).

In contrast with previous studies, this study focuses on how fertility behaviors affect senile depression among parents (e.g., based on the number of births, age of first childbirth, childbearing period, and the birth gender structure). More specifically, the study examined the heterogeneity of such effects and tested the applicability of existing theoretical findings among a Chinese sample. Considering that senile depression cannot affect fertility behaviors at younger ages, this study adopted ordinary least squares (OLS) and ordered probit models. Data from the 2013 China Health and Retirement Survey (CHARLS) were used to investigate how younger-age fertility behaviors impacted the degree of depression among Chinese individuals aged 50 years and above. Specific variables assessed included the number of births, age of first childbirth, childbearing period, and birth gender structure. This study also analyzed whether these impacts differed between those living in urban and rural areas as well as between those of different ages and genders.

The remainder of this paper is organized as follows: section Materials and Methods describes the CHARLS database, variable selection process, and model setup; section Results analyzes the effects of younger-age fertility behaviors on depression among elderly persons (e.g., based on the number of births, age of first childbirth, childbearing period, and the birth gender structure); section Discussion analyzes the different effects of fertility behaviors on depression among elderly persons across those living in urban and rural areas, of different genders, and at different ages. Finally, section Conclusion provides some conclusions based on those findings. This study tested the applicability of existing conclusions among a Chinese sample, thus expanding upon and deepening the current understanding of how younger-age fertility behaviors affect depression among the elderly.

## Materials and Methods

### Data Sources and Study Ethics

This study extracted data from the 2013 CHARLS database, which was compiled by the National School of Development, Peking University. The baseline survey began in 2011, while longitudinal surveys were conducted in 2013, 2015, and 2018. Households were randomly chosen using a probability proportionate to size sampling strategy from 28 provinces in China. Participants aged over 45 years old and their spouses (if any, and of any age) were interviewed every 2 years. The survey sample consisted of ~19,000 total respondents, who completed questionnaire items covering information on their personal profiles, family members, personal income, and expenditures. Specifically, the 2013 longitudinal survey involved 10,624 households from 150 counties/districts in 28 provincial regions of China, thus ensuring high representativeness. Considering the sample characteristics, this study used data from respondents aged 50 years and above. After excluding improper household data and forms with missing values, the final sample size was 10,553. The CHARLS study was approved by the Ethical Review Committee at Peking University (IRB00001052–11015).

### Depression

Childbearing primarily affects mental health of the elderly in the areas of cognition, depression, loneliness, and life satisfaction. In this study, the degree of depression was used as the dependent variable. When completing the 2013 CHARLS questionnaire, respondents were prompted to answer items that addressed the individual's degree of depression. For example, this included “I felt lonely,” “I felt depressed,” “my sleep was restless,” and “I felt fearful.” Ten questions constituted the depression rating scale (17). All scores were summed to indicate the degree of individual depression, with higher scores indicating more severe depression (scores ranged from 0 to 30). Approximately 70% of all respondents were deemed to be without depression (sample distribution was approximately normal).

### Fertility Behavior

This study considered fertility behavior as the explanatory variable. Following previous studies, specific fertility behaviors instead of the age of first childbirth, childbearing period, the birth gender structure, and number of births (4, 14, 18). The age of first childbirth was equal to the mother's age minus the age of her eldest child, the childbearing period was equal to the difference between their age of first childbirth and age of last childbirth, and the number of births was equal to the number of children born by the mother. Next, the birth gender structure indicated whether any children were male (1: yes; 0: no). Birth gender structure was represented as a dummy variable in the statistical models. In the selected sample, the age of first childbirth ranged from 18 to 60, the childbearing period was <37 years, and the number of births was <11. Respondents with both sons and daughters accounted for ~80% of the total sample.

### Covariates

Following previous studies of mental health among the elderly, this study considered several control variables, including individual age, gender, spouse status, domicile registration, years of education, quality of children, cohabitation with children, personal income, medical insurance, and pension insurance. Among these, gender, spouse status, domicile registration, cohabitation with children, medical insurance, and pension insurance were set as dummy variables, which were assigned the following values: gender (1: female; 0: male), spouse status (1: available; 0: not available), domicile registration (1: urban; 0: rural), cohabitation with children (1: yes; 0: no), medical insurance (1: covered; 0: not covered), and pension insurance (1: covered; 0: not covered). Age, years of education, quality of children, and personal income were all set as continuous variables. The lower age limit was set to 50 years, at which time women generally lose their fertility, fertility behaviors tend to terminate, and fertility behavior variables no longer change. Years of education was determined based on the following survey question: “What is your highest academic qualification as of your last visit?” Quality of children primarily referred to the children's years of education, which affects both the child's own economic status and the availability of resources to support their parents—more years of education was associated with higher quality of children. Finally, personal income indicated personal financial standing, including salaries, deposits, and investments, the sums of which were logarithmized.

### Models

As mentioned above, this study adopted the degree of depression as the dependent variable (scores ranged from 0 to 30). Based on the criteria for depression among the elderly established through the CES-D-10 project, different individual values were assigned as follows: (1) a value of 1 was assigned to individuals with scores ranging from 0 to 5 (not depressed), (2) a value of 2 was assigned to individuals with scores ranging from 6 to 11 (possibly not depressed), (3) a value of 3 was assigned to individuals with scores ranging from 12 to 17 (possibly depressed), (4) a value of 4 was assigned to individuals with scores ranging from 18 to 23 (probably depressed), (5) a value 5 of was assigned to individuals with scores ranging from 24 to 30 (depressed). As such, an ordered categorical variable was created to define the degree of depression. The degree of depression was then analyzed using two models.

Model 1 was the benchmark OLS model, in which the dependent variable was continuous (values ranged from 0 to 30). Regarding the endogeneity problem, younger-age fertility behaviors may affect depression later in life, but it is impossible for depression during later years to affect younger-age fertility behaviors. Therefore, no two-way causality problem existed in this study. Model 1 was defined as follows:

(1) $$Depress=\alpha_{0}Fertility+\alpha_{1}X+\varepsilon$$

where *Depress* denotes the degree of depression, *Fertility* denotes fertility behaviors, *X* denotes control variables, ε denotes the random error term, and α_0_ and α_1_ are parameter estimates.

In Model 2, the dependent variable was a five-category ordered categorical variable. The traditional OLS method was no longer suitable; therefore, a specific ordered probit model method was adopted. If the respondent's real degree of depression is y^*^, then the evaluated degree of depression is *y* (*y* ∈ [1, 2, 3, 4, 5]). In addition, assume that the latent variable is y^*^ = *xβ* + η, where *x* denotes a series of control variables (e.g., age and gender), η denotes the residual term, and the conditional probability distribution of η with respect to *x* is a standardized normal distribution. y^*^ cannot be observed directly. By setting critical values (α_1_, α_2_, α_3_, and α_4_), the observable degree of depression (*y*) can be expressed by the real degree of depression (y^*^). The following equation is thus obtained:

(2) $$y=\{\begin{array}{c} 1,y^{*}\leq \alpha_{1}\\ 2,\alpha_{1}<y^{*}\leq \alpha_{2}\\ 3,\alpha_{2}<y^{*}\leq \alpha_{3}\\ 4,\alpha_{3}<y^{*}\leq \alpha_{4}\\ 5,\alpha_{4}<y^{*} \end{array}$$

The residual η follows a conditional standardized normal distribution, so that it is possible to further infer the conditional distribution of the given *x* and *y* and calculate the response probability. For details, refer to the following equations:

(3) $$p(y=1|x)=p(y^{*}\leq \alpha_{1}|x)=\phi (\alpha_{1}-x\beta )$$

(4) $$\begin{array}{l} p(y=2|x)=p(\alpha_{1}|x<y^{*}\leq \alpha_{2}|x)\\ \;=\phi (\alpha_{2}-x\beta )-\phi (\alpha_{1}-x\beta ) \end{array}$$

(5) $$\begin{array}{l} p(y=3|x)=p(\alpha_{2}|x<y^{*}\leq \alpha_{3}|x)\\ \;\phi (\alpha_{3}-x\beta )-\phi (\alpha_{2}-x\beta ) \end{array}$$

(6) $$\begin{array}{l} p(y=4|x)=p(\alpha_{3}|x<y^{*}\leq \alpha_{4}|x)\\ \;\phi (\alpha_{4}-x\beta )-\phi (\alpha_{3}-x\beta ) \end{array}$$

(7) $$p(y=5|x)=p(y^{*}>\alpha_{4}|x)=1-\phi (\alpha_{4}-x\beta )$$

The following ordered probit model was thus constructed:

(8) $$Depress_{ij}=\beta_{1}Fertility_{ij}+\mu X_{ij}+\varepsilon_{ij}$$

where *Depress*_*ij*_ denotes the degree of depression of the *i*-th respondent in the *j*-th period, *Fertility*_*ij*_ denotes the fertility behaviors of the *i*-th respondent in the *j*-th period, *x*_*ij*_ denotes the control variables (e.g., personal and socioeconomic characteristics) of the *i*-th respondent in the *j*-th period, and ε_*ij*_ denotes the random error term.

## Results

### Basic Descriptive Analysis

Table 1 summarizes the general characteristics of the 10,553 participants (5,327 males and 5,226 females) by gender. Among the 10,553 participants, female respondents reported a significantly higher degree of depression than male respondents. The age of first childbirth was <30 years among most respondents, while at that time, male respondents were 2 years older than female respondents on average. The overall mean childbearing period was 5.71 years, without obvious gender differences. Most respondents had sons (those without sons only accounted for 13.02% of the total sample). Respondents with three or more births accounted for 52.79% of the sample. In terms of the socioeconomic status of the respondents, 97% were covered by medical insurance and 83% of them had pension insurance—there were no significant gender differences. Significant differences were found between genders in terms of years of education and personal income.

**Table 1:** Sample characteristics.

	**Total (*****N*** **= 10,553)**		**Male (*****N*** **= 5,327)**		**Female (*****N*** **= 5,226)**		
**Characteristic**	***n***	**%**	***n***	**%**	***n***	**%**	**Differences in mean**
**Degree of depression**							
0–5	4,122	39.06	2,352	44.15	1,770	33.87	−1.842^***^
6–11	4,081	38.67	2,105	39.52	1,976	37.81	
12–17	1,559	14.77	618	11.60	941	18.01	
18–23	615	5.83	205	3.85	410	7.85	
24–30	176	1.67	47	0.88	129	2.47	
**Age of first childbirth**							2.177^***^
18–20	1,315	12.46	332	6.23	983	18.81	
21–25	5,636	53.41	2,586	48.55	3,050	58.36	
26–30	2,886	27.35	1,871	35.12	1,015	19.42	
31–60	716	6.78	538	10.10	178	3.41	
**Childbearing period**							−0.188^***^
0–2	10,443	98.96	1,598	30.00	1,505	28.80	
3–5	81	0.77	1,409	26.45	1,389	26.58	
6–10	22	0.21	1,551	29.12	1,539	29.45	
11–37	7	0.07	769	14.44	793	15.17	
**Gender structure**							−0.003
No male	1,374	13.02	702	13.18	672	12.86	
Male	9,179	86.98	4,625	86.82	4,554	87.14	
**Number of births**							−0.081^***^
1	1,354	12.83	706	13.25	648	12.40	
2	3,628	34.38	1,860	34.92	1,768	33.83	
3 or more	5,571	52.79	2,761	51.83	2,810	53.77	
**Age**							0.965^***^
50–59	4,722	44.75	2,242	42.09	2,480	47.46	
60–69	3,978	37.70	2,074	38.93	1,904	36.43	
70–79	1,548	14.67	855	16.05	693	13.26	
80–93	305	2.89	156	2.93	149	2.85	
**Spouse status**							0.088^***^
No	1,305	12.37	426	8.00	879	16.82	
Yes	9,248	87.63	4,901	92.00	4,347	83.18	
**Domicile registration**							0.029^***^
Rural	7,944	75.28	3,934	73.85	4,010	76.73	
Urban	2,609	24.72	1,393	26.15	1,216	23.27	
**Education years**							2.434^***^
Illiteracy or primary school	6,937	65.73	3,026	56.80	3,911	74.84	
Junior and high school	3,424	32.45	2,155	40.45	1,269	24.28	
College or higher	192	1.82	146	2.74	46	0.88	
**Quality of children**							0.063
Illiteracy or primary school	6,937	65.73	3,026	56.80	3,911	74.84	
Junior and high school	3,424	32.45	2,155	40.45	1,269	24.28	
College or higher	192	1.82	146	2.74	46	0.88	
**Cohabitation with children**			−0.012				
No	4,880	46.24	2,495	46.84	2,385	45.64	
Yes	5,673	53.76	2,832	53.16	2,841	54.36	
**Personal income**							0.553^***^
Low	3,577	33.90	1,641	30.81	1,936	37.05	
Middle	3,456	32.75	1,685	31.63	1,771	33.89	
High	3,520	33.36	2,001	37.56	1,519	29.07	
**Medical insurance**							0.010^***^
No	310	2.94	130	2.44	180	3.44	
Yes	10,243	97.06	5,197	97.56	5,046	96.56	
**Pension insurance**							0.003
No	1,780	16.87	890	16.71	890	17.03	
Yes	8,773	83.13	4,437	83.29	4,336	82.97	

*** *p < 0.01*.

### Basic Regression Results

Table 2 shows the results of the regression modeling the effects of fertility behaviors on mental health among the elderly. The regression results of Models 1, 2, 3, and 4, respectively, describe the degree depression among the elderly arising from the age of first childbirth, childbearing period, birth gender structure, and number of births.

**Table 2:** Fertility behavior and depression among the elderly (OLS).

**Variable**	**Model (1)**	**Model (2)**	**Model (3)**	**Model (4)**
Age of first childbirth	0.044^***^ (0.015)			
Childbearing period		0.032^**^ (0.013)		
Gender structure			−0.104 (0.157)	
Number of births				0.085^*^ (0.050)
Age	−0.032^***^ (0.008)	−0.037^***^ (0.008)	−0.036^***^ (0.009)	−0.036^***^ (0.009)
Gender	1.528^***^ (0.117)	1.428^***^ (0.114)	1.424^***^ (0.114)	1.425^***^ (0.114)
Spouse status	−1.021^***^ (0.188)	−1.025^***^ (0.188)	−1.021^***^ (0.188)	−1.024^***^ (0.188)
Domicile registration	−0.688^***^ (0.138)	−0.618^***^ (0.139)	−0.634^***^ (0.141)	−0.627^***^ (0.140)
Education years	−0.059^***^ (0.016)	−0.057^***^ (0.016)	−0.058^***^ (0.016)	−0.058^***^ (0.016)
Quality of children	−0.203^***^ (0.018)	−0.193^***^ (0.018)	−0.190^***^ (0.018)	−0.189^***^ (0.018)
Cohabitation with children	−0.237^**^ (0.109)	−0.241^**^ (0.110)	−0.223^**^ (0.109)	−0.224^**^ (0.109)
Personal income	−0.195^***^ (0.018)	−0.194^***^ (0.018)	−0.195^***^ (0.019)	−0.194^***^ (0.019)
Medical insurance	−0.438 (0.340)	−0.450 (0.340)	−0.456 (0.340)	−0.456 (0.340)
Pension insurance	−0.104 (0.149)	−0.109 (0.149)	−0.107 (0.149)	−0.106 (0.149)
_cons	13.168^***^ (0.759)	14.321^***^ (0.699)	14.132^***^ (0.686)	14.172^***^ (0.694)
Observed value	10,559	10,559	10,559	10,559
*R*-square	0.085	0.085	0.085	0.085

*Standard errors are in parentheses*;

* *p < 0.1*,

** *p < 0.05*,

*** *p < 0.01. Robust standard errors are reported. The number of births was further controlled during the regression analysis of the birth gender structure*.

In Model 1, the age of first childbirth was significantly and positively correlated with the degree of depression; an increase of 1 in the age of first childbirth increased the degree of depression by ~0.05 points. Model 2 revealed a significant and positive correlation between the childbearing period and the degree of depression. Namely, an increase of 1 year in the childbearing period significantly increased the degree of depression by 0.03 points. Model 3 revealed that elderly persons in families with sons exhibited a lower degree of depression than those in families without sons. However, this difference was not significant. Model 4 revealed that an increased number of births significantly aggravated the degree of depression. That is, each new birth increased the degree of depression by ~0.09 points.

The control variables were also associated with the mental health of the respondents. For instance, increased age was associated with significantly reduced degree of depression. Specifically, an increase of 1 year of age reduced the degree of depression by 0.03 points. However, the female respondents exhibit a greater degrees of depression than the male respondents. Spousal companionship is known to have positive effects on the mental health of the elderly; older women with spouses also exhibited lower degrees of depression than those without. Further, elderly persons living in urban areas exhibited lower degrees of depression than those living in rural areas. More years of education, high quality of children, high personal income, and social insurance coverage also significantly inhibited depression. These findings are fundamentally consistent with those of Fabrizio (19), Dannefer (20), and Rueger et al. (21).

### Results of the Ordered Probit Regression

The dependent variable was ordered and categorical. Based on the standard regression presented above, an ordered probit method was used to analyze the effects of fertility behaviors on depression among the elderly. Table 3 shows the empirical analysis results. The age of first childbirth, childbearing period, and number of births were significantly and positively correlated with the degree of depression. The degree of depression was not significantly affected by whether individuals had sons. These findings are consistent with the basic regression results. Indeed, the actual living conditions of the elderly are aptly described by the following sayings: “It is more worrisome than exciting to bear sons at high ages” and “more children are not equal to more happiness.” While the degree of depression among the elderly was reduced based slightly on the presence of sons, mental health of the elderly was not significantly affected by the birth gender structure.

**Table 3:** Fertility behavior and depression among the elderly (ordered probit regression).

**Variable**	**Model (5)**	**Model (6)**	**Model (7)**	**Model (8)**
Age of first childbirth	0.007^**^ (0.003)			
Childbearing period		0.006^***^ (0.003)		
Gender structure			−0.019 (0.033)	
Number of births				0.021^**^ (0.010)
Intercept term 1	−1.264^***^ (0.150)	−1.449^***^ (0.138)	−1.437^***^ (0.139)	−1.424^***^ (0.137)
Intercept term 2	−0.172 (0.149)	−0.358^***^ (0.138)	−0.346^**^ (0.139)	−0.333^**^ (0.137)
Intercept term 3	0.543^***^ (0.149)	0.357^***^ (0.138)	0.369^***^ (0.139)	0.382^***^ (0.137)
Intercept term 4	1.270^***^ (0.151)	1.084^***^ (0.140)	1.096^***^ (0.141)	1.109^***^ (0.139)
Control variables	YES	YES	YES	YES
Observed value	10,559	10,559	10,559	10,559

*Standard errors are in parentheses*.

** *p < 0.05*,

*** *p < 0.01. Robust standard errors are reported. The number of births was further controlled during the regression analysis of the birth gender structure*.

### Robustness Test

A proxy variable was established to further increase the robustness of this study's conclusions. Specifically, this variable was set as self-rated health of the participants. The 2013 CHARLS survey asked respondents the following question: “How satisfied are you with your health?” the answers consisted of “very good,” “good,” “fair,” “poor,” and “very poor” This reflected their comprehensive self-rated health condition, which was comprised of multiple dimensions, including the physiological and psychological. Generally, respondents with better self-rated health exhibited better mental health, and vice versa. The effects of fertility behaviors on the health of elderly persons were also estimated using the probit regression models (Table 4). The regression results were consistent with those reported in the previous section. Therefore, the above conclusions are robust.

**Table 4:** Effects of fertility behaviors on self-rated health among the elderly.

**Variable**	**Model (9)**	**Model (10)**	**Model (11)**	**Model (12)**
Age of first childbirth	−0.009^*^ (0.005)			
Childbearing period				
Gender structure				
Number of births				−0.030^*^ (0.017)
Intercept term 1	−1.204^***^ (0.267)	−0.973^***^ (0.249)	−0.995^***^ (0.250)	−0.996^***^ (0.248)
Intercept term 2	1.114^***^ (0.267)	1.345^***^ (0.249)	1.322^***^ (0.250)	1.321^***^ (0.248)
Control variables	YES	YES	YES	YES
Observed value	10,551	10,551	10,551	10,551

*Standard errors are in parentheses*.

* *p < 0.1*,

*** *p < 0.01. Robust standard errors are reported. The number of births was further controlled during the regression analysis of the birth gender structure*.

### Heterogeneity Analysis

According to life course theory, an individual's overall health condition is the result of social, mental, and biological advantages and disadvantages that accumulate over time (22). Numerous studies have shown that the mental health of the elderly varies based on personal characteristics and socioeconomic factors. This study explored the heterogeneous effects of fertility behaviors on the mental health of the elderly by conducting a regression analysis based on gender, urban/rural living, and the different ages of the respondents. The results are shown in Table 5.

**Table 5:** Effects of fertility behaviors on depression among the elderly (heterogeneity analysis).

**Variable**	**Gender**		**Area**		**Age**			
	**Model (13)**	**Model (14)**	**Model (15)**	**Model (16)**	**Model (17)**	**Model (18)**	**Model (19)**	**Model (20)**
	**Female**	**Male**	**Urban**	**Rural**	**50 to 60**	**60 to 70**	**70 to 80**	**80 or more**
Age of first childbirth	0.018 (0.025)	0.063^***^ (0.019)	0.045^*^ (0.027)	0.043^**^ (0.018)	0.033 (0.028)	0.047^*^ (0.025)	0.062^*^ (0.032)	0.037 (0.047)
Childbearing period	0.053^***^ (0.020)	0.014 (0.016)	0.008 (0.023)	0.039^**^ (0.015)	0.066^***^ (0.023)	0.053^**^ (0.021)	0.002 (0.027)	−0.040 (0.044)
Gender structure	0.088 (0.238)	−0.209 (0.204)	0.154 (0.226)	−0.166 (0.211)	0.099 (0.200)	−0.381 (0.296)	−0.379 (0.547)	0.047 (1.226)
Number of births	0.126 (0.077)	0.048 (0.064)	0.006 (0.091)	0.108^*^ (0.058)	0.198^**^ (0.092)	0.136^*^ (0.081)	0.015 (0.106)	−0.083 (0.161)
Control variables	YES	YES	YES	YES	YES	YES	YES	YES
Observed value	5,222	5,329	2,615	7,936	4,724	3,976	1,547	304
R-square	0.063	0.062	0.064	0.067	0.078	0.103	0.086	0.139

*Standard errors in parentheses*.

* *p < 0.1*,

** *p < 0.05*,

*** *p < 0.01. Robust standard errors are reported. The number of births was further controlled during the regression analysis of the birth gender structure*.

As shown in model (13) and model (14), the regression results revealed that the age of first childbirth was not significantly correlated with the degree of depression among older women. However, it was significantly and positively correlated with the degree of depression among older men. Health risks generated during the physiological process of childbirth affect women in the direct sense but appear to affect the health of men through a social mechanism (23). Further, longer childbearing periods presented mental health risks to older women; namely, this factor tended to increase their degree of depression. However, the childbearing period had no significant effects on the mental health of older men. Next, the birth gender structure had no significant effects on mental health for any respondents. Nevertheless, the number of births was positively correlated with the degree of depression without any gender-based heterogeneity. The childbearing period and number of births had significant effects on the degree of depression among elderly persons in rural areas. Specifically, an increase of 1 year in the childbearing period increased the degree of depression among these respondents by ~0.04 units; each extra birth increased their degree of depression by ~0.11 units. From model (17) to model (20), the regression results revealed that the age of first childbirth was significantly and positively correlated with the degree of depression among respondents aged 60–80. Compared with respondents aged 70 and above, those aged 50–70 showed a higher degree of depression when they had experienced longer childbearing periods. A given increase in the number of births also increased the degree of depression among those aged 50–70 but had no significant effects on those aged 70 and above.

## Discussion

To the best of our knowledge, this is the first study focused on the association between fertility behavior and depression among the elderly in China. We found that the age of first childbirth, childbearing period, and number of births were significantly and positively correlated with the degree of depression. Mental health status did not vary according to whether the respondents had a son.

In this study, we found a significant positive correlation between the age of first childbirth and the degree of depression. This is consistent with a previous study that showed the degree of depression of both younger (childbearing at and before the age of 20) and older (childbearing after the age of 30) persons increases with greater age of first childbirth (24). The increase in the length of the childbearing period significantly increased the degree of depression in our cohort. This can be attributed to the ubiquitous preference for bearing sons in China. Indeed, many Chinese families choose to prolong the childbearing period in order to have sons, thus bearing children at advanced ages. For various reasons (e.g., poorer physical functioning due to advanced age and increased child-rearing costs), people who bear children during later years exhibit significantly worse mental health than those who bear children at younger ages (25). In terms of gender structure, the elderly persons with sons exhibited no better mental health status than those without. Notably, traditional preferences and circumstances still exist in China (e.g., boys are preferred over girls, while the mother's honor increases as her son's position in life improves). Families with sons may thus gain satisfaction regarding perceived social needs, therefore improving the mental health of older family members (26). However, due to improvements in social status among women and decreasing numbers of children in families, daughters have begun to provide more support for their aging parents. This has weakened the role of sons in the context of elder care. Together, these two factors have created a birth gender structure that does not significantly affect mental health among the elderly.

The results indicate that having more children is not necessarily associated with greater happiness. Three main factors contribute to this issue. First, more births implies more time spent on child rearing and more difficulties in balancing parenting and work (27). Parents are thus more likely to experience work interruptions. At the same time, social activities are reduced, thus reducing the level of social support. Indeed, social support theory posits that people without social support are more likely than people with social support to become depressed. Second, mothers who bear more children are more likely to experience women-specific diseases (2–4, 14, 28–30). The resulting cumulative changes to physical health also affect mental health of older women. Third, women with more births tend to face greater pressures due to the need to raise children during their younger years; there is also a stronger crowding-out effect on their consumption and leisure habits, thus exerting negative effects on mental health (30–33). In general, child rearing decreases the ability to engage in other opportunities, presents significant economic costs, and diminishes both personal consumption and leisure habits, thus increasing pressure on parents' mental health. As such, the sense of happiness derived from giving birth several times at younger ages does not sufficiently offset the resulting mental pressure, which increases the overall degree of depression. The average lifespan in China was 77 years of age as of 2018. During their final stages, elderly persons tend to have more in-depth understanding of life while preferring peace-of-mind and interpersonal communication over fame, wealth, power, or romance. Further, they are no longer intensely focused on material possessions.

In terms of demographic characteristics, increased age was negatively correlated with depression in the older cohort. This may be due to the optimistic mentality generally shared by the elderly, an ongoing study shows that persons of advanced age are more open-minded and exhibit better mental health than elderly persons of less advanced age (34). Older women exhibited greater depression than older men. Perhaps due to the social division of labor and expected social roles, women tend to undertake more housework and are more often engaged in long-standing family care. Women without social support are even more likely to experience depression. Further, elderly persons living in urban areas exhibited lower degrees of depression than did those living in rural areas. These urban-rural differences in the degree depression may be due to differences in economic conditions and health status (35). Compared with elderly persons in urban areas, those in rural regions experience decreased social security, loneliness, and a lower overall quality of life.

## Conclusion

We evaluated the relationship between fertility behaviors and depression among the elderly in China, using a nationally representative dataset, namely the China Health and Retirement Longitudinal Study (CHARLS). Our results revealed a strong association between fertility behaviors and depression among elderly persons in China. Specifically, the age of first childbirth was significantly and positively correlated with the degree of depression, longer childbearing periods and larger numbers of births significantly increased the degree of depression, and parents with sons exhibited no significantly better mental health status than those without. In addition, compared with elderly persons in urban areas, the childbearing period and number of births had significant effects on the degree of depression of elderly persons in rural areas. The age of first childbirth was significantly and positively correlated with the degree of depression among respondents aged 60–80.

The implication of reducing the impact of fertility behavior on depression in the elderly are in the following aspects. First, it is proposed that parents who have more than one child been provided with free birth and child-rearing knowledge training to reduce the negative emotions of stress, fear, loneliness, and worry caused by childbirth, and thus reduce the probability of chronic diseases caused by the birth of children. Second, reduce the financial burden of the families. It is recommended to provide free medical examination services, free or low-cost vaccines for women, such as cervical cancer, and to provide moderate health care services in old age; to develop inclusive childcare institutions to reduce the cost of parenting time and economic costs for families. Third, carry out family entertainment training for families with children, increase family social interaction and entertainment activities, effectively increase the time spent on entertainment and then reduce the mental stress of parenting; Fourth, reduce career risks caused by fertility behavior. It is recommended to provide paid maternity leave for women who give birth to children, increase maternity subsidies, and protect women from the risk of interrupting their work in the workplace or even being dismissed due to childbearing behaviors.

The limitations of this study are that the study is limited by its cross-sectional design; causal inferences cannot be straightforwardly drawn from these results, although we adjusted for some variables related to depression. In addition, depression was evaluated by CES-D10 and not by clinical diagnosis. However, the CES-D10 provides stable assessment of depression and is commonly used for measuring depression in the elderly (36). In the future, panel data and scientific methods such as random controlled experiments should be used to explore the relationship between fertility behavior and depression among the elderly.

## Data Availability Statement

The datasets for this study can be found in the CHARLS [http://charls.pku.edu.cn]. Please see the http://charls.pku.edu.cn, for more details.

## Author Contributions

HY and YW conceived this research. ZH was responsible for the methodology. LX conducted software analyses. XL and AZ conducted necessary validations. YW conducted a formal analysis and managed the investigation. HY and XZ gathered resources, curated all data, wrote/prepared the original draft, and were responsible for project administration. ZH and YN reviewed and edited the manuscript, were responsible for visualization, supervised the project, and acquired funding. All authors contributed to the article and approved the submitted version.

## Conflict of Interest

The authors declare that the research was conducted in the absence of any commercial or financial relationships that could be construed as a potential conflict of interest.

## Footnotes

^1^Gender structure refers to whether there are males among the children born.
