# Supplementary material for: Effects of Fertility Behaviors on Depression Among the Elderly: Empirical Evidence From China
Source: Front Public Health. 2021 Jan 26;8:570832. doi: 10.3389/fpubh.2020.570832 (PMC7870999; doi:10.3389/fpubh.2020.570832)
Supplement: Supplementary file 1 [file Data_Sheet_1.ZIP › Questionnaire.pdf]

China Health and Retirement Longitudinal Study Followup Questionnaire 2013

中国健康与养老追踪调查 2013 年追访问卷

Jan 2015

China Center for Economic Research
Peking University

Contents 目 录

B DEMOGRAPHIC BACKGROUNDS 基本信息 1

C FAMILY 家庭 21

CM LISTED HOUSEHOLD MEMBERS 21

C1 PARENT, CHILDREARING AND SIBLING INFORMATION 父母、子女、兄弟姐妹信息 22

CA PARENT INFORMATION 父母信息 22

CC SIBLINGS 兄弟姐妹 28

CG Living Arrangements preferences 居住安排偏好 28

CB CHILDREARING INFORMATION 子女信息 30

A OTHER HOUSEHOLD MEMBER 其他家户成员 48

C2 TIME TRANSFER AND TRANSFERS 家庭交往与经济帮助 51

CD TIME TRANSFER 与父母、子女间的交往 51

CE TRANSFERS 家庭得到及提供的经济帮助 53

CF TIME SPENT PROVIDING CARE 提供照料时间 61

D HEALTH STATUS AND FUNCTIONING 健康状况和功能 65

DA HEALTH STATUS 健康状况 65

DA HEALTH STATUS 健康状况 66

PART I: GENERAL HEALTH STATUS AND DISEASE HISTORY 第一部分：一般健康状况和疾病史 67

PART II: LIFESTYLE AND HEALTH BEHAVIORS 第二部分：生活方式和健康行为 85

DB FUNCTIONAL LIMITATIONS AND HELPERS 身体功能障碍以及辅助者 91

DC COGNITION & DEPPRESSION 认知和抑郁 104

E HEALTH CARE AND INSURANCE 医疗保健与保险 109

PART I MEDICAL INSURANCE 第一部分：医疗保险 109

PART II HEALTH CARE COSTS AND UTILIZATION 第二部分：医疗成本与使用情况 115

F WORK, RETIREMENT AND PENSION 工作、退休和养老金 135

FA JOB STATUS 135

FB WORK HISTORY 工作史 143

FC CURRENT PRIMARY JOB/OCCUPATION 当前、主要工作 146

FARM EMPLOYED 农业打工 146
HOUSEHOLD AGRICULTURAL WORK 自家农业生产活动 147
FD EMPLOYED 受雇 149
FE QUESTIONS ABOUT LABOR SUPPLY 劳动力供给 160
FF QUESTIONS ABOUT WAGES 工资问题 161
FG Fringe Benefits 单位福利 163
FH NON-FARM SELF-EMPLOYED AND UNPAID FAMILY BUSINESS 非农自雇和为家庭经营活动帮工 166
FI Social insurance questions for the self-employed. 自雇部分中的社会保险问题 172
FJ SIDE JOB (EMPLOYED OR SELF-EMPLOYED) 非主要职业（受雇或自雇） 175
FK UNEMPLOYMENT AND JOB SEARCH ACTIVITIES 失业，求职经历 . 175
FL LAST JOB 最近一份工作 176
FM RETIREMENT 退休与退职 182
FN PENSION INSURANCE 养老保险 192
Part 1 政府机关、事业单位退休金及企业职工基本养老保险 193
Part 2 企业补充养老保险 199
Part 3 商业养老保险 201
Part 4 人寿保险 204
Part 6 New Rural Social Pension Insurance 新型农村社会养老保险 . . 207
Part 7 征地养老保险（失地养老保险/被征地农民养老保险） 209
Part 8 高龄老人补贴 211
Part 9 其他养老保险 211

G&H INCOME, EXPENDITURES AND ASSETS 收入、支出与资产 215
G1 RELATIVE INCOME 相对收入 215
G2 HOUSEHOLD INCOME AND EXPENDITURES 家户收入与支出 216
PART 1 Household Wage Income and Individual-based transfers 家户工资收入和个人获得的转移收入 216
PART 2 HOUSEHOLD AGRICULTURAL INCOME AND EXPENDITURE 家户农业收入与支出 220
PART 3 Self-employed Activities 个体经营或开办私营企业 223
PART 4 HOUSEHOLD PUBLIC TRANSFER INCOME 家户政府转移支付收入 224

PART 5 HOUSEHOLD LIVING EXPENDITURE 家户生活支出 225
HA HOUSEHOLD ASSETS 家户资产 228
PART 1 Current Residence 第一部分：现有住宅 228
PART 2 Other Residences 第二部分：其他房产 237
PART 3 Land 第三部分：土地 242
PART 4 Equipments, Consumption durables, and Valuables. 家用设备、耐用消费品和其他贵重物品 243
HB INDIVIDUAL ASSETS 个人资产 246
PART 1 Financial Assets 第一部分：金融资产 246
PART 2 DEBTS 第二部分：债务 250

I HOUSING CHARACTERISTICS 住房情况 253

J INTERVIEWER OBSERVATION 访问员观察 257

B DEMOGRAPHIC BACKGROUNDS 基本信息

NOTE: ONLY THOSE WHO WERE BORN BEFORE JULY 1, 1968 AND THEIR SPOUSES WILL BE INTERVIEWED. **注意**：1968 年 7 月 1 日之前出生的人及其配偶需要做下列问卷。

Type of Interview R 受访者类型		
XRType = REIW	This is a reinterview R	受访者类型为回访受访者
XRType = NEWIW	This is a new interview R	受访者类型为新受访者

BA000_W2_1 We record your name is [name], is it right? 我们系统记录您的姓名是 [姓名]，对吗？

[**PROCEDURE**: Preload the name of R.]

1. Yes 是 → Skip to BA000_W2_3 跳至BA000_W2_3
2. No 否

BA000_W2_2 What's your name? 您的姓名是？ ________

BA000_W2_3 Interviewer record R's gender. 访员记录受访者性别。

[**PROCEDURE**: Preload R's gender.]

1. Male 男
2. Female 女

BA001 BRANCHPOINT:

IF XRType = REIW AND CHINESE ZODIAC SIGN IN THE LAST WAVE IS MISSING, GO TO BA001.

IF XRType = REIW AND CHINESE ZODIAC SIGN IN THE LAST WAVE IS NOT MISSING, GO TO BA002 BRANCHPOINT.

IF XRType = NEWIW, GO TO BA001.

[Show Card 出示卡片 2]

BA001 What is your Chinese Zodiac sign? 您的属相是？ _______

[IWER: Choose from the list of Chinese Zodiac signs，see Appendix 1 请选择属相，选项见附表一。]

BA002 BRANCHPOINT:

IF XRType = REIW AND DATE OF BIRTH IN THE LAST WAVE IS MISSING, GO TO BA002.

IF XRType = REIW AND DATE OF BIRTH IN THE LAST WAVE IS NOT MISSING, PRELOAD THE BIRTH TIME AND GO TO BA001_W2_1.

IF XRType = NEWIW, GO TO BA002.

BA001_W2_1 We record your birth time is [Birth Time], is it right? 我们系统记录您的出生日期是 [出生日期]，对吗？

1. Yes 是 → Skip to BA005 跳至 BA005
2. No 否

BA002 When were you born? 您的出生日期是？

____ 1900...2013 (**BA002_1**) year 年 __0...12 (**BA002_2**) month 月 __0..31 (**BA002_3**) day 日

[IWER: The year must be a number in the range [1900 – 2013]. Mark the year using four digits. Take down the month as its actual number. For example, write January as ”1” not ”01”, December as ”12”. If do not remember month and day, fill ’0’. 访员注意：年份填写范围 [1900 — 2013]。用 4 位数表示年，按照实际的月份填写月。例：1 月写作“1”，而不是“01”,12 月写作“12”。如果记不住月份和日期，请填入‘0’。]

[CAPI: Check date of birth by Zodiac.]

PROCEDURE 程序:

If the person does not know the date of birth, BA002 = DK or BA002 = RF, skip BA003.
如果受访者不知道出生日期即BA002选择不知道或拒绝回答，跳过BA003.

BA003 Is your answer to BA002 based on the solar or the lunar calendar? 您刚才回答的是公历（阳历）还是农历（阴历）？

1. Solar calendar 公历（阳历）
2. Lunar calendar 农历（阴历）

PROCEDURE 程序:

If the person does not know the date of birth, BA002 = DK or BA002 = RF, ask BA004.
如果受访者不知道出生日期即BA002 选择不知道或拒绝回答，请询问BA004

BA004 What is your age? 您今年多大年纪？ ________1...120 years old 岁

[IWER: You can refer to the year for some major events or born in which year during the republic of China. (Begin in 1912). 访员注意：可以提示受访者一些大事件发生的时间或者民国出生哪一年 (民国元年为 1912 年)。]

F1：“民国元年”为 1912 年。民国年数 +1911= 公元年

BA005 Do you go home almost every day, or attend school or work away from home but return to this home almost every week, or go home once two weeks or more than two weeks? 您是几乎每天都回家，还是平时上学/上班但通常每周末回这个家，还是两个星期及以上回一次家？

1. go home almost everyday 每天都回家
2. go home almost every weekend 平常上班/上学，周末回家
3. go home once two weeks or more than two weeks 两个星期及以上回一次家

BA006_W2_1 Are you communist? 您是共产党员吗？

1. Yes 是
2. No 否

BA007_W2_1 What's your nationality? 您的民族是？

1. Han 汉族
2. Hui 回族
3. Zhuang 壮族
4. Weiwuer 维吾尔族
5. Yi 彝族
6. Zang 藏族
7. Miao 苗族
8. Mongol 蒙古族
9. Dai 傣族
10. Other 其它 _____ (**BA007_W2_1_1**)

[INTRO: Next are some questions about your birth place, some changes in your housing location, your Hukou and education. 接下来我们将问到您的出生地、居住地的变化情况，以及您的户口和受教育程度。]

PROCEDURE 程序:

If XRType = REIW, preload the permanent address. 如果这是一个回访受访者，加载上一期的常住地址 in the last wave

If XRType = NEWIW, preload the map address. 如果这是一个新的受访者，加载本期中的绘图地址 this wave.

BB000_W2_1 According to our recorded [permanent address], what's your current permanent address? 系统记录您的常住地址是 [常住地址]，您现在的常住地址是？

1. The same address 同一个地方
2. ______(**BB000_W2_1_1**) province_city_county/city/district 省 _ 市 _ 县/市/区，
   ______(**BB000_W2_1_2**) village/neighborhood 村/社区
3. Abroad 国外

[IWER: Confirm the permanent address with respondents, and fill in the changed. If do not know the name, please fill in "other" 访员注意：请与受访者确认常住地址，并且记录变化的地址。如果不知道村、市、省的具体名称，请填入"其它"。]

[IWER: county/city includes county-level administrative units/county-level city/district. 访员注意：县/市包括县级行政单位/县级市/区.]

BB000_W2_2 What's type of the permanent address? 常住地址的类型是？

1. Family housing 家庭住宅
2. Nursing home 养老院或其他养老机构
3. Hospital 医院

4. Other ____ 其他 ____ 请注明（**BB000_W2_2_1**）

BB001 Where were you born? 您的出生地是哪里？

1. The same as permanent address 和目前常住地一样
2. Another village/neighborhood in permanent address's county/city/district 常住地所在县/市/区的其他村/社区 ____(**BB001_1**) village/neighborhood 村/社区
3. Other 其它：____(**BB001_2**) province_city_county/city/district 省 _ 市 _ 县/市/区，____(**BB001_3**) village/neighborhood 村/社区
4. Abroad 国外

[IWER: county/city includes county-level administrative units/county-level city/district. 访员注意：县/市包括县级行政单位/县级市/区.]

BB002 BRANCHPOINT:

IF XRType = REIW AND TYPE OF BIRTH PLACE IN THE LAST WAVE IS MISSING, GO TO BB002.

IF XRType = REIW AND TYPE OF BIRTH PLACE IN THE LAST WAVE IS NOT MISSING, GO TO BB003 BRANCHPOINT.

IF XRType = NEWIW, GO TO BB002.

BB002 What is the type of your birth place? Is it rural village or urban community? 您出生地的类型是什么？是农村还是城镇社区？

1. Rural Village 农村
2. Urban Community 城镇社区

PROCEDURE 程序:

If BB001 = 1, then skip to BB006 BRANCHPOINT.

If BB001 = 2, then skip to BB005.

BB003 When did you first live in permanent address's county/city/district? 您第一次搬到常住地所在县/市/区是在什么时候？ ____ 1900..2013 Year 年

[IWER: Mark the year using four digits. 访员注意：请用 4 位数表示年。]

BB004 When you first moved to permanent address, did you live in the same village/community as you currently do? 您第一次搬到常住地，就是住在当前村/社区吗？

1. Yes 是 → skip to BB006 BRANCHPOINT 跳至 BB006 跳转点
2. No 否

BB005 In what year did you first live in permanent address's village/community? 您第一次搬来常住地所在村/社区居住是在什么时候？ ____1900..2013 Year 年

[IWER: Mark the year using four digits. 访员注意：请用 4 位数表示年。]

BB006 BRANCHPOINT:

IF XRType = REIW AND BB006 IN THE LAST WAVE IS NOT MISSING, THEN SKIP TO PROCEDURE BEFORE BB007.

BB006 Where did you mainly live before you were 16 years old? Is it in village or city/town? 16 岁以前，您主要住在农村还是城镇？

[IWER: City or Village when the person was living there. 受访者当时住的时候是农村还是城镇。]

1. Village 农村
2. City/Town 城镇

PROCEDURE 程序:
If BB001 = 1 or BB001 = 2 go to BB009. 如果BB001 = 1 or BB001 = 2，跳至BB009.

BB007 Ever since you first came to permanent city/county/district, have you ever lived outside your permanent city/county/district for more than 6 months? 自从您第一次搬到常住地这个县/市/区以来，您有没有在常住地县/市/区以外的地方连续住过六个月以上？

1. Yes 是
2. No 否 → Go to BC001 请跳至BC001

BB008 Ever since you first came to your permanent county/city/district, how long have you lived outside your permanent county/city/districtin total? 自从您第一次搬到常住地所在县/市/区以来，您在其他县/市/区一共住了多长时间？ ____ (**BB008_1**) years 年 ____ (**BB008_2**) months 月

→ Skip to BB011 请跳至BB011

BB009 Have you ever lived outside your permanent county/city/district for more than 6 months? 您曾在常住地所在县/市/区以外的地方连续住过六个月以上吗？

1. Yes 是
2. No 否 → Skip to BC001 请跳至BC001

BB010 How long have you lived outside your permanent county/city/district in total? 您在常住地所在县/市/区以外的地方一共住了多长时间？

____ (**BB010_1**) years 年 ____ (**BB010_2**) months 月

[hard check: years <= 2013-BB003 此处的年份应该 <= 2013-BB003]

BB011 When did you most recently live in your permanent county/city/district (after being away for 6 months or longer)? 您最近一次搬到常住地所在县/市/区居住是在什么时候（指最近一次六个月或以上外出之后）？ ____ 1900..2013 year 年

[IWER: Mark the year using four digits. 访员注意：用 4 位数表示年。]

[hard check: year >= BB003 此处的年份应该 >= BB003]

[Show Card 出示卡片 3]

BB012 Before moving to the current permanent county/city/district, where else did you live for at least six months? (Not less than six months, otherwise ask about previous locations where the respondent lived for at least six months) 搬到常住地所在县/市/区之前，您连续住过六个月以上的地方是哪里（必须至少六个月，否则问上一个超过六个月的居住地点）？

1. Birth place 出生地 skip to BC001
2. Other 其它: ____ (**BB012_1**) province_city_county/city/district 省 _ 市 _ 县/市/区, ____(**BB012_2**) village/neighborhood 村/社区
3. Abroad 国外

[IWER: county/city includes county-level administrative units/county-level city/district. 访员注意：县/市包括县级行政单位/县级市/区]

BB013_W2_1 Why did you live in other county/city? 您住在其他县/市或其他市的原因是?

1. School 上学
2. Marriage 结婚
3. Look after relatives 照顾亲戚
4. Live in children's house 住在儿女家
5. Travel 旅游
6. Work 工作
7. In the hospital 住院
8. Other 其他 ________ （**BB013_W2_1_1**）

BC001 What is your current HuKou status? 目前您的户口类型是?

1. Agricultural Hukou 农业
2. Non-agricultural Hukou 非农业
3. Unified Residence Hukou 统一居民户口
   [IWER: for the place where agricultural hukou is abandoned. 访员注意：对取消农村户口的地方]
4. Do not have Hukou 没有户口 → Skip to procedure before BD001_W2_1 跳至BD001_W2_1 之前的程序
   F1：“统一居民户口”指的是某些地方实行户口制度改革后，不再区分农业与非农业户口，而是统一为“居民户口”。

PROCEDURE 程序:
IF XRType = NEWIW, skip to procedure before BC002.

PROCEDURE 程序: Preload the answer of BC001 in last wave, then compare the two answers. 加载上一期BC001的答案与这期的答案进行对比.
If they are different then ask BC001_W2_1. 如果两次的答案不一样，询问BC001_W2_1。
If they are the same then skip to BC005_W2_1 BRANCHPOINT. 如果两次答案一样，跳至BC005_W2_1 跳转点。

BC001_W2_1 We record your hukou status is [hukou status] in last wave, your hukou status has changed during this two years, right? 我们系统记录您上一次访问时的户口类型是 [户口类型]，您的户口类型两年期间发生了改变，对吗?

1. Yes 是

2. No 否, 上一次访问数据错误 → SKIP TO BC005_W2_1 BRANCHPOINT 跳至 BC005_W2_1 跳转点

PROCEDURE 程序:
If BC001 = 1 then ask BC001_W2_2. 如果BC001 = 1, 则询问BC001_W2_2
If BC001 = 2 then ask BC001_W2_3. 如果BC001 = 2, 则询问BC001_W2_3
If BC001 = 3 then ask BC001_W2_4. 如果BC001 = 3, 则询问BC001_W2_4

BC001_W2_2 What is the reason that you changed to the agricultural Hukou? 您变成农业户口是因为什么情况?

1. live in rural areas aftergraduate from college 大学上完后回到农村居住
2. marriage 结婚
3. employment 就业
4. migration of the whole village 整村搬迁
5. No change 没有变化 → SKIP TO BC005_W2_1 BRANCHPOINT
6. others, pls specify ____ 其它, 请注明 ____ (**BC001_W2_2_1**)

PROCEDURE 程序:
SKIP TO BC001_W2_4

BC001_W2_3 What is the reason that you changed to the non-agricultural Hukou? 您变成非农业户口是因为什么情况?

1. Go to a college 上学
2. marriage 结婚
3. employment 就业
4. Land is acquired by the government 土地被征用
5. migration of the whole village 整村搬迁
6. No change 没有变化 → SKIP TO BC005_W2_1 BRANCHPOINT
7. others, pls specify ____ 其它, 请注明 ____ **(BC001_W2_3_1)**

BC001_W2_4 When did you change the status of Hukou? 您什么时候改的户口类型? ____ year 年

PROCEDURE 程序:
IF XRType = REIW, SKIP TO BC005_W2_1 BRANCHPOINT.

PROCEDURE 程序:
If BC001 = 3, ask BC002. 如果BC001 = 3, 询问BC002到BC003 to BC003.

BC002 What is your Hukou status before you have the unified residence Hukou? 您在获得统一居民户口之前是什么户口?

1. Agricultural Hukou 农业户口
2. Non-agricultural Hukou 非农业户口

3. Do not have Hukou 没有户口
   F1：“统一居民户口”指的是某些地方实行户口制度改革后，不再区分农业与非农业户口，而是统一为“居民户口”。

BC003 When did you have the unified residence Hukou? 您什么时候获得统一居民户口？
____ (**BC003_1**) 1900...2013 year 哪年 ____ (**BC003_2**) 0...12 month 月

PROCEDURE 程序:
Go to BC005_W2_1 BRANCHPOINT. 请跳至BC005_W2_1 跳转点

BC004 When did you get your current Hukou? 您是什么时候得到目前的这个户口？
____ 1900...2013 (**BC004_1**) year 年 ____ 0...12 (**BC004_2**) month 月
[hard check: enforce year >= BA002_1 此处的年份应该 >= BA002_1 (出生年份)]
[IWER: You must fill year [1900 - 2013]. Mark the year using four digits. Take down the month as its actual number. For example, write January as ”1” not ”01”, December as ”12”. If do not remember month, fill ’0’. 访员注意：年份填写范围 [1900-2013]。用 4 位数表示年，按照实际的月份填写月。例：1 月写作“1”，而不是“01”，12 月写作“12”。如果记不住月份，请填入‘0’。]

BC005_W2_1 BRANCHPOINT:
IF XRType = REIW AND HUKOU LOCATION IS NOT MISSING IN THE LAST WAVE, GO TO BC005_W2_1.
IF XRType = REIW AND HUKOU LOCATION IS MISSING IN THE LAST WAVE, GO TO BC005.
IF XRType = NEWIW, GO TO BC005.

BC005_W2_1 Since last interview, have your Hukou location changed? 自从上次访问以来，户口所在地有没有改变？
1. Yes 有
2. No 没有 → skip to procedure before BD001_W2_1 跳至 BD001_W2_1 之前的程序

[CAPI: Preload the permanent address.]

BC005 Where is your current HuKou location? 目前您的户口所在地是？
[IWER: If birthplace is R’s permanent address, pls choose the first choice. 如果出生地是常住地址，此处请选择第一个选项]
1. Permanent address 常住地址
2. Birthplace 出生地
3. Another village/neighborhood in permanent address county/city/district 常住地所在县/市/区的其他村/社区 ___(**BC005_1**) village/neighborhood 村/社区
4. Other 其它：____ (**BC005_2**) province_city_county/city/district 省 _ 市 _ 县/市/区，____ (**BC005_3**) village/neighborhood 村/社区

[IWER: county/city includes county-level administrative units/county-level city/district. 访员注意: 县/市包括县级行政单位/县级市/区]

**BC005_W2_2 BRANCHPOINT**:<br>IF XRType = REIW AND BC005_W2_1 = 1, GO TO BC005_W2_2.<br>IF XRType = NEWIW, GO TO THE PROCEDURE BEFORE BC006.

BC005_W2_2 Why the location of current Hukou is different from your lastwave? 为什么现在户口所在地与上一次访问时不一样?

1. marriage 结婚
2. go to school 上学
3. employment 就业
4. retirement/revolutionary retirement 退休/离休
5. migration of the whole village 整村搬迁
6. others, pls specify____ 其它,请注明 ____ (**BC005_W2_2_1**)

BC005_W2_3 When did you change the location of Hukou? 您什么时候改的户口地址? ________1900...2013 year 年

**PROCEDURE 程序**:<br>If (BC005 = 2 and BB001 = 3/4) or BC005 = 4, please ask BC006. 如果 (BC005 = 2 且 BB001 = 3/4) 或 BC005 = 4, 请询问BC006

BC006 How long have you been away from your current Hukou county/city/distric? 您离开目前户口所在县/市/区的时间有多久了? ____ (**BC006_1**) 1900...2013 Years 年 ____ (**BC006_2**) ___0...12 Months 月

[hard check: impose that years <= 2013-BA002_1 (birth year) 此处的年份应该 <= 2013-BA002_1 (出生年份)]

**PROCEDURE 程序**:<br>IF XRType = REIW, skip to the procedure before BD001_W2_1. 回访受访者跳至BD001_W2_1 之前的程序。

BC007 Is your first Hukou the same as your current Hukou? (Not including the change of unified Residence Hukou.) 您的第一个户口就是您目前的户口吗?(统一居民户口的变化不算变化。)

1. Yes 是
2. No 否

BC008 Were there any other Hukou between your first Hukou and your current Hukou? (Including changes of hukou type and place. Hukou type change only means changes between Agricultural Hukou and non-agricultural hukou.) 第一个户口与目前户口之间,您还有过其他户口吗?(户口变化指户口类型或户口所在地的变化,其中户口类型变化只指农业与非农业户口的变化。)

1. Yes 有
2. No 没有

PROCEDURE 程序:
If BC007 = 1 and BC008 = 2, go to the procedure before BD001_W2_1. 如果 BC007 = 1 并且BC008 = 2, 跳至 BD001_W2_1 之前的程序

BC009 Has your Hukou type or place ever changed since your first Hukou? (Hukou type change only means changes between Agricultural Hukou and non-agricultural hukou.) 从第一个户口开始，您的户口类型或户口所在地发生过什么变化？(户口类型变化指农业与非农业户口的变化。)
1. Both Hukou type and place have changed 户口类型和户口所在地都变化过
2. Only Hukou type has changed 只有户口类型变化
3. Only Hukou place has changed 只有户口所在地变化

PROCEDURE 程序:
If BC009 = 1/2, ask BC010. 如果 BC009 = 1/2, 提问BC010。
If BC009 = 3. skip BC010. 如果 BC009 = 3. 跳过 BC010

BC010 What was your first HuKou status? 您拿到的第一个户口的类型是？
1. Agricultural Hukou 农业
2. Non-agricultural Hukou 非农业

PROCEDURE 程序:
If BC008 = 1 and (BC009 = 1/3), ask BC011, else skip BC011. 如果BC008 = 1 且 BC009 = 1/3, 提问BC011, 否则跳过BC011

[CAPI: Preload the permanent address]

BC011 Where was your last HuKou location? 您上一个户口所在地是？
[IWER: If last HuKou location is R's birthplace, and both of them are in the county/city of R's permanent address, pls choose the first choice. 如果 R 的上一个户口所在地就是出生地，并且都在常住地所在县市，此处请选择第一个选项]
1. Birthplace 出生地
2. Permanent address 常住地
3. Another village/neighborhood in permanent address county/city/district 常住地所在县/市/区的其他村/社区 ____(**BC011_1**) village/neighborhood 村/社区
4. Other 其它: ____ (**BC011_2**) province_city_county/city/district 省 _ 市 _ 县/市/区, ____ (**BC011_3**) village/neighborhood 村/社区

[IWER: county/city includes county-level administrative units/county-level city/district. 访员注意: 县/市包括县级行政单位/县级市/区]

PROCEDURE 程序:
If BC011 = 2/3/4, ask BC012 , else go to the procedure before BC013. 如果BC011 = 2/3/4, 提问BC012, 否则跳至BC013前的程序控制。

BC012 Why the location of last Hukou different from your birth Hukou location? 为什么上一个户口所在地与出生地不一样？

1. marriage 结婚
2. go to school 上学
3. employment 就业
4. retirement/ revolutionary retirement 退休/离休
5. escape of famine 逃荒
6. sent down to the countryside to do manual labor 上山下乡
7. migration of the whole village 整村搬迁
8. With the parents migration 随迁
9. others, pls specify____ 其它，请注明 ____ （**BC012_1**）

PROCEDURE 程序:
If BC009 = 2, go to procedure before BC015. 如果BC009 = 2, 跳至BC015前的程序控制

[CAPI: Preload the permanent address.]

BC013 What was the location of your first HuKou? 您第一个户口的所在地是？
[IWER: If the location of R's first hukou is the same as R's birthplace/current hukou/permanent address, the choose the first answer. 访员注意：如果第一个户口所在地与出生地、目前户口所在地、常住地址都一样，那么请选 1]

1. Birthplace 出生地
2. Same as current Hukou 与目前户口所在地一样
3. permanent address 常住地址
4. Another village/neighborhood in permanent address county/city/district 在常住地址所在县/市/区的其他村/社区 ____(**BC013_1**) village/neighborhood 村/社区
5. Other 其它: ____ (**BC013_2**) province_city_county/city/district 省 _ 市 _ 县/市/区, ____ (**BC013_3**) village/neighborhood 村/社区

[IWER: county/city includes county-level administrative units/county-level city/district. 访员注意: 县/市包括县级行政单位/县级市/区]

PROCEDURE 程序:
If BC001 = 1 and BC009 = 1/2, ask BC015 to BC016, then skip to procedure before BD001_W2_1. 如果BC001 = 1 且 BC009 = 1/2, 询问BC015至BC016 ，然后跳至BD001_W2_1之前的程序。

BC015 What is the reason that you had the non-agricultural Hukou? 您为什么以前有非农业户口况？

1. sent down during 1967-1977 and did not come back to city 1967-1977 年之间下乡以前是非农业户口
2. was a student 上学
3. marriage 结婚

4. employment 就业
5. migration of the whole village 整村搬迁
6. escape of famine 逃荒
7. others, pls specify____ 其它，请注明 ____ (**BC015_1**)

BC016 When did you have the non-agricultural Hukou? 您什么时候有非农业户口？
From ____(**BC016_1**) 1900...2013 year to ____(**BC016_2**) 1900...2013 year 从哪年到哪年

**PROCEDURE 程序:**
If BC001 = 2 and BC009 = 1/2, ask BC017 to BC018. 如果BC001 = 2 且 BC009 = 1/2, 询问BC017到BC018。

BC017 What is the reason that you had the agricultural Hukou? 您为什么以前有农业户口？
1. sent down during 1967-1977 and then come back to city 1967-1977 年之间下乡
2. born in rural areas 出生在农村
3. marriage 结婚
4. employment 就业
5. escape of famine 逃荒
6. others, pls specify ____ 其它，请注明 ____(**BC017_1**)

BC018 When did you have the agricultural Hukou? 您什么时候有农业户口？
From______(**BC018_1**) 1900...2013 year to ______(**BC018_2**) 1900...2013 year 从哪年到哪年

**PROCEDURE 程序:**
If XRType = NEWIW, ask BD001.<br>If XRType = REIW, and BD001 in last wave is missing, ask BD001.<br>If XRType = REIW, and BD001 in last wave is not missing, ask BD001_W2_1.

[CAPI: Preload the R's highest level of education in last wave]

BD001_W2_1 We record your highest level of education is [education level] in last wave, is it right? 我们系统记录上一次访问时您的最高学历是 [最高学历]，对吗？
1. Yes 是
2. No 否 → skip to BD001_W2_3 跳至BD001_W2_3

BD001_W2_2 Is the highest level of education contains adult education in last wave? 上一期回答的最高学历是否包括成人教育？
1. Yes 是
2. No 否 → skip to BD001_W2_4

BD001_W2_3 What's the highest level of education in last wave? (not including adult education) 您上一次访问时的最高学历是什么（不包括成人教育）？

1. No formal education (illiterate) 未受过教育（文盲）
2. Did not finish primary school but capable of reading and/or writing 未读完小学，但能够读、写
3. Sishu/home school 私塾
4. Elementary school 小学毕业
5. Middle school 初中毕业
6. High school 高中毕业
7. Vocational school 中专（包括中等师范、职高）毕业
8. Two-/Three-Year College/Associate degree 大专毕业
9. Four-Year College/Bachelor's degree 本科毕业
10. Master's degree 硕士毕业
11. Doctoral degree/Ph.D. 博士毕业

BD001_W2_4 Have your highest level of education changed from last wave? If so, what is the highest level of education you have attained now? (not including adult education) 自从上次访问以来，您的最高学历发生变化了吗？如果有变化，您现在获得的最高学历是？（不包括成人教育）

1. No formal education (illiterate) 未受过教育（文盲）→ skip to the procedure before BD007_W2_1 跳至BD007_W2_1 之前的程序
2. Did not finish primary school but capable of reading and/or writing 未读完小学，但能够读、写
3. Sishu/home school 私塾
4. Elementary school 小学毕业
5. Middle school 初中毕业
6. High school 高中毕业
7. Vocational school 中专（包括中等师范、职高）毕业
8. Two-/Three-Year College/Associate degree 大专毕业
9. Four-Year College/Bachelor's degree 本科毕业
10. Master's degree 硕士毕业
11. Doctoral degree/Ph.D. 博士毕业
12. No change 没有变化

PROCEDURE 程序:
Skip to the procedure before BD002

BD001 What is the highest level of education you have attained? (not including adult education) 您现在获得的最高学历是？（不包括成人教育）

1. No formal education (illiterate) 未受过教育（文盲）→ Skip to the procedure before BD007_W2_1 请跳至BD007_W2_1 之前的程序
2. Did not finish primary school but capable of reading and/or writing 未读完小学，但能够读、写
3. Sishu/home school 私塾

4. Elementary school 小学毕业
5. Middle school 初中毕业
6. High school 高中毕业
7. Vocational school 中专（包括中等师范、职高）毕业
8. Two-/Three-Year College/Associate degree 大专毕业
9. Four-Year College/Bachelor's degree 本科毕业
10. Master's degree 硕士毕业
11. Doctoral degree/Ph.D. 博士毕业

PROCEDURE 程序:
If BD001 = 3 or BD001_W2_4 = 3, ask BD002. 如果BD001 = 3 或者 BD001_W2_4 = 3, 提问BD002。

BD002 What is the highest grade did you finish in primary school? 您小学读到 __1...6 年级?

PROCEDURE 程序:
If BD001 = 4 or BD001_W2_4 = 4, ask BD002_W2_1. 如果BD001 = 4 或者 BD001_W2_4 = 4, 提问BD002_W2_1

BD002_W2_1 How many years did you spend in sishu/home school? 您上过几年私塾? __ 年

PROCEDURE 程序:
If BD001 >= 5 or BD001_W2_4 >= 5, ask BD003. 如果BD001 >= 5 或者 BD001_W2_4 >= 5, 提问BD003.

BD003 How many additional years of schooling did you complete after [THE ANSWER CHOSEN IN BD001]? 您在 [BD001的答案] 之后又上过几年级学? ____ years 年

PROCEDURE 程序:
If BD001 > 7 or BD001_W2_4 > 7, ask BD004. 如果 BD001 > 7 或者 BD001_W2_4 > 7, 提问 BD004。
If XRType = REIW and BD004 is not missing in last IW, skip to the procedure before BD005. 如果 XRType = REIW 且上一期BD004 不缺失, 跳至 BD005 之前的程序。
If XRType = REIW and BD004 is missing in last IW, ask BD004. 如果 XRType = REIW 且上一期BD004 缺失, 提问 BD004。
If XRType = NEWIW, ask BD004. 如果 XRType = NEWIW, 提问 BD004。

BD004 When did you go to college? 您从哪一年开始读大学/大专? ____1900...2013 year 年

PROCEDURE 程序:
IF XRType = REIW and BD005 is not missing in last IW, skip to BD006. 如果 XRType = REIW 且上一期 BD005 不缺失, 跳至 BD006。
IF XRType = REIW and BD005 is missing in last IW, ask BD005. 如果 XRType = REIW 且上一期 BD005 缺失, 提问 BD005.
IF XRType = NEWIW, ask BD005. 如果 XRType = NEWIW, 提问 BD005.

BD005 At what age did you begin formal schooling? 您从几岁开始上小学/私塾？ _____1…120 years old 岁 [soft check, < 6, > 11]

BD006 At what age did you finish schooling? 您几岁读完书？ ____1…120 years old 岁 [IWER: It asks age when R finishes schooling, not age when R finished elementary school. 访员注意：此处询问的是停止上学的年龄，并非读完小学/私塾的年龄]

**BD007_W2_1 BRANCHPOINT**:
IF XRType = REIW AND BD007 IN THE LAST WAVE IS NOT MISSING, GO TO BD007_W2_1.
IF XRType = REIW AND BD007 IN THE LAST WAVE IS MISSING, GO TO BD007.
IF XRType = NEWIW, GO TO BD007

BD007_W2_1 Have you attended school for adult education since last interview? 从上次访问以来，您参加过成人教育（如电大，夜校，自考，函授，扫盲班，速成班）吗？(可多选)

1. None 没有 → Go to BE001 请跳至BE001
2. TV University 电大 → Go to BD008_W2_1
3. Night School 夜校 → Go to BD008_W2_1
4. Zikao (examinations for self-taught students) 自考 → Go to BD008_W2_1
5. Hanshou/Correspondence course/Distance learning 函授 → Go to BD008_W2_1
6. Literacy course 扫盲班 → Go to BD008_W2_1
7. Accelerated education course 速成班 → Go to BD008_W2_1
8. Other (explain:) 其他，请注明 ____(**BD007_W2_1**) → Go to BD008_W2_1

BD007 Have you ever attended school for adult education? 您曾经参加过成人教育（如电大，夜校，自考，函授，扫盲班，速成班）吗？(可多选)

1. None 没有 → Go to BE001 请跳至BE001
2. TV University 电大
3. Night School 夜校
4. Zikao (examinations for self-taught students) 自考
5. Hanshou/Correspondence course/Distance learning 函授
6. Literacy course 扫盲班
7. Accelerated education course 速成班
8. Other (explain:) 其他，请注明 ____(**BD007_1**)

BD008 How many years did you spend in adult education? 您成人教育一共读了几年？
______year 年 [soft check > 9] → GO TO BD009

BD008_W2_1 How many years did you spend in adult education since last interview? 从上次访问以来，您成人教育一共读了几年？
______year 年 [soft check > 2] → GO TO BD009_W2_1

BD009 Did you get a diploma or degree from the adult education program you attended? 您从成人教育中获得了学位或文凭吗？

1. Yes 是 → GO TO BD010
2. No 否 → GOTO BE001 跳至BE001

BD009_W2_1 Did you get a diploma or degree from the adult education program you attended since last interview? 从上次访问以来，您从成人教育中获得了学位或文凭吗？

1. Yes 是 → GO TO BD010
2. No 否 → GOTO BE001 跳至BE001

BD010 When did you receive the diploma? 您什么时候获得该学位或者文凭？

______1900...2013 year 年

BD011 BD011] What is the highest level of schooling you obtained from the adult education program? 您获得最高的成人教育学位或文凭是什么？

1. Vocational school 中专
2. Two/Three Year College / Associate degree 大专
3. Four Year College / Bachelor's degree 本科
4. Others 其他

[Show Card 出示卡片 1]

BE001 RMaritalStatus: What is your marital status? 您目前的婚姻状态？

[IWER: common-law marriage is considered as married, 即使没有领结婚证但自称结婚者也可视为已婚]

1. Married with spouse present 已婚与配偶一同居住
2. Married but not living with spouse temporarily for reasons such as work 已婚，但因为工作等原因暂时没有跟配偶在一起居住
3. Separated 分居（不再作为配偶共同生活）
4. Divorced 离异
5. Widowed 丧偶
6. Never married 从未结婚
7. Cohabitated 同居

PROCEDURE 程序:

If BE001 = 6, then skip to BF008. 如果 BE001 = 6，请跳至 BF008.

[INTRO: Many people have more than one marriage through whole life. Please bear me a few more questions on this. 很多人一生中不止结过一次婚，所以，我们想请您回答以下这些婚姻题目]

PROCEDURE 程序:

IF XRType = REIW, ask BE003_W2_1

IF XRType = NEWIW, ask BE003

BE003 How many times have you been married? 您到目前为止结过几次婚？____ times 次 [soft check > 2]

PROCEDURE 程序:
IF XRType = NEWIW, skip to the procedure before BE004.

BE003_W2_1 How many times have you been married since last interview? 从上次访问以来您结过几次婚? ____ times 次 [soft check > 2]

PROCEDURE 程序:
If BE003 = 1, continue with BE004 - BE005, then go to the procedure before BF001. 如果受访者只结过一次婚，继续回答BE004 - BE005，然后跳至BF001之前的程序。
If BE003_W2_1 = 0, go to the procedure before BF001. 如果BE003_W2_1 = 0，跳至BF001之前的程序。
If BE003 > 1, continue with BE009. 如果BE003 > 1，继续提问 BE009.
If BE003_W2_1 > 0, continue with BE009. 如果BE003_W2_1 > 0，继续提问 BE009.

BE004 When did you get married? 你什么时候结的婚?
_____1900..2013 (**BE004_1**) year 年 ____ 0..12 (**BE004_2**) month 月 [soft check: year > BA002_1 + 16, 此处的年份应该 >BA002_1 + 16]
[IWER: You must fill year[1900 - 2013]. Mark the year using four digits. Take down the month as its actual number. For example, write January as ”1” not ”01”, December as ”12”. If do not remember month, fill ’0’. 访员注意：年份填写范围 [1900 - 2013]。用 4 位数表示年，按照实际的月份填写月。例：1 月写作“1”，而不是“01”，12 月写作“12”。如果记不住月份，请填入‘0’。]

BE005 When you were married, what was the total value of cash and goods (including housing) that your parents gave to you and your spouse? 您结婚时，在当年的物价水平下，您父母总共给了您和您的配偶多少价值的现金和物品（包括房子）? ______Yuan 元

PROCEDURE 程序:
Skip to procedure before BF001 跳至BF001前的程序控制

BE009 When was your most recent marriage? 你最近一次是什么时候结的婚?
_____1900...2013 (**BE009_1**) year 年 ____ 0...12 (**BE009_2**) month 月
[hard check: enforce year>=BE007, 此处的年份应该 >=BE007]
[IWER: You must fill year[1900 - 2013]. Mark the year using four digits. Take down the month as its actual number. For example, write January as ”1” not ”01”, December as ”12”. If do not remember month, fill ’0’. 访员注意：年份填写范围 [1900-2013]。用 4 位数表示年，按照实际的月份填写月。例：1 月写作“1”，而不是“01”，12 月写作“12”。如果记不住月份，请填入‘0’。]

[IWER: I’d like to ask you a few questions about your current (or most recent) spouse/partner. 访员注意：这里有几个关于受访者配偶的问题。若受访者属于结婚多次，则问最近一次婚姻的配偶]

[INTRO: An important part of this study is understanding how people make decisions during different stages of life, we also need to a general age range for your spouse. 了解人们在不同的人生阶段如何进行决策是我们研究的一个重要方面，因此接下来我们会问到您配偶的年龄]

BF001 BRANCHPOINT:
IF THIS R HAS SPOUSE (BE001 = 1/2/7), THEN SKIP TO BF008.
IF R DID NOT CHANGE HIS/HER SPOUSE (BE003_W2_1=0) AND SPOUSE/PARTNER'S CHINESE ZODIAC SIGN IS NOT MISSING AT LAST WAVE, SKIP TO BF002 BRANCHPOINT.

[Show Card 出示卡片 2]

BF001 What is your spouse/partner's Chinese Zodiac sign? 您配偶的属相是？____
[IWER: Choose from the list of Chinese Zodiac signs, see Appendix 1. 请选择属相，选项见附表一。]

BF002 BRANCHPOINT:
IF THIS R DID NOT CHANGE HIS/HER SPOUSE (BE003_W2_1=0) AND SPOUSE/PARTNER'S BIRTH DATE IS NOT MISSING AT LAST WAVE, SKIP TO BF004 BRANCHPOINT.

BF002 When was your spouse/partner born? 您配偶的出生日期是？
______1900...2013 (**BF002_1**) year 年 ____0...12 (**BF002_2**) month 月 [preload spouse's birthdate]
[IWER: Mark the year using four digits. Take down the month as its actual number. For example, write January as "1" not "01", December as "12". If do not remember month, fill '0'. 访员注意：用 4 位数表示年，按照实际的月份填写月。例：1 月写作"1"，而不是"01"，12 月写作"12"。如果记不住月份，请填入'0'。]

BF003 Is your spouse/partner's date of birth based on the solar calendar or the lunar calendar? 您刚才答的是公历（阳历）还是农历（阴历）？
1. Solar calendar 公历（阳历）
2. Lunar calendar 农历（阴历）

BF004 What is the highest level of education your spouse/partner has attained? (not including adult education) 您现在获得的最高学历是？(不包括成人教育)
1. No formal education (illiterate) 未受过教育（文盲）
2. Did not finish primary school but capable of reading and/or writing 未读完小学，但能够读、写
3. Sishu/home school 私塾
4. Elementary school 小学毕业
5. Middle school 初中毕业
6. High school 高中毕业
7. Vocational school 中专（包括中等师范、职高）毕业
8. Two-/Three-Year College/Associate degree 大专毕业
9. Four-Year College/Bachelor's degree 本科毕业
10. Master's degree 硕士毕业

11. Doctoral degree/Ph.D. 博士毕业

**BF005 BRANCHPOINT**:
IF (BE001 = 3), THEN ASK BF005.

BF005 When did you separate? 你们什么时候分居的？

____ 1900...2013 (**BF005_1**) year 年 __0...12 (**BF005_2**) month 月 [hard check: enforce year > year married BE004 IF BE003 = 1) BE009 IF BE003 > 1)

[IWER: Mark the year using four digits. Take down the month as its actual number. For example, write January as ”1” not ”01”, December as ”12”. If do not remember month and day, fill ’0’. 访员注意：用 4 位数表示年，按照实际的月份填写月。例：1 月写作“1”，而不是“01”,12 月写作“12”。如果记不住月份和日期，请填入‘0’。]

**BF006 BRANCHPOINT**:
IF (BE001 = 4), THEN ASK BF006.

BF006 When did you divorce? 你们什么时候离婚的？

____ 1900...2013 (**BF006_1**) year 年 __0...12 (**BF006_2**) month 月 [hard check: enforce year > year married BE004 IF BE003 = 1) BE009 IF BE003 > 1)

[IWER: Mark the year using four digits. Take down the month as its actual number. For example, write January as ”1” not ”01”, December as ”12”. If do not remember month and day, fill ’0’. 访员注意：用 4 位数表示年，按照实际的月份填写月。例：1 月写作“1”，而不是“01”,12 月写作“12”。如果记不住月份和日期，请填入‘0’。]

BF006_W2_1 What is the reason of your divorce? 您离婚的原因是？

1. Emotional feud 感情不和
2. Live in different places 两地分居
3. In order to facilitate the property purchase transactions 为了便利不动产等购买交易
4. Other 其他原因，请注明 ____(**BF006_W2_1_1**)

BF006_W2_2 Divided assets, including real estate, how much is yours and how much is your ex-wife/husband? 关于双方共同财产（包括不动产、汽车等）分割情况，以货币价值计算，你得到 ____ (**BF006_W2_2_1**) 元，前夫/妻得到 ____ (**BF006_W2_2_2**) 元

BF006_W2_3 Do you have any infancy children? Owed to whom? 有未成年的孩子吗？归谁抚养？

1. Myself 我自己
2. My last spouse 我前夫/妻
3. None → Skip to BF007

BF006_W2_4 How much money you or your ex-wife/husband pay for upbringing the children? 你或者你的前夫/前妻付给抚养孩子一方多少抚养费？ ______Yuan 元

**BF007 BRANCHPOINT**:
IF (BE001 = 5), THEN ASK BF007.

BF007 When did your spouse pass away? 您配偶是什么时候去世的?

____ 1900...2013 (**BF007_1**) year 年 __0...12 (**BF007_2**) month 月 [hard check: enforce year > year married BE004 IF BE003 = 1) BE009 IF BE003 > 1)

[IWER: Mark the year using four digits. Take down the month as its actual number. For example, write January as "1" not "01", December as "12". If do not remember month and day, fill '0'. 访员注意：用 4 位数表示年，按照实际的月份填写月。例：1 月写作"1"，而不是"01"，12 月写作"12"。如果记不住月份和日期，请填入'0'。]

BF008 How often did the respondent receive assistance in answering section Demographics? 受访者填写该部分问卷时是否求助?

[IWER: If it is answered by a proxy, please record the respondent's reaction. 访员注意：如果是协助回答，请记录受访者的反应。]

1. Never 从未 → Skip to next module 跳至下一模块
2. A few times 偶尔几次 → Skip to next module 跳至下一模块
3. Most or all of the time 大多数 → Skip to next module 跳至下一模块
4. The section was completed by a proxy respondent 请人代填 → Skip to next module 跳至下一模块

BF009 What is your relationship to R? 您和受访者是什么关系?

[IWER: What is the proxy's relationship to R? If unknown, please ask the proxy. 访员注意：代填问卷的人和受访者是什么关系。如果说不清楚，请问代填者。]

1. Spouse 配偶
2. Mother 母亲
3. Father 父亲
4. Mother-in-law 岳母/婆婆
5. Father-in-law 岳父/公公
6. Sibling 兄弟姐妹
7. Brother-in-law, sister-in-law 姐夫妹夫/嫂子弟媳
8. Child 孩子
9. Spouse of child 孩子的配偶
10. Grandchild 孙子女
11. Other relative 其他亲戚
12. Helper or other non-relative 帮忙的人或者其他非亲属

BF010 What is the main reason for proxy (the respondent is absent)? 受访者不在场，请人代填的主要原因是什么?

1. The respondent has serious physical handicaps 受访者有严重身体障碍
2. The respondent has serious mental handicaps 受访者有严重精神障碍
3. The respondent has rejected this interview 受访者拒访
4. Other 其他，请注明 ____(**BF010_1**)

C FAMILY 家庭

Type of Interview R 受访者类型		
XRType = REIW	This is a reinterview R	受访者类型为回访受访者
XRType = NEWIW	This is a new interview R	受访者类型为新受访者

CM LISTED HOUSEHOLD MEMBERS

PROCEDURE 程序:

If this is a reinterview household, please preload the household member list in last wave, if the number of household member is "0", skip to CM002_W2_1. 如果这是一个回访家户，加载上一期记录的家户成员名单，如果家户成员个数为"0"，跳至CM002_W2_1.

If this is a new household, please preload the household member list recorded in coverscreen, if the number of household members is "0", skip to CM002_W2_1. 如果这是一个新的家户，加载本期中过滤问卷中的人员名单，如果人员个数为"0"，则跳至CM002_W2_1.

[INTRO: Types of household members are as followed: People who live together (in respondent's permanent address) and share daily expenses; parents who rotate amongst their children but stay mostly in this household; those who do not live here, but live in dormitories, workplaces, or other places that are not normal residential areas; live-in nannies, drivers or any other service people. 家户成员的类型包括：住在这个家（被访者的常住地址），满足共担生活费用的条件；轮流赡养老人，在这家住的时间最长；因工作、上学等原因住在集体宿舍或单位住房，而不是住在居民区的人；住家的保姆或司机或其他服务人员。]

CM001_W2_1 I will go through a list of people living in this household that our system recorded. Please let me know whether this list is correct. 这是我们系统记录的您家的家户成员列表。我想跟您确认这个名单对不对。

[IWER: MARK THE MEMBER THAT LIVE IN THE HH. 访员注意：请勾选家户成员名单。]

1. Prev wave hhmember[1] 家户成员名单列表
2. Prev wave hhmember[2]
3. ...

CM002_W2_1 Is there any other household member that we haven't recorded? (除了我们刚才记录的以外)，您的家户成员（还）有哪些？

[IWER: Record each additional household member's name. 访员注意：记录下每一个新增的家户成员的姓名。]

PROCEDURE 程序:

For each household member that is not selected in CM001_W2_1, please ask CM003_W2_1. 对CM001_W2_1里面没被勾选的每一个家户成员询问CM003_W2_1.

CM003_W2_1 I know this might be difficult to talk about, but what happened to [hhmember name] that didn't choose from CM001_W2_1? 我知道这个问题可能很难回答，但是我们想知道 [没在CM001_W2_1里勾选出的名单] 为什么现在不是家户成员了？

1. Household member moved out 搬出去了
2. Household member passed away 去世了
3. Household member never lived here 从未在这里住过
4. Other 其它，请注明 ____________ (**CM003_W2_1_1**)

NOTE: DEFINITIONS FOR MOTHER, FATHER-IN-LAW AND MOTHER-IN-LAW ARE SIMILAR TO FATHER'S. FOR THE LOOPS ON FATHER, MOTHER, FATHER-IN-LAW AND MOTHER-IN- LAW, THEY ARE NAMED AS VARNAME[1], VARNAME[2], VARNAME[3] AND VARNAME[4] RESPECTIVELY.

[IWER: Section CA and Section CC and Section CG are asked for each Rs and Section CB is only asked for Family R. If Family R is not in or couldn't answer the questions, spouse can be a proxy, but other people are not allowed. 访员注意：CA/CC/CG 部分需要询问每个受访者，CB，CD，CE 和 CF 部分只需要询问家庭受访者。如果家庭受访者不在家或者无法回答，可以由其配偶来替代回答，但除配偶以外的其他人不可以回答。]

C1 PARENT, CHILDREARING AND SIBLING INFORMATION 父母、子女、兄弟姐妹信息

[In the following three parts: CA parent information, CB childrering information and CC sibling information, I'd like to ask you some questions about your family. 接下来的三部分：父母、子女、兄弟姐妹信息，我们想问您一些有关您这些家人的问题。]

CA PARENT INFORMATION 父母信息

[I'd like to ask you some questions about your parents. 首先我们想问您一些关于您父母的问题。]

CA000_W2_1 BRANCHPOINT:

IF WE KNOW THAT R's FATHER WAS NOT LIVING AT R's LAST IW, ASK CA000_W2_1, OTHERWISE GO TO CA001.

CA000_W2_1 We recorded that your father was dead at last wave, is it right? 我们的记录显示您父亲在上次访问的时候（两年前）已经去世了，对吗？

1. Yes 是 → Skip to CA002 跳至CA002
2. No 否

CA001 Is your father still living? 您父亲还健在吗？

1. Yes 是
2. No 否

[IWER: If the respondent reports more than one father, ask about the father who raised the respondent. 访员注意：如果受访者报告有多个父亲，询问抚养受访者的那个。]

**CA001_W2_1 BRANCHPOINT**:
IF WE KNOW R'S FATHER'S NAME AT LAST IW, ASK CA001_W2_1, OTHERWISE, GO TO CA002.

CA001_W2_1 Is your father's name is [Father Name]? 您父亲是叫 [父亲姓名] 吗？

1. Yes 是 → Skip to CA003 BRANCHPOINT 跳至 CA003 跳转点
2. No 否

[CAPI: Preload the household member's name list. 加载家户成员名单。]

CA002 What is his name? 您父亲叫什么名字？ ________

[IWER: Choose from the list, if there is none, choose "other" and fill in, be careful the near sound words. 访员注意：从家户成员名单中选择，如果名单中没有，选择"其它"并填入正确的姓名，注意近音字的判断

IWER: Mark "father" if respondent doesn't want to give the name. 访员注意：如果受访者不愿给出，请填写"父亲"。]

**CA003 BRANCHPOINT**:
If CA000_W2_1 = 1, THEN SKIP TO CA014 AND THEN SKIP ALL THE OTHER QUESTIONS ABOUT FATHER.<br>IF WE KNOW R'S RELATIONSHIP WITH FATHER AT R'S LAST IW, GOTO THE PROCEDURE BEFORE CA004.

CA003 Is your father your...您父亲是你的亲生父亲、继父、养父或者其他 ...?

1. Biological father 亲生父亲
2. Adoptive father 养父
3. Stepfather 继父
4. A different biological relative who raised you 抚养你长大的有血缘的亲戚
5. Another individual who raised you 其他抚养过您的人

**PROCEDURE 程序**:
If CA001 = 1, then ASK CA004 AND CA005; otherwise, skip them.

[CAPI: Preload the R's permanent address]

CA004 Where was your father born? 您父亲是在哪里出生的？

1. Your permanent address 您的常住地址
2. Another village/neighborhood in your permanent address's county/city/district 在您的常住地所在县/市/区的其他村/社区 ________ (**CA004_1**) village/neighborhood 村/社区

3. Other 其它: ______ (**CA004_2**) province_city_county/city/district 省 _ 市 _ 县/市/区, ________ (**CA004_3**) village/neighborhood 村/社区
4. Abroad 国外

[IWER: county/city includes county-level administrative units/county-level city/district. 访员注意: 县/市包括县级行政单位/县级市/区]

**CA005 BRANCHPOINT**:
IF WE KNOW WHETHER R'S FATHER HAD GROWN UP IN AN URBAN OR A RURAL AREA AT R'S LAST IW, GO TO CA006 BRANCHPOINT.

CA005 Did your father grow up in an urban area or a rural area? 您父亲是在城镇还是农村长大的?
1. City 城镇
2. Village 农村

**CA006 BRANCHPOINT**:
IF WE KNOW WHAT R'S FATHER'S ZODIAC SIGN WAS AT R'S LAST IW, GO TO CA007 BRANCHPOINT

[Show Card 出示卡片 2]

CA006 What is your father' s Chinese Zodiac sign? 您父亲的属相是 _______
[IWER: Choose from the list of Chinese Zodiac signs, see Appendix 1. 访员注意: 请选择属相, 选项见附表一]

**CA007 BRANCHPOINT**:
IF WE KNOW THE BIRTH YEAR OF R'S FATHER AT R'S LAST IW, GO TO CA008 BRANCHPOINT

CA007 In what year was your father born? 您父亲是哪一年出生的? ____1850...1950 Year 年
[IWER: Mark the year using four digits. 访员注意: 用 4 位数表示年。]

**CA008 BRANCHPOINT**:
IF WE KNOW THE DEATH YEAR OF R'S FATHER AT R'S LAST IW, GO TO CA009 BRANCHPOINT

**PROCEDURE 程序**:
If CA001 = 2, ask CA008; otherwise, skip it.

CA008 In what year did your father pass away? 您父亲哪年去世的? ___ 1900...2013 (**CA008_1**) year 年 or ___ 1...120 (**CA008_2**) Years old 岁.
[IWER: Mark the year using four digits. 访员注意: 用 4 位数表示年。]
[hard check: year > CA008. 此处的年份应该 > CA007]

CA009 BRANCHPOINT:
IF WE KNOW THE EDUCATION OF R'S FATHER AT R'S LAST IW, GO TO CA010 BRANCHPOINT

CA009 What is the highest level of education your father has completed? (not including adult education) 您父亲的最高学历是？(不包括成人教育)
1. No formal education (illiterate) 未受过教育（文盲）
2. Did not finish primary school but capable of reading and/or writing 未读完小学，但能够读、写
3. Sishu/home school 私塾
4. Elementary school 小学毕业
5. Middle school 初中毕业
6. High school 高中毕业
7. Vocational school 中专（包括中等师范、职高）毕业
8. Two-/Three-Year College/Associate degree 大专毕业
9. Four-Year College/Bachelor's degree 本科毕业
10. Master's degree 硕士毕业
11. Doctoral degree/Ph.D. 博士毕业

PROCEDURE 程序:
If CA001 = 1, ask CA010-CA022_a; otherwise, ask CA014 and then skip to procedure before CA024

CA010 What is your father's current marital status? 您父亲目前的婚姻状态？
[IWER：Common-law marriage is considered as married. 访员注意：即使没有领证但自称结婚者也可视为已婚。]
1. Married，with my mother 已婚，配偶是我母亲
2. Married，but not with my mother 已婚，但配偶不是我母亲
3. Separated 分居（不再作为配偶共同生活）
4. Divorced 离异
5. Widowed 丧偶
6. Never married 从未结婚
7. Cohabitated 同居

CA012 Does your father work currently (work includes agricultural work, earning wage work, self-employed activities, and unpaid family business work, et al.)? 请问您父亲现在工作吗 (工作包括务农、挣工资工作、从事个体、私营活动或不拿工资为家庭经营活动帮工等)
1. Yes 在工作
2. No 没工作

CA013 How is your father' s health? Very good, good, fair, poor or very poor? 请问您父亲身体情况怎么样？很好，好，一般，不好还是很不好？
1. Very good 很好

2. Good 好
3. Fair 一般
4. Poor 不好
5. Very poor 很不好

CA014 Which is/was the highest occupation of your father? 您父亲的职位最高的职业是什么?
1. Managers 国家机关、党群组织、企业、事业单位负责人
2. Professionals and technicians 专业技术人员
3. Clerks 办事人员和有关人员
4. Commercial and service workers 商业、服务业人员
5. Agricultural, forestry, husbandry and fishery producers 农、林、牧、渔、水利业生产人员
6. Production and transportation workers 生产、运输设备操作人员及有关人员
7. Can't be specified 无法分类的工作

CA015 What is your father's average income at present? (not including the money from children, but including other all income like pension and government allowance). 目前您父亲的收入有多少?(不包括子女给的钱,但包括养老金和政府津贴等所有的收入) ______ yuan/year 元/年 (**CA015_1**) or _____ yuan/month 元/月 (**CA015_2**).

[Show Card 出示卡片 4]

[CAPI: Preload the R's permanent address.]

CA016 Where does your father normally live? 您父亲一般住在哪里?
1. The same household 与我同住
2. The same or an adjacent dwelling/courtyard with me 与我同一个院子(公寓)或者相邻的院子(公寓)
3. His birthplace 他的出生地
4. Another household in your permanent address's village/neighborhood 您的常住地所在村/社区的其他房子里
5. Another village/neighborhood in your permanent address's county/city/district ______ village/neighborhood, how far away ____ km 您的常住地所在县/市/区的其他村/社区 ______ 村/社区 (**CA016_1**), 离这里有多远: ____ 公里 (**CA016_2**)
6. Other 其它: ______(**CA016_3**) province_city_county/city/district 省 _ 市 _ 县/市/区, ______(**CA016_4**) village/neighborhood 村/社区
7. Abroad 国外

[IWER: county/city includes county-level administrative units/county-level city/district. 访员注意: 县/市包括县级行政单位/县级市/区]

**PROCEDURE 程序:**
If CA016 = 5/6/7, ask CA017.

CA017 What kind of location does your father live in? 您父亲现在居住地的类型是？
1. City 城市
2. County 县城
3. Town 乡镇
4. Village 农村

CA018 Is his hukou in the same place as his current residence? 他的户口是在他现在住的地方吗？
1. Yes 是 → Skip to CA020 跳至 CA020
2. No 否
3. Does not have Hukou 没有户口 → Skip to CA021 跳至 CA021

[CAPI: Preload the R's permanent address.]

CA019 What is the location of your father's current hukou? 您父亲目前的户口所在地是？
1. His birthplace 他的出生地
2. Your birthplace 您的出生地
3. Your permanent address' s village/neighborhood 您的常住地所在的村/社区
4. Another village/neighborhood in your permanent address's county/city/district ______ village/neighborhood 您的常住地所在县/市/区的其他村/社区 ______ 村/社区 (**CA019_1**)
5. Other 其它：______(**CA019_2**) province_city_county/city/district 省 _ 市 _ 县/市/区，______(**CA019_3**) village/neighborhood 村/社区
6. Abroad 国外

[IWER: county/city includes county-level administrative units/county-level city/district. 访员注意：县/市包括县级行政单位/县级市/区]

CA020 What is your father's current hukou status? 你父亲目前的户口状态是？
1. Agriculture Hukou 农业户口
2. Non-Agriculture Hukou 非农业户口
3. Unified Residency Hukou 统一居民户口
4. Do not have Hukou 没有户口

[F1："统一居民户口"指的是某些地方实行户口制度改革后，不再区分农业与非农业户口，而是统一为"居民户口"。]

CA021 Does your father own a house? 您父亲有房产吗？
1. Yes 是
2. No 否 → Skip CA022 and CA022_a 跳过 CA022 和 CA022_a

CA022 Do you know the present value of your father's house? 您父亲的房产现在值多少钱？
____ 10000 Yuan 万元

CA022_a Does your father shares the house with others? 您父亲的房产是与其他人共有的吗？

1. with my mother 与我母亲共有
2. with other people 是与其他人共有
3. with my mother and other people 与我母亲和其他人共有
4. No 不是

PROCEDURE 程序:
Add one more section for respondent's mother, with questions identical to those asked about the respondent's father. 询问受访者有关其母亲的问题，问题与刚才问到的父亲问题相同。

PROCEDURE 程序:
if CV033 = 5, ask CA024

CA024 Do you keep in contact with your spouse's parents? 您还和您岳父母 (公公婆婆) 保持联系吗?
1. Yes 是
2. No 否

PROCEDURE 程序:
If widowed but keeping in contact with spouse's parents CA024 = 1, then ask the same information about spouse's parents. 如果受访者丧偶但是和岳父母 (公公婆婆) 保持联系 (CA024 = 1)，询问受访者岳父母/公公婆婆同样的信息.
If there are more than one spouse's parents, then ask the last spouse's parents. 如果有两对及以上的岳父母（公公婆婆），则问最后一个配偶的父母。

CC SIBLINGS 兄弟姐妹

[INTRO: Next I have some questions about your(spouse's) brothers and sisters. 接下来我们会问一些有关您（配偶）兄弟姐妹的问题。]

CC002 How many of your living siblings are: 现在活着的兄弟姐妹中，Older brothers 哥哥有几个 __0...25 (**CC002_1**), Younger brothers 弟弟有几个 __0...25 (**CC002_2**) Older sisters 姐姐有几个 __0...25 (**CC002_3**), Younger sisters 妹妹有几个 __0...25 (**CC002_4**)

CC004 How many of your deceased siblings were: 您去世的兄弟姐妹中，Older brothers 哥哥有几个 __0...25 (**CC004_1**), Younger brothers 弟弟有几个 __0...25 (**CC004_2**) Older sisters 姐姐有几个 __0...25 (**CC004_3**), Younger sisters 妹妹有几个 __0...25 (**CC004_4**)

CG Living Arrangements preferences 居住安排偏好

PROCEDURE 程序:
Main respondent and spouse both answers CG001_W2 and CG002_W2 and no proxy is allowed for these two questions. 主要受访者及其配偶都要回答CG001_W2 和CG002_W2 并且这两个问题不允许代答。

CG001_W2 Suppose an elderly person has a spouse and adult children, and has good relationship with them, what do you think is the best living arrangement for the elderly person? 假定一个老年人有配偶和成年子女，而且与子女关系融洽，您觉得怎么样的居住安排对他最好?

1. Live with adult children 与成年子女一起住
2. Don't live with them in the same house, but live in the same community or village. 不与子女一起住，但是跟子女住在同一个村/社区
3. Don't live with them in the same house and the same community or village. 不与子女一起住，也不跟子女住在同一个村/社区
4. Live in a nursing house 住养老院
5. Other 其他

CG002_W2 Suppose an elderly person has no spouse but has adult children, and has good relationship with them. What do you think is the best living arrangement for him/her? 假定一个老年人没有配偶，但是有成年子女，而且与子女关系融洽，您觉得怎么样的居住安排对他最好?

1. Live with adult children 与成年子女一起住
2. Don't live with them in the same house, but live in the same community or village. 不与子女一起住，但是跟子女住在同一个村/社区
3. Don't live with them in the same house and the same community or village. 不与子女一起住，也不跟子女住在同一个村/社区
4. Live in a nursing house 住养老院
5. Other 其他

CC005_W2_1 If the main R's spouse is in, please ask the spouse about spouse's parents and siblings questions; if the spouse is not in, please ask main R for proxy. For the following questions about spouse' s parents and siblings (Section CA/Section CC/Section CG), who answered the questions? Please record. 如果受访者配偶在的话，就请配偶回答他/她自己的父母/兄弟姐妹住安排的问题，如果配偶不在，则由家庭受访者回答。接下来的配偶的父母/弟姐妹住安排部分的问题是由谁来回答的？请记录。

1. Response answered him/herself 受访者自己回答
2. Spouse 配偶回答

CG003_W2 How often did the respondent receive assistance in answering section CA/CC/CG? 受访者填写 CA/CC/CG 部分问卷时是否求助？

[IWER: If it is answered by a proxy, please record the respondent' s reaction. 访员注意：如果是协助回答，请记录受访者的反应。]

1. Never 从未
2. A few times 偶尔几次
3. Most or all of the time 大多

CB CHILDREARING INFORMATION 子女信息

[INTRO: Next we will ask some questions about your fostering of your children/about raising your children. 下面，我将询问一些关于您抚养您子女的问题。]

PROCEDURE 程序:
If the respondent is currently unmarried BE001 = 3 – 6, ask CB001-CB016. 如果受访者现在婚姻状态不是已婚 BE001 = 3 – 6，询问CB001-CB016

CB003 How many biological children do you have who have passed away? 您亲生子女中有去世的吗？如果有，多少个已经去世？ __ 0...25 Persons 人
[IWER: Mark “0” if none. 访员注意：如果没有请填 0。]

PROCEDURE 程序:
If CB003= 0, then skip to CB001. 如果CB003= 0，跳至CB001.

[CAPI: Preload all the living children’s name in the last wave. 程序：加载上一期所有健在子女名单。]

CB003_W2_1 What are the names of your deceased biological children? 您去世的亲生孩子叫什么名字？

[IWER: Choose all from the list; if it is not on the list, please choose “other”, and then fill in the names of those children. Pay attention to the near sound words. 访员注意：在上一期所有健在子女名单中选出受访者说出的所有去世子女姓名；如果名单中没有，请选择“其他”，并写出姓名，注意近音字的判断。]

PROCEDURE 程序:
Repeat questions CB004-CB008 for each child marked in CB003_W2_1 ; If the name is chosed from the list, then skip to CB007. 程序：请针对每个新增的去世的孩子姓名询问CB004-CB008；如果是上一期健在的子女，则跳至CB007.

CB004 When was CHILDn’s NAME born? [孩子姓名的出生日期是？
____ 1900...2013 (**CB004_1**) year 年 __0...12 (**CB004_2**) month 月

PROCEDURE 程序:
If the person does not know the date of birth, CB004 = DK or CB004 = RF, skip CB005. 如果受访者不知道出生日期即CB004选择不知道或拒绝回答，跳过CB005.

CB005 Is your answer to CB020 based on the solar or the lunar calendar? 您刚才回答的是公历（阳历）还是农历（阴历）？
1. Solar calendar 公历（阳历）
2. Lunar calendar 农历（阴历）

CB006 Sex of CHILDn’s NAME [孩子姓名] 的性别是？

1. Male 男
2. Female 女

CB007 When did CHILDn's NAME pass away[孩子姓名] 去世的日期是？____ 1900...2013 (**CB007_1**) year 年 __0...12 (**CB007_2**) month 月

PROCEDURE 程序:
If the person does not know the date of birth, CB007 = DK or CB007 = RF, skip CB008.
如果受访者不知道去世日期即CB007选择不知道或拒绝回答，跳过CB008.

CB008 Is your answer to CB007 based on the solar or the lunar calendar? 您刚才回答的是公历（阳历）还是农历（阴历）?
1. Solar calendar 公历（阳历）
2. Lunar calendar 农历（阴历）

CB001 Have you ever given birth to any (other) child? If yes, how many are currently living? 您（还）有过亲生孩子吗？如果有过，总共多少个健在？__ 0...25 Persons 人
[IWER: Mark "0" if none. 访员注意：如果没有请填 0。]

PROCEDURE 程序:
If CB001 $\neq 0$, ask CB002; If CB001 $= 0$, go to CB011. 如果CB001 $\neq 0$，询问CB002题；如果CB001 $= 0$，跳至CB011.

CB001_W2_1 We recorded that [read all the preloaded children's name] last wave, which [children's name] is your biological children? 我们系统中显示上次访问时有这些子女 [读出所有子女姓名]，我们想确认一下，哪些是您的亲生子女？
[IWER: Choose names of those children. Choose all that apply. 访员注意：选出此类亲生子女姓名，可多选。]

[CAPI: Preload all the household members' name. 加载所有家户成员的名字。]

CB002 What are the name of your biological children？亲生子女的姓名是？
[IWER: Choose from the household member list, choose all that apply; if there is none, then fill in names of those children. 访员注意：在家户成员列表中选出姓名，可多选；如果名单中没有，则手动填出此类亲生子女姓名。]

CB011 How many adopted or foster children or step children do you have who have passed away? 您收养或抚养的子女或继子女中有去世的吗？如果有，多少个已经去世了？__ 0...25 Persons 人
[IWER: Mark "0" if none. 访员注意：如果没有请填 0。]

PROCEDURE 程序:
If CB011 $= 0$, then skip to CB009. 如果CB011 $= 0$，跳至CB009.

[CAPI: Preload all the living children's name in the last wave. 加载上一期所有健在的子女名单。]

CB011_W2_1 What are the names of your deceased adopted foster or step children? 您去世的收养或抚养的子女或继子女叫什么名字?

[IWER: Choose all from the list; if it is not on the list, please choose “other”, and then fill in the names of those children. Pay attention to the near sound words. 访员注意：在上一期所有健在子女名单中选出受访者说出的所有去世子女姓名；如果名单中没有，请选择“其他”，并写出姓名，注意近音字的判断。]

PROCEDURE 程序:

Repeat questions CB012-CB016 for each child marked in CB011_W2_1 who has passed away; If the name is chosed from the list, then skip to CB015. 请针对每个新增的去世的孩子姓名询问CB012-CB016；如果是上一期健在的子女，则跳至CB015.

CB012 When was CHILDn's NAME born [孩子姓名] 的出生日期是?
____ 1900...2013 (**CB012_1**) year 年 __0...12 (**CB012_2**) month 月

PROCEDURE 程序:

If the person does not know the date of birth, CB012 = DK or CB012 = RF, skip CB013. 如果受访者不知道出生日期即CB012选择不知道或拒绝回答，跳过CB013.

CB013 Is your answer to CB012 based on the solar or the lunar calendar? 您刚才回答的是公历（阳历）还是农历（阴历）?

1. Solar calendar 公历（阳历）
2. Lunar calendar 农历（阴历）

CB014 Sex of CHILDn's NAME [孩子姓名] 的性别是?

1. Male 男
2. Female 女

CB015 When did CHILDn's NAME pass away [孩子姓名] 去世的日期是? ____ 1900...2013 (**CB015_1**) year 年 __0...12 (**CB015_2**) month 月

PROCEDURE 程序:

If the person does not know the date of birth, CB015 = DK or CB015 = RF, skip CB016. 如果受访者不知道去世日期即CB015选择不知道或拒绝回答，跳过CB016.

CB016 Is your answer to CB015 based on the solar or the lunar calendar? 您刚才回答的是公历（阳历）还是农历（阴历）?

1. Solar calendar 公历（阳历）
2. Lunar calendar 农历（阴历）

CB009 Have you ever adopted or fostered any (other) child or (other) step child? If yes, how many are currently living? 您（还）收养或者抚养过（其他）子女或继子女吗? 如果有，总共有多少个健在? __ 0...25 Persons 人
[IWER: Mark “0” if none. 访员注意：如果没有请填 0。]

PROCEDURE 程序:

If CB009 $\neq 0$, ask CB010; If CB009 $= 0$, go to the procedure before CB019. 如果CB009 $\neq 0$，询问CB010题；如果CB009 $= 0$，跳至CB019前的程序.

CB009_W2_1 We recorded that [read all the preloaded children's name] last wave, which [children's name] is your adopted or foster or step children? 我们系统中显示上次访问时有 [读出所有子女姓名]，我们想确认一下，哪些是您收养或抚养的子女或继子女？
[IWER: Choose names of those children, choose all that apply. 访员注意：选出此类子女姓名，可多选。]

[CAPI Preload all the household members' name. 加载所有家户成员的名字。]

CB010 What's the name of your adopted or foster children? 收养的子女或继子女的姓名是？
[IWER: Choose from the household member list, choose all that apply; if there is none, then fill in names of those children. 访员注意：在家户成员列表中选出姓名，可多选；如果名单中没有，则手动填出此类亲生子女姓名。]

PROCEDURE 程序:

If the respondent is currently married BE001 $= 1/2/7$, ask CB017 -CB048. 如果受访者现在婚姻状态是已婚 BE001 $= 1/2/7$, 询问CB017 -CB048

CB019 How many biological children do you and your (current) spouse have together who have passed away? 您跟您（现在的）配偶的亲生子女中，有去世的吗？如果有，多少个已经去世？ __ 0...25 Persons 人
[IWER: Mark "0" if none. 访员注意：如果没有请填 0。]

PROCEDURE 程序:

If CB019 $= 0$, then skip to CB017. 如果CB019 $= 0$，跳至CB017.

[CAPI: Preload all the living children's name in the last wave. 加载上一期所有健在的子女名单。]

CB019_W2_1 What are the names of you and your (current) spouse's deceased biological children? 您和您配偶去世了的亲生孩子叫什么名字？
[IWER: Choose all from the list; if it is not on the list, please choose "other", and then fill in the names of those children. Pay attention to the near sound words. 访员注意：在上一期所有健在子女名单中选出受访者说出的所有去世子女姓名；如果名单中没有，请选择"其他"并写出姓名，注意近音字的判断。]

PROCEDURE 程序:

Repeat questions CB020-CB024 for each child marked in CB019_W2_1 who has passed away; If the name is chosed from the list, then skip to CB023. 请针对每个新增的去世的孩子姓名询问CB020-CB024；如果是上一期健在的子女，则跳至CB023.

CB020 When was CHILDn's NAME born [孩子姓名] 的出生日期是?
____ 1900...2013 (**CB020_1**) year 年 __0...12 (**CB020_2**) month 月

**PROCEDURE 程序**:<br>If the person does not know the date of birth, CB020 = DK or CB020 = RF, skip CB021.<br>如果受访者不知道出生日期即CB020选择不知道或拒绝回答，跳过CB021.

CB021 Is your answer to CB020 based on the solar or the lunar calendar? 您刚才回答的是公历（阳历）还是农历（阴历）?
1. Solar calendar 公历（阳历）
2. Lunar calendar 农历（阴历）

CB022 Sex of CHILDn's NAME [孩子姓名] 的性别是?
1. Male 男
2. Female 女

CB023 When did CHILDn's NAME pass away [孩子姓名] 去世的日期是? ____ 1900...2013 (**CB023_1**) year 年 __0...12 (**CB023_2**) month 月

**PROCEDURE 程序**:<br>If the person does not know the date of birth, CB023 = DK or CB023 = RF, skip CB024.<br>如果受访者不知道去世日期即CB023选择不知道或拒绝回答，跳过CB024.

CB024 Is your answer to CB023 based on the solar or the lunar calendar? 您刚才回答的是公历（阳历）还是农历（阴历）?
1. Solar calendar 公历（阳历）
2. Lunar calendar 农历（阴历）

CB017 Do you and your (current) spouse have any (other) biological children? If yes, how many are currently living? 您跟您（现在的）配偶（还）生过孩子吗? 如果有，总共有多少个健在? __ 0...25 Persons 人

**PROCEDURE 程序**:<br>If CB017 $\neq 0$, ask CB018; if CB017 $= 0$, go to CB027. 程序：如果CB017 $\neq 0$，询问CB018；如果CB017 $= 0$，跳至CB027.

CB017_W2_1 We recorded that [read all the preloaded children's name] last wave, which [children's name] is you and your (current) spouse have together? 我们系统中显示上次访问时有 [读出所有子女姓名]，我们想确认一下，哪些是您和您现在的配偶共同亲生的?
[IWER: Choose names of those children, choose all that apply. 访员注意：选出此类子女姓名，可多选。]

[CAPI: Preload all the household members' name. 加载所有家户成员的名字。]

CB018 What are the names of those children? 这些子女的姓名是?
[IWER: Choose from the household member list, choose all that apply; if there is none, then fill in names of those children 访员注意：在家户成员列表中选出姓名，可多选；如果名单中没有，则手动填出此类亲生子女姓名。]

CB027 How many additional biological children do you have who have passed away? 除了跟您（现在的）配偶一起生的子女之外，您自己的亲生子女中，有去世的吗？如果有，多少个去世了？ __ 0...25 Persons 人
[IWER: Mark "0" if none. 访员注意：如果没有请填 0。]

PROCEDURE 程序:
If CB027 = 0, then skip to CB025. 如果CB027 = 0，跳至CB025.

[CAPI: Preload all the living children's name in the last wave. 加载上一期所有健在的子女名单。]

CB027_W2_1 What are the names of your deseased biological children? 您去世的亲生孩子叫什么名字?
[IWER: Choose all from the list; if it is not on the list, please choose "other", and then fill in the names of those children. Pay attention to the near sound words. 访员注意：在上一期所有健在子女名单中选出受访者说出的所有去世子女姓名，可多选；如果名单中没有，请选择"其他"，并写出姓名，注意近音字的判断。

PROCEDURE 程序:
Repeat questions CB028-CB032 for each child marked in CB027_W2_1 who has passed away; If the name is chosed from the list, then skip to CB031. 请针对每个新增的去世的孩子姓名询问CB028-CB032；如果是上一期健在的子女，则跳至CB031.

CB028 When was CHILDn's NAME born [孩子姓名] 的出生日期是?
____ 1900...2013 (**CB028_1**) year 年 __0...12 (**CB028_2**) month 月

PROCEDURE 程序:
If the person does not know the date of birth, CB028 = DK or CB028 = RF, skip CB029. 如果受访者不知道出生日期即CB028选择不知道或拒绝回答，跳过CB029.

CB029 Is your answer to CB028 based on the solar or the lunar calendar? 您刚才回答的是公历（阳历）还是农历（阴历）?

1. Solar calendar 公历（阳历）
2. Lunar calendar 农历（阴历）

CB030 Sex of CHILDn's NAME [孩子姓名] 的性别是?

1. Male 男
2. Female 女

CB031 When did CHILDn's NAME pass away [孩子姓名] 去世的日期是? ____ 1900...2013 (**CB031_1**) year 年 __0...12 (**CB031_2**) month 月

PROCEDURE 程序:
If the person does not know the date of birth, CB031 = DK or CB031 = RF, skip CB032. 如果受访者不知道去世日期即CB031选择不知道或拒绝回答, 跳过CB032.

CB032 Is your answer to CB031 based on the solar or the lunar calendar? 您刚才回答的是公历（阳历）还是农历（阴历）?
1. Solar calendar 公历（阳历）
2. Lunar calendar 农历（阴历）

CB025 How many (other) additional biological children do you have who are currently living? 您自己还有其他亲生子女吗，如果有，总共有多少个健在? __ 0...25 Persons 人

PROCEDURE 程序:
If CB025 $\neq 0$, ask CB026; if CB025 $= 0$, go to CB035. 程序: 如果CB025 $\neq 0$, 询问CB026题; 如果CB025 $= 0$, 跳至CB035.

CB025_W2_1 We recorded that [read all the preloaded children's name] last wave, which [children's name] is your additional biological children? 我们系统中显示上次访问时有 [读出所有子女姓名]，我们想再确认一下，除了与您现在的配偶一起生的子女以外，哪些是您自己亲生的?
[IWER: Choose names of those children, choose all that apply. 访员注意: 选出此类子女姓名, 可多选。]

[CAPI: Preload all the household members' name. 加载所有家户成员的名字。]

CB026 What are the names of those children? 这些子女的姓名是?
[IWER: Choose from the household member list, choose all apply; if there is none, then fill in names of those children 访员注意: 在家户成员列表中选出姓名, 可多选; 如果名单中没有, 则手动填出此类子女姓名。]

CB035 How many additional biological children does your (current) spouse have who have passed away? 除了您和您（现在的）配偶一起生的子女之外，您配偶的亲生子女中，有去世的吗? 如果有，有多少个去世了? __ 0...25 Persons 人
[IWER: Mark "0" if none. 访员注意: 如果没有请填 0。]

PROCEDURE 程序:
If CB035 $= 0$, then skip to CB033. 程序: 如果CB035 $= 0$, 跳至CB033.

[CAPI: Preload all the living children's name in the last wave. 加载上一期所有健在的子女名单。]

CB035_W2_1 What are the names of your (current) spouse's deceased biological children? 您配偶去世的亲生孩子叫什么名字?

[IWER: Choose all from the list; if it is not on the list, please choose "other", and then fill in the names of those children. Pay attention to the near sound words. 访员注意:在上一期所有健在子女名单中选出受访者说出的所有去世子女姓名,可多选;如果名单中没有,请选择"其他",并写出姓名,注意近音字的判断。]

PROCEDURE 程序:
Repeat questions CB036-CB040 for each child marked in CB035_W2_1 who has passed away; If the name is chosed from the list, then skip to CB039. 请针对每个新增的去世的孩子姓名询问CB036-CB040;如果是上一期健在的子女,则跳至CB039.

CB036 When was CHILDn's NAME born [孩子姓名] 的出生日期是?
____ 1900...2013 (**CB036_1**) year 年 __0...12 (**CB036_2**) month 月

PROCEDURE 程序:
If the person does not know the date of birth, CB036 = DK or CB036 = RF, skip CB037. 如果受访者不知道出生日期即CB036选择不知道或拒绝回答,跳过CB037.

CB037 Is your answer to CB036 based on the solar or the lunar calendar? 您刚才回答的是公历(阳历)还是农历(阴历)?

1. Solar calendar 公历(阳历)
2. Lunar calendar 农历(阴历)

CB038 Sex of CHILDn's NAME [孩子姓名] 的性别是?

1. Male 男
2. Female 女

CB039 When did CHILDn's NAME pass away [孩子姓名] 去世的日期是? ____ 1900...2013 (**CB039_1**) year 年 __0...12 (**CB039_2**) month 月

PROCEDURE 程序:
If the person does not know the date of birth, CB039 = DK or CB039 = RF, skip CB040. 如果受访者不知道去世日期即CB039选择不知道或拒绝回答,跳过CB040.

CB040 Is your answer to CB039 based on the solar or the lunar calendar? 您刚才回答的是公历(阳历)还是农历(阴历)?

1. Solar calendar 公历(阳历)
2. Lunar calendar 农历(阴历)

CB033 How many (other) additional biological children does your (current) spouse have who are currently living? 您(现在的)配偶(还)有自己的亲生子女吗,如果有,总共有多少个健在? __ 0...25 Persons 人

PROCEDURE 程序:
If CB033 $\neq 0$, ask CB034; if CB033 $= 0$, go to CB043. 程序：如果CB033 $\neq 0$，询问CB034；如果CB033 $= 0$，跳至CB043.

CB033_W2_1 We recorded that [read all the preloaded children's name] last wave, which [children's name] is your spouse's additional biological children? 我们系统中显示上次访问时有【读出所有子女姓名】，我们想确认一下，除了与您一起生的子女以外，哪些是您配偶亲生的？
[IWER: Choose names of those children, choose all apply. 访员注意：选出此类子女姓名，可多选。]

[CAPI: Preload all the household members' name. 加载所有家户成员的名字。]

CB034 What are the names of those children? 这些子女的姓名是？
[IWER: Choose from the household member list, choose all that apply; if there is none, then fill in names of those children 访员注意：在家户成员列表中选出姓名，可多选；如果名单中没有，则手动填出此类亲生子女姓名。]

CB043 How many adopted or foster children do you or your (current) spouse have who have passed away? 您或您（现在的）配偶收养或抚养的子女中有去世的吗？如果有，多少个已经去世了？ __ 0...25 Persons 人
[IWER: Mark "0" if none. 访员注意：如果没有请填 0。]

PROCEDURE 程序:
If CB043 $= 0$, then skip to CB041. 如果CB043 $= 0$，跳至CB041.

[CAPI: Preload all the living children's name in the last wave. 加载上一期所有健在的子女名单。]

CB043_W2_1 What are the names of your or your (current) spouse's deceased adopted, foster or step children? 您或您配偶去世的收养或抚养的孩子或继子女叫什么名字？
[IWER: Choose all from the list; if it is not on the list , please choose "other", then fill in the names of those children. Pay attention to the near sound words. 访员注意：在上一期所有健在子女名单中选出受访者说出的所有去世子女姓名，可多选；如果名单中没有，请选择"其他"，并写出姓名，注意近音字的判断。]

PROCEDURE 程序:
Repeat questions CB044-CB048 for each child marked in CB043_W2_1 who has passed away; If the name is chosed from the list, then skip to CB047. 请针对每个填进去的去世的孩子姓名询问CB044-CB048；如果是在名单中选择的姓名，则跳至CB047.

CB044 When was CHILDn's NAME born [孩子姓名] 的出生日期是？
____ 1900...2013 (**CB044_1**) year 年 __0...12 (**CB044_2**) month 月

PROCEDURE 程序:
If the person does not know the date of birth, CB044 = DK or CB044 = RF, skip CB045.
如果受访者不知道出生日期即CB044选择不知道或拒绝回答，跳过CB045.

CB045 Is your answer to CB044 based on the solar or the lunar calendar? 您刚才回答的是公历（阳历）还是农历（阴历）?
1. Solar calendar 公历（阳历）
2. Lunar calendar 农历（阴历）

CB046 Sex of CHILDn's NAME [孩子姓名] 的性别是?
1. Male 男
2. Female 女

CB047 When did CHILDn's NAME pass away[孩子姓名] 去世的日期是? ____ 1900...2013 (**CB047_1**) year 年 __0...12 (**CB047_2**) month 月

PROCEDURE 程序:
If the person does not know the date of birth, CB047 = DK or CB047 = RF, skip CB048.
如果受访者不知道去世日期即CB047选择不知道或拒绝回答，跳过CB048.

CB048 Is your answer to CB047 based on the solar or the lunar calendar? 您刚才回答的是公历（阳历）还是农历（阴历）?
1. Solar calendar 公历（阳历）
2. Lunar calendar 农历（阴历）

CB041 How many (other) adopted or foster children or step children do you or your (current) spouse have who are currently living? 您和您（现在的）配偶（还）有其他收养领养的子女或继子女吗? 如果有，总共有多少个健在? __ 0...25 Persons 人

PROCEDURE 程序:
If CB041 $\neq 0$, ask CB042; if CB041 $= 0$, go to procedure before CB049. 如果CB041 $\neq 0$, 询问CB042, 如果CB041 $= 0$, 跳至CB049之前的程序.

CB041_W2_1 We recorded that [read all the preloaded children's name] last wave, which [children's name] is you or your spouse's adopted or foster children? 我们系统中显示上次访问时有 [读出所有子女姓名]，我们想确认一下，您或您配偶收养或抚养的子女有?
[IWER: Choose names of those children, choose all that apply. 访员注意：选出此类子女姓名。]

[CAPI: Preload all the household members' name. 加载所有家户成员的名字。]

CB042 What are the names of those children? 这些子女的姓名是?
[IWER: Choose from the household member list, choose all that apply; if there is none, then fill in names of those children 访员注意：在家户成员列表中选出姓名，可多选；如果名单中没有，则手动填出此类亲生子女姓名。]

PROCEDURE 程序:
Repeat questions CB049 - CB079 for each living child. 请针对每个健在的孩子询问CB049 - CB079.

CB049 Is [CHILD's NAME] a boy or a girl? [孩子姓名] 是男孩还是女孩?
1. Boy (Son) 男孩（儿子）
2. Girl (Daughter) 女孩（女儿）

[Show Card 出示卡片 2]

CB050 What is the Chinese Zodiac sign for CHILDn's NAME? [孩子姓名] 的属相是 _______
[IWER: Choose from the list of Chinese Zodiac signs, see Appendix 1 访员注意: 请选择属相, 选项见附表一]

CB051 Birth month and year for CHILDn's NAME? [孩子姓名] 的出生年月?
____ 1900...2013 (**CB051_1**) year 年 __0...12 (**CB051_2**) month 月
[IWER: The year must be a number in the range [1900 – 2013]. Mark the year using four digits. Take down the month as its actual number. For example, write January as "1" not "01", December as "12". If do not remember month and day, fill '0'. 访员注意: 年份填写范围 [1900 — 2013]。用 4 位数表示年, 按照实际的月份填写月。例: 1 月写作 "1", 而不是 "01" ,12 月写作 "12"。如果记不住月份和日期, 请填入 '0'。]

CB052 Is your answer to CB051 based on the solar or the lunar calendar? 您刚才回答的是公历（阳历）还是农历（阴历）?
1. Solar calendar 公历（阳历）
2. Lunar calendar 农历（阴历）

[Show Card 出示卡片 4]

[CAPI: Preload the R's permanent address.]

CB053 Where does this CHILDn's NAME normally live now? 目前, [孩子姓名] 在哪里常住?
1. This household, and economicly dependent. 这个家里且经济上不独立
2. This household, but economicly independent. 这个家里, 但是经济上独立
3. The same or adjacent dwelling/courtyard with you 与您同一个院子（公寓）或者相邻的院子（公寓）
4. Another household in your permanent address's village/neighborhood 您的常住地所在村/社区的其他房子里
5. Another village/neighborhood in your permanent address's county/city/district; distance from here: ____km 您的常住地所在县/市/区的其他村/社区 ______ 村/社区 (**CB053_1**), 离这里有多远: ____(**CB053_2**) 公里
6. Other 其它: ______(**CB053_3**) province_city_county/city/district 省 _ 市 _ 县/市/区, ______(**CB053_4**) village/neighborhood 村/社区

7. Abroad 国外

CB051 BRANCHPOINT:
If CB053 = 5 – 7, ASK CB054; OTHERWISE, GO TO CB055

CB054 In what type of location does CHILDn's NAME live? [孩子姓名] 现居住地的类型是？
1. City 城市
2. County 县城
3. Town 乡镇
4. Village 农村

CB055 What is the current hukou status of CHILDn's NAME? [孩子姓名] 目前的户口状态是？
1. Agriculture Hukou 农业 → Skip to CB056 跳至CB056
2. Non-Agriculture Hukou 非农业 → Skip to CB056 跳至CB056
3. Unified Residency Hukou 统一居民户口
4. Does not have Hukou 没有户口 → Skip to CB058 跳至CB058

F1："统一居民户口"指的是某些地方实行户口制度改革后，不再区分农业与非农业户口，而是统一为"居民户口"。

CB055_W2_1 What is [child name]'s Hukou status before he/she has the unified residency hukou? [孩子姓名] 在获得统一居民户之前是什么户口？
1. Agriculture Hukou 农业
2. Non-Agriculture Hukou 非农业
3. Do not have Hukou 没有户口

F1："统一居民户口"指的是某些地方实行户口制度改革后，不再区分农业与非农业户口，而是统一为"居民户口"。

CB055_W2_2 When did [child name] have the unified residence Hukou? [孩子姓名] 什么时候获得统一居民户口？ ____2000...2013 year 年

F1："统一居民户口"指的是某些地方实行户口制度改革后，不再区分农业与非农业户口，而是统一为"居民户口"。

CB056 Is CHILDn's NAME's hukou location the same as his/her place of residence? [孩子姓名] 的户口所在地与居住地相同吗?
1. Yes 是 → Skip to CB081 BRANCHPOINT 跳至 CB081 BRANCHPOINT
2. No 否

[CAPI: Preload the R's permanent address.]

CB057 Where is the current hukou location of CHILDn's NAME? [孩子姓名] 目前的户口所在地是？
1. This household 这个家里
2. His|her birthplace 他/她的出生地

3. Your permanent address’s village/neighborhood 您的常住地所在村/社区
4. Another village/neighborhood in your permanent address’s county/city/district 您的常住地所在县/市/区的其他村/社区 ____ village/neighborhood 村/社区 (**CB057_1**)
5. Other 其它: ______(**CB057_2**) province_city_county/city/district 省 _ 市 _ 县/市/区, ______(**CB057_3**) village/neighborhood 村/社区
6. Other(specify) 其他 (请注明) ______(**CB057_4**)

[IWER: county/city includes county-level administrative units/county-level city/ district. 访员注意: 县市包括县级行政单位/县级市/区.]

CB081 Where was the birth place of CHILD’s NAME? [孩子姓名] 出生地是?
1. Your permanent address ‘s village/neighborhood 您的常住地所在的村/社区
2. Another village/neighborhood in your permanent address ‘s county/city/district 您的常住地所在县/市/区的其他村/社区 ____ village/neighborhood 村/社区 (**CB081_1**)
3. Other 其它: ______(**CB081_2**) province_city_county/city/district 省 _ 市 _ 县/市/区, ______(**CB081_3**) village/neighborhood 村/社区
4. Abroad 国外

CB082 Is [CHILDn’s NAME]’s present hukou status and location the same as his/her first Hukou? [孩子姓名] 目前户口与他/她出生时获得户口是否一样?
1. Yes 是 → Skip to CB058 BRANCHPOINT 跳至CB058 BRANCHPOINT
2. No 否

CB083 How did [CHILDn’s NAME]’s Hukou status or location change? [孩子姓名] 的户口发生过什么变化?
1. Both Hukou status and location have changed 户口类型和户口所在地都变化过
2. Only Hukou status has changed 只有户口类型变化
3. Only Hukou location has changed 只有户口所在地变化 → Skip to CB085 BRANCHPOINT 跳至CB085 BRANCHPOINT

CB084 What was [CHILDn’s NAME]’s first HuKou status? [孩子姓名] 第一个户口的类型是?
1. Agricultural Hukou 农业
2. Non-agricultual Hukou 非农业

CB085 BRANCHPOINT:

IF CB083 = 2, GO TO CB058 BRANCHPOINT

IF WE KNOW THE FIRST HUKOU LOCATION OF [CHILDn’s NAME] AT THE LAST IW, GO TO CB058 BRANCHPOINT

CB085 What was the location of [CHILDn’s NAME]’s first hukou? [孩子姓名] 第一个户口所在地?
1. Your permanent address’s village/neighborhood 您的常住地所在村/社区

2. Another village/neighborhood in your permanent address's county/city/district 您的常住地所在县/市/区的其他村/社区 ______ (**CB085_1**)
3. Other 其它: ______ (**CB085_2**) province_city_county/city/district 省 _ 市 _ 县/市/区, ______ (**CB085_3**) village/neighborhood 村/社区
4. Abroad 国外

CB058 BRANCHPOINT:
IF [CHILDn's AGE] IS LESS THAN 6, GO TO NEXT CHILD

CB058 Is CHILDn's NAME still in school now? 请问 [孩子姓名] 现在还在上学吗?
1. Yes 是
2. No 否 → Skip to CB060 BRANCHPOINT 跳至CB060 BRANCHPOINT

CB059 What level of schooling and grade is CHILDn's NAME currently enrolled in? [孩子姓名] 现在读几年级?
1. Primary school grade 1 小学 1 年级
2. Primary school grade 2 小学 2 年级
3. Primary school grade 3 小学 3 年级
4. Primary school grade 4 小学 4 年级
5. Primary school grade 5 小学 5 年级
6. Primary school grade 6 小学 6 年级
7. Middle school grade 1 初中 1 年级
8. Middle school grade 2 初中 2 年级
9. Middle school grade 3 初中 3 年级
10. Middle school grade 4 初中 4 年级
11. High school, grade 1 高中 1 年级
12. High school, grade 2 高中 2 年级
13. High school, grade 3 高中 3 年级
14. Vocational/technical high school year 1 中专 1 年级
15. Vocational/technical high school year 2 中专 2 年级
16. Vocational/technical high school year 3 中专 3 年级
17. College year 1 大专/大学 1 年级
18. College year 2 大专/大学 2 年级
19. College year 3 大专/大学 3 年级
20. College year 4 大专/大学 4 年级
21. College year 5 大专/大学 5 年级
22. College year 6 大专/大学 6 年及 6 年级以上
23. Master' s degree 硕士
24. Doctoral degree/Ph.D. degree 博士

PROCEDURE 程序:
Skip to CB063 BRANCHPOINT 请跳至CB063 BRANCHPOINT

CB060 BRANCHPOINT:
IF [CHILDn's AGE] IS LESS THAN 12, GO TO NEXT CHILD

CB060 What is the highest level of education CHILDn's NAME completed? (not including adult education) [孩子姓名] 的最高学历是？(不包括成人教育)

1. No formal education (illiterate) 未受过正规教育（文盲）→ Skip to CB063 BRANCHPOINT 跳至CB063 BRANCHPOINT
2. Did not finish primary school but capable of reading or writing 未读完小学，但能够读、写 → Skip to CB061 BRANCHPOINT 跳至 CB061 BRANCHPOINT
3. Sishu/home school 私塾
4. Elementary school 小学毕业 → Skip to CB062 BRANCHPOINT 跳至 CB062 BRANCHPOINT
5. Middle school 初中毕业 → Skip to CB062 BRANCHPOINT 跳至 CB062 BRANCHPOINT
6. High school 高中毕业 → Skip to CB062 BRANCHPOINT 跳至 CB062 BRANCHPOINT
7. Vocational school 中专毕业（包括中等师范、职高）→ Skip to CB062 BRANCHPOINT 跳至 CB062 BRANCHPOINT
8. Two-/Three-Year College / Associate degree 大专毕业 → Skip to CB062 BRANCHPOINT 跳至 CB062 BRANCHPOINT
9. Four-Year College / Bachelor’s degree 本科毕业 → Skip to CB062 BRANCHPOINT 跳至 CB062 BRANCHPOINT
10. Post-graduate, Master’s degree 硕士毕业 → Skip to CB062 BRANCHPOINT 跳至 CB062 BRANCHPOINT
11. Post-graduate, doctoral degree/Ph.D. 博士毕业 → Skip to CB063 BRANCHPOINT 跳至CB063 BRANCHPOINT

CB060_W2_1 How many years did CHILDN's NAME spend in sishu/home school? [孩子姓名] 上过几年私塾？ __ 年 → Skip to CB063 BRANCHPOINT 跳至CB063 BRANCHPOINT

CB061 How many years did CHILDn's NAME spend in primary school? [孩子姓名] 上过几年小学？ __ 年 → Skip to CB063 BRANCHPOINT 跳至CB063 BRANCHPOINT

CB062 How many additional years of schooling did CHILDn's NAME receive after [THE ANSWER CHOSEN IN CB060]? [孩子姓名] 在 [CB060的答案] 之后又上过几年学？ __ 年

CB063 BRANCHPOINT:
IF [CHILDn's AGE] IS LESS THAN 16, GO TO NEXT CHILD

CB063 What is CHILDn's NAME marital status? [孩子姓名] 的婚姻状况是？

1. Married with spouse present 已婚并与配偶一同居住
2. Married but not living with spouse temporarily for reasons such as work 已婚，但因为工作等原因暂时没有跟配偶在一起居住
3. Separated 分居（不再作为配偶共同生活）
4. Divorced 离异
5. Widowed 丧偶

6. Never married 从未结婚
7. Cohabitated 同居

PROCEDURE 程序:
Ask CB064 if child has any college education, i.e. CB059 = 17 – 24 or CB060 = 8 – 11
如果孩子接受过大专或以上教育 (即CB059 = 17 – 24 或者CB060 = 8 – 11) 请提问CB064

CB064 How much did you and your spouse spend to support this [CHILDn's NAME]'s college education? 您和您的配偶花了多少钱供 [孩子的姓名] 上大学（大专或以上）? _____ Yuan 元

PROCEDURE 程序:
If the children has never married(CB063 = 6), please skip CB065 -CB068. 如果这个孩子从未结婚 (CB063 = 6)，那么跳过CB065 -CB068

CB065 How many children does [CHILDn's NAME] have? [孩子姓名] 有多少个子女? __ 0...25 Persons 人

PROCEDURE 程序:
If CB065 = 0, skip to CB067. 如果回答CB065 = 0，跳至CB067.

CB066 How many children under age 16 does CHILDn's NAME have? [孩子姓名] 有多少个 16 岁以下的子女? __ 0...25 Persons 人
[Softcheck: if the number of sons is smaller than the number of adult sons. 如果CB065 < CB066, 则进行检查]

CB067 How many grandchildren does CHILDn's NAME have? [孩子姓名] 有多少个孙子女? __ 0...25 Persons 人

PROCEDURE 程序:
If CB067 = 0, skip to CB069. 如果CB067 = 0，跳至CB069.

CB068 How many grandchildren under age 16 does CHILDn's NAME have? [孩子姓名] 有多少个 16 岁以下的孙子女? __ 0...25 Persons 人
[Softcheck: if the number of sons is smaller than the number of adult sons. 如果CB067 <CB068, 则进行检查]

CB069 Which category did the total income of CHILDn's NAME (and his/her spouse) in the past year belong to? [孩子姓名](和他/她配偶) 去年的总收入属于下面哪一类?
1. None 没有收入
2. under 2,000 yuan 少于 2 千元
3. 2，000 – 5，000 yuan 2 千与 5 千之间
4. 5，000 – 10，000 yuan 5 千与 1 万之间
5. 10,000 - 20,000 yuan 在 1 万与 2 万之间
6. 20,000 - 50,000 yuan 在 2 万与 5 万之间

7. 50,000 - 100,000 yuan 在 5 万与 10 万之间
8. 100,000 - 150,000 yuan 在 10 万与 15 万之间
9. 150,000 - 200,000 yuan 在 15 万与 20 万之间
10. 200,000 - 300,000 yuan 在 20 万与 30 万之间
11. Above 300,000 yuan 高于 30 万

CB070 Is [Child's Name] working now (work includes agricultural work, earning wage work, self-employed activities, and unpaid family business work, et al.)? [孩子姓名] 现在工作吗？(工作包括务农、挣工资工作、从事个体、私营活动或不拿工资为家庭经营活动帮工等)

1. Yes 是
2. No 否 → Skip to CB074 跳至CB074

[Softcheck: if the child is reported to be in school and also is reported to be working. 如果这孩子既在上学又在工作，则进行检查。]

[Show Card 出示卡片 5]

CB071 What is [CHILDn's NAME]'s main occupation? [孩子姓名] 现在主要干什么工作？

1. Managers 国家机关、党群组织、企业、事业单位负责人
2. Professionals and technicians 专业技术人员
3. Clerks 办事人员和有关人员
4. Commercial and service workers 商业、服务业人员
5. Agricultural, forestry, husbandry and fishery producers 农、林、牧、渔、水利业生产人员
6. Production and transportation workers 生产、运输设备操作人员及有关人员

CB072 What is the highest administrative level that [CHILDn's NAME] has attained? [孩子姓名] 现在有什么行政职务吗？

1. Team Leader 组长（股长）
2. Section Chief 科长
3. Director of a division 处长
4. Director-General of a bureau and above 局长及以上
5. Township Leader 乡镇干部
6. None 无行政职务

F1：行政职务：是国家行政机关为实施行政管理而设置的国家公职。

CB073 What is/was your [CHILDn's NAME] highest professional/technical level? [孩子姓名] 现在有什么专业/技术职称吗？

1. Technician 技术员
2. Primary level 初级职称
3. Intermediate level 中级职称
4. Advanced level 高级职称
5. None 无专业/技术职称

F1：“专业/技术职称”指专业技术人员的专业技术与学识水平和工作能力的等级称号。

CB074 Has [Child's Name] ever worked before? (Work includes agricultural work, earning wage work, self-employed activities, and unpaid family business work, et al.) [孩子姓名] 以前工作过吗？(工作包括务农、挣工资工作、从事个体、私营活动或不拿工资为家庭经营活动帮工等)

1. Yes 是
2. No 否 → Skip to CB076 BRANCHPOINT 跳至 CB076 BRANCHPOINT

[Show Card 出示卡片 5]

CB075 What sort of work did [CHILDn's NAME] mainly do? [孩子姓名] 以前主要做什么工作？

1. Managers 国家机关、党群组织、企业、事业单位负责人
2. Professionals and technicians 专业技术人员
3. Clerks 办事人员和有关人员
4. Commercial and service workers 商业、服务业人员
5. Agricultural, forestry, husbandry and fishery producers 农、林、牧、渔、水利业生产人员
6. Production and transportation workers 生产、运输设备操作人员及有关人员

CB076 Did [CHILDn's NAME] live with others, away from you and your spouse, before age 16 for more than six months? [孩子姓名] 在 16 岁之前有没有与您和您配偶分开、与别人在一起生活过超过 6 个月？

1. Yes 是
2. No 否 → Repeat questions for additional children 返回问另一个子女

CB077 What was the earliest age that [CHILDn's NAME] lived separately with others for more than six months? [孩子姓名] 最早是在几岁的时候与您和您配偶分开与别人住六个月以上？ ____ 1...16 years of age 岁

[Softcheck: if the age reported here is older than the current age of the child. 如果CB077大于孩子现在的年龄，则进行检查。]

CB078 With whom did [CHILDn's NAME] live for the longest period of time when not living with you nor your spouse? 在不和你们夫妻住在一起的日子里，[孩子姓名] 和谁一起生活的时间最长？

1. Your parents 你的父母
2. Your spouse's parents 你配偶的父母
3. Your brother's or sister's family 你兄弟姐妹的家庭
4. Other family 其他家庭
5. Dormitory 学校宿舍
6. Other 其他

CB079 Cumulatively, how many years did CHILDn's NAME live separately with others before age 16? [孩子姓名]16 岁以前总共与您和您配偶分开与别人住六个月以上的时间有多少年？

____0.00...16.00 (**CB079_1**)years 年 __ 0...11 (**CB079_2**) months 月

PROCEDURE 程序:
Repeat questions for additional children. 返回问另一个子女

A OTHER HOUSEHOLD MEMBER 其他家户成员

[INTRO: Relatives can have important effects on your life. We'd like to ask you some questions about other members of your household. 引语：您的家人对您的生活可能会有重要影响，因此我们想问问除了您和您配偶外的其他家户成员的情况。]

[IWER: The names of other household members excluding MR, spouse, and parents/parents-in-law/children who have been asked in PART C, should be preloaded from PART CM. Ask the following questions on each of the other household members. 访员注意：其他家户成员不包括主要受访者及其配偶，以及在 PART C 家庭部分中已经问过的父母、岳父母（公公婆婆)、子女，其他家户成员的名单要从 CM 部分加载获得。对于其他的每一个家户成员，分别问下面的问题：]

A006 What is the relationship of [household member name] to you? [家户成员姓名] 是您的？

1. Mother 母亲
2. Father 父亲
3. Mother-in-law 岳母/婆婆
4. Father-in-law 岳父/公公
5. Sibling 兄弟姐妹
6. Brother-in-law, sister-in-law 姐夫妹夫/嫂子弟媳
7. Child 孩子
8. Spouse of child 儿媳/女婿
9. Grandchild 孙子女
10. Nanny 保姆
11. Driver 司机
12. Other relative（specify）其他亲戚（请注明）____(**A006_1**)

A002 BRANCHPOINT:
IF THIS IS A NEW HOUSEHOLD MEMBER, GO TO A002.
IF THIS IS AN OLD HOUSEHOLD MEMBER AND GENDER IS NOT MISSING, GO TO A003 BRANCHPOINT.
IF THIS IS AN OLD HOUSEHOLD MEMBER AND GENDER IS MISSING, GO TO A002.

A002 Gender of [household member name] [家户成员姓名] 的性别是？

1. Male 男
2. Female 女

A003 BRANCHPOINT:
IF THIS IS A NEW HOUSEHOLD MEMBER, GO TO A003.
IF THIS IS AN OLD HOUSEHOLD MEMBER AND DATE OF BIRTH IS NOT MISSING, GO TO A004.
IF THIS IS AN OLD HOUSEHOLD MEMBER AND DATE OF BIRTH IS MISSING, GO TO A003.

A003 When was [household member name] born? [家户成员姓名] 的出生日期是?
____(**A003_1**)1900...2013 (HBirthyear) Year 年 __(**A003_2**)0...12 (HBirthmonth) Month 月

[Show Card 出示卡片 1]

A004 What is [household member name]'s marital status? [家户成员姓名] 目前的婚姻状态?
[IWER: Common-law marriage is considered as married. 注意：即使没有领证但自称结婚者也可视为已婚。]
1. Married with spouse present 已婚并与配偶一同居住
2. Married but not living with spouse temporarily for reasons such as work 已婚，但因为工作等原因暂时没有跟配偶在一起居住
3. Separated 分居（不再作为配偶共同生活）
4. Divorced 离异
5. Widowed 丧偶
6. Never married 从未结婚
7. Cohabitated 同居

A009 What is the current hukou status of [household member name]? [家户成员姓名] 目前的户口状态是?
1. Agriculture Hukou 农业
2. Non-Agriculture Hukou 非农业
3. Unified Residency Hukou 统一居民户口
   [IWER: for the place where agricultural hukou is abandoned. 访员注意：对取消农村户口的地方]
4. Do not have Hukou 没有户口

F1："统一居民户口"指的是某些地方实行户口制度改革后，不再区分农业与非农业户口，而是统一为"居民户口"。

PROCEDURE 程序:
Ask each person on the list. 询问名单上的每一个人。

PROCEDURE 程序:
If the person is less than 6 years old, skip to the next person. 如果这人年龄不到 6 岁，请接着询问下一个人

A013 Is [household member name] still in school now? 请问 [家户成员姓名] 现在还在上学吗?

1. Yes 是
2. No 否 → Skip to procedure before A015 请跳至A015之前的程序

[IWER: Fulltime student, not on-the-job student. 访员注意：指全职读书，在职不算]

A014 What level of schooling and grade is [household member name] currently enrolled in? [家户成员姓名] 现在读几年级？

1. Primary school grade 1 小学 1 年级
2. Primary school grade 2 小学 2 年级
3. Primary school grade 3 小学 3 年级
4. Primary school grade 4 小学 4 年级
5. Primary school grade 5 小学 5 年级
6. Primary school grade 6 小学 6 年级
7. Middle school grade 1 初中 1 年级
8. Middle school grade 2 初中 2 年级
9. Middle school grade 3 初中 3 年级
10. Middle school grade 4 初中 4 年级
11. High school, grade 1 高中 1 年级
12. High school, grade 2 高中 2 年级
13. High school, grade 3 高中 3 年级
14. Vocational/technical high school year 1 中专 1 年级
15. Vocational/technical high school year 2 中专 2 年级
16. Vocational/technical high school year 3 中专 3 年级
17. College year 1 大专/大学 1 年级
18. College year 2 大专/大学 2 年级
19. College year 3 大专/大学 3 年级
20. College year 4 大专/大学 4 年级
21. College year 5 大专/大学 5 年级
22. College year 6 大专/大学 6 年及 6 年级以上
23. Master’s degree 硕士
24. Doctoral degree/ Ph.D. degree 博士

PROCEDURE 程序:
If the person is less than 12 years old, skip to the next person. 如果这人年龄不到 12 岁，请询问下一个人

A015 What is [household member name]'s highest level of education completed? (not including adult education) [家户成员姓名] 完成的最高学历是？(不包括成人教育)

1. No formal education (illiterate) 未受过正式教育（文盲）
2. Did not finish primary school but capable of reading or writing 未读完小学，但能够读、写
3. Sishu/home school 私塾
4. Graduate from elementary school 小学毕业

5. Graduate from middle school 初中毕业
6. Graduate from high school 高中毕业
7. Graduate from vocational school 中专（包括中等师范、职高）毕业
8. Graduate from Two/Three Year College / Associate degree 大专毕业
9. Graduate from Four Year College / Bachelor’s degree 本科毕业
10. Graduate from Post-graduate, Master’s degree 硕士毕业
11. Graduate from Post-graduate, Doctoral degree/Ph.D. 博士毕业

PROCEDURE 程序:
Go/Proceed to the next person. 请询问下一个人

C2 TIME TRANSFER AND TRANSFERS 家庭交往与经济帮助

[Introduction: In the following three parts: CD time transfer, CE transfer and CF time spent providing care. we will ask you how you contact with parents and children, and economic trancfers. 接下来的三部分，我们将询问您与父母、子女间的交往和家庭得到及提供的经济帮助。]

CD TIME TRANSFER 与父母、子女间的交往

CONTACT WITH PARENTS 与父母的交往

PROCEDURE 程序:
Skip to CD003 if father/mother/father-in-law/mother-in-law is not alive OR father/mother/father-in-law/mother-in-law is household member. 如果父亲/母亲/岳父(公公)/岳母(婆婆)去世了，或者父亲/母亲/岳父(公公)/岳母(婆婆)是家户成员，跳至CD003.

PROCEDURE 程序:
Repeat each living and non-resident father/mother/father-in-law/mother-in-law for CD002-CD002_W2_1. 循环提问健在的没住在一起的父亲/母亲/岳父(公公)/岳母(婆婆) CD002-CD002_W2_1.

PROCEDURE 程序:
IF PARENTS OR PARENTS-IN-LAW LIVE TOGETHER, THEN ASK CD002_W2_1. 如果父母住在一起或岳父母住在一起，询问CD002_W2_1.

[Show Card 出示卡片 6]

CD002 How often do you/your spouse see your father/mother/ father-in-law/mother-in-law? 您或您的配偶多长时间去看望一次您的父亲/母亲/岳父(公公)/岳母(婆婆)?

1. Almost every day 差不多每天
2. 2-3 times a week 每周 2-3 次
3. Once a week 每周一次

4. Every two weeks 每半个月一次
5. Once a month 每月一次
6. Once every three months 每三个月一次
7. Once every six months 半年一次
8. Once a year 每年一次
9. Almost never 几乎从来没有
10. Other 其他

[Show Card 出示卡片 6]

CD002_W2_1/CD002_W2_2 How often do you/your spouse see your parents/ parents-in-law? 您或您的配偶多长时间去看望一次您的父母/岳父母（公公婆婆）?
1. Almost every day 差不多每天
2. 2-3 times a week 每周 2-3 次
3. Once a week 每周一次
4. Every two weeks 每半个月一次
5. Once a month 每月一次
6. Once every three months 每三个月一次
7. Once every six months 半年一次
8. Once a year 每年一次
9. Almost never 几乎从来没有
10. Other 其他

[IWER: If the father and mother or father-in-law and mother-in-law live together, do not repeat the other one. 访员注意：如果父母亲或者岳父母/公婆两人住在一起，那访问完其中一个人之后不再循环问另一个人。]

CONTACT WITH CHILDREN 与子女的交往

PROCEDURE 程序:
If respondent has no non-cohabiting children, skip to CF001. 如果受访者没有不在一起生活的子女，跳至CF001

PROCEDURE 程序:
For each of the non-coresident child, ask the following two questions CD003-CD004. 对每一个不住在一起的子女，询问以下两个问题CD003-CD004.

[Show Card 出示卡片 6]

CD003 How often do you see CHILDn's NAME? 您多长时间见到 [孩子姓名]?
1. Almost every day 差不多每天
2. 2-3 times a week 每周 2-3 次
3. Once a week 每周一次

4. Every two weeks 每半个月一次
5. Once a month 每月一次
6. Once every three months 每三个月一次
7. Once every six months 半年一次
8. Once a year 每年一次
9. Almost never 几乎从来没有
10. Other 其他

PROCEDURE 程序:
IF CD003 = 1 – 3, skip CD004. 如果CD003 = 1 – 3，跳过CD004.

[Show Card 出示卡片 6]

CD004 How often do you have contact with CHILDn's NAME either by phone, text mess-age, mail, or email, when you didn't live with CHILDn's NAME? 您和 [孩子姓名] 不在一起住的时候，您多长时间跟 [孩子姓名] 通过电话、短信、信件或者电子邮件联系?

1. Almost every day 差不多每天
2. 2-3 times a week 每周 2-3 次
3. Once a week 每周一次
4. Every two weeks 每半个月一次
5. Once a month 每月一次
6. Once every three months 每三个月一次
7. Once every six months 半年一次
8. Once a year 每年一次
9. Almost never 几乎从来没有
10. Other 其他

CE TRANSFERS 家庭得到及提供的经济帮助

[Introduction: Families sometimes help one another in a variety of ways, and each type of help can be important. The next questions are about help you (and your spouse) have given to or received from your non-coresident family members in the past year. 有时候，家庭之间会有多种形式的互相帮助，而每一种形式的帮助都很重要。所以，接下来我们想了解一下，您和您配偶有没有从其他人那里得到或者给其他人什么经济帮助。]

[IWER: money/in-kind support means support living expenses / foodstuff / vegetables / clothes / water and electricity / telephone rate and other daily consumption; Marriage and funeral / move to new house / in hospital/ go to university / new born and other economic transfer. 访员注意：给钱/东西是指提供生活费、粮食、买菜、衣服、水电费及电话费等日常消费支出，以及婚丧嫁娶、搬迁新房、生病、升学、有新生儿及其他情况下的经济往来。]

CE002 BRANCHPOINT:

IF BOTH PARENTS WERE NOT LIVING AT LAST IW OR BOTH OF THEM ARE HOUSEHOLD MEMBERS , OR ONE PARENT WERE NOT LIVING AT LAST IW AND THE OTHER IS A HOUSEHOLD MEMBER , GO TO CE005 BRANCHPOINT

CE002 In the past year, how much economic supports did you or your spouse receive from your non-coresident parents? 过去一年，您或您的配偶从您的不住在一起的父母那里共收到过多少经济支持？

1. Total money support ____ yuan (**CE002_1**), among which how much is regular ____ yuan (**CE002_2**). 总共给钱 ____ 元 (**CE002_1**)，其中，定期给 ____ 元 (**CE002_2**) (for example, support living expenses/water and electricity/telephone rate/return loan or other cost in regular. 如定期提供生活费、支付每月水电费及电话费、支付房贷/房租费用或其他定期的费用)
2. Total in-kind support ____ yuan (**CE002_3**), among which how much is regular ____ yuan (**CE002_4**). 总共给物 ____ 元 (**CE002_3**)，其中，定期给物 ____ 元 (**CE002_4**) (for example, support food/vegetables/clothes or other in-kind support in regular. 如定期提供粮食、买菜、买衣服或其他物品等)

[IWER: Regular means supporting at fixed time such as per month/quarter of a year/half of a year/year, etc. 访员注意：定期是指按月、按季度、按半年、按年给钱或东西，时间上大致固定。]

[IWER: If give no money or in-kind support, please fill in “0”; if respondent answer RF or DK, then ask CE003, or skip to CE021. 访员注意：如果没有给钱或者没有给物，请填入 “0”；拒绝回答或不知道的询问CE003，否则跳至 CE021.]

CE003 Add unfolding brackets (100/200/400/800/1600 yuan) for CE002_1 or CE002_3 answered RF or DK. 请在此处对CE002_1或者CE002_3中回答不知道或拒绝回答的添加分级展开问题（100/200/400/800/1600 元）。

CE022 In the past year, how much economic supports did you or your spouse provide to your non-coresident parents? 过去一年，您或您的配偶给您的不住在一起的父母多少经济支持？

1. Total money support ____ yuan (**CE022_1**), among which how much is regular ____ yuan (**CE022_2**). 总共给钱 ____ 元 (**CE022_1**)，其中，定期给 ____ 元 (**CE022_2**) (for example, support living expenses/water and electricity/telephone rate/return loan or other cost in regular. 如定期提供生活费、支付每月水电费及电话费、支付房贷/房租费用或其他定期的费用)
2. Total in-kind support ____ yuan (**CE022_3**), among which how much is regular ____ yuan (**CE022_4**). 总共给物 ____ 元 (**CE022_3**)，其中，定期给物 ____ 元 (**CE022_4**) (for example, support food/vegetables/clothes or other in-kind support in regular. 如定期提供粮食、买菜、买衣服或其他物品等)

[IWER: Regular means supporting at fixed time such as per month/quarter of a year/half of a year/year, etc. 访员注意：定期是指按月、按季度、按半年、按年给钱或东西，时间上大致固定。]

[IWER: If give no money or in-kind support, please fill in “0”; if respondent answer RF or DK, then ask CE023, otherwise skip to CE005 BRANCHPOINT. 访员注意：如果没有给钱或者没有给物，请填入“0”；拒绝回答或不知道的询问CE023，否则跳至CE005 BRANCHPOINT.]

CE023 Add unfolding brackets (100/200/400/800/1600 yuan) for CE022_1 or CE022_3 answered RF or DK. 请在此处对CE022_1或者CE022_3中回答不知道或拒绝回答的添加分级展开问题（100/200/400/800/1600 元）。

CE005 BRANCHPOINT:
IF BOTH PARENTS-IN-LAW WERE NOT LIVING AT LAST IEW OR BOTH OF THEM ARE HOUSEHOLD MEMBERS, OR ONE PARENT-IN-LAW WERE NOT LIVING AT LAST IW AND THE OTHER IS A HOUSEHOLD MEMBER, GO TO CE009 BRANCHPOINT

CE005 In the past year, how much economic supports did you or your spouse receives from your non-coresident parents-in-law? 过去一年，您或您的配偶从您的没住在一起的岳父母（公公婆婆）那里收到多少经济支持？

1. Total money support _____ yuan (**CE005_1**), among which how much is regular _____ yuan (**CE005_2**). 总共给钱 _____ 元 (**CE005_1**)，其中，定期给 _____ 元 (**CE005_2**) (for example, support living expenses/water and electricity/telephone rate/return loan or other cost in regular. 如定期提供生活费、支付每月水电费及电话费、支付房贷/房租费用或其他定期的费用)
2. Total in-kind support _____ yuan (**CE005_3**), among which how much is regular _____ yuan (**CE005_4**). 总共给物 _____ 元 (**CE005_3**)，其中，定期给物 _____ 元 (**CE005_4**) (for example, support food/vegetables/clothes or other in-kind support in regular. 如定期提供粮食、买菜、买衣服或其他物品等)

[IWER: Regular means supporting at fixed time such as per month/quarter of a year/half of a year/year, etc. 访员注意：定期是指按月、按季度、按半年、按年给钱或东西，时间上大致固定。]

[IWER: If give no money or in-kind support, please fill in “0”; if respondent answer RF or DK, then ask CE006, otherwise skip to CE025. 访员注意：如果没有给钱或者没有给物，请填入“0”；拒绝回答或不知道的询问CE006，否则跳至CE025.]

CE006 Add unfolding brackets (100/200/400/800/1600 yuan) for CE005_1 or CE005_3 answered RF or DK. 请在此处对CE005_1或者CE005_3中回答不知道或拒绝回答的添加分级展开问题（100/200/400/800/1600 元）。

CE025 In the past year, how much economic supports did you or your spouse provide to your non-coresident parents-in-law? 过去一年，您或您的配偶给您的没住在一起的岳父母（公公婆婆）多少经济支持？

1. Total money support _____ yuan (**CE025_1**), among which how much is regular _____ yuan (**CE025_2**). 总共给钱 _____ 元 (**CE025_1**)，其中，定期给 _____ 元 (**CE025_2**) (for example, support living expenses/water and electricity/telephone rate/return loan or other cost in regular. 如定期提供生活费、支付每月水电费及电话费、支付房贷/房租费用或其他定期的费用)
2. Total in-kind support _____ yuan (**CE025_3**), among which how much is regular _____ yuan (**CE025_4**). 总共给物 _____ 元 (**CE025_3**)，其中，定期给物 _____ 元 (**CE025_4**) (for example, support food/vegetables/clothes or other in-kind support in regular. 如定期提供粮食、买菜、买衣服或其他物品等)

[IWER: Regular means supporting at fixed time such as per month/quarter of a year/half of a year/year, etc. 访员注意：定期是指按月、按季度、按半年、按年给钱或东西，时间上大致固定。]

[IWER: If give no money or in-kind support, please fill in “0”; if respondent answer RF or DK, then ask CE026, otherwise skip to CE009 BRANCHPOINT. 访员注意：如果没有给钱或者没有给物，请填入“0”；拒绝回答或不知道的询问CE026，否则跳至CE009 BRANCHPOINT.]

CE026 Add unfolding brackets (100/200/400/800/1600 yuan) for CE025_1 or CE025_3 answered RF or DK. 请在此处对CE025_1或者CE025_3中回答不知道或拒绝回答的添加分级展开问题（100/200/400/800/1600 元）。

CE009 BRANCHPOINT:
IF THE RESPONDENT AND SPOUSE HAVE NO NON-CORESIDENT CHILDREN, GO TO CE012 BRANCHPOINT.

[CAPI: Preload all living children’s names. 加载所有活着的子女姓名。]

PROCEDURE 程序:
For each non-coresident child ask CE009-CE030. 针对每一个不住在一起的孩子询问CE009-CE030.

CE009 In the past year, how much economic supports did you or your spouse receive from your non-coresident children [child name]? 过去一年，您或您的配偶从您的没住在一起的孩子 [孩子姓名] 那里收到过多少经济支持？

1. Total money support _____ yuan (**CE009_1**), among which how much is regular _____ yuan (**CE009_2**). 总共给钱 _____ 元 (**CE009_1**)，其中，定期给 _____ 元 (**CE009_2**) (for example, support living expenses/water and electricity/telephone rate/return loan or other cost in regular. 如定期提供生活费、支付每月水电费及电话费、支付房贷/房租费用或其他定期的费用)
2. Total in-kind support _____ yuan (**CE009_3**), among which how much is regular _____ yuan (**CE009_4**). 总共给物 _____ 元 (**CE009_3**)，其中，定期给物 _____ 元 (**CE009_4**) (for example, support food/vegetables/clothes or other in-kind support in regular. 如定期提供粮食、买菜、买衣服或其他物品等)

[IWER: Regular means supporting at fixed time such as per month/quarter of a year/half of a year/year, etc. 访员注意：定期是指按月、按季度、按半年、按年给钱或东西，时间上大致固定。]

[IWER: If give no money or in-kind support, please fill in “0”; if respondent answer RF or DK, then ask CE010, or skip to CE029. 访员注意：如果没有给钱或者没有给物，请填入“0”；拒绝回答或不知道的询问CE010，否则跳至CE029.]

CE010 Add unfolding brackets (100/200/400/800/1600 yuan) for CE009_1 or CE009_3 answered RF or DK. 请在此处对CE009_1或者CE009_3中回答不知道或拒绝回答的添加分级展开问题（100/200/400/800/1600 元）。

CE029 In the past year, how much economic supports did you or your spouse provide to your non-coresident children [child name]? 过去一年，您或您的配偶给您的没住在一起的[孩子姓名] 多少经济支持？

1. Total money support _____ yuan (**CE029_1**), among which how much is regular _____ yuan (**CE029_2**). 总共给钱 _____ 元 (**CE029_1**)，其中，定期给 _____ 元 (**CE029_2**) (for example, support living expenses/water and electricity/telephone rate/return loan or other cost in regular. 如定期提供生活费、支付每月水电费及电话费、支付房贷/房租费用或其他定期的费用)
2. Total in-kind support _____ yuan (**CE029_3**), among which how much is regular _____ yuan (**CE029_4**). 总共给物 _____ 元 (**CE029_3**)，其中，定期给物 _____ 元 (**CE029_4**) (for example, support food/vegetables/clothes or other in-kind support in regular. 如定期提供粮食、买菜、买衣服或其他物品等)

[IWER: Regular means supporting at fixed time such as per month/quarter of a year/half of a year/year, etc. 访员注意：定期是指按月、按季度、按半年、按年给钱或东西，时间上大致固定。]

[IWER: If give no money or in-kind support, please fill in “0”; if respondent answer RF or DK, then ask CE030, or skip to CE012 BRANCHPOINT. 访员注意：如果没有给钱或者没有给物，请填入“0”；拒绝回答或不知道的询问CE030，否则跳至CE012 BRANCHPOINT.]

CE030 Add unfolding brackets (100/200/400/800/1600 yuan) for CE029_1 or CE029_3 answered RF or DK. 请在此处对CE029_1或者CE029_3中回答不知道或拒绝回答的添加分级展开问题（100/200/400/800/1600 元）。

CE012 BRANCHPOINT:

IF THE RESPONDENT AND SPOUSE HAVE NO NON-CORESIDENT GRANDCHILDREN OR GRANDCHILDREN AGE YOUNGER THAN 16, GO TO CE072_W2.

[CAPI: Preload all the children’s name. 加载所有子女姓名.]

CE012 Including both giving to you and receiving from you, did any of your non-coresident grandchildren have economic supports with you or your spouse in last year, if so, which child is the parent of him/her/them? 过去一年，您或您的配偶与没住在一起的孙子女之间有没有经济往来，包括给你们和你们给经济支持，如果有，是哪个子女的孩子？

1. Choose children's name 子女姓名
99 No grandchildren had economic support 没有孙子女与我或我配偶有经济往来 → Skip to CE072_W2 跳至CE072_W2

[IWER: If there is no grandchildren had economics supports last year, choose the third choice. 访员注意：如果没有孙子女与受访者或其配偶有经济往来，则选择第 3 项.]

[IWER: If there are two or more than two children of R' s one child, then ask the total amount of the grandchildren from the same child. 访员注意：如果受访者一个子女有两个及两个以上的孩子与受访者或其配偶有经济往来，则经济往来的数目是受访者的同一个子女的所有孩子的数目总和。]

PROCEDURE 程序:
Ask CE013-CE034 for each child recorded in CE012. 对CE012中记录的每个子女询问CE013-CE034

CE013 In the past year, how much economic supports did you or your spouse receive from your non-coresident [child name]'s children? 过去一年，您或您的配偶从没跟您住在一起的 [孩子姓名] 的孩子那里收到过多少经济支持？

1. Total money support _____ yuan (**CE013_1**), among which how much is regular _____ yuan (**CE013_2**). 总共给钱 _____ 元 (**CE013_1**)，其中，定期给 _____ 元 (**CE013_2**) (for example, support living expenses/water and electricity/telephone rate/return loan or other cost in regular. 如定期提供生活费、支付每月水电费及电话费、支付房贷/房租费用或其他定期的费用)
2. Total in-kind support _____ yuan (**CE013_3**), among which how much is regular _____ yuan (**CE013_4**). 总共给物 _____ 元 (**CE013_3**)，其中，定期给物 _____ 元 (**CE013_4**) (for example, support food/vegetables/clothes or other in-kind support in regular. 如定期提供粮食、买菜、买衣服或其他物品等)

[IWER: Regular means supporting at fixed time such as per month/quarter of a year/half of a year/year, etc. 访员注意：定期是指按月、按季度、按半年、按年给钱或东西，时间上大致固定。]

[IWER: If give no money or in-kind support, please fill in "0"; if respondent answer RF or DK, then ask CE014, or skip to CE031. 访员注意：如果没有给钱或者没有给物，请填入"0"；拒绝回答或不知道的询问CE014，否则跳至CE031.]

CE014 Add unfolding brackets (100/200/400/800/1600 yuan) for CE013_1 or CE013_3 answered RF or DK. 请在此处对CE013_1或者CE013_3中回答不知道或拒绝回答的添加分级展开问题（100/200/400/800/1600 元）。

CE031 In the past year, how much economic supports did you or your spouse provide to your non-coresident [child name]'s children? 过去一年，您或您的配偶给没跟您住在一起的 [孩子姓名] 的孩子多少经济支持？

1. Total money support _____ yuan (**CE031_1**), among which how much is regular _____ yuan (**CE031_2**). 总共给钱 _____ 元 (**CE031_1**)，其中，定期给 _____ 元 (**CE031_2**) (for example, support living expenses/water and electricity/telephone rate/return loan or other cost in regular. 如定期提供生活费、支付每月水电费及电话费、支付房贷/房租费用或其他定期的费用)
2. Total in-kind support _____ yuan (**CE031_3**), among which how much is regular _____ yuan (**CE031_4**). 总共给物 _____ 元 (**CE031_3**)，其中，定期给物 _____ 元 (**CE031_4**) (for example, support food/vegetables/clothes or other in-kind support in regular. 如定期提供粮食、买菜、买衣服或其他物品等)

[IWER: Regular means supporting at fixed time such as per month/quarter of a year/half of a year/year, etc. 访员注意：定期是指按月、按季度、按半年、按年给钱或东西，时间上大致固定。]

[IWER: If give no money or in-kind support, please fill in “0”; if respondent answer RF or DK, then ask CE034, or skip to CE072_W2. 访员注意：如果没有给钱或者没有给物，请填入“0”；拒绝回答或不知道的询问CE034，否则跳至CE072_W2.]

CE034 Add unfolding brackets (100/200/400/800/1600 yuan) for CE031_1 or CE031_3 answered RF or DK. 请在此处对CE031_1或者CE031_3中回答不知道或拒绝回答的添加分级展开问题（100/200/400/800/1600 元）。

CE072_W2 In the past year, how much economic supports did you or your spouse receive from your non-coresident siblings? 过去一年，您或您的配偶从没住在一起的兄弟姐妹那里收到过多少经济支持？Total money and in-kind support ______ yuan. 总共给钱物 _____ 元

[IWER: If give no money nor in-kind support, please fill in “0”; if respondent answered RF or DK, please ask CE073_W2, or skip to CE074_W2. 访员注意：如果没有给钱物，请填入“0”；拒绝回答或不知道的询问CE073_W2，否则跳至CE074_W2.]

CE073_W2 Add unfolding brackets (100/200/400/800/1600 yuan) for CE072_W2. 请在此处对CE072_W2中回答不知道或拒绝回答的添加分级展开问题（100/200/400/800/1600 元）。

CE074_W2 In the past year, how much economic supports did you or your spouse provide to your non-coresident siblings? 过去一年，您或您的配偶给没住在一起的兄弟姐妹过多少经济支持？Total money and in-kind support ______ yuan. 总共给钱物 ______ 元

[IWER:If give no money nor in-kind support, please fill in “0”; if respondent answered RF or DK, please ask CE075_W2, or skip to CE016. 访员注意：如果没有给钱物，请填入“0”；拒绝回答或不知道的询问CE075_W2，否则跳至CE016.]

CE075_W2 Add unfolding brackets (100/200/400/800/1600 yuan) for CE074_W2. 请在此处对CE074_W2中回答不知道或拒绝回答的添加分级展开问题（100/200/400/800/1600 元）。

CE016 In the past year, how much economic supports did you or your spouse receive from your non-coresident other relatives or friends? 过去一年，您或您的配偶从您的没住在一起的其他亲戚朋友那里收到过多少经济支持？Total money and in-kind support ______ yuan. (for example, marriage and funeral/move to new house/new born/go to university, and economic aid for fall ill or difficult to live, but not including borrowing money). 总共给钱物 ______ 元（给钱是指婚丧嫁娶、搬迁新房、新生儿、子女升学等情况下的随礼，以及生病、生活困难等情况下的经济资助等，但不包括借钱）

[IWER: If give no money nor in-kind support, please fill in "0"; if respondent answered RF or DK, please ask CE017, or skip to CE036. 访员注意：如果没有给钱物，请填入"0"；拒绝回答或不知道的询问CE017，否则跳至CE036.]

CE017 Add unfolding brackets (100/200/400/800/1600 yuan) for CE016. 请在此处对CE016中回答不知道或拒绝回答的添加分级展开问题（100/200/400/800/1600 元）。

CE036 In the past year, how much economic supports did you or your spouse provide to your non-coresident other relatives or friends? 过去一年，您或您的配偶给您的没住在一起的其他亲戚朋友过多少经济支持？Total money and in-kind support ______ yuan. (for example, marriage and funeral/move to new house/new born/go to university, and economic aid for fall ill or difficult to live, but not including borrowing money). 总共给钱物 ______ 元（给钱是指婚丧嫁娶、搬迁新房、新生儿、子女升学等情况下的随礼，以及生病、生活困难等情况下的经济资助等，但不包括借钱）

[IWER:If give no money nor in-kind support, please fill in "0"; if respondent answered RF or DK, please ask CE037, or skip to CE066_W2. 访员注意：如果没有给钱物，请填入"0"；拒绝回答或不知道的询问CE037，否则跳至CE066_W2.]

CE037 Add unfolding brackets (100/200/400/800/1600 yuan) for CE036. 请在此处对CE036中回答不知道或拒绝回答的添加分级展开问题（100/200/400/800/1600 元）。

CE066_W2 BRANCHPOINT:
IF [CHILDN'S NAME] HAS MARITAL HISTORY (CB063 = 1/2/3/4/5/7), ASK CE066_W2

[CAPI: Preload each child that is not unmarried. 加载每一个非未婚的子女。]

[IWER: please ask about each child CE066_W2-CE070_W2. 访员注意：询问每一个非未婚的子女CE066_W2-CE070_W2。]

[IWER: If the child got married more than one times, please ask about the first one. 访员注意：如果子女结婚次数大于 1 次，则询问第 1 次结婚的情形。]

CE066_W2 When did your [childn's name] get married? [孩子姓名] 是什么时候结婚的？
____1900...2013 (**CE066_W2_1_1**) year 年 __0...12 (**CE066_W2_1_2**) month 月 __0...31 (**CE066_W2_1_3**) day 日

CE067_W2_1 Did you give betrothal gifts when [child name] got married? [孩子姓名] 结婚时您给彩礼了吗?

1. Yes 是
2. No 否 → Skip to CE069_W2_1 跳至CE069_W2_1

CE068_W2_1 At that time, how much was the total value of the betrothal gifts? 在当时的物价水平下，这些彩礼值总共值多少钱? ____ 万元

CE069_W2_1 Did you buy a house for him/her when [child name] got married? [孩子姓名] 结婚时您给买房了吗?

1. Yes 是
2. No 否 → Skip to CF001 跳至CF001

CE070_W2_1 At that time, how much was the total value of the house? 在当时的物价水平下，这房子值多少钱? ____ 万元

CF TIME SPENT PROVIDING CARE 提供照料时间

PROCEDURE 程序:

If the respondent has any grandchildren under 16, ask CF001 - CF003; otherwise, skip to CF004. 如果受访者有 16 岁以下的未成年孙子/孙女，提问CF001 - CF003；否则，请跳至CF004 .

CF001 Did you spend any time taking care of your grandchildren or great-grandchildren last year? 过去一年，您或您配偶是否花时间照看了您的孙子女或重孙子女?

1. Yes 是
2. No 否 → Skip to the procedure before CF004 跳至CF004之前的程序

CF002 For which child's children or grandchildren did you provide care? 您或您配偶照看哪一个子女的孩子或孙子女?

[IWER: Please list all the children including coresident ones, and add a choice "deceased children" in CAPI list. Select from list displayed by CAPI (child's name) 访员注意：请列出所有人，包括在一起住的孩子和去世的子女名单，从孩子名单中选出]

PROCEDURE 程序:

Repeat question CF003 according to the list of names in CF002. 根据CF002 的名单重复问CF003

CF003 Approximately how many weeks and how many hours per week did you spend last year taking care of this child's children or grandchildren? 过去一年，您和您配偶大约花几周，每周花多少时间来照看这个子女的孩子或孙子女? Myself 我 ____ (**CF003_1**)weeks 周; ____ (**CF003_2**) 0.00...168.00 hours per week 小时/周, My spouse 我爱人 ____

(**CF003_3**) weeks 周; ____ (**CF003_4**) 0.00...168.00 hours per week 小时/周
[IWER: Mark '1' if the period is less than 7 days. 访员注意：如果小于 7 天填写“1”]
[Hardcheck: if more than 52 weeks are reported or more than 140 hours are reported. 如果答案大于 52 周或者大于 140 小时。]

PROCEDURE 程序:
If both parents were not living at last IW or both of them are household members, or one parent were not living at last IW and the other is a household member, go to CF007_W2.

CF004 Did you or your spouse take care of your parents or parents-in-law during the last year in assisting them in their daily activities or other activities (e.g., household chores, meal preparation, laundry, going out, grocery shopping, financial management, etc.)? 过去一年，您或您配偶有没有在日常活动（或其他活动）方面给您父母或您配偶的父母提供帮助(例如家务劳动，做饭，洗衣，外出，购物和财务管理)?

1. Yes 是
2. No 否 → Skip to next module 跳至下一个模块

CF005 Approximately how many weeks and how many hours per week did you yourself spend last year taking care of your parents or parents-in-law? 过去一年，您自己大约花几周，每周花多少时间来照看您的父母和岳父母（或公公婆婆)?

1. Your father 您父亲 __ (**CF005_1**) weeks 周; ___ (**CF005_2**) 0.00...168.00 hours per week 小时/周
2. Your mother 您母亲 __ (**CF005_3**) weeks 周; ___ (**CF005_4**) 0.00...168.00 hours per week 小时/周
3. Your father-in-law 您的岳父（或公公）__ (**CF005_5**) weeks 周; ___ (**CF005_6**) 0.00...168.00 hours per week 小时/周
4. Your mother-in-law 您的岳母（或婆婆）__ (**CF005_7**) weeks 周; ___ (**CF005_8**) 0.00...168.00 hours per week 小时/周

[Softcheck: if (1) is checked in CF004 and 0 or missings are reported in CF005. 如果CF004 = 1，而且CF005 = 0 或答案缺失]
[Softcheck: if more than 52 weeks are reported or more than 140 hours are reported. 如果答案大于 52 周或者大于 140 小时]

CF006 Approximately how many weeks and how many hours per week did your spouse spend last year taking care of your parents or parents-in-law? 过去一年，您的配偶大约花几周，每周花多少时间来照看您的父母和岳父母（或公公婆婆)?

1. Your father 您父亲 __ (**CF006_1**) weeks 周; ___ (**CF006_2**) 0.00...168.00 hours per week 小时/周
2. Your mother 您母亲 __ (**CF006_3**)weeks 周; ___ (**CF006_4**) 0.00...168.00 hours per week 小时/周
3. Your father-in-law 您的岳父（或公公）__ (**CF006_5**) weeks 周; ___ (**CF006_6**) 0.00...168.00 hours per week 小时/周

4. Your mother-in-law 您的岳母（或婆婆）__ (**CF006_7**) weeks 周; ___ (**CF006_8**) 0.00...168.00 hours per week 小时/周

[Softcheck: if (1) is checked in CF004 and 0 or missings are reported in CF006. 如果CF004 = 1, 而且CF006 = 0 或答案缺失]

[Softcheck: if more than 52 weeks are reported or more than 140 hours are reported. 如果答案大于 52 周或者大于 140 小时]

CF007_W2 How often did the respondent receive assistance in answering sections CB, CD, CE and CF? 受访者填写 CB, CD, CE 和 CF 部分问卷时是否求助?

[IWER: If it is answered by a proxy, please record the respondent's reaction. If the Family R isn't in or can't answer questions, and proxyed by his/her spouse, please choose the (4). 访员注意：如果是协助回答，请记录受访者的反应。如果家庭受访者不在家或者无法回答问题，而是由其配偶回答的，请选择（4）。]

1. Never 从未
2. A few times 偶尔几次
3. Most or all of the time 大多数
4. Proxy by the spouse. 由家庭受访者的配偶代理回答

D HEALTH STATUS AND FUNCTIONING 健康状况和功能

DA HEALTH STATUS 健康状况

Type of Interview R 受访者类型		
XRType = REIW	This is a reinterview R	受访者类型为回访受访者
XRType = NEWIW	This is a new interview R	受访者类型为新受访者
XRType = EXITIW	This is a exit R	受访者类型为退出受访者

Gender of Interview R 受访者性别		
R IS MALE	(XRGender = 1)	受访者为男性 (XRGender = 1)
R IS FEMALE	(XRGender = 2)	受访者为女性 (XRGender = 2)

R's LAST IW Time (ZIWTime) 上次访问的日期 (ZIWTime)

**HEALTH CONDITIONS REPORTED IN LAST WAVE INTERVIEW: 基线访问时受访者报告的健康状况**	
ZDA005[i]	IF ZDA005[i]=Yes, R had kind of disabilities listed in DA005 at ZIWTime
ZDA006[i]	IF ZDA006[i]=Yes, R reported disabled time at ZIWTime
ZDA007[1]	IF DA007[1]=Yes, ZDA007[1]=Yes, R had hypertension at ZIWTime
ZDA007[2]	IF DA007[2]=Yes, ZDA007[2]=Yes, R had dyslipidemia at ZIWTime
ZDA007[3]	IF DA007[3]=Yes, ZDA007[3]=Yes, R had diabetes or high blood sugar at ZIWTime
ZDA007[4]	IF DA007[4]=Yes, ZDA007[4]=Yes, R had cancer or malignant tumor (excluding minor skin cancers) at ZIWTime
ZDA007[5]	IF DA007[5]=Yes or DA008[5]=Yes, ZDA007[5]=Yes, R had chronic lung diseases, such as chronic bronchitis, emphysema at ZIWTime
ZDA007[6]	IF DA007[6]=Yes, ZDA007[6]=Yes, R had liver disease at ZIWTime
ZDA007[7]	IF DA007[7]=Yes, ZDA007[7]=Yes, R had heart attack, coronary heart disease, angina, congestive heart failure, or other heart problems at ZIWTime
ZDA007[8]	IF DA007[8]=Yes, ZDA007[8]=Yes, R had stroke at ZIWTime
ZDA007[9]	IF DA007[9]=Yes, ZDA007[9]=Yes, R had kidney disease at ZIWTime
ZDA007[10]	IF DA007[10]=Yes, ZDA007[10]= Yes, R had stomach or other digestive disease (except for tumor or cancer) at ZIWTime
ZDA007[11]	IF DA007[11]= Yes, ZDA007[11]= Yes, R had emotional, nervous, or psychiatric problems at ZIWTime
ZDA007[12]	IF DA007[12]= Yes, ZDA007[12]= Yes, R had memory-related disease at ZIWTime

ZDA007[13]	IF DA007[13]= Yes, ZDA007[13]= Yes, R had arthritis or rheumatism at ZIWTime
ZDA007[14]	IF DA007[14]= Yes, ZDA007[14]= Yes, R had asthma at ZIWTime
ZDA008[1]	IF ZDA008[1]= Yes, R had known R had hypertension at ZIWTime
ZDA008[5]	IF ZDA008[5]= Yes, R had known R had chronic lung diseases at ZIWTime
ZDA008[11]	IF ZDA008[11]= Yes, R had known R had emotional problems at ZIWTime
ZDA009[i]	When was the condition first dignosed or known by yourself?
ZDA026	Began menarche time
ZDA027	IF DA027=Yes, ZDA027=Yes, R had started menopause at R's last interview
ZDA028	Time of menopause
ZDA036	IF DA036= 1, ZDA036= 1, R had cataract surgery for one eye at ZIWTime
ZDA037	IF DA037=Yes, ZDA037=Yes, R had Glaucoma at ZIWTime
ZDA038	IF DA038=Yes, ZDA038=Yes, R had ever wear hearing aid at ZIWTime
ZDA040	IF ZDA040=Yes, R had lost all teeth at ZIWTime
ZDA048	How would you evaluate your health during childhood, up to and including age 15?
ZDA059	IF DA059=Yes, ZDA059=Yes, R had ever smoked at R's last interview

NOTE: NOTE ON PRELOADED HEALTH CONDITIONS:
IN THIS SECTION MUCH OF THE FLOW OF THE INTERVIEW AND THE PHRASING OF THE QUESTIONS DEPENDS ON WHETHER THE RESPONDENT REPORTED HAVING CERTAIN HEALTH CONDITIONS IN A PREVIOUS IW AND/OR CONFIRMED THEM IN THE LAST IW.
这一模块大部分的流量和问题的问法取决于受访者在上一期访问时报告的身体状况。

DA HEALTH STATUS 健康状况

PROCEDURE 程序
SKIP PATTERN CHECKPOINT: SELF-REPORTED HEALTH STATUS
TWO SCALES ARE USED TO MEASURE SELF-REPORTED HEALTH STATUS. R WILL BE ASKED TO RATE THEIR HEALTH STATUS TWICE, ONCE AT THE BEGINNING OF THIS SECTION AND AGAIN AT THE END OF THE SECTION. QUESTION ORDER WILL BE ASSIGNED RANDOMLY.

IF R IS RANDOMLY ASSIGNED TO ORDER 1(SEC_DA_LIST= 1), SKIP TO DA001。
IF R IS RANDOMLY ASSIGNED TO ORDER 2(SEC_DA_LIST= 2), SKIP TO DA002。

跳转检查：自我报告健康状况
在这个部分中，我们将要求受访者完成两次自我健康状况评价。第一次是在这个部分的开头；第二次则放在这个部分的结尾处。在问卷中自我评价的问题以随机的形式出现。

如果受访者被随机分在第一组（DA 部分 = 1），跳至 DA001。
如果受访者被随机分在第二组（DA 部分 = 2），跳至 DA002。

PART I: GENERAL HEALTH STATUS AND DISEASE HISTORY 第一部分：一般健康状况和疾病史

DA001 Next, I have some questions about your health. Would you say your health is excellent, very good, good, fair, or poor? 接下来我们问一些与您健康状况相关的问题。您觉得您的健康状况怎么样？是极好，很好，好，一般，还是不好？[IWER：Interviewer should read all the following options 访员注意：必须读出所有的选项]

1. Excellent 极好
2. Very good 很好
3. Good 好
4. Fair 一般
5. Poor 不好

DA002 Next, I have some questions about your health. Would you say your health is very good, good, fair, poor or very poor? 下面我将问到一些关于您的健康状况的问题。您认为您的健康状况怎样？是很好，好，一般，不好，还是很不好？[IWER：Interviewer should read all the following options 访员注意：必须读出所有的选项]

1. Very good 很好
2. Good 好
3. Fair 一般
4. Poor 不好
5. Very poor 很不好

**PROCEDURE 程序**:
IF XRType = REIW, ASK DA002_W2_1. 如果是回访受访者，询问DA002_W2_1。

DA002_W2_1 Compared with your health when we talked with you in R's LAST IW MONTH, YEAR, would you say that your health is better now, about the same, or worse? 与上一次访问时 [加载日期] 您的健康相比，您觉得您的健康状况变好了，差不多，还是变差了？

1. Better 变好
2. About the same 差不多
3. Worse 变差

[IWER: Please do not ask proxy the following questions from DA003-DA004. 访员注意：DA003-DA004 题不允许请代理人回答。]

DA003 **PROCEDURE 程序**: If XRType = NEWIW 如果是新的受访者

Do you ever feel pain on the left side of your chest? 您左侧胸部曾感到过疼痛吗？

PROCEDURE 程序: If XRType = REIW 如果是回访受访者

Do you ever feel pain on the left side of your chest [since R's LAST IW MONTH, YEAR/ in the last two years]? 自上次访问以来（加载上次访问时间），您左侧胸部感到过疼痛吗？

1. Yes 是
2. No 否

DA004 **PROCEDURE 程序**: If XRType = NEWIW 如果是新的受访者

Do you ever feel chest pains when climbing stairs/uphill or walking quickly? 当您爬楼梯、登山或快速走的时候，曾感到过胸部疼痛吗？

PROCEDURE 程序: If XRType = REIW 如果是回访受访者

Do you ever feel chest pains when climbing stairs/uphill or walking quickly [since R's LAST IW MONTH, YEAR/ in the last two years]? 自上次访问以来（加载上次访问时间），当您爬楼梯、登山或快速走的时候，感到过胸部疼痛吗？

1. Yes 是
2. No 否
3. Not applicable 不适用

DA005 BRANCHPOINT:

IF XRType = REIW WHO DID HAVE DISABILITIE IN LAST IW TIME (ZDA005[i]=yes), GO TO DA007. 如果是上期回答有此项残疾的回访受访者，跳至DA007。

IF THIS IS A NEW INTERVIEW R OR THIS IS A REINTERVIEW R WHO REPORTED NO IN LAST WAVE (ZAD005[i]=no). 如果是新的受访者或上期回答没有此项残疾的回访受访者询问：

DA005 Do you have one of the following disabilities? 您是否有下列残疾问题？

1. Physical disabilities 躯体残疾
2. Brain damage/mental retardation 大脑受损/智力缺陷
3. Vision problem 失明或半失明
4. Hearing problem 聋或半聋
5. Speech impediment 哑或严重口吃

DA006 **PROCEDURE 程序**: If XRType = NEWIW AND DA005[i] = 1 如果是新的受访者回答患有残疾问题

In what year did you become disabled? 您是什么时候开始患有 [preload DA005]？

PROCEDURE 程序: If XRType = REIW AND ZDA005[i] = null 如果是回访受访者上期访问时DA006题未回答：

Our records from your last interview in R's LAST IW MONTH, YEAR show that you have had [preload ZDA005] 上一次的访问记录显示您患有 [...], In what year did you become disabled? 您是什么时候开始患有 [preload ZDA005]？

____Year 年

[IWER: Mark the year using four digits. 访员注意：用 4 位数表示年。]

[IWER: If the records from last wave is wrong, please note -9999. 访员注意：如果从未患有此项残疾，请填 -9999。]

PROCEDURE 程序:

IF XRType = NEWIW 如果是新的受访者

[Show Card 出示卡片 7]

DA007 Have you been diagnosed with [conditions listed below, read one by one] by a doctor? 是否有医生曾经告诉过您有以下这些慢性病?

1. Hypertension 高血压病
2. Dyslipidemia (elevation of low density lipoprotein, triglycerides (TGs),and total cholesterol, or a low high density lipoprotein level) 血脂异常（高血脂或低血脂）
3. Diabetes or high blood sugar 糖尿病或血糖升高（包括糖耐量异常和空腹血糖升高）
4. Cancer or malignant tumor (excluding minor skin cancers) 癌症等恶性肿瘤（不包括轻度皮肤癌）
5. Chronic lung diseases, such as chronic bronchitis , emphysema (excluding tumors, or cancer) 慢性肺部疾患如慢性支气管炎或肺气肿、肺心病（不包括肿瘤或癌）
6. Liver disease (except fatty liver, tumors, and cancer) 肝脏疾病（除脂肪肝、肿瘤或癌外）
7. Heart attack, coronary heart disease, angina, congestive heart failure, or other heart problems 心脏病（如心肌梗塞、冠心病、心绞痛、充血性心力衰竭和其他心脏疾病）
8. Stroke 中风
9. Kidney disease (except for tumor or cancer) 肾脏疾病（不包括肿瘤或癌）
10. Stomach or other digestive disease (except for tumor or cancer) 胃部疾病或消化系统疾病（不包括肿瘤或癌）
11. Emotional, nervous, or psychiatric problems 情感及精神方面问题
12. Memory-related disease 与记忆相关的疾病（如老年痴呆症、脑萎缩、帕金森症）
13. Arthritis or rheumatism 关节炎或风湿病
14. Asthma 哮喘

NOTE: The screen displays whether or not this condition was reported in R's LAST IW 屏幕上显示受访者上期访问时是否有慢性病

DA007_W2_1 Our records from your last interview in R's LAST IW MONTH, YEAR show that you have had/not had [conditions listed below], is this right? 上一次的访问记录显示您患有/没有 [加载慢性病], 这个记录对吗?

1. Agree 上次访问记录正确
2. Disagree 上次访问记录错误

PROCEDURE 程序:

For R reported in last iw that he/she had [conditions listed below] (ZDA007[i] = Yes OR ZDA008[i] = Yes) 受访者上次访问时报告患有 [加载慢性病]:

If DA007_W2_1 = 1, skip to next loop 如果上次访问正确，询问下一个慢性病

If DA007_W2_1 = 2, skip to DA007_W2_2 如果上次访问错误，询问上次访问以后是否患有此项慢性病DA007_W2_2

PROCEDURE 程序:

For R reported in last iw that he/she did not have [conditions listed below],(ZDA007[i] = not yes OR ZDA008[i] = not yes) 受访者上次访问时报告没有患有 [加载慢性病]:

If DA007_W2_1 = 1, skip to DA007_W2_2 如果上次访问正确，询问上次访问以后是否患有此项慢性病DA007_W2_2

If DA007_W2_1 = 2, skip to EXDA009 如果上次访问错误，说明上次访问已经患有此项慢性病，询问患病时间DA009

DA007_W2_2 Have you been diagnosed with [conditions listed below, read one by one] by a doctor [since R's LAST IW MONTH, YEAR/ in the last two years]? 自上次访问以后（过去两年），是否有医生曾经告诉过您有以下这些慢性病？

1. Yes 是 skip to DA008_W2_1 跳至DA008_W2_1
2. No 否 skip to next loop 开始下一个慢性病的循环

DA008 BRANCHPOINT:

For XRType = REIW and XRType = NEWIW, ask DA008 if $i = 1, 5, 11$, and DA007[i] = no or DA007_W2_1[i] = 3 or 4. 对于每个DA007和DA007_W2_1的第 1，5，11 选项中回答否的选项，问DA008

DA008 Do you know if you have [preload the current choice in DA007]? 您是否知道自己患有？[依次显示所有DA007 中第 1，5，11 选项中回答否的选项的疾病名称]

1. Yes 知道自己患有
2. No 知道自己没有
3. Don't know 不知道有没有

PROCEDURE 程序:

IF DA007[i] = 1 or DA007_W2_1[i] = 1 or DA008[i] = 1 如果医生诊断或自我诊断患有慢性病：

DA008_W2_1 How did you know that you had had [preload disease], through routine or charls physical examination, or any other? 您是通过何种方式知道自己患有 [加载慢性病] 的，是生病后体检，[加载慢性病] 发作后体检，常规体检，charls 体检还是其他方式？

1. Physical examination after had [preload disease] attack [加载慢性病] 发作后体检
2. Physical examination after had ill 生病后体检
3. Physical examination organized by work unit 单位组织的体检
4. Physical examination organized by community 社区组织的体检
5. charls physical examination charls 体检
6. other 其他方式

DA009 BRANCHPOINT:

For XRType = REIW, XRType = NEWIW and XRType = EXITIW, ask DA009, if DA007[i]= 1 or DA008[i]= 1 or ZDA009[i]= null. 如果医生诊断或自我诊断患有慢性病，或者上期访问时DA009 题未答，询问DA009。

DA009 When was the condition first diagnosed or known by yourself? 第一次诊断出或者自己知道患有 [...] 在 ____(**DA009_1**) Year 年或 ____(**DA009_2**) Age 岁？[IWER: Mark the year using four digits. 访员注意：用 4 位数记录年份。]

PROCEDURE 程序:
Answer DA010 if you have No. 2, 5, 6, 7, 9, 10, 12, 13 chronic dieases of DA007. 如果医生诊断或自己知道患有DA007中第 2，5，6，7，9，10，12，13 项慢性病，回答DA010。

DA010 Are you now taking any of the following treatments to treat [...] or its complications (Check all that apply)? Taking Chinese traditional medicine, taking Western modern medicine, other treatments? 您目前有没有正在采用以下方式来治疗 [...] 及其并发症 (选择所有采用的方式)？服用中药，服用西药，吃药以外的其他治疗方法？（可多选）[IWER: Read one by one. 访问员注意：请逐项读出以下答案，并让受访者逐一回答。]

1. Taking Chinese traditional medicine 服用中药 (**DA010_1**)
2. Taking Western morden medicine 服用西药 (**DA010_2**)
3. Other treatments 吃药以外的其他治疗方法 (**DA010_3**)
4. None of the above 以上都没有 (**DA010_4**)

PROCEDURE 程序:
Answer DA010_W2_1 if you have hypertension or diabetes. 如果医生诊断或自己知道患有高血压或糖尿病，回答DA010_W2_1。

DA010_W2_1 Is your [Blood pressure/ sugar] generally under control? 您的 [血压/血糖] 现在是否控制住了？

1. Yes 是
2. No 否

PROCEDURE 程序:
IF XRType = REIW that had condition in last wave (ZDA007[i] = yes and ZDA007_W2_1[i]= 1): 如果是回访受访者，上次访问时的确患有 [...] 慢性病：

DA010_W2_2 Compared to when we interviewed you in R's LAST IW MONTH, YEAR, is your condition better, about the same as it was then or worse? 与上次访问相比 (加载上期访问时间)，您的 [...] 好一些了，跟原来差不多还是更差？

1. Better 更好
2. Same 与原来差不多
3. Worse 更差

PROCEDURE 程序: If respondents have hypertension then answer DA011. 如果医生诊断或自我诊断患有高血压，回答DA011。

DA011 Are you now taking any of the following treatments to treat or control your hypertension?(Check all that apply) Taking Chinese traditional medicine, taking Western modern medicine? 您目前有没有正在采用以下方式来治疗控制高血压？服用中药，服用西药？（可多选）[IWER: Read one by one. 1 = yes, 2 = no. 访问员注意：请逐项读出以下答案，并让受访者逐一回答，1 = 是，2 = 否。]

1. Taking Chinese traditional medicine 服用中药
2. Taking Western modern medicine 服用西药
3. None of the above 以上都没有

DA011_W2_1 **PROCEDURE 程序**: If XRType = REIW 如果是回访受访者

Since R's LAST IW MONTH, YEAR, have you had your blood pressure checked by a doctor or nurse? 自上次访问以来 [加载上次访问日期]，是否曾有医生或护士给您检查血压？

PROCEDURE 程序: OTHERWISE:

Have you ever had your blood pressure checked by a doctor or nurse? 是否曾有医生或护士给您检查血压？

1. Yes 是
2. No 否 skip DA011_W2_2 and DA012

DA011_W2_2 When did you last have it checked? 最近一次检查是什么时候？

Year 年 ____ Month 月 ____

DA012 During last year (last 12 months), how many times have you had blood pressure examination? 在过去一年（12 个月）中，您检查过几次血压？

____0 . . . 999 Times 次

DA013 Have your care providers ever given you health education/advice on the following (check all that apply)? Weight control, exercise, diet and/or smoking control? 有没有医生建议您注意以下问题？（可多选）控制体重，身体锻炼，饮食调理，控烟？ [IWER: Read one by one. 1=yes, 2=no. 访问员注意：请逐项读出以下答案，并让受访者逐一回答，1= 是，2= 否。]

1. Weight control 控制体重
2. Exercise 身体锻炼
3. Diet 饮食调理
4. Smoking control 控烟
5. None of the above 以上都没有

PROCEDURE 程序: If respondents have diabetes, then answer DA014-DA016. 如果受访者患有糖尿病，回答DA014-DA016。

DA014 Are you now taking any of the folloing treatments to treat or control your diabetes?(Check all that apply) Taking Chinese traditional medicine, taking Western morden medicine ,taking insulin injections? 您目前有没有正在采用以下方式来治疗控制糖尿病？服用中药，服用西药，注射胰岛素？（可多选） [IWER: Read one by one. 访问员注意：请逐项读出以下答案，并让受访者逐一回答]

1. Taking Chinese traditional medicine 服用中药
2. Taking Western modern medicine 服用西药
3. Taking insulin injections 注射胰岛素

4. None of the above 以上都没有

DA015 During last year (last 12 months), how many times have you had the following? 过去一年（12 个月）中，下列各种检测您分别做了几次？

1. Blood glucose test 血糖检测 (**DA015_1**)____ 0...999 Times 次
2. Urine glucose test 尿糖检测 (**DA015_2**)____ 0...99 Times 次
3. Fundus examination 眼底检查 (**DA015_3**)____ 0...99 Times 次
4. Micro-albuminuria test 微蛋白尿检查 (**DA015_4**)____ 0...99 Times 次
5. None of the above 以上都没有

DA016 Have your care providers ever given you health education/advice on the following? (check all that apply) 有没有医生建议您注意以下问题？控制体重，身体锻炼，饮食调理，控烟，足部自我护理？（可多选）[IWER: Read one by one. 1=yes, 2=no. 访问员注意：请逐项读出以下答案，并让受访者逐一回答，1= 是，2= 否。]

1. Weight control 控制体重
2. Exercise 身体锻炼
3. Diet 饮食调理
4. Smoking control 控烟
5. Foot self-care 足部自我护理
6. None of the above 以上都没有

PROCEDURE 程序：If reinterview and exit respondents have heart attack, then answer DA007_W2_5. 如果回访和退出受访者患有心脏病，回答DA007_W2_5。

DA007_W2_5 [Since R's LAST IW MONTH, YEAR/In the last two years], have you had a heart attack? 自上次访问以来的两年内，您是否发作过心脏病？

1. Yes 是
2. No 否 → Skip to DA007_W2_6

DA007_W2_6 When was [his/her] (most recent) heart attack? 最近一次发病是什么时候？

____ Year 年 ____Month 月

PROCEDURE 程序：If respondents have cancer or maligant tumor (excluding minor skin cancers), answer DA017 and DA018. 如果受访者患有癌症或恶性肿瘤（不包括轻度皮肤癌），回答DA017 及 DA018。

[Show Card 出示卡片 8]

DA017 In which organ or part of your body do you have cancer? Including the origins and metastasis of tumor.（circle all that apply）身体的哪个器官或部位患有或曾经患有癌症？包括原发和已转移的肿瘤。（可多选）[IWER: Read one by one. We should still ask R even if he/she has already been cured. 访员注意：依次读出所有选项，若受访者所患癌症已痊愈，仍需记录。]

1. Brain 大脑

2. Oral cavity 口腔
3. Larynx 喉
4. Other pharynx 咽
5. Thyroid 甲状腺
6. Lung 肺
7. Breast 乳房
8. Oesophagus 食管
9. Stomach 胃
10. Liver 肝脏
11. Pancreas 胰腺
12. Kidney 肾脏
13. Prostate 前列腺
14. Testicle 睾丸
15. Ovary 卵巢
16. Cervix 子宫颈
17. Endometrium 子宫内膜
18. Colon or rectum 结肠或直肠
19. Bladder 膀胱
20. Skin 皮肤
21. Non-Hodgkin lymphoma 非何杰金淋巴瘤（非霍奇金淋巴瘤）
22. Leukemia 白血病
23. Other organ 其他器官 (**DA017_1**)

DA018 Have you taken any of the following treatments to treat your cancer or relieve its/their symptoms (e.g., pain, nausea, etc.) in the past two years? (Check all that apply) Taking Chinese traditional medicine ,taking Western modern medicine ,chemotherapy ,surgery ,radiation therapy? 在过去的两年中，您有没有采用以下方式来治疗肿瘤或缓解肿瘤所引起的疼痛、恶心等症状 (可多选)？服用中药，服用西药，化学疗法，手术治疗，放射疗法？[IWER: Read one by one. 访问员注意：请逐项读出以下答案，并让受访者逐一回答。]

1. Taking Chinese traditional medicine 服用中药
2. Taking Western morden medicine 服用西药
3. Chemotherapy 化学疗法
4. Surgery 手术治疗
5. Radiation therapy 放射疗法
6. None of the above 以上都没有

[F1：(1)“化学疗法”指用化学合成药物治疗疾病的方法。化学药物治疗 (简称化疗) 是目前治疗肿瘤及某些身免疫性疾病的主要手段之一。
(2)“手术治疗”是最早应用的治疗癌症的方法，也是目前许多早期癌症治疗的首选疗法。
(3)“放射疗法”是用各种不同能量的射线照射肿瘤，以抑制和杀灭癌细胞的一种治疗方法。]

PROCEDURE 程序：If respondents have stroke, then answer DA019. 如果受访者患有中风，回答DA019。

DA019 Are you now taking any of the folloing treatments because of your stroke?(Check all that apply) Taking Chinese traditional medicine, taking Western modern medicine, physical therapy, acupuncture and moxibustion, occupational therapy? 您目前有没有正在采用以下方式来治疗或控制由于中风引起的痉挛或者其它并发症？服用中药，服用西药，物理治疗，针灸治疗，职业治疗？（可多选）[IWER: Read one by one. 1 = yes, 2 = no. 访问员注意：请逐项读出以下答案，并让受访者逐一回答，1 = 是，2 = 否。]

1. Taking Chinese traditional medicine 服用中药
2. Taking Western morden medicine 服用西药
3. Physical therapy 物理治疗
4. Acupuncture and moxibustion 针灸治疗
5. Occupational therapy 职业治疗
6. None of the above 以上都没有

F1：（1）“物理治疗”指主要借着自然界中的物理因子（声光水冷电热力）、运用人体生理学原理法则等，针对人体局部或全身性的功能障碍或病变，施予适当的非侵入性、非药物性治疗来处理患者身体不适和病痛治疗方式，使其尽可能地恢复其原有的生理功能。
（2）“针灸治疗”指针法和灸法的合称。针法是把毫针按一定穴位刺入患者体内，运用捻转与提插等针刺手法来治疗疾病。灸法是把燃烧着的艾绒按一定穴位熏灼皮肤，利用热的刺激来治疗疾病。
（3）“职业治疗”指透过悉心选择而适用于病者的康复活动，帮助在精神、情绪、体能或智能上有障碍的人恢复/增强身体及心智功能，阻止/减轻伤残所带来的影向，使他们能适应工作，社会及家庭上的要求，达至正常和独立的生活。]

PROCEDURE 程序：If reinterview respondents have stroke 如果回访受访者上次访问的确患有中风：

DA019_W2_1 Since R’s LAST IW MONTH, YEAR, has a doctor told you that you had another stroke? [加载上次访问日期] 自上次访问以来，是否有医生诊断您中风复发？

1. Yes 是
2. No 否

DA019_W2_2 When was your most recent stroke? 最近一次中风的诊断时间是？
____ Year 年 ____Age 岁

PROCEDURE 程序：If respondents have emotional, nervous, or psychiatric problems, then answer DA020 如果受访者患有精神、心理和情感方面的疾病，回答DA020

DA020 Are you now taking any of the folloing treatments for your emotional, nervous, or psychiatric problems?(Check all that apply) Receiving psychiatric or psychological treatment, taking anti depressants, taking tranquilizers or sleeping pills? 您目前有没有正在采用以下方式来治疗精神、心理和情感方面的疾病？（可多选）[IWER: Read one by one. 1 =yes, 2 =no. 访问员注意：请逐项读出以下答案，并让受访者逐一回答，1 = 是，2 = 否。]

1. Receiving psychiatric or psychological treatment 接受精神科治疗或心理治疗
2. Taking anti depressants 服用抗抑郁药物

3. Taking tranquilizers or sleeping pills 服用镇静或安眠药物
4. None of the above 以上都没有

[IWER: Please do not ask proxy the following questions from DA021-DA078. 访员注意：DA021-DA078 题不允许请代理人回答。]

DA021 **PROCEDURE 程序**: If XRType = NEWIW 如果是新的受访者

Have you ever been in a traffic accident or any other kind of major accidental injury and received medical treatment? 您是否经历过交通事故，或任何的重大意外伤害，并接受了治疗？

PROCEDURE 程序: If XRType = REIW 如果是回访受访者

Have you ever been in a traffic accident or any other kind of major accidental injury and received medical treatment [since R's LAST IW MONTH, YEAR/ in the last two years]? 自上次访问以来 (加载上次访问日期), 您是否经历过交通事故，或任何的重大意外伤害，并接受了治疗？

1. Yes 是
2. No 否 → Skip to DA023 跳至 DA023

DA022 Does your injury caused by the accident limit your daily activities? 事故导致的伤害是否影响您现在的日常活动？

1. Yes 是
2. No 否

DA023 Have you fallen down since R's LAST IW MONTH,YEAR? [加载上次访问日期] 自上次访问以来，您有没有摔倒？

1. Yes 是
2. No 没有 → Skip to DA025 跳至 DA025

DA024 How many times have you fallen down seriously enough to need medical treatment? 有多少次摔倒受伤严重到需要接受治疗?____ times 次

DA025 **PROCEDURE 程序**: If XRType = NEWIW 如果是新的受访者

Have you ever fractured your hip? 您有没有过髋骨骨折？

PROCEDURE 程序: If XRType = REIW 如果是回访受访者

PREVIOUS WAVE (ZDA025): [Yes/No/Unknown] 加载上一期受访者的答案 (ZDA025): [是/否/不知道]

1. Yes 是
2. No 没有

F1：“髋骨”指人体腰部的骨骼，共左右两块。幼年时，髋骨分为髂骨，坐骨和耻骨以及软骨连接。成年后，它们之间的软骨会骨化，成为一个整体，即髋骨。

SKIP PATTERN CHECKPOINT: PROSTATE ILLNESS/INCONTINENCE 跳转格式检查: 前列腺疾病/大小便失禁

PROCEDURE 程序: IF R IS MALE, SKIP TO DA029. 男性跳至DA029。

BRANCHPOINT:
IF XRType = REIW REPORTED IN PREVIOUS WAVE THAT HAS NOT STARTED MENOPAUSE , SKIP TO DA027 如果是回访受访者并且没有绝经, 跳至DA027题
IF XRType = REIW REPORTED IN PREVIOUS WAVE THAT HAS STARTED MENOPAUSE, SKIP TO DA032 如果回访受访者已绝经, 跳至DA032题
IF XRType = NEWIW 如果是新的受访者, 询问DA026-DA028 题

DA026 When did you begin the menarche? 您什么时候开始来月经的?
____1900...2011 (**DA026_1**) Year 年 Or 或 Age 年龄 ____1...120 (**DA026_2**) Years 岁
[IWER: Mark the year using four digits. 访员注意: 用 4 位数表示年。]

DA027 Have you started menopause? 您绝经了吗?
1. Yes 是
2. No 没有 → Skip to DA032 跳至 DA032

DA028 When did you begin the menopause? 您什么时候开始绝经的?
____ (**DA028_1**) Year 年 Or 或 Age 年龄 ____ (**DA028_2**) Years 岁 → Skip to DA032 跳至 DA032 [IWER: Mark the year using four digits. 访员注意: 用 4 位数表示年。]

DA029 **PROCEDURE 程序**: If XRType = NEWIW AND IS MALE 如果是新的男性受访者
Have you ever been diagnosed with a prostate illness, such as prostate hyperplasia (excluding prostatic cancer) ? 有没有医生诊断您得了前列腺疾病如前列腺增生 (排除前列腺癌)?
1. Yes 是 Skip to DA030 跳至 DA030
2. No 没有

PROCEDURE 程序: If XRType = REIW AND IS MALE 如果是回访男性受访者
Have you ever been diagnosed with a prostate illness, such as prostate hyperplasia (excluding prostatic cancer) since we talked (in R's LAST IW MONTH,YEAR/in the last two years)? 上一次访问之后 (过去两年), 有没有医生诊断您得了前列腺疾病如前列腺增生 (排除前列腺癌)?
1. Yes 是 → Skip to Skip to DA030 跳至 DA030
2. No 没有

F1: "前列腺增生" 主要症状有排尿困难, 轻者夜里起床小便次数增多, 有尿不净或尿完后还有少量排出的现象; 严重者出现尿流变细, 甚或排不出的现象; 同时常伴有腰酸腰痛、四肢无力、遗精等症状。

DA029_W2_1 Do you know if you had a prostate illness, such as prostate hyperplasia (excluding prostatic cancer) ? 您是否知道自己患有前列腺疾病如前列腺增生 (排除前列腺癌)?

1. Yes 知道自己患有
2. No 知道自己没有 → Skip to DA032 跳至 DA032
3. Don't know 不知道有没有 → Skip to DA032 跳至 DA032

DA030 When was the condition first diagnosed? 第一次诊断出您有前列腺疾病是在什么时候?
____(**DA030_1**)Year 年 Or 或 Age 年龄 ____ (**DA030_2**) Years 岁 [IWER: Mark the year using four digits. 访员注意: 用 4 位数表示年。]

DA031 Are you now taking medication or other treatment for your prostate illness? 您目前是否正在服药或以其他方式治疗前列腺疾病?

1. Yes 是
2. No 否

DA032 Now I have some questions about your eyesight. Do you usually wear glasses or corrective lenses? 下面问您一些关于视力的问题。您通常是否戴眼镜（包括矫正视力镜片)?

1. Yes 是
2. Legally blind 失明 → Skip to DA038 跳至 DA038
3. No 否
4. Sometimes 偶尔

DA033 How good is your eyesight for seeing things at a distance, like recognizing a friend from across the street (with glasses or corrective lenses if you wear them)? Would you say your eyesight for seeing things at a distance is excellent, very good, good, fair, or poor? 您看远处的东西怎么样? 比如说能不能隔着马路认出朋友 (包括戴着眼镜)。是极好，很好，好，一般还是不好?

1. Excellent 极好
2. Very good 很好
3. Good 好
4. Fair 一般
5. Poor 不好

DA034 How good is your eyesight for seeing things up close, like reading ordinary newspaper print (with glasses or corrective lenses if you wear them)? Would you say your eyesight for seeing things up close is excellent, very good, good, fair, or poor? 您看近处的东西怎么样? 比如说戴着眼镜能不能看报纸? 是极好，很好，好，一般还是不好?

1. Excellent 极好
2. Very good 很好
3. Good 好
4. Fair 一般
5. Poor 不好

**PROCEDURE 程序**: If XRType = NEWIW: 如果是新的受访者:

DA035 Have you ever had cataract surgery? 您是否做过白内障手术?

1. Yes 是
2. No 否 → Skip to DA037 跳至 DA037

**PROCEDURE 程序**: If XRType = REIW AND R REPORTED IN LAST IW THAT HAD CATARACT SURGERY ON ONE EYE(ZDA036 = 1): 如果是回访受访者，上期访问回答一只眼睛做过白内障手术 (ZDA036 = 1):

[IWER: PREVIOUSLY REPORTED CATARACT SURGERY ON ONE EYE 访员注意：上期访问受访者报告一只眼睛做过白内障手术]

DA035_W2_1 Have you had another cataract surgery since we last talked to you (in R's LAST IW MONTH, YEAR) other than what you told us about then? 上次访问记录显示您一只眼睛做过白内障，(加载上次访问日期）之后（过去两年）您的另一只眼睛是否做过白内障手术?

1. Yes 是
2. No 否

**PROCEDURE 程序**: If XRType = REIW AND R DID NOT REPORT IN LAST IW THAT HAD CATARACT SURGERY ((ZDA035 ! = 1) : 如果是回访受访者，上期访问回答没有做过白内障手术:

DA035_W2_2 Have you had cataract surgery (since R's LAST IW MONTH, YEAR/in the last two years)? (加载上次访问日期）自上次访问以来，您是否做过白内障手术?

1. Yes 是
2. No 否

DA036 Have you had cataract surgery on both eyes or just one? 白内障手术是做的一只眼还是两只眼?

1. One eye only 一只眼
2. Both eyes 两只眼

**PROCEDURE 程序**: If XRType = NEWIW OR XRType = REIW AND R DID NOT REPORT IN LAST IW THAT HAD GLAUCOMA(ZDA037 = 2): 如果是新的受访者或者是上期回答没有得过青光眼的回访受访者 (ZDA037 ! = 1):

DA037 Has a doctor/nurse/paramedical/ doctor of traditional Chinese medicine doctor ever treated you for glaucoma? 是否有医生诊断过您得了青光眼?

1. Yes 是
2. No 否

**PROCEDURE 程序**: If XRType = REIW AND R REPORTED IN LAST IW THAT HAD GLAUCOMA(ZDA037 = 1): 如果是上期回答得过青光眼的回访受访者 (ZDA037 = 1):

DA037_W2 You told us you had glaucoma, has a doctor/nurse/paramedical/ doctor of traditional Chinese medicine doctor ever treated you for glaucoma relapses since last iw time? [加载基线时间] 上一次的访问记录显示您患有青光眼，之后您的青光眼是否复发？

1. Yes 是
2. No 否
3. Never had glaucoma 一直没有青光眼

**PROCEDURE 程序**：IF R REPORTED IN PREVIOUS IW THAT WEARS HEARING AID (ZDA038 = 1), SKIP TO DA039 如果是回访受访者，上期访问回答戴助听器，跳至DA039

DA038 Now I have some questions about your hearing. Do you ever wear a hearing aid? 现在有几个关于听力的问题。您有没有戴过助听器？

1. Yes 是
2. No 否

DA039 Is your hearing very good, good, fair, poor, or very poor (with a hearing aid if you normally use it and without if you normally don’t)? Would you say your hearing is excellent, very good, good, fair, or poor? 您的听力如何？(如果您经常戴助听器，那么戴助听器时听力如何？如果您不经常戴助听器，那么不戴助听器时听力如何？) 是极好，很好，好，一般还是不好？

1. Excellent 极好
2. Very good 很好
3. Good 好
4. Fair 一般
5. Poor 不好

**PROCEDURE 程序**：If XRType = NEWIW or XRType = REIW that did not lost all teeth in last iw(ZDA040 $\neq 1$), ask DA040: 如果是新的受访者或上期回答牙齿未掉光的回访受访者，询问DA040：

DA040 Have you lost all of your teeth? 您的牙齿是否已经掉光？

1. Yes 是
2. No 否

[Now, please pause briefly to think about YESTERDAY, from the morning until the end of the day. Think about where you were, what you were doing, who you were with, and how you felt. 现在请您简单回忆一下昨天一整天的生活，回忆一下您在何处，做什么事，和谁在一起，以及感觉如何。]

WB01 What day-of-the-week was it yesterday? (Mark (X) one box.) 昨天是星期几？

1. Right 对
2. Wrong 错
3. Don’t know 不知道

WB02 What was the date yesterday? (Month / day) 昨天是几月几号？

1. Right 对
2. Wrong 错
3. Don't know 不知道

WB03 What time did you wake up yesterday? (Hour, Minute, AM/PM, 12hours system) 昨天您是几点起床的？（12 小时制）
上午 ____ 点 ____ 分
或下午 ____ 点 ____ 分

WB04 What time did you go to sleep at the end of the day yesterday? (Hour, Minute, AM/PM, 12hours system) 昨天您几点睡觉的？（12 小时制）
上午 ____ 点 ____ 分
或下午 ____ 点 ____ 分

[The next questions are about your experiences yesterday. Mark (X) in one box for each line for the extent you felt the following. 现在请您花点时间回想一下昨天，从早晨一直到晚上，想想您去了哪里，干了什么，跟谁在一起，以及心情如何。想好了吗？那我问您：]
[Show Card 出示卡片 10]

WB05 Yesterday, did you feel Frustrated 昨天您是否曾经有不顺心的感觉？
1. Not at all 完全没有
2. A little 有一点
3. Somewhat 有一些
4. Quite a bit 比较多
5. Very 非常多

[Show Card 出示卡片 10]

WB06 Yesterday, did you feel Sad? 昨天您是否曾经有悲伤的感觉？
1. Not at all 完全没有
2. A little 有一点
3. Somewhat 有一些
4. Quite a bit 比较多
5. Very 非常多

[Show Card 出示卡片 10]

WB07 Yesterday, did you feel Enthusiastic? 昨天您是否曾经有精神振奋的感觉？
1. Not at all 完全没有
2. A little 有一点
3. Somewhat 有一些
4. Quite a bit 比较多
5. Very 非常多

[Show Card 出示卡片 10]

WB08 Yesterday, did you feel Lonely? 昨天您是否曾经有孤独的感觉?

1. Not at all 完全没有
2. A little 有一点
3. Somewhat 有一些
4. Quite a bit 比较多
5. Very 非常多

[Show Card 出示卡片 10]

WB09 Yesterday, did you feel Content? 昨天您是否曾经有心满意足的感觉?

1. Not at all 完全没有
2. A little 有一点
3. Somewhat 有一些
4. Quite a bit 比较多
5. Very 非常多

[Show Card 出示卡片 10]

WB10 Yesterday, did you feel Worried? 昨天您是否曾经有忧虑的感觉?

1. Not at all 完全没有
2. A little 有一点
3. Somewhat 有一些
4. Quite a bit 比较多
5. Very 非常多

[Show Card 出示卡片 10]

WB11 Yesterday, did you feel Bored? 昨天您是否曾经有无聊的感觉?

1. Not at all 完全没有
2. A little 有一点
3. Somewhat 有一些
4. Quite a bit 比较多
5. Very 非常多

[Show Card 出示卡片 10]

WB12 Yesterday, did you feel Happy? 昨天您是否曾经有愉快的感觉?

1. Not at all 完全没有
2. A little 有一点
3. Somewhat 有一些
4. Quite a bit 比较多
5. Very 非常多

[Show Card 出示卡片 10]

WB13 Yesterday, did you feel Angry? 昨天您是否曾经有愤怒的感觉?

1. Not at all 完全没有
2. A little 有一点
3. Somewhat 有一些
4. Quite a bit 比较多
5. Very 非常多

[Show Card 出示卡片 10]

WB14 Yesterday, did you feel Tired? 昨天您是否曾经有疲惫无力的感觉?

1. Not at all 完全没有
2. A little 有一点
3. Somewhat 有一些
4. Quite a bit 比较多
5. Very 非常多

[Show Card 出示卡片 10]

WB15 Yesterday, did you feel Stressed? 昨天您是否曾经有精神紧张的感觉?

1. Not at all 完全没有
2. A little 有一点
3. Somewhat 有一些
4. Quite a bit 比较多
5. Very 非常多

WB16 Yesterday, did you feel any pain? 昨天，您感到疼痛吗?

1. None 完全不 → Skip DA042 and DA042_W2_1 跳过 DA042和 DA042_W2_1
2. A little 有一点
3. Some 有一些
4. Quite a bit 比较严重
5. A lot 非常严重

[Show Card 出示卡片 9]

DA042 On what part of your body do you feel pain? Please list all parts of body you are currently feeling pain. 身体哪些部位感到疼痛? 请列出所有部位。

1. Head (Headache) 头
2. Shoulder 肩膀
3. Arm 胳膊
4. Wrist 手腕
5. Fingers 手指

6. Chest 胸
7. Stomach (Stomachache) 胃
8. Back 背
9. Waist 腰
10. Buttocks 臀部
11. Leg 腿
12. Knees 膝盖
13. Ankle 脚踝
14. Toes 脚趾
15. Neck 脖子

DA042_W2_1 Are you taking measures to reduce the pain? 您是否采取以下措施以减轻疼痛？(可多选)

1. Taking Chinese traditional medicine 服用中药
2. Taking Western modern medicine 服用西药
3. Acupuncture treatment 针灸疗法
4. Professional massage therapy 推拿按摩
5. Other 其他
6. None 以上都没有

DA045 Are there any other medical diseases or conditions that are important to your health now that we have not talked about? 是否还有其他严重的疾病或身体不适我没问到？

1. Yes 是 → Skip to DA046 跳至DA046
2. No 否 → Skip to DA047 跳至DA047

DA046 What illness is that? 这些疾病是什么？ ____

DA047 Have you gained or lost 5 or more kilograms in the last year? (excluding pregnancy) 过去一年有没有增重 10 斤以上，或减轻 10 斤以上？(怀孕除外)

1. Yes, I only gained weight 是，只增重。
2. Yes, I only lost weight 是，只减轻。
3. Yes, I first gained and then lost weight 是，先增重再减轻。
4. Yes, I first lost and then gained weight 是，先减轻再增重。
5. No 没有
6. I don't know 我不知道

**PROCEDURE 程序**：If XRType = NEWIW(ZDA048 = null): 如果是新的受访者：

DA048 How would you evaluate your health during childhood, up to and including age 15? Excellent, very good, good,fair, poor? 您 15 岁之前（包括 15 岁）的身体状况怎么样？极好，很好，好，一般，不好？

1. Excellent 极好
2. Very Good 很好

3. Good 好
4. Fair 一般
5. Poor 不好

PART II: LIFESTYLE AND HEALTH BEHAVIORS 第二部分：生活方式和健康行为

DA049 During the past month, how many hours of actual sleep did you get at night (average hours for one night)? (This may be shorter than the number of hours you spend in bed.) 过去一个月内，您平均每天晚上真正睡着的时间大约是几小时？（可能短于您在床上躺着的时间）
____0...24 hours 小时

DA050 During the past month, how long did you take a nap after lunch? 过去一个月内，您通常午睡多长时间？____minutes 分钟 [IWER：If R didn't take a nap, please record for 0. 访员注意：如果受访者不午睡，请记录为 0。]

**PROCEDURE 程序**：DA051 will be presented ONLY to a random subsample of households (half). Main respondent and spouse in the selected households should answer DA051 仅仅在二次抽样被选中的家庭中询问DA051题。选中家庭中的主要受访者及其配偶都要回答DA051 题。

DA051 Now we would like to ask about the amount of time you spend on different types of physical activities in a usual week. 下面有一些问题是有关您通常每周花了多少时间做这些活动？

DA051_1 What's the purpose for doing these physical activities, for entertainment, job demand or exercise in doing these physical activities? 是因为工作需要、娱乐活动、体育锻炼还是其他？

1. Job demands 工作需要
2. Entertainments 娱乐
3. Exercise 体育锻炼
4. Other 其他

[ACTIVITIES IN LAST MONTH 过去一个月的活动]
[Show Card 出示卡片 11]

DA056 Have you done any of these activities in the last month? (Code all that apply) 您过去一个月是否进行了下列社交活动？(可多选)

1. Interacted with friends 串门、跟朋友交往
2. Played Ma-jong, played chess, played cards, or went to community club 打麻将、下棋、打牌、去社区活动室
3. Provided help to family, friends, or neighbors who do not live with you and who did not pay you for the help 无偿向与您不住在一起的亲人、朋友或者邻居提供帮助
4. Went to a sport, social, or other kind of club 去公园或者其他场所跳舞、健身、练气功等

PHYSICAL ACTIVITIES 身体活动 (KKTYPE)	**DA051** During a usual week, did you do any [...] for at least 10 minutes continuously? 您通常每周有没有至少持续做[...]十分钟？	**DA052** During a usual week, on how many days did you do [...] for at least 10 minutes? 您通常每周有多少天做 [...] 至少十分钟？	How much time did you usually spend doing [...] on one of those days? 在做 [...] 的这些天里，您一天花多少时间做 [...]?	
A. Now, think about all the vigorous activities requiring hard/high-intensity physical effort that you do in a usual week. Vigorous activities make you breathe much harder than normal and may include heavy lifting, digging, plowing, aerobics, fast bicycling, and cycling with a heavy load. Think only about those physical activities that you did for at least 10 minutes at a time. 下面请回忆下您通常每周做的非常消耗体力的激烈活动。激烈的活动会让你呼吸急促，比如搬运重物、挖地、耕作、有氧运动、快速骑车、骑车载货等。只需回忆您每次运动了至少十分钟的活动。	2 No 否 ↓<br>1 Yes 是 →	___ 1...7 days 天	**DA053**<br>1. $<$ 2 hours 小时<br>2. $\geq$ 2 hours 小时	**DA054**<br>1. $<$ 30 minutes 分钟<br>2. $\geq$ 30 minutes 分钟<br>**DA055**<br>3. $<$ 4 hours 小时<br>4. $\geq$ 4 hours 小时
B. Now think about activities which take moderate physical effort that you do in a usual week. Moderate physical activities make you breathe somewhat harder than normal and may include carrying light loads, bicycling at a regular pace, or mopping the floor. Again, think about only those physical activities that you did for at least 10 minutes at a time. 下面请回忆下您通常每周做的中等强度的体力活动。中等体力的活动让您的呼吸比平时快一些，比如搬运轻便的东西、常规速度骑自行车、拖地、打太极拳、疾走。只要回忆您每次持续了至少十分钟的活动。	2 No 否 ↓<br>1 Yes 是 →	___ 1...7 days 天	**DA053**<br>1. $<$ 2 hours 小时<br>2. $\geq$ 2 hours 小时	**DA054**<br>1. $<$ 30 minutes 分钟<br>2. $\geq$ 30 minutes 分钟<br>**DA055**<br>3. $<$ 4 hours 小时<br>4. $\geq$ 4 hours 小时
C. Now think about the time you spend walking in a usual week. This includes at work and at home, walking to travel from place to place, and any other walking that you might do solely for recreation, sport, exercise, or leisure. 下面回忆一下您通常每周走路花了多少时间。走路包括工作或者在家的时候从一个地方走到另一个地方，以及其他您为了休闲、运动、锻炼或娱乐而散步。	2 No 否 ↓<br>1 Yes 是 →	___ 1...7 days 天	**DA053**<br>1. $<$ 2 hours 小时<br>2. $\geq$ 2 hours 小时	**DA054**<br>1. $<$ 30 minutes 分钟<br>2. $\geq$ 30 minutes 分钟<br>**DA055**<br>3. $<$ 4 hours 小时<br>4. $\geq$ 4 hours 小时

5. Took part in a community-related organization 参加社团组织活动
6. Done voluntary or charity work 志愿者活动或者慈善活动
7. Cared for a sick or disabled adult who does not live with you and who did not pay you for the help 无偿照顾与您不住在一起的病人或残疾人
8. Attended an educational or training course 上学或者参加培训课程
9. Stock investment 炒股（基金及其他金融证券）
10. Used the Internet 上网
11. Other 其他社交活动
12. None of these 以上均没有

[CHECK: You cannot select “None of these” together with any other answer. Please change your answer. 检查点：您不能同时选择“以上答案均不是”和其他选项。请重新选择。]

PROCEDURE 程序：IF cnt IN DA056 (ACTIVITIES IN LAST MONTH) 如果 DA056题答案为1-11（过去一个月的活动）

DA057 Frequency of activity in the last month 过去一个月的活动频率

How often in the last month [did/have][you] [do voluntary or charity work/cared for a sick or disabled adult/provided help to family, friends or neighbors/attended an educational or training course/ Interacted with friends /go to a sport,social or other kind of club/taken part in a community-related organization]? Almost daily, almost every week, or not regularly? 过去一个月，您每隔多长时间会做刚才说的这些活动（志愿者活动或者慈善活动、照顾病人或残疾人、给家里人、朋友或者邻居提供帮助、接受教育或者参加培训课程、与朋友来往、去运动娱乐、社交或者其他类型的俱乐部、参加社团组织活动、炒股、上网）？差不多每天，差不多每周或不经常？

1. Almost daily 差不多每天
2. Almost every week 差不多每周
3. Not regularly 不经常

DA058 How many meals do you normally eat every day? More than 4 meals per day,4 meals per day,3 meals per day ,2 meals per day,1 meal per day or <1 meal per day? 您一般每天吃几顿饭？每天多于 4 顿，每天 4 顿，每天 3 顿，每天 2 顿，每天一顿，每天少于一顿?

[IWER:Read each choice of the answers. 访员注意：请读出各选项。]

1. More than 4 meals per day 每天多于 4 顿
2. 4 meals per day 每天 4 顿
3. 3 meals per day 每天 3 顿
4. 2 meals per day 每天 2 顿
5. 1 meal per day 每天一顿
6. <1 meal per day 每天少于一顿

[INTRO: Next, I would like to ask whether you have had the habit of smoking cigarettes/smoking a pipe/chewing tobacco, now or in the past. By smoking we mean smoking more than 100 cigarettes in your life) 下面我想知道您现在或者过去是否有抽烟、用烟管吸烟或咀嚼烟草的习惯（吸烟指的是一生吸烟 100 支以上)？]

BRANCHPOINT:

IF XRType = REIW AND R REPORTED EVER SMOKED(ZDA050 = 1), SKIP TO DA061. 如果回访受访者吸过烟，跳至DA061。

IF XRType = REIW AND R DID NOT REPORT EVER SMOKED(ZDA050 not 1); OR XRType = NEWIW, ASK DA059: 如果是从未吸过烟的回访受访者或新的受访者，询问 hyperref[DA059]DA059：

DA059 Have you ever chewed tobacco, smoked a pipe, smoked self-rolled cigarettes, or smoked cigarettes/cigars? 您吸过烟吗？(包括香烟、旱烟、用烟管吸烟或咀嚼烟草)

1. Yes 是
2. No 否 Skip to DA067 请跳至DA067

DA061 PROCEDURE 程序: If XRType = REIW 如果是回访受访者

Our records from your last interview in R's LAST IW MONTH, YEAR show that you have ever smoked, 上一次的访问记录显示您曾经吸烟,
Do you still have the habit or have you totally quit? 您现在还在吸烟还是戒烟了?

PROCEDURE 程序: If XRType = NEWIW 如果是新受访者
Do you still have the habit or have you totally quit? 您现在还在吸烟还是戒烟了?

1. Still have 仍然抽烟 Skip DA062 请跳过DA062
2. Quit 戒烟

DA062 At what age did you totally quit smoking? 您这次成功戒烟是在多少岁或那一年? Age 年龄 ____1...120 (**DA062_1**) years 岁 Or 或 ____1900...2011 (**DA062_2**) Year 年 [IWER: Mark the year using four digits. 访员注意: 用 4 位数表示年。]

DA063 In one day about how many cigarettes do/did you consume [preload:now/before totally quitting]? 您 [系统加载: 现在/戒烟前] 平均一天抽多少支香烟? ____cigarettes 支

DA064 How much does/did it cost per pack = 20 cigarettes? 您 [系统加载: 现在/戒烟前] 前, 抽的香烟多少钱一包 (一包 20 支) ? ____Yuan 元 [IWER: Prompt R: we' re asking price at that time, not current price 访员注意: 提示受访者, 此处询问的是当时价格, 非现在的价格]

PROCEDURE 程序: If XRType = REIW THAT HAS BEEN ASKED WHEN STARTED SMOKING(ZDA065 not null), SKIP DA065. 如果回访受访者之前被问到过开始吸烟的年龄, 跳过DA065题

DA065 At what age did you start to smoke on a regular basis? 您是多少岁开始经常抽烟的? Age 年龄 ____ 1...120 (**DA065_1**) years 岁 Or 或 ____ 1900...2011 (**DA065_2**) Year 年 [IWER: Mark the year using four digits. 访员注意: 用 4 位数表示年。]

DA066 How soon after you wake up did/do you smoke your first cigarette, cigar, or pipe? 您 [系统加载: 现在/戒烟前] 前一般睡醒后多久就开始抽烟?

1. Within 5 minutes 5 分钟之内
2. Within 6-30 minutes 6-30 分钟之内
3. Within 31-60 minutes 31-60 分钟之内
4. More than 1 hour 1 个小时以后

DA067 Did you drink any alcoholic beverages, such as beer, wine, or liquor in the past year? How often? 在过去的一年, 您喝酒吗, 包括啤酒、葡萄酒或白酒等? 喝酒频率如何?

1. Drink more than once a month. 喝酒, 每月超过一次
2. Drink but less than once a month 喝酒, 但每月少于一次 Skip to DA069 请跳至DA069
3. None of these 什么都不喝 Skip to DA069 请跳至DA069

DA068 What type of alcoholic beverages did you drink? Liquor, wine, or beer? (code all that apply) 您平时喝哪种酒? 烈性酒、葡萄酒、啤酒还是其他? (可多选)

1. Liqor, including white liquor, whisky, and others 烈性酒，包括白酒、威士忌和其他酒 Skip to DA071 请跳至DA071
2. Beer 啤酒 Skip to DA071 请跳至DA071
3. Wine or rice wine 葡萄酒、米酒或黄酒 Skip to DA071 请跳至DA071

**PROCEDURE 程序**：If XRType = REIW THAT HAS BEEN ASKED WHETHER DRINKING ALCOHOLIC BEVERAGES IN THE PAST, SKIP DA069. 如果回访受访者之前被问过以前是否喝酒，跳过DA069 题

DA069 Did you ever drink alcoholic beverages in the past? How often? 您以前喝酒吗？喝酒频率如何？

1. I never had a drink. 我从不或极少喝酒 →Skip to procedure before DA079 请跳至DA079 前面的程序控制
2. I used to drink less than once a month. 我很少喝酒，每月少于一次 →Skip to procedure before DA079 请跳至DA079 前面的程序控制
3. I used to drink more than once a month. 我以前喝酒，每月超过一次

**PROCEDURE 程序**：If XRType = REIW THAT HAS BEEN ASKED WHEN QUITING ALCOHOLIC BEVERAGES IN THE PAST, SKIP DA070. 如果回访受访者之前被问过何时戒酒的，跳过DA070题

DA070 When did you quit or reduce drinking? 请问您什么时候戒酒或减少喝酒次数的？

____1900...2011 (**DA070_1**)Year 年 Or age 或年龄： ____1...120 (**DA070_2**) Years 岁

[IWER: Record year in 4 digits. 访员注意：年请用 4 位数表示]

**PROCEDURE 程序**：If XRType = REIW THAT HAS BEEN ASKED WHEN STARTED DRINKING, SKIP DA071. 如果回访受访者之前被问到过开始饮酒的年龄，跳过DA071 题

DA071 When did you start drinking? 请问您什么时候开始饮酒??

____1900...2011 (**DA071_1**)Year 年 Or age 或年龄： ____1...120 (**DA071_2**) Years 岁

[IWER: Record year in 4 digits. 访员注意：年请用 4 位数表示]

SKIP PATTERN CHECKPOINT: DRINKING 跳转格式检查：喝酒

[INTRO: Now, I am going to ask you how often and how much you drank during the past year. Please tell me how often you drank per month, and how much you drank at a time on average. I will repeat the questions for different types of alcoholic beverages. 现在我想了解过去一年您喝酒的情况。请回答您平均每个月喝多少次酒，一次大概喝多少。我会针对不同的酒类，分别提问。]

**CAPI 程序**：If DA067 answered 1, ask DA072: 如果DA067 选择了 1，询问DA072。

DA072 How often did you drink liquor, including white liquor, whisky, and others per month in the last year? 过去一年内您平均一个月喝几次烈性酒，包括白酒、威士忌？

1. Once a month 每月一次

2. 2-3 times a month 每月 2-3 次
3. Once a week 每周一次
4. 2-3 times a week 每周 2-3 次
5. 4-6 times a week 每周 4-6 次
6. Once a day 每天一次
7. Twice a day 一天两次
8. More than twice a day 一天超过两次

DA073 The last time you drank liquor last year, how many liang of liquor did you drink? (1 liang = 50cc/50ml) 请问您过去一年内最近一次喝烈性酒，喝了多少两？（1 两 = 50 毫升）
____liang 两

**CAPI 程序**：If DA068 = 2, ask DA074. 如果DA068 选择了 2，询问DA074。]

DA074 How many times per month did you drink beer in the last year? 过去一年内您平均一个月喝几次啤酒?

1. Once a month 每月一次
2. 2-3 times a month 每月 2-3 次
3. Once a week 每周一次
4. 2-3 times a week 每周 2-3 次
5. 4-6 times a week 每周 4-6 次
6. Once a day 每天一次
7. Twice a day 一天两次
8. More than twice a day 一天超过两次

DA075 The last time you drank beer last year, how many bottles of beer did you drink? (1bottle= 2.5 mugs，1mug= 220cc) 请问您过去一年内最近一次喝啤酒，喝了多少瓶？（1 瓶 =2.5 杯，1 杯 =220 毫升） ____ **(DA075_1**)0...120bottles 瓶 or 或者 ____**(DA075_2**)0...300mugs 杯

**CAPI 程序**：If DA068 = 3, ask DA076. 如果DA068 选择了 3，询问DA076。]

DA076 How often did you drink wine or rice wine per month in the last year? 过去一年内您平均一个月喝几次葡萄酒、米酒或黄酒？

1. Once a month 每月一次
2. 2-3 times a month 每月 2-3 次
3. Once a week 每周一次
4. 2-3 times a week 每周 2-3 次
5. 4-6 times a week 每周 4-6 次
6. Once a day 每天一次
7. Twice a day 一天两次
8. More than twice a day 一天超过两次

DA077 The last time you drank it last year, how many liang of wine did you drink? (1 liang=50cc) 请问您过去一年内最近一次喝葡萄酒、米酒或黄酒，喝了多少两？（1 两 =50 毫升） ____0.00...100.00liang 两

DA078 In the last year, have you ever taken a drink first thing in the morning to steady your nerves or get rid of a hangover? 过去一年内，请问您是否曾经在早晨做的第一件事就是喝酒，以平缓紧张情绪或为了摆脱宿醉的烦恼？

1. Yes 是
2. No 否

[IWER: Please do not ask proxy the following question DA079 and DA080. 访员注意：DA079 and DA080 题不允许请代理人回答。]

DA079 How would you rate your health status? Would you say your health is very good, good, fair, poor or very poor? 您觉得您的健康状况是很好，好，一般，不好，还是很不好？

1. Very good 很好
2. Good 好
3. Fair 一般
4. Poor 不好
5. Very poor 很不好

DA080 Next I have some questions about your health. Would you say your health is excellent, very good, good, fair, or poor? 您觉得您自己的健康状况怎么样？是极好，很好，好，一般还是不好？[IWER：interviewer should read all the following options. 访员注意：请读出所有的选项。]

1. Excellent 极好
2. Very good 很好
3. Good 好
4. Fair 一般
5. Poor 不好

DB FUNCTIONAL LIMITATIONS AND HELPERS 身体功能障碍以及辅助者

CAPI 程序：If R is younger than 50(year of birth is after 1963) and If DA001 = 1 or 2 or DA002 = 1 or 2 and DA003 = 2 and DA004 = 2 and DA005 = 2 and DA007 = 2 and DA008 = 2 skip DB001- DB015. 如果受访者年龄小于 50 岁 (出生年份在 1963 年之后)，并且自我评价健康状况良好、没有残疾、慢性病和身体疼痛，跳过 DB001-DB015。]

[We need to understand difficulties people may have with various activities because of a health or physical problem. Please tell me whether you have difficulty performing any of the

**INTERVIEWER CHECK AGE OF RESPONDENT? 访员请检查受访者的年龄。**	**1.** < 65 **YEAR 岁 →COLUMN A A 栏**<br>**2.** 65 – 69 **YEAR 岁 →COLUMN B B 栏**<br>**3.** 70 – 74 **YEAR 岁 →COLUMN C C 栏**<br>**4.** 75 – 79 **YEAR 岁 →COLUMN D D 栏**<br>**5.** 80 – 84 **YEAR 岁 →COLUMN E E 栏**				**6.** 85 – 89 **YEAR 岁 →COLUMN F F 栏**<br>**7.** 90 – 94 **YEAR 岁 →COLUMN G G 栏**<br>**8.** 95 – 99 **YEAR 岁 →COLUMN H H 栏**<br>**9.** ≥ 100 **YEAR 岁 →COLUMN I I 栏**				
	A	B	C	D	E	F	G	H	I
AGE 年龄	75 years 岁	80 years 岁	85 years 岁	90 years 岁	95 years 岁	100 years 岁	105 years 岁	110 years 岁	115 years 岁
**DA081** Suppose there are 5 steps, where the lowest step represents the smallest chance and the highest step represents the highest chance, on what step do you think is your chance in reaching the age of [...]? 假定有五个级别，最低一级代表可能性最小，最高一级代表可能性最大，您设想您活到这个年龄的可能性有多大。1 Almost impossible 几乎不可能 2 Not very likely 不太可能 3 Maybe 有可能 4 Very likely 很可能 5 Almost certain 简直一定	1<br>2<br>3<br>4<br>5	1<br>2<br>3<br>4<br>5	1<br>2<br>3<br>4<br>5	1<br>2<br>3<br>4<br>5	1<br>2<br>3<br>4<br>5	1<br>2<br>3<br>4<br>5	1<br>2<br>3<br>4<br>5	1<br>2<br>3<br>4<br>5	1<br>2<br>3<br>4<br>5

following tasks on a regular basis. Exclude any difficulties that you expect to last less than three months. 人们可能会由于健康或身体的原因而在多种活动中有困难。请说说您每天做事的时候是否遇到了这些困难（排除那些你预计在三个月内能解决的问题）. 每道题目的选项都是一样的，包括没有困难，有困难但仍可以完成，有困难且需要帮助，无法完成。请选择合适的答案。]

DB001 Do you have any difficulty with running or jogging about 1 Km? 您现在跑或慢跑 1 公里，有困难吗？

1. No, I don’t have any difficulty 没有困难 → Skip to DB004 跳至DB004
2. I have difficulty but can still do it. 有困难但仍可以完成。
3. Yes, I have difficulty and need help. 有困难，需要帮助。
4. I can not do it. 无法完成

DB002 Do you have difficulty ...Walking 1 km...? 您现在走 1 公里，有困难吗？

1. No, I don’t have any difficulty 没有困难 → Skip DB003 跳过DB003
2. I have difficulty but can still do it. 有困难但仍可以完成。
3. Yes, I have difficulty and need help. 有困难，需要帮助。
4. I can not do it. 无法完成

DB003 Do you have difficulty ...Walking 100 metres...? 您走 100 米，有困难吗？

1. No, I don’t have any difficulty 没有困难
2. I have difficulty but can still do it. 有困难但仍可以完成。
3. Yes, I have difficulty and need help. 有困难，需要帮助。
4. I can not do it. 无法完成

DB004 Do you have difficulty ...Getting up from a chair after sitting for a long period...? 您在椅子上坐时间久了再站起来，有困难吗？

1. No, I don’t have any difficulty 没有困难
2. I have difficulty but can still do it. 有困难但仍可以完成。
3. Yes, I have difficulty and need help. 有困难，需要帮助。
4. I can not do it. 无法完成

DB005 Do you have difficulty ...Climbing several flights of stairs without resting...? 您连续不停地爬几层楼，有困难吗？

1. No, I don't have any difficulty 没有困难
2. I have difficulty but can still do it. 有困难但仍可以完成。
3. Yes, I have difficulty and need help. 有困难，需要帮助。
4. I can not do it. 无法完成

DB006 Do you have difficulty ...Stooping, kneeling, or crouching...? 弯腰、屈膝或者下蹲，您有困难吗？

1. No, I don't have any difficulty 没有困难
2. I have difficulty but can still do it. 有困难但仍可以完成。
3. Yes, I have difficulty and need help. 有困难，需要帮助。
4. I can not do it. 无法完成

DB007 Do you have difficulty ...Reaching or extending your arms above shoulder level...? (he/she is regarded as not having difficulty only if he/she can extend both of his/her arms, otherwise he/she is regarded as having difficulty.) 您把手臂沿着肩向上伸展，有困难吗？（两个手都没困难才算没困难，否则算有困难）

1. No, I don't have any difficulty 没有困难
2. I have difficulty but can still do it. 有困难但仍可以完成。
3. Yes, I have difficulty and need help. 有困难，需要帮助。
4. I can not do it. 无法完成

DB008 Do you have difficulty ...Lifting or carrying weights over 10 jin, like a heavy bag of groceries...? 您提 10 斤重的东西，有困难吗？(注意是市斤)

1. No, I don't have any difficulty 没有困难
2. I have difficulty but can still do it. 有困难但仍可以完成。
3. Yes, I have difficulty and need help. 有困难，需要帮助。
4. I can not do it. 无法完成

DB009 Do you have difficulty ...Picking up a small coin from a table...? 您从桌上拿起一小枚硬币，有困难吗？

1. No, I don't have any difficulty 没有困难
2. I have difficulty but can still do it. 有困难但仍可以完成。
3. Yes, I have difficulty and need help. 有困难，需要帮助。
4. I can not do it. 无法完成

CAPI 程序：IF (DB001 = 1 & DB003 = 1 . . . DB009 = 1), THEN SKIP TO DB016, 如果受访者在DB001～ DB009 没有任何困难，请跳至DB016

Here are a few more everyday acivities. Please tell me if you have any difficulties with these because of a physical, mental, emotional or memory problem. Again, exclude any that you expect to last less than three months. 下面我们想了解一下您日常生活的情况。请问您目前是否因为身体、精神、情感或者记忆方面的原因导致完成下面我们提到的一些日常行为有困难。我们指的“困难”不包括那些预计三个月内能够解决的困难。

DB010 Because of health and memory problems, do you have any difficulty with dressing? Dressing includes taking clothes out from a closet, putting them on, buttoning up, and fastening a belt. 请问您是否因为健康和记忆的原因，自己穿衣服有困难？穿衣服包括从衣橱中拿出衣服，穿上衣服，扣上钮扣，系上腰带。

1. No, I don't have any difficulty 没有困难 → Skip to DB011 跳至DB011
2. I have difficulty but can still do it. 有困难但仍可以完成。
3. Yes, I have difficulty and need help. 有困难，需要帮助。
4. I can not do it. 无法完成

DB010_W2 Does anyone ever help you dress? 穿衣服的时候是否有人帮助你？

1. Yes 有
2. No 没有

DB011 Because of health and memory problems, do you have any difficulty with bathing or showering? 请问您是否因为健康和记忆的原因，洗澡有困难？

1. No, I don't have any difficulty 没有困难 → Skip to DB012 跳至DB012
2. I have difficulty but can still do it. 有困难但仍可以完成。
3. Yes, I have difficulty and need help. 有困难，需要帮助。
4. I can not do it. 无法完成

DB011_W2 Does anyone ever help you bathe? 洗澡的时候是否有人帮助你？

1. Yes 有
2. No 没有

DB012 Because of health and memory problems, do you have any difficulty with eating, such as cutting up your food? 请问您是否因为健康和记忆的原因，自己吃饭有困难，比如自己夹菜？(Definition: By eating, we mean eating food by oneself when it is ready. 定义：当饭菜准备好以后，自己吃饭定义为用餐。)

1. No, I don't have any difficulty 没有困难 → Skip to DB013 跳至DB013
2. I have difficulty but can still do it. 有困难但仍可以完成。
3. Yes, I have difficulty and need help. 有困难，需要帮助。
4. I can not do it. 无法完成

DB012_W2 Does anyone ever help you eat? 吃饭的时候是否有人帮助你？

1. Yes 有
2. No 没有

DB013 Do you have any difficulty with getting into or out of bed? 您起床、下床有没有困难？

1. No, I don't have any difficulty 没有困难 → Skip to DB014 跳至DB014
2. I have difficulty but can still do it. 有困难但仍可以完成。
3. Yes, I have difficulty and need help. 有困难，需要帮助。
4. I can not do it. 无法完成

DB013_W2 Does anyone ever help you get in or out of bed? 起床、下床是否有人帮你？

1. Yes 有
2. No 没有

DB014 Because of health and memory problems, do you have any difficulties with using the toilet, including getting up and down? 请问您是否因为健康和记忆的原因，上厕所有困难，包括蹲下、站起？

1. No, I don't have any difficulty 没有困难 → Skip to DB022_W2_1 跳至DB022_W2_1
2. I have difficulty but can still do it. 有困难但仍可以完成。
3. Yes, I have difficulty and need help. 有困难，需要帮助。
4. I can not do it. 无法完成

DB014_W2 Does anyone ever help you use the toilet? 上厕所是否有人帮你？

1. Yes 有
2. No 没有

DB015 Because of health and memory problems, do you have any difficulties with controlling urination and defecation? If you use a catheter (conduit) or a pouch by yourself, then you are not considered to have difficulties. 请问您是否因为健康和记忆的原因，控制大小便有困难？（自己能够使用导尿管或者尿袋算能够控制自理）

1. No, I don’t have any difficulty 没有困难
2. I have difficulty but can still do it. 有困难但仍可以完成。
3. Yes, I have difficulty and need help. 有困难，需要帮助。
4. I can not do it. 无法完成

DB022_W2_1 LOOP BRANCHPOINT: IF THIS IS A CORE INTERVIEW and R DID NOT REPORT THAT RECEIVES HELP WITH LAST SERIES OF ADLs (DB010_W2 and DB011_W2 and DB012_W2 and DB013_W2 and DB014_W2 NOT 1), GO TO DB016

PRODUCERE 程序: BEGINNING OF DB022_W2_1 LOOP: QUESTIONS DB022_W2_1 THROUGH DB022_W2_3 ARE REPEATED FOR UP TO SEVEN HELPERS. THESE QUESTIONS ARE BASED ON THE ACTIVITIES WITH WHICH THE R REPORTED RECEIVING HELP IN THE ADL SERIES, DB010_W2 THROUGH DB014_W2 (DB010_W2 or DB011_W2 or DB012_W2 or DB013_W2 or DB014_W2 = 1): 如果受访者在 [穿衣/洗澡/吃饭/上下床/上厕所] 任何一项有困难且有人帮助，询问：

DB022_W2_1 PROCEDURE 程序: IF THIS IS THE FIRST TIME THROUGH THE ADL HELPER LOOP: 如果是第一次询问 ADL 的帮助者：

Who most often helps you with [dressing/bathing/eating/getting (in/out of) bed/using the toilet]? 请问在穿衣、洗澡、吃饭、起床、入厕这些活动的困难中，谁帮助您最多 (最多可选择 7 个)？

PROCEDURE 程序: IF THIS IS THE SECOND OR LATERTIME THROUGH THE ADL HELPER LOOP: 如果是第二次及以后询问 ADL 的帮助者：

Who is that? 帮助您的人是谁？

1. Spouse 配偶
2. Ex-spouse 前妻/前夫
3. Mother 母亲
4. Father 父亲
5. Mother-in-law 岳母/婆婆
6. Father-in-law 岳父/公公
7. Children[preload name] 子女 [子女姓名]
8. Sibling 兄弟姐妹
9. Sibling of spouse 配偶的兄弟姐妹
10. Brother-in-law, sister-in-law 姐夫/妹夫/嫂子/弟媳
11. Grandson 孙子/外孙子
12. Granddaughter 孙女/外孙女
13. Other relative 其他亲属
14. Paid helper(such as nanny) 雇佣人员（如：保姆）
15. Volunteer or Employee of facility 志愿者或者志愿机构人员
16. Nursing home 养老院人员
17. Other 其他人员

NOTE: "EMPLOYEE(S) OF FACILITY" APPEARS ON LIST ONLY FOR AN R CURRENTLY.LIVING IN A NURSING HOME OR WHO WAS LIVING IN A NURSING HOME OR HOSPICE WHEN S/HE DIED. 注意：养老院人员选项仅针对住在养老院或在养老院去世的受访者。

PROCEDURE 程序：IF helper is "grandchild" (DB022_W2_1 = 35 or 36)

DB022_W2_2 Which child was the parent of the grandchildren? 是哪个孩子的孩子？(choose all that apply 可多选)

1-20 Provide the list of children
21 Dead child 去世孩子

PROCEDURE 程序：IF helper is "other" (DB022_W2_1 = 41)

DB022_W2_3 What's that person's relationship with you? 提供帮助的人是您的什么人？____

DB022_W2_4 BRANCHPOINT: IF SEVEN ADL HELPERS HAVE ALREADY BEEN DESCRIBED, GO TO DB016 (OUT OF LOOP)

DB022_W2_4 Does anyone else help you with (this activity/these activities)? 除了上面提到的人以外，还有没有其他人帮助您？

1. Yes 有 Go to DB022_W2_1 loop
2. No 没有

DB016 Because of health and memory problems, do you have any difficulties with doing household chores? 请问您是否因为健康和记忆的原因，做家务活的时候有困难？(Definition: By doing household chores, we mean house cleaning, doing dishes, making the bed, and arranging the house. 定义：做家务，我们指的是房屋清洁，洗碗盘，整理被褥和房间摆设。) [IWER: If R cannot mop the floor, but can scrub, or R cannot fold heavy bedding, but is able to do light ones, then mark (3). 访员注意：如果受访者不能拖地，但是可以擦洗桌子，或者受访者不能整理重的被褥，但是可以整理一些轻便的，请选择 (3)。]

1. No, I don't have any difficulty 没有困难 → skip to DB017 跳至DB017
2. I have difficulty but can still do it. 有困难但仍可以完成。
3. Yes, I have difficulty and need help. 有困难，需要帮助。
4. I can not do it. 无法完成

DB016_W2 Does anyone help you do household chores? 做家务的时候是否有人帮助你？

1. Yes 有
2. No 没有

DB017 Because of health and memory problems, do you have any difficulties with preparing hot meals? 请问您是否因为健康和记忆的原因，做饭有困难？(Definition: By preparing hot meals, we mean preparing ingredients, cooking, and serving food. 定义：做饭我们定义为准备原材料，做饭菜，端上餐桌。) [IWER: If another person prepares ingredients or if R can cook rice, but is not able to prepare side dishes, then mark (3). 访员注意：如果由于健康原因，受访者需要别人帮忙洗菜切菜，或者受访者只能自己煮米饭但不能做菜，也就是说，由于健康原因受访者只能完成做饭的一些简单的动作，那么选择（3）。]

1. No, I don't have any difficulty 没有困难 → skip to DB018 跳至DB018
2. I have difficulty but can still do it. 有困难但仍可以完成。
3. Yes, I have difficulty and need help. 有困难，需要帮助。
4. I can not do it. 无法完成

**DB017_W2 BRANCHPOINT**: IF R LIVES IN NURSING HOME, GO TO DB018

DB017_W2 Does anyone help you prepare hot meals? 做饭的时候是否有人帮助你？

1. Yes 有
2. No 没有

DB018 Because of health and memory problems, do you have any difficulties with shopping for groceries? By shopping, we mean deciding what to buy and paying for it. 请问您是否因为健康和记忆的原因，自己去商店买食品杂货有困难？我们这里说的买东西是指决定买什么和付钱。

1. No, I don't have any difficulty 没有困难 → skip to DB035 跳至DB035
2. I have difficulty but can still do it. 有困难但仍可以完成。
3. Yes, I have difficulty and need help. 有困难，需要帮助。

4. I can not do it. 无法完成

DB018_W2 Does anyone help you shop for groceries? 是否有人帮助你去商店买食品杂货等?

1. Yes 有
2. No 没有

DB035 Because of health and memory problems, do you have any difficulties with making phone calls? 请问您是否因为健康和记忆的原因，拨打电话有困难?

1. No, I don't have any difficulty 没有困难 → skip to DB020 跳至DB020
2. I have difficulty but can still do it. 有困难但仍可以完成。
3. Yes, I have difficulty and need help. 有困难, 需要帮助。
4. I can not do it. 无法完成
5. Not relevant to me (no phone) 不适用，家里没有电话 → skip to DB020 跳至DB020

DB035_W2 Does anyone help you make telephone calls? 打电话的时候是否有人帮助你?

1. Yes 有
2. No 没有

DB020 Because of health and memory problems, do you have any difficulties with taking medications? By taking medications, we mean taking the right portion of medication right on time. 请问您是否因为健康和记忆的原因，自己吃药有困难? 吃药是指能记得什么时间吃和吃多少。

1. No, I don't have any difficulty 没有困难 → skip to DB022_W2_5 跳至DB022_W2_5
2. I have difficulty but can still do it. 有困难但仍可以完成。
3. Yes, I have difficulty and need help. 有困难, 需要帮助。
4. I can not do it. 无法完成

DB020_W2 Does anyone help you take medications? 吃药的时候是否有人帮助你?

1. Yes 有
2. No 没有

DB022_W2_5 LOOP BRANCHPOINT: IF THIS IS A CORE INTERVIEW and R DID NOT REPORT THAT SOMEONE HELPED WITH ANY OF THE 4 IADLs (DB016_W2 and DB018_W2 and DB035_W2 and DB020_W2 NOT 1), GO TO DB021

PROCEDURE 程序: BEGINNING OF DB022_W2_5 LOOP: QUESTIONS DB022_W2_5 THROUGH DB022_W2_8 ARE REPEATED FOR UP TO SIX HELPERS. THESE QUESTIONS ARE BASED ON THE ACTIVITIES WITH WHICH THE R REPORTED RECEIVING HELP IN THE IADL SERIES (DB016_W2 or DB017_W2 or DB018_W2 or DB020_W2 = 1): 如果受访者在 [做饭/购物/打电话/吃药] 任何一项需要帮助，询问:

DB022_W2_5 PROCEDURE 程序: IF THIS IS THE FIRST TIME THROUGH THE IADL HELPER LOOP: 如果是第一次询问 IADL 的帮助者:

Who most often helps you with [do household chroces/preparing hot meals/shopping/making telephone calls/taking medications]? 请问在 [做家务/做饭/购物/打电话/吃药] 这些活动的困难中，谁帮助您最多 (最多可选择 6 个)?

PROCEDURE 程序: IF THIS IS THE SECOND OR LATERTIME THROUGH THE ADL HELPER LOOP: 如果是第二次及以后询问 ADL 的帮助者:

Who is that? 帮助您的人是谁?

1. Spouse 配偶
2. Ex-spouse 前妻/前夫
3. Mother 母亲
4. Father 父亲
5. Mother-in-law 岳母/婆婆
6. Father-in-law 岳父/公公
7. Children[preload name] 子女 [子女姓名]
8. Sibling 兄弟姐妹
9. Sibling of spouse 配偶的兄弟姐妹
10. Brother-in-law, sister-in-law 姐夫/妹夫/嫂子/弟媳
11. Grandson 孙子/外孙子
12. Granddaughter 孙女/外孙女
13. Other relative 其他亲属
14. Paid helper(such as nanny) 雇佣人员（如：保姆）
15. Volunteer or Employee of facility 志愿者或者志愿机构人员
16. Nursing home 养老院人员
17. Other 其他人员

NOTE: “EMPLOYEE(S) OF FACILITY” APPEARS ON LIST ONLY FOR AN R CURRENTLY.LIVING IN A NURSING HOME OR WHO WAS LIVING IN A NURSING HOME OR HOSPICE WHEN S/HE DIED. **注意**: 养老院人员选项仅针对住在养老院或在养老院去世的受访者。

PROCEDURE 程序: IF helper is grandchild(DB022_W2_5 = 35 or 36)

DB022_W2_6 Which child was the parent of the grandchildren? 是哪个孩子的孩子? (choose all that apply 可多选)

1-20 Provide the list of children

21 Dead child 去世孩子

PROCEDURE 程序: IF helper is “other” (DB022_W2_5 = 41)

DB022_W2_7 What’s that person’s relationship with you? 提供帮助的人是您的什么人?

1-20 Provide the list of children

21 Dead child 去世孩子

DB022_W2_8 BRANCHPOINT: IF SIX IADL HELPERS HAVE ALREADY BEEN DESCRIBED, GO TO DB021 (OUT OF LOOP)

DB022_W2_8 Does anyone else help you with (this activity/these activities)? 除了上面提到的人以外，还有没有其他人帮助您？

1. Yes 有 Go to DB022_W2_5 loop
2. No 没有

DB021 Do you use the following auxiliary? (Code all that apply) 你使用以下辅助么？（可多选）

1. Walking stick 拐杖
2. Travel device 代步器
3. Manual wheelchair 手动轮椅
4. Electric Wheelchair 电动轮椅
5. Not any 一个都不用

DB019 Because of health and memory problems, do you have any difficulties with managing your money, such as paying your bills, keeping track of expenses, or managing assets? 请问您是否因为健康和记忆的原因，管钱有困难，比如支付账单、记录支出项目、管理财物？

1. No, I don't have any difficulty 没有困难
2. I have difficulty but can still do it. 有困难但仍可以完成。
3. Yes, I have difficulty and need help. 有困难，需要帮助。
4. I can not do it. 无法完成

DB019_W2 Does anyone help you manage your money? 是否有人帮助你管钱？

1. Yes 有
2. No 没有 skip DB022_W2_9 跳过 DB022_W2_9

**NOTE**: QUESTIONS DB022_W2_9 THROUGH DB022_W2_12 ARE REPEATED FOR UP TO TWO HELPERS.

DB022_W2_9 **PROCEDURE 程序**: IF THIS IS THE FIRST TIME THROUGH THE MANAGE MONEY HELPER LOOP: 如果是第一次询问管钱的帮助者：
Who most often helps you manage your money? 通常是谁给您管钱呢？(最多可选择 2 个)？
PROCEDURE 程序: IF THIS IS THE SECOND OR LATERTIME THROUGH THE MANAGE MONEY HELPER LOOP: 如果是第二次及以后询问管钱的帮助者：
Who is that? 帮助您的人是谁？

1. Spouse 配偶
2. Ex-spouse 前妻/前夫
3. Mother 母亲
4. Father 父亲
5. Mother-in-law 岳母/婆婆
6. Father-in-law 岳父/公公
7. Children[preload name] 子女 [子女姓名]
8. Sibling 兄弟姐妹
9. Sibling of spouse 配偶的兄弟姐妹
10. Brother-in-law, sister-in-law 姐夫/妹夫/嫂子/弟媳

11. Grandson 孙子/外孙子
12. Granddaughter 孙女/外孙女
13. Other relative 其他亲属
14. Paid helper(such as nanny) 雇佣人员（如：保姆）
15. Volunteer or Employee of facility 志愿者或者志愿机构人员
16. Nursing home 养老院人员
17. Other 其他人员

**PROCEDURE 程序**：IF helper is grandchild (DB022_W2_9 = 35 or 36)

DB022_W2_10 Which child was the parent of the grandchildren? 是哪个孩子的孩子？(choose all that apply 可多选)
1-20 Provide the list of children
21 Dead child 去世孩子

**PROCEDURE 程序**：IF helper is “other”(DB022_W2_9 = 41)

DB022_W2_11 What’s that person’s relationship with you? 他/她是您的什么人？____

**DB022_W2_12 BRANCHPOINT**: IF MANAGE MONEY HELPERS HAVE ALREADY BEEN DESCRIBED, GO TO DB022_1 (OUT OF LOOP)

DB022_W2_12 Does anyone else help you with (this activity/these activities)? 除了上面提到的人以外，还有没有其他人帮助您？
1. Yes 有 Go to DB022_W2_9 loop
2. No 没有

**PROCEDURE 程序**: For 7-30 or 31 chosen in DB022_W2_1, DB022_W2_5 and DB022_W2_9, ask DB022_1. 如果DB022_W2_1, DB022_W2_5和DB022_W2_9选择 7-30 或 31（即子女或孩子配偶提供帮助最多），对每一个人询问DB022_1。

DB022_1 Whether it is the [helper’s name chosen from DB022] him/herself taking the time? [DB022 选择的帮助者] 亲自帮助您的吗？
1. Yes 是
2. No 否

**PROCEDURE 程序**：For each helper chosen in DB022, ask DB023-DB026 对 DB022 选择的每一位提供帮助的人，提问DB023-DB026。

DB023 During the last month, on about how many days did [helper’s name chosen from DB022] help you? 在过去一个月内，[DB022 中选择的帮助者] 帮助了您多少天？ ____1…31 Days 天

DB024 On the days [helper's name chosen from DB022 helps you, about how many hours per day is that? 在 [DB022 中选择的帮助者] 帮助您的那些天，他／她大概每天花多少小时帮助您？ ____ 1...24Hours 小时 [IWER: less than an hour = 1 访员注意：少于一个小时请记为 1]

DB025 Is he/she living in your home? 他/她是否住在您家里？

1. Yes 是
2. No 否

DB026 Is [helper's name chosen from DB022 paid to help you? 请问 [DB022] 中选择的帮助者] 帮助您，您要付钱吗？

1. Yes 是
2. No 否

SKIP PATTERN CHECKPOINT: PAID HELPER 跳转点检查：付费的帮手

PROCEDURE 程序：IF R PAID FOR HELP IN DB026, SKIP TO DB027. 如果在DB026受访者为获得帮助而支付费用，请跳转至DB027。IF R DID NOT PAY FOR HELP, SKIP TO DB029. 如果受访者不需要帮助付费，那么跳转至DB029。

DB027 About how much in total did you pay (including value of the goods you gave them as repayment for their help) for the help during the past month? 在过去一个月内您给这些帮助您的人大约总共付了多少钱？

____ Yuan 元

DB028 Who contributed most to paying this cost? Please choose one person who paid the most. 这些钱谁出的最多，请选择一个支付的最多的人。(Select from the list displayed by CAPI) (请从 CAPI 列表中选择)

1. Yourself 您自己
2. Spouse 配偶
3. Mother 母亲
4. Father 父亲
5. Mother-in-law 岳母/婆婆
6. Father-in-law 岳父/公公
7. Children 子女
8. Spouse of child 子女的配偶
9. Sibling 兄弟姐妹
10. Sibling of spouse 配偶的兄弟姐妹
11. Brother-in-law, sister-in-law 姐夫/妹夫/嫂子/弟媳
12. Grandson 孙子/外孙子
13. Granddaughter 孙女/外孙女
14. Other relative 其他亲属

15. Other 其他人员

DB029 Do you use the following auxiliary? (Code all that apply) 你使用以下辅助工具么？（可多选）

1. Walking stick 拐杖
2. Travel device 代步器
3. Manual wheelchair 手动轮椅
4. Electric Wheelchair 电动轮椅
5. Catheter, urine collection bag 导尿管，导尿袋
6. Toilet Series 座便器
7. None of the above 以上都没有

DB030 Suppose that in the future, you needed help with basic daily activities like eating or dressing. Do you have relatives or friends (besides your spouse/partner) who would be willing and able to help you over a long period of time? 如果以后您在日常生活方面需要照顾，比如吃饭，穿衣，有亲人（除了配偶以外）或朋友能长期照顾您吗？

1. Yes 是
2. No 否 → Skip to DB032 请跳至DB032

DB031 What is the relationship to you of that person or those persons?（Choose all that apply）他/她是您的什么人？（可多选）(Select from the list displayed by CAPI)（请从 CAPI 列表中选择）

1. Yourself 您自己
2. Spouse 配偶
3. Mother 母亲
4. Father 父亲
5. Mother-in-law 岳母/婆婆
6. Father-in-law 岳父/公公
7. Children 子女
8. Spouse of child 子女的配偶
9. Sibling 兄弟姐妹
10. Sibling of spouse 配偶的兄弟姐妹
11. Brother-in-law, sister-in-law 姐夫/妹夫/嫂子/弟媳
12. Grandson 孙子/外孙子
13. Granddaughter 孙女/外孙女
14. Other relative 其他亲属
15. Other 其他人员

DB032 How often did the respondent receive assistance in answering this section? 受访者填写该部分问卷时是否求助？[IWER: If it is answered by a proxy, please record the respondent's reaction. 访员注意：如果是协助回答，请记录受访者的反应。]

1. Never 从不

2. A few times 有一些时候
3. Most or all of the time 大多数时候
4. The section was completed by a proxy respondent（the respondent is absent）受访者不在场，完全请人代填 → Skip to DB033 请跳至DB033

DB033 What is your relationship to R? 您和受访者是什么关系？[IWER: What is the proxy's relationship to R? If unknown, please ask the proxy. 访员注意：代填问卷的人和受访者是什么关系。如果不清楚，请问代填者。]

1. Spouse 配偶
2. Mother 母亲
3. Father 父亲
4. Mother-in-law 岳母/婆婆
5. Father-in-law 岳父公公
6. Sibling 兄弟姐妹
7. Brother-in-law, sister-in-law 姐夫妹夫/嫂子弟媳
8. Child 孩子
9. Spouse of child 孩子的配偶
10. Grandchild 孙子女
11. Other relative 其他亲戚
12. Helper or other non-relative 帮忙的人或者其他非亲属

DB034 [IWER: Please record the reason for proxy 访员注意：请记录代答的原因。]
What is the main reason for proxy（the respondent is absent）？受访者不在场，完全请人代填的主要原因是什么？

1. The respondent has serious physical handicaps 受访者有严重身体障碍
2. The respondent has serious mental handicaps 受访者有严重精神障碍
3. The respondent has rejected this interview. 受访者拒访
4. Other____. 其他 (**DA034_1**)

DC COGNITION & DEPPRESSION 认知和抑郁

[IWER: If DB032 = 4, then skip to Section E , health care and insurance. Sections Cc and Cd must not be answered by proxy respondents.] [访员注意：如果DB032 = 4，请跳至 E 部分医疗保健与保险。DC、DE 部分不能由别人代答.]

DC001 Now I'm going to ask several simple questions. Some may be easy and some may be hard to answer. Please try to answer as honestly as you can. Are you ready? Please tell me today's date.（Check all that apply）现在我将问几个问题，有些问题对于您来说可能很简单、有些问题可能就比较难。请告诉我今天的日期，是哪一年哪一月哪一日？（可多选）[IWER: R doesn't have to answer in this order. If R is an elderly person and marked the date by lunar calendar, that date is correct if it matches with the solar calendar. You

can check the accuracy, using the converter. 受访者不必按顺序回答，如果受访者是老人，并按农历来回答，而且如果日期对应的阳历是准确的，那么您可以按日历转换来检查其准确性。]

1. Year is correct 年正确
2. Month is correct 月正确
3. Day is correct 日正确

DC002 Please tell me the day of the week. Is it Monday, Tuesday, Wednesday, Thursday, Friday, Saturday, or Sunday? 请告诉我今天是星期几？

1. Day of week OK/correct 正确
2. Day of week not OK/incorrect 错误

DC003 What is the current season (among Spring, Summer, Fall, or Winter)? 现在是什么季节？

1. Season OK 季节正确
2. Season not OK 季节不正确

DC004 How would you rate your memory at the present time? Would you say it is excellent, very good, good, fair or poor? 您觉得自己现在的记忆力怎么样？是极好，很好，好，一般还是不好？

1. Excellent 极好
2. Very good 很好
3. Good 好
4. Fair 一般
5. Poor 不好

We are going to read a list consisting of 10 words and we would like you to memorize as many as you can. We deliberately made the list long to make it difficult for anyone to memorize all of the words; most people will only remember a few of them. Please listen carefully as we read the list because we cannot repeat it. When we finish reading the list, we will ask you to recall and tell us as many words as you can remember, and they don't have to be in the order that you heard them. Is this explanation clear? 我们将会给您读十个词，请仔细听，我们不会重复读。请尽可能多地记。我们是有意列这么多词以增加记忆的难度。读完后，会请您回忆这些词，不需要按顺序。您明白了吗？
[IWER: Do not allow proxy answers.] [访员注意：请不要让代理人答]

DC008 [CAPI automatically record the current time: hour and minute. CAPI 自动输入现在的时间：___(**DC008_1**) 时 _____(**DC008_2**) 分（24 小时制）]。

Try to remember the words I just read to you. I'll ask you to recall them later. 请尽量记住我刚才念给您听的词，一会我将请您回忆它们。[IWER: Read once more if R did not recall any of the words, up to 3 times, and then go on. If R does not recall any of the words,assure them that it is OK so that R will feel comfortable/at ease.] [访员注意：如果受访者没回忆出任何一个词汇，请再读 1 遍，直到读第 3 遍。如果受访者回忆不出这些词汇，请告诉他们没关系，以便让他们放松。]

DC006_1 [IWER: Record the times you read to the respondent] 请记录为受访者读了几遍！

Try to remember the words I just read to you. I'll ask you to recall them later. 请尽量记住我刚才念给您听的词，一会我将请您回忆它们。
The 10 items below refer to how you have felt and behaved during the last week. Choose the appropriate response. 下面 10 道问题是有关您上周的感觉及行为，每道题目的答案都是一样的，如卡片 12 所示，包括很少或者根本没有，不太多，有时或者说有一半的时间还是大多数的时间，请您选择合适的答案。
[Show Card 出示卡片 12]

DC009 I was bothered by things that don't usually bother me. 我因一些小事而烦恼。
1. Rarely or none of the time (< 1 day) 很少或者根本没有（< 1 天）
2. Some or a little of the time (1 – 2 days) 不太多（1 – 2 天）
3. Occasionally or a moderate amount of the time (3 – 4 days) 有时或者说有一半的时间（3 – 4 天）
4. Most or all of the time (5 – 7 days) 大多数的时间 (5 – 7 天)

DC010 I had trouble keeping my mind on what I was doing. 我在做事时很难集中精力。
1. Rarely or none of the time (< 1 day) 很少或者根本没有（< 1 天）
2. Some or a little of the time (1 – 2 days) 不太多（1 – 2 天）
3. Occasionally or a moderate amount of the time (3 – 4 days) 有时或者说有一半的时间（3 – 4 天）
4. Most or all of the time (5 – 7 days) 大多数的时间 (5 – 7 天)

DC011 I felt depressed. 我感到情绪低落。
1. Rarely or none of the time (< 1 day) 很少或者根本没有（< 1 天）
2. Some or a little of the time (1 – 2 days) 不太多（1 – 2 天）
3. Occasionally or a moderate amount of the time (3 – 4 days) 有时或者说有一半的时间（3 – 4 天）
4. Most or all of the time (5 – 7 days) 大多数的时间 (5 – 7 天)

DC012 I felt everything I did was an effort. 我觉得做任何事都很费劲。
1. Rarely or none of the time (< 1 day) 很少或者根本没有（< 1 天）
2. Some or a little of the time (1 – 2 days) 不太多（1 – 2 天）
3. Occasionally or a moderate amount of the time (3 – 4 days) 有时或者说有一半的时间（3 – 4 天）
4. Most or all of the time (5 – 7 days) 大多数的时间 (5 – 7 天)

DC013 I felt hopeful about the future. 我对未来充满希望。
1. Rarely or none of the time (< 1 day) 很少或者根本没有（< 1 天）
2. Some or a little of the time (1 – 2 days) 不太多（1 – 2 天）
3. Occasionally or a moderate amount of the time (3 – 4 days) 有时或者说有一半的时间（3 – 4 天）

4. Most or all of the time (5 – 7 days) 大多数的时间 (5 – 7 天)

DC014 I felt fearful. 我感到害怕。

1. Rarely or none of the time (< 1 day) 很少或者根本没有（< 1 天）
2. Some or a little of the time (1 – 2 days) 不太多（1 – 2 天）
3. Occasionally or a moderate amount of the time (3 – 4 days) 有时或者说有一半的时间（3 – 4 天）
4. Most or all of the time (5 – 7 days) 大多数的时间 (5 – 7 天)

DC015 My sleep was restless. 我的睡眠不好。

1. Rarely or none of the time (< 1 day) 很少或者根本没有（< 1 天）
2. Some or a little of the time (1 – 2 days) 不太多（1 – 2 天）
3. Occasionally or a moderate amount of the time (3 – 4 days) 有时或者说有一半的时间（3 – 4 天）
4. Most or all of the time (5 – 7 days) 大多数的时间 (5 – 7 天)

DC016 I was happy. 我很愉快

1. Rarely or none of the time (< 1 day) 很少或者根本没有（< 1 天）
2. Some or a little of the time (1 – 2 days) 不太多（1 – 2 天）
3. Occasionally or a moderate amount of the time (3 – 4 days) 有时或者说有一半的时间（3 – 4 天）
4. Most or all of the time (5 – 7 days) 大多数的时间 (5 – 7 天)

DC017 I felt lonely. 我感到孤独。

1. Rarely or none of the time (< 1 day) 很少或者根本没有（< 1 天）
2. Some or a little of the time (1 – 2 days) 不太多（1 – 2 天）
3. Occasionally or a moderate amount of the time (3 – 4 days) 有时或者说有一半的时间（3 – 4 天）
4. Most or all of the time (5 – 7 days) 大多数的时间 (5 – 7 天)

DC018 I could not get ”going.” 我觉得我无法继续我的生活。

1. Rarely or none of the time (< 1 day) 很少或者根本没有（< 1 天）
2. Some or a little of the time (1 – 2 days) 不太多（1 – 2 天）
3. Occasionally or a moderate amount of the time (3 – 4 days) 有时或者说有一半的时间（3 – 4 天）
4. Most or all of the time (5 – 7 days) 大多数的时间 (5 – 7 天)

[IWER: Try to persuade R to answer if R refuses at first. Record the exact number R says.]
[访员注意：尽力说服受访者作答。正确地记录受访者回答的数字。]

DC019 Let’s try some subtraction of numbers this time. What does 100 minus 7 equal? 我们将问您一些减法。100 减去 7 等于多少？____

DC020 And 7 from that? 再减去 7 等于多少? ____

DC021 And 7 from that? 再减去 7 等于多少? ____

DC022 And 7 from that? 再减去 7 等于多少? ____

DC023 And 7 from that? 再减去 7 等于多少? ____

DC024 [IWER: Please indicate whether the respondent used paper and pencil or any other aid when completing the number subtraction.] [访员注意：受访者在回答这些算术题时，是否用了纸、笔或其他辅助工具？]

1. used aid 用了辅助工具
2. did not use aid 没用辅助工具

[Show Card 出示卡片 32]

DC025 Do you see this picture? Please draw that picture on this paper. 你看到这张图片了吗？请在这张纸上把该图片画出来。[IWER: Show the picture of two pentagons overlapped.] [访员注意：出示一张两个五角星重叠的图片]

1. Drew the picture 画出了图片
2. Failed to draw the picture 不能画出该图片

DC026 [CAPI automatically record the current time: hour and minute. 输入现在的时间：___(**DC026_1**) 时 _____(**DC026_2**) 分（24 小时制）]

DC027 A little while ago, I read you a list of words and you repeated the ones you could remember. Please tell me any of the words that you remember now. 刚才我给您读了一些词汇，您也重复了您记得的词汇。请告诉我现在您还记得的词汇。[IWER: Answers are displayed only for interviewer. Please do not show the screen to R.] [访员注意：只有访员可以看到答案。请勿将显示屏展示给受访者看。]

DC028 Please think about your life-as-a-whole. How satisfied are you with it? Are you completely satisfied, very satisfied, somewhat satisfied, not very satisfied, or not at all satisfied? 总体来看，您对自己的生活是否感到满意？是极其满意，非常满意，比较满意，不太满意还是一点也不满意？

1. Completely satisfied 极其满意
2. Very satisfied 非常满意
3. Somewhat satisfied 比较满意
4. Not very satisfied 不太满意
5. Not at all satisfied 一点也不满意

E HEALTH CARE AND INSURANCE 医疗保健与保险

CARD21 : Health Facilities 医疗机构

1. General hospital 综合医院（即全科医院，不包括中医院）
2. Specialized hospital 专科医院（不包括中医院）
3. Chinese medicine hospital 中医院
4. Community healthcare center 社区卫生服务中心
5. Township hospital 乡镇卫生院
6. Health care post 卫生服务站
7. Village clinic/Private clinic 村诊所/私人诊所
8. Other 其他

CARD20 : Health Insurance 医疗保险

1. Urban employee medical insurance (yi-bao) 城镇职工医疗保险（医保）
2. Urban resident medical insurance 城镇居民医疗保险
3. New cooperative medical insurance (he-zuo-yi-liao) 新型农村合作医疗保险（合作医疗）
4. Urban and rural resident medical insurance 城乡居民医疗保险（合并城镇居民和新型农村合作医疗保险）
5. Government medical insurance (gong-fei) 公费医疗
6. Medical aid 医疗救助
7. Private medical Insurance: Purchased by R's union 商业医疗保险：单位购买
8. Private medical Insurance: Purchased by Individual 商业医疗保险：个人购买
9. Urban non-employed persons's health insurance 城镇无业居民大病医疗保险
10. Other medical insurance (specify) 其他医疗保险（请注明）
11. No insurance 没有保险

CARD23 : Health Facilities for Inpatient Care 住院治疗的医疗机构

1. General Hospital 综合医院（即全科医院，不包括中医院）
2. Specialized hospital 专科医院（不包括中医院）
3. Chinese Medicine Hospital 中医院
4. Community Healthcare Center 社区卫生服务中心
5. Township Hospital 乡镇卫生院
6. Health care post 卫生服务站
7. Other 其他

PART I MEDICAL INSURANCE 第一部分：医疗保险

Now we would like to know about health insurance or benefits that you might have. 现在我们想了解一下您享受的健康保险或福利。

[Show Card 出示卡片 14]

EA001 Are you the policy holder/primary beneficiary of any of the types of health insurance listed below? (circle all that apply) 您本人目前是否参加了以下医疗保险？(可多选)

1. Urban employee medical insurance (yi-bao) 城镇职工医疗保险（医保）
2. Urban resident medical insurance 城镇居民医疗保险
3. New cooperative medical insurance (he-zuo-yi-liao) 新型农村合作医疗保险（合作医疗）
4. Urban and rural resident medical insurance 城乡居民医疗保险（合并城镇居民和新型农村合作医疗保险）
5. Government medical insurance (gong-fei) 公费医疗
6. Medical aid 医疗救助
7. Private medical Insurance: Purchased by R's union 商业医疗保险：单位购买
8. Private medical Insurance: Purchased by Individual 商业医疗保险：个人购买
9. Urban non-employed persons's health insurance 城镇无业居民大病医疗保险
10. Other medical insurance (specify) 其他医疗保险，请注明 ____ (**EA001_1**)
11. No insurance 没有保险 Skip to EA009 请跳至EA009

[Soft check: If pick 11, cannot pick any other, "you chose no insurance and a specific type of insurance, this is not possible" 如果选 11，不能选其他项，"您不能既选择没有保险，又选择有某一种保险"]

F1:（1）"城镇职工医疗保险"指依法对城镇职工的基本医疗权利给予保障的社会医疗保险制度。

(2)"城镇居民医疗保险"指 2007 年 7 月国务院决定在全国 79 个城市实行城镇居民基本医疗保险试点。参保范围：城镇非从业居民，包括：不属于城镇职工基本医疗保险制度覆盖范围的中小学阶段的学生（包括职业高中、中专、技校学生)、少年儿童和其他非从业城镇居民。

(3)"新型农村合作医疗保险"指由政府组织、引导、支持，农民自愿参加，个人、集体和政府多方筹资，以大病统筹为主的农民医疗互助共济制度。采取个人缴费、集体扶持和政府资助的方式筹集资金。

(4)"城乡居民医疗保险"指一些地区率先将城镇居民医疗保险与新型农村合作医疗合并，推行统一的城乡居民医疗保险制度。

(5)"外来务工人员医疗保险"指在某些地区在城镇职工基本医疗保险以外，专为外来务工人员设置的一种医疗保险制度。

(6)"公费医疗"指国家为保障国家工作人员身体健康而实行的一项社会保障制度。其享受对象主要是各级国家机关、党派、团体以及文化、教育、科研、卫生、体育等事业单位的工作人员和离退休人员，二等乙级以上革命伤残军人以及在校大学生等。

(7)"医疗救助"指在政府的支持下，依靠社会力量建立的旨在向困难群体提供某些或全部基本医疗服务的制度。

(8)"商业医疗保险"是相对于社会保险而言的。商业医疗保险由商业保险公司经办，以营利为目的，企业或职工自愿参加。

(9)"其他医疗保险"如中国职工保险互助会《在职职工重大疾病互助保障计划》和《生育保险》。

（10）"医疗救助"在部分区县试点，救助标准是由各区县根据自身经济情况制定，导致救助标准有高有低。主要三种，一是提供社会医疗救助金，给救助对象以经济补偿；二是给医疗机构一定的经济补贴，使后者直接减免救助对象的部分医疗费；三是由社会医疗救助机构举办专门医疗机构，免费为救助对象提供医疗服务。

（11）"医疗保险"是指基本医疗保险，是为补偿疾病所带来的医疗费用的一种保险。职工因疾病、负

伤、生育时，由社会或企业提供必要的医疗服务或物质帮助的社会保险。如中国的公费医疗、劳保医疗。
(12) 城镇无业居民大病医疗保险参保范围是具有本市非农业户籍，男满 16 周岁不满 60 周岁，女满 16 周岁不满 50 周岁，未纳入城镇职工基本医疗保险覆盖范围的居民。筹资标准为每人每年 700 元，其中个人缴纳 600 元、财政补助 100 元。

For each circled type of insurance (1-10), ask the following questions EA002 - EA008. 对以上选择的 1-10 的保险类型，回答以下问题EA002 - EA008。

**PROCEDURE 程序**：IF EA001 = 7 or 8, skip EA002 如果参保的是商业保险，不询问是否参加补充医保

EA002 Do you have supplemental insurance to this plan? 你有没有参加补充医疗保险？(例如大病医疗等)

1. Yes 有
2. No 无

F1："补充医疗保险"指由于国家的基本医疗保险只能满足参保人的基本医疗需求，超过基本医疗保险范围之外的医疗需求可以通过补充医疗保险予以补充。是相对于基本医疗保险而言的，包括企业补充医疗保险、商业医疗保险、社会互助和社区医疗保险等多种形式。

EA003 Where did you set up your insurance account/policy? 您的保险是在哪里办的?

1. This county 本县/市
2. (if it is not in this county) the place of your HuKou (如户口不在本县/市) 户籍所在地
3. Other 其它 ______(**EA003_1**)province 省 ________(**EA003_2**)county 市/县

EA004 Method of reimbursement 报销方式如何？

1. Get reimbursement immediately 看病当时即可报销
2. You pay first and get reimbursed later. 自己当时先垫付以后报销

EA005 Through which agency did you purchase your primary plan? 您的主要保险是通过什么机构办理的？

1. Community committee/ village committee 居委会/村委会
2. R's union 单位
3. Agency of Social insurance 社保经办机构
4. Private insurance company 商业保险机构
5. Other 其他

EA006 What's your out-of-pocket yearly premium? 您每年要自己缴纳多少保险费 (包括主保险费和补充医疗保险费)?

____Yuan 元 [soft check upper limit: 15,000 for choice 1, 3,000 choice 2, 1,000 choice 3, 1,500 choice 4, 4,000 choice 7, 2,500 choice 9 程序检查：上限：选项 1，15,000；选项 2，3,000；选项 3，1,000；选项 4，1,500；选项 7，4,000；选项 9，2,500]

EA007 Who pay the premium for you? (choose all that apply) 谁给您交保费？(可多选)

1. Myself 自己
2. Children 子女
3. Relatives 亲属
4. Government 政府
5. R's union 单位（包括村集体）
6. Loan 借款
7. Donate 捐赠
8. Others 其他 Specify 请注明 (**EA007_1**)

EA008 When did this benefit begin? 您是什么时候参加这份保险的？[IWER: Mark the year using four digits. Take down the month as its actual number. For example, write January as "1" not "01", December as "12". If do not remember month, fill '0'. 访员注意：用 4 位数表示年，按照实际的月份填写月。例：1 月写作 "1"，而不是 "01"，12 月写作 "12"。如果记不住月份，请填入 '0'。]

1900...2013Year 年 (**EA008_1**)____, 0...12Month 月 (**EA008_2**)____

**PROCEDURE 程序**：Skip to EC001 for new R 如果是新受访者，请跳至EC001

**PROCEDURE 程序**：For XRType = REIW who had health insurance in last wave 上期参保的回访受访者：

EA010[i]_W2 Our records from your last interview in R's LAST IW MONTH, YEAR show that you have had [preload health insurance], is this right? [加载上次访问日期] 的访问记录显示您当时参加了 [医疗保险加载]，这个记录对吗？

1. Yes 是
2. No 否

**PROCEDURE 程序**：IF change to another health insurance program in this wave 如果本期参保的项目与上期不一致：IF DEA010_W2 = 1, ask EB003,EB004 如果上次访问正确，询问EB003,EB004；IF DEA010_W2 = 2, ask EB002 - EB004 如果上次访问错误，询问是参保历史EB002 - EB004。

**PROCEDURE 程序**：IF uninsured this wave 如果上期有保险，本期没有保险：IF DEA010_W2 = 1, ask EA009,EB003,EB004 如果上次访问正确，询问退保情况EA009,EB003,EB004 ；IF DEA010_W2 = 2, ask EA009,EB002 - EB004 如果上次访问错误，询问参保历史EA009,EB001 - EB004。

EA009 What is your main reason for not having health insurance? 您目前没有参加任何医疗保险的主要原因是什么？

1. I do not need it 不需要
2. Cannot afford it 支付不起保险费

3. Do not know where or from whom to get it 不知道该去哪办
4. Do not trust the institutions that offer health insurance 不相信健康保险机构
5. Do not have suitable programs for me to buy 没有合适的保险项目
6. Do not know/never thought of it 从没有想过这个问题
7. Others 其他原因

EB001 Did you have health insurance at any time? 您以前参加过医疗保险吗？
1. Yes 是
2. No 否 → Skip to EC001 请跳至 EC001

F1:"医疗保险"是指基本医疗保险，是为补偿疾病所带来的医疗费用的一种保险。职工因疾病、负伤、生育时，由社会或企业提供必要的医疗服务或物质帮助的社会保险。如中国的公费医疗、劳保医疗。

[Show Card 出示卡片 14]

EB002 What type of insurance did you have? 您参加的是以下哪几种保险？（可多选）
1. Urban employee medical insurance (yi-bao) 城镇职工医疗保险 (医保)
2. Urban resident medical insurance 城镇居民医疗保险
3. New cooperative medical insurance (he-zuo-yi-liao) 新型农村合作医疗保险（合作医疗）
4. Urban and rural resident medical insurance 城乡居民医疗保险（合并城镇居民和新型农村合作医疗保险）
5. Government medical insurance (gong-fei) 公费医疗
6. Medical aid 医疗救助
7. Private medical Insurance: Purchased by R's union 商业医疗保险：单位购买
8. Private medical Insurance: Purchased by Individual 商业医疗保险：个人购买
9. Urban non-employed persons's health insurance 城镇无业居民大病医疗保险
10. Other medical insurance (specify) 其他医疗保险，请注明 ____

[Soft check: If pick 11, cannot pick any other, "you chose no insurance and a specific type of insurance, this is not possible" 如果选 11，不能选其他项，"您不能既选择没有保险，又选择有某一种保险"]
F1：(1)"城镇职工医疗保险"指依法对城镇职工的基本医疗权利给予保障的社会医疗保险制度。
(2)"城镇居民医疗保险"指 2007 年 7 月国务院决定在全国 79 个城市实行城镇居民基本医疗保险试点。参保范围：城镇非从业居民，包括：不属于城镇职工基本医疗保险制度覆盖范围的中小学阶段的学生（包括职业高中、中专、技校学生）、少年儿童和其他非从业城镇居民。
(3)"新型农村合作医疗保险"指由政府组织、引导、支持，农民自愿参加，个人、集体和政府多方筹资，以大病统筹为主的农民医疗互助共济制度。采取个人缴费、集体扶持和政府资助的方式筹集资金。
(4)"城乡居民医疗保险"指一些地区率先将城镇居民医疗保险与新型农村合作医疗合并，推行统一的城乡居民医疗保险制度。
(5)"外来务工人员医疗保险"指在某些地区在城镇职工基本医疗保险以外，专为外来务工人员设置的一种医疗保险制度。
(6)"公费医疗"指国家为保障国家工作人员身体健康而实行的一项社会保障制度。其享受对象主要是

各级国家机关、党派、团体以及文化、教育、科研、卫生、体育等事业单位的工作人员和离退休人员，二等乙级以上革命伤残军人以及在校大学生等。
(7)“医疗救助”指在政府的支持下，依靠社会力量建立的旨在向困难群体提供某些或全部基本医疗服务的制度。
(8)“商业医疗保险”是相对于社会保险而言的。商业医疗保险由商业保险公司经办，以营利为目的，企业或职工自愿参加。
(9)“其他医疗保险”如中国职工保险互助会《在职职工重大疾病互助保障计划》和《生育保险》。
(10)“医疗救助”在部分区县试点，救助标准是由各区县根据自身经济情况制定，导致救助标准有高有低。主要三种，一是提供社会医疗救助金，给救助对象以经济补偿；二是给医疗机构一定的经济补贴，使后者直接减免救助对象的部分医疗费；三是由社会医疗救助机构举办专门医疗机构，免费为救助对象提供医疗服务。
(11)“医疗保险”是指基本医疗保险，是为补偿疾病所带来的医疗费用的一种保险。职工因疾病、负伤、生育时，由社会或企业提供必要的医疗服务或物质帮助的社会保险。如中国的公费医疗、劳保医疗。
(12)城镇无业居民大病医疗保险参保范围是具有本市非农业户籍，男满 16 周岁不满 60 周岁，女满 16 周岁不满 50 周岁，未纳入城镇职工基本医疗保险覆盖范围的居民。筹资标准为每人每年 700 元，其中个人缴纳 600 元、财政补助 100 元。

**PROCEDURE 程序**: For each circled type of insurance (1-10), ask EA008_W2_1 and EB003<br>针对每种所选的 1-10 的保险，分别问EA008_W2_1 and EB003

EA008_W2_1 When did this benefit begin? 您是什么时候参加这份保险的？[IWER: Mark the year using four digits. Take down the month as its actual number. For example, write January as “1” not “01” , December as “12” . If do not remember month, fill ‘0’. 访员注意：用 4 位数表示年，按照实际的月份填写月。例：1 月写作 “1”，而不是 “01” ,12 月写作 “12”。如果记不住月份，请填入’0’。]

1900. . .2013 Year 年 (**EA008_W2_1_1**)____, 0. . .12 Month 月 (**EA008_W2_1_2**)____

EB003 When did you lose it? 请问您什么时候退出这种保险的？[IWER: Mark the year using four digits. Take down the month as its actual number. For example, write January as “1” not “01” , December as “12” . If do not remember month, fill ‘0’. 访员注意：用 4 位数表示年，按照实际的月份填写月。例：1 月写作 “1”，而不是 “01” ,12 月写作 “12”。如果记不住月份，请填入’0’。]

1900. . .2013 Year 年 (**EB003_1**)____, 0. . .12Month 月 (**EB003_2**)____

EB004 Why did you lose it? 请问您为什么会退出这种保险？

1. Employer no longer exists. 单位不存在了
2. Such insurance will not be provided locally anymore. 本地不再提供这种保险
3. I resigned/was fired. 我从单位辞职/被辞退了
4. Other(please specify____ 其它，请注明 ____(**EB004_1**)

PART II HEALTH CARE COSTS AND UTILIZATION 第二部分：医疗成本与使用情况

[IWER: Please do not allow proxy to answer Part II. 访员注意：“医疗成本与使用情况”这部分不允许请别人代为回答]

EC001 PROCEDURE 程序: For XRType = NEWIW 新的受访者

When did you take the last physical examination? 您最近一次常规体检时什么时候？

PROCEDURE 程序: For XRType = REIW 回访受访者

When did you take the last physical examination since R's LAST IW MONTH, YEAR/in the last two years? 自上次访问以来，您最近一次常规体检时什么时候？

[IWER: Mark the year using four digits. Take down the month as its actual number. For example, write January as “1” not “01”, December as “12”. If do not remember month, fill ‘0’. 访员注意：用 4 位数表示年，按照实际的月份填写月。例：1 月写作“1”，而不是“01”,12 月写作“12”。如果记不住月份，请填入‘0’。]

1. 1900...2013Year 年 (**EC001_1**)____0...12 Month 月 (**EC001_2**)____
2. Didn't ever take physical examination yet 一辈子都没有参加过体检
3. Didn't take physical examination last two years 过去两年没有参加过常规体检

EC002 Who paid the physical examination cost? 谁付的体检费？

1. Myself 自己
2. Children 子女
3. Relatives 亲属
4. Government 政府
5. R's union 单位（包括村集体）
6. R's insurance 保险
7. Loan 借款
8. Donate 捐赠
9. Others 其他 ____ Specify 请注明 ____(**EC002_1**)

The next questions pertain to medical facilities or medical providers you may have visited for outpatient care during the past 1 month (excluding hospitalization). 下面我们要问问您过去一个月，到门诊看病或者接受治疗的情况（不包括住院）。

ED001 In the last month have you visited a public hospital, private hospital, public health center, clinic, or health worker's or doctor's practice, or been visited by a health worker or doctor for outpatient care? 过去一个月里，您是否去医疗机构看过门诊或者接受过上门医疗服务？(不包括去医院做体检)

1. Yes 是 Skip to ED004请跳至ED004
2. No 否

ED002 Have you been ill in the last month? 您在过去一个月内生过病吗？

1. Yes 是
2. No 否

PROCEDURE 程序:
If ED001 = 2 and ED002 = 2 skip to EE001 如果ED001 = 2 并且 ED002 = 2，请跳至EE001。
If ED001 = 1 skip to ED004 如果ED001 = 1，请跳至ED004。
If ED002 = 1 and ED001 = 2 go to ED003. 如果ED002 = 1 并且ED001 = 2，请跳至ED003。

ED003 What's the main reason for not seeking medical treatment? 您没有去看病的主要原因是什么?
1. Already under treatment. 之前已经看过医生了
2. Illness is not serious. Don't need treatment 病情不严重，不需要看医生
3. Poor 没有钱看病
4. No time 没有时间看病
5. Inconvenient traffic 去医院交通不方便
6. Poor service 医院服务态度不好
7. No available treatment 认为看医生没有用
8. Other 其他

[Show Card 出示卡片 15]

ED004 Which types of medical facilities have you visited in the last 4 weeks for outpatient treatment? (circle all that apply) 过去一个月，您去过哪些医疗机构接受门诊治疗?(可多选)
1. General hospital 综合医院（即全科医院，不包括中医院）
2. Specialized hospital 专科医院（不包括中医院）
3. Chinese medicine hospital 中医院
4. Community healthcare center 社区卫生服务中心
5. Township hospital 乡镇卫生院
6. Health care post 卫生服务站
7. Village clinic/Private clinic 村诊所/私人诊所
8. Other 其他

PROCEDURE 程序: For each item 1-7 checked in ED004, ask ED005: 对ED004 选择的 1-7 的每一种医疗机构，提问ED005:

ED005 How many times did you visit/been visited by [...] during the last month? 过去一个月中，您去这家医疗机构看过几次门诊? ____Times 次

PROCEDURE 程序: If sum(ED005)> 1, then ask ED006; otherwise, skip ED006。如果ED005 中的次数总和 > 1，则问ED006；否则，就跳过ED006。

ED006 How much did all the visits to [ED004 answer] cost during the last month? 您过去一个月去 [ED004 answer] 看病的总费用大概是多少? [IWER: If possible, please check the list of cost. 访员注意：如果可能，查看一下明细单]

1. Total cost 总费用 ____(ED006_1)Yuan 元; [soft check upper bound: 30,000 上限: 30,000. Brackets50/100/200/500/1000]
2. Didn't pay anything 没有付任何钱

**PROCEDURE 程序**：IF ED006 = 1, ASK ED007

ED007 1. Self-paid part 其中自付部分 ____ (ED007_1)Yuan 元 [Brackets50/100/200/500/1000]
2. Didn't pay anything. 没有付任何钱

Now I'd like to ask you some questions about your most recent visit to a health care provider in the last month. 下面我想了解一下您过去一个月最近一次就诊的情况。

ED008 Which health care provider did you visit most recently during the past month? 过去一个月，您最近一次去了哪种医疗机构就诊？[CAPI：Preload the health care providers in ED004. 程序：加载 ED004填的医疗机构。]

ED009 Is this facility public or private? 这家机构是公立的还是私立的？

1. Public 公立的
2. Private 私立的

**PROCEDURE 程序**：If ED008 = 1 – 3, ask ED010 如果 ED008 = 1 – 3，请回答 ED010

ED010 What's the level of this facility? 这家医疗机构是什么级别的？

1. County/district 县/市/区级
2. Regional /city 地/市
3. Provincial/ affiliated to a ministry 省/部属
4. Military 军队
5. Others 其他
6. Not applicable 不适用

ED012 Did the provider visit you at home? 这次是不是医护人员上门服务？

1. Yes 是 skip ED013 to ED016 and ED022，跳过 ED013 到 ED016 和 ED022
2. No 否

ED014 What is the travel time (one-way) to that facility? 您上次去这家医疗机构单程花多少时间？Minutes 分钟 ____(**ED014_1**)

How to go to the facility? 用什么方式去这家医疗机构？(**ED014_2**)

1. Walk 步行
2. Bus 公交车
3. Car 私家车或出租车

4. Ambulance 救护车
5. Bicycle or other manual vehicles 自行车或其他人力车
6. Electric bicycle/electric tricycle 电动自行车/电动三轮车
7. Motorcycle 摩托车
8. Tractor 拖拉机
9. Train 火车
10. Animal or animal-pulled cart 骑牲畜或坐畜力车

PROCEDURE 程序：If ED014 = 1, skip ED015 如果 ED014 = 1，跳过 ED015

ED015 What was the total transportation cost to the facility (including fuel cost, one way trip)? 您上次去这家医疗机构的单程交通成本是多少？（包括燃料费） ____ RMB 元 [softcheck upper limit: 600 上限：600]

ED017 What was the purpose of your visit? (circle all that apply) 您上次去这家医疗机构的目的是什么？(可多选)

1. Immunization 免疫接种
2. Consultation 咨询
3. Medical check-up 体检
4. Treatment for Illness 看病
5. Other 其他

PROCEDURE 程序：If ED017 = 4, then ask ED018 —ED021, ELSE ASK ED023. 如果ED017 答案中包含 4 "看病"，继续问ED018 —ED021 的问题，否则提问ED023。

ED018 Could you tell me the disease name? 您能告诉我您得了什么病吗？ ____

ED019 Was the visit a first visit or a follow-up visit for the symptom? 这次看病是第一次就诊还是复诊？

1. First 第一次就诊
2. Follow-up 复诊

ED020 Was the visit for ordinary outpatient service or an emergency? 这次看病是普通门诊还是急诊？

1. 普通门诊
2. 急诊

[Show Card 出示卡片 16]

ED021 What kind of treatment did you receive? (circle all that apply) 您这次去看病接受了以下哪种治疗？(可多选)

1. Injection 注射
2. Laboratorium test 化验
3. Surgery 手术

4. X-ray, CT, B ultrasonic, MRI X-光、CT、B 超、核磁共振等检查
5. Medications and purchase medical 用药、拿药
6. IV (Drip Infusion) 输液
7. Traditional treatment, eg massage, acupuncture 中医治疗，如按摩、针灸等
8. Other 其他

ED023 What was the total cost of this visit, including both treatment and medication cost (includes prescriptions you received)? 这次就诊（包括药费和诊疗费，药费包括在这家医疗机构或其他药店购买医生开的处方药）总共花了多少钱？

1. ____ RMB 元 (**ED023_1**) [softcheck upper limit 上限：30,000] [Brackets 25/50/120/400/1200]
2. There was no cost 没有费用

**PROCEDURE 程序**：IF ED023 = 1, ASK ED024

ED024 How much did you pay out of pocket, after reimbursement from insurance? 这次就诊所花的费用里，您自己支付了多少？

1. ____RMB 元 (**ED024_1**) [soft check upper limit: 30,000, also ED025 <= ED023_1, else "Pay out of pocket cannot be more than total cost" 上限：30,000，而且ED025 <= ED023_1，因为"自己支付的费用不可能多于总费用"]
2. Did not pay anything 没有付任何钱 Go to ED026 请跳至 ED026 [Brackets 15/10/100/300/1000]

ED025 Who contribute most for paying the out-of-pocket cost? 自付部分谁给您交得最多？

1. Myself 自己
2. Children 子女
3. Relatives 亲属
4. Government 政府
5. R's union 单位（包括村集体）
6. Loan 借款
7. Donate 捐赠
8. Others 其他 Specify 请注明

ED026 What was the total medication cost for this visit, including prescriptions you received? 这次就诊所付的药费 (包括在这家医疗机构或其他药店购买医生开的处方药) 一共是多少钱？

1. ____ RMB 元 (**ED026_1**) [soft check upper limit: 5,000, and must be no more than ED023_1. 上限：5,000，而且不能多于ED023_1] [Brackets 10/30/80/250/600]
2. Doctor did not write a prescription 没有开药 skip ED027 跳过ED027
3. Didn't fill prescription 开了方子，没有去取药 skip ED027 跳过ED027

ED027 How much will you eventually pay out of pocket for the medications from this visit, including prescriptions you received? 这次就诊所花的药费 (包括在这家医疗机构或其他药店购买医生开的处方药) 里，您自己支付了多少钱？

1. ____RMB 元 (**ED027_1**) [softcheck upper limit: 5,000 and ED027_1 must be no more than ED026_1 and no more than ED023_1. 上限：5,000 而且ED027_1 必须少于ED026_1，少于ED023_1] [Brackets 10/20/70/200/500]
2. Didn't pay anything 没有自付费用

[Show Card 出示卡片 14]

ED028 What insurance did you use or will you use?(circle all that apply) 这次就诊的费用（包含诊疗费和药费）您使用或将使用哪种医疗保险报销？（可多选）

1. Urban employee medical insurance (yi-bao) 城镇职工医疗保险（医保）
2. Urban resident medical insurance 城镇居民医疗保险
3. New cooperative medical insurance (he-zuo-yi-liao) 新型农村合作医疗保险（合作医疗）
4. Urban and rural resident medical insurance 城乡居民医疗保险（合并城镇居民和新型农村合作医疗保险）
5. Government medical insurance (gong-fei) 公费医疗
6. Medical aid 医疗救助
7. Private medical Insurance: Purchased by R's union 商业医疗保险：单位购买
8. Private medical Insurance: Purchased by Individual 商业医疗保险：个人购买
9. Urban non-employed persons's health insurance 城镇无业居民大病医疗保险
10. Other medical insurance (specify) 其他医疗保险，请注明 ____
11. Reimbursed by R's union 单位报销
12. No insurance 没有保险
13. Not revelent to me . 不适用

F1：（1）"城镇职工医疗保险"指依法对城镇职工的基本医疗权利给予保障的社会医疗保险制度。
（2）"城镇居民医疗保险"指 2007 年 7 月国务院决定在全国 79 个城市实行城镇居民基本医疗保险试点。参保范围：城镇非从业居民，包括：不属于城镇职工基本医疗保险制度覆盖范围的中小学阶段的学生（包括职业高中、中专、技校学生）、少年儿童和其他非从业城镇居民。
（3）"新型农村合作医疗保险"指由政府组织、引导、支持，农民自愿参加，个人、集体和政府多方筹资，以大病统筹为主的农民医疗互助共济制度。采取个人缴费、集体扶持和政府资助的方式筹集资金。
（4）"城乡居民医疗保险"指一些地区率先将城镇居民医疗保险与新型农村合作医疗合并，推行统一的城乡居民医疗保险制度。
（5）"外来务工人员医疗保险"指在某些地区在城镇职工基本医疗保险以外，专为外来务工人员设置的一种医疗保险制度。
（6）"公费医疗"指国家为保障国家工作人员身体健康而实行的一项社会保障制度。其享受对象主要是各级国家机关、党派、团体以及文化、教育、科研、卫生、体育等事业单位的工作人员和离退休人员，二等乙级以上革命伤残军人以及在校大学生等。
（7）"医疗救助"指在政府的支持下，依靠社会力量建立的旨在向困难群体提供某些或全部基本医疗服务的制度。
（8）"商业医疗保险"是相对于社会保险而言的。商业医疗保险由商业保险公司经办，以营利为目的，企业或职工自愿参加。

(9)“其他医疗保险”如中国职工保险互助会《在职职工重大疾病互助保障计划》和《生育保险》。
(10)“医疗救助”在部分区县试点，救助标准是由各区县根据自身经济情况制定，导致救助标准有高有低。主要三种，一是提供社会医疗救助金，给救助对象以经济补偿；二是给医疗机构一定的经济补贴，使后者直接减免救助对象的部分医疗费；三是由社会医疗救助机构举办专门医疗机构，免费为救助对象提供医疗服务。
(11)“医疗保险”是指基本医疗保险，是为补偿疾病所带来的医疗费用的一种保险。职工因疾病、负伤、生育时，由社会或企业提供必要的医疗服务或物质帮助的社会保险。如中国的公费医疗、劳保医疗。
(12)“单位报销”指一些福利较好的单位可以对本单位职工发生的除基本医疗保险报销部分以外的医疗费用进行全额或一定额度的报销。
(13)城镇无业居民大病医疗保险参保范围是具有本市非农业户籍，男满 16 周岁不满 60 周岁，女满 16 周岁不满 50 周岁，未纳入城镇职工基本医疗保险覆盖范围的居民。筹资标准为每人每年 700 元，其中个人缴纳 600 元、财政补助 100 元。

ED029 Did you give any “red envelopes” to the doctors for this visit? 您最近一次就诊有没有给医生送红包？

1. Yes 是
2. No 否

The following questions pertain to hospitalization (inpatient care) that you have had during the past year. 下面我们想了解一下您过去一年接受住院治疗的情况。

EE001 In the past year, did a doctor suggest that you needed inpatient care but you did not get hospitalized? 过去一年，是否有医生说您应该住院而没有住院的情况？

1. Yes 有
2. No 没有 Skip to EE003 请跳至EE003

EE002 What’s the main reason for not seeking hospitalization? 您没去住院的主要原因是什么？

1. Not enough money 没有钱
2. Not willing to go to the hospital 不愿意住院
3. Felt that hospital was unlikely to cure problem—hospital quality poor 住院也没有用，医院医疗条件差
4. Felt that care was unlikely to cure the problem—problem too serious 觉得问题很严重，住院也没用
5. No ward available 医院没有床位
6. Other 其他

EE003 Have you received inpatient care in the past year? 过去一年内，您住过院吗？

1. Yes 是
2. No 否 Skip to EF001 请跳至EF001

EE004 How many times have you received inpatient care during the past year? 过去一年，您接受过几次住院治疗？ ____Times 次

**PROCEDURE 程序**：If EE004 = 1, skip to EE007. 如果EE004 = 1，跳至EE007。

EE005 What was the medical cost for all the hospitalizations you received during the past year? (Only include fees paid to the hospital, including ward fees but excluding wages paid to a hired nurse, transportation costs, and accommodation costs for yourself or family members.) 过去一年住院的总费用大概是多少?（只包括付给医院的费用，不包括陪护的工资、自己或家人的交通费和住宿费，但包括医院病房费。）

1. Total cost 总费用 ____(**EE005_1**)Yuan 元; [Brackets 1500/3000/7000/15000/30000]

2. Didn't pay anything. 没有付任何钱。[softcheck upper limit 上限: 300,000]

**PROCEDURE 程序**：IF EE005 = 1 ASK EE006.

EE006

1. Self-paid part 其中自付部分 ____(**EE006_1**)Yuan 元
2. Didn't pay anything. 没有付任何钱

[softcheck upper limit: 300,000 and EE005 B.1<=EE005 A.1. 上限：300,000，而且EE005 B.1<=EE005 A.1.] [Brackets 600/1500/4000/8000/18000]

We want details about the last hospitalization you had in the past year. 我们想了解您在过去一年中，最后一次住院的详细情况。

**PROCEDURE 程序**：If ED001 = 1 ask EE007. 如果ED001 = 1 问EE007。

EE007 Is this the same facility as mentioned in ED008 for outpatient care? 这家医院是[ED008] 吗？

1. Yes 是 Skip to EE016 跳至EE016
2. No 否

**PROCEDURE 程序**：If EE007 = 2 or ED001 = 2 ask EE008 -EE015. 如果EE007 = 2 或者ED001 = 2 问EE008-EE015。

[Show Card 出示卡片 17]

EE008 What is the type of health or service facility which you visited for last inpatient care (hospital admissions)/for your most recent hospitalization in the past year? 过去一年中，您最后一次接受住院治疗所在的医疗机构的类型是什么？

1. General Hospital 综合医院（即全科医院，不包括中医院）
2. Specialized hospital 专科医院（不包括中医院）
3. Chinese Medicine Hospital 中医院
4. Community Healthcare Center 社区卫生服务中心
5. Township Hospital 乡镇卫生院

6. Health care post 卫生服务站
7. Other 其他

EE009 Is this facility public or private? 这家医疗机构是公立的还是私立的?
1. Public 公立的
2. Private 私立的

PROCEDURE 程序: If EE008 = 1 – 3, ask EE010, 如果EE008 = 1 – 3, 请回答EE010。

EE010 What's the level of this facility? 这家医疗机构是什么级别的?
1. County/district 县/市/区级
2. Regional /city 地/市
3. Provincial/ affiliated to a ministry 省/部属
4. Military 军队
5. Others 其他

EE012 What is the location of this facility? 这家医疗机构的具体的地址是什么?
1. province 省 (**EE012_1**)
   A. This province 本省
   B. Other province, (preload province) 外省（选择省份）(**EE012_1_1**) [IWER: Choose from the list of provinces, see appendix 2 访员注意：请选择省份，见附表二]
2. county/city 县/市 (**EE012_2_1**)
   A. This county / city 本县/市
   B. Other county / city, specify 外县/市县/市 (**EE012_2_2**)
3. township/district 乡/区 (**EE012_3_1**)
   A. This township/district 本乡/ 区
   B. Other township/district, specify 外乡/区 (**EE012_3_2**) 乡/区
4. village/street 村/街道 (**EE012_1_1**)
   A. This village/street 本村/街道
   B. Other village/street, specify 外村/街道 (**EE012_4_2**)

EE013 How many kilometers is it from the medical facility to your residence? 从您家到这家医疗机构有几公里? ____ Km 公里 [softcheck upper limit 上限：3000]

EE014 What is the travel time (one-way) to that facility? 您上次去这家医疗机构接受住院治疗单程花多少时间?

Unit 单位 (**EE014_1**)
1. ____Minute 分钟 [check range 范围：1-59]
2. ____Hour 小时 [softcheck upper limit 上限：20]

How to go to the facility? 用什么方式去这家医疗机构？(**EE014_2**)
1. Walk 步行
2. Bus 公交车

3. Car 私家车或出租车
4. Ambulance 救护车
5. Bicycle or other manual vehicles 自行车或其他人力车
6. Electric bicycle/electric tricycle 电动自行车/电动三轮车
7. Motorcycle 摩托车
8. Tractor 拖拉机
9. Train 火车
10. Animal or animal pulled cart 骑牲畜或坐畜力车

**PROCEDURE 程序**：IF EE014_2 = 2 – 10, ask EE015

EE015 What was the total transportation cost to the facility (including fuel cost, one way trip)? 您上次去这家医疗机构接受住院治疗的单程交通成本是多少钱？（包括燃料费）____RMB 元 [soft check upper limit 上限：600]

EE016 How many nights were you hospitalized there? 您在那里住了几晚？____Nights 个晚上 [softcheck upper limit 上限：40]

EE017 What was the starting date of your hospital stay? 您从哪一天开始住院？
____1900...2013 (**EE017_1**)Year 年 ____0...12 (**EE017_2**)Month 月 ____0...31 (**EE017_3**)Day 日 [IWER: Mark the year using four digits. Take down the month as its actual number. For example, write January as "1" not "01", December as "12". If do not remember month and day, fill '0'. 访员注意：用 4 位数表示年，按照实际的月份填写月。例：1 月写作"1"，而不是"01"，12 月写作"12"。如果记不住月份和日期，请填入'0'。]

EE018 What was your date of exit? 您哪天出院？
1. ____1900...2011 (**EE018_1**) Year 年 ____0...12 (**EE018_2**) Month 月 ____0...31 (**EE018_3**) Day 日
2. Still there 仍然在住院

[soft check: date of exit should be not before starting date, "exit date is before starting date, please ask R again", also exit date should be within 1 year of today 出院日期应该比住院日期要晚，"出院日期早于住院日期，请再问一次受访者"，出院日期应该在从今天往前数的一年之内。]

EE019 Why were you hospitalized? (Choose one choice) 您住院的原因是什么？（单选）
1. Sickness 生病
2. Accident 意外
3. Violence 暴力
4. Other 其他

EE020 Could you tell me the name of the disease? 您能告诉我们您是得了什么病住院的吗？
____

[Show Card 出示卡片 18]

EE021 During hospitalization, what kind of treatment did you receive? (circle all that apply) 在最近的一次住院期间，您接受了哪些治疗？（可多选）[IWER: Read one by one. 访问员注意：请逐项读出以下答案。]

1. Medical check-up/consultation 体检或者咨询
2. Injection 注射
3. Laboratory test 化验
4. Surgery 手术
5. X-ray, CT, B ultrasonic, MRI X-光、CT、B 超、核磁共振等检查
6. Medications 用药、拿药
7. IV (Drip Infusion) 输液
8. Traditional treatment, e.g., massage, acupuncture 中医治疗，如按摩、针灸等
9. Delivery 分娩
10. Other 其他

PROCEDURE 程序：IF EE018 = 2, please skip EE022. 程序：如果EE018 = 2，请跳过EE022。

EE022 Under what conditions did you leave the hospital? 您这次是在什么情况下出院的？

1. Fully recovered from illness, received doctor's approval 病完全好了，医生同意出院 Skip EE023 跳过EE023
2. Didn't recover from illness, but received doctor's suggestion to leave 病没有完全好，医生建议出院 Skip EE023 跳过EE023
3. Didn't recover from illness, requested to leave without doctor's suggestion 病没有完全好，自己要求出院
4. Other reasons 其他原因 Skip EE023 请跳过EE023

EE023 Why did you want to leave the hospital before you were recovered? 您要求提前出院的原因是什么？

1. Can't recover from illness 久病不愈
2. Poor 经济困难
3. No space in the hospital 没有床位
4. Limited hospital conditions 医院医疗条件所限
5. Poor quality and service from health care providers 医护人员服务态度不好
6. Other reasons 其他原因

EE024 What was the total medical cost of hospitalization? (Only include the fees paid to the hospital, excluding the wage of hired nurse, the fare or rent, but including the ward fees.) 这次住院一共花了多少钱？（只包括付给医院的费用，不包括陪护的工资、自己或家人的交通费和住宿费，但包括医院病房费。）

1. ____RMB 元 (**EE024_1**)[softcheck upper limit 上限：100,000] [Brackets 700/1500/3500/8000/15000]
2. There was no cost 没有费用

EE025 What was the total cost for hired nurse? 雇佣陪护照顾病人花了多少钱?

1. ____RMB 元 (**EE025_1**)
2. There was no cost 没有费用

EE026 What was the total cost for transportation, food and accommodation of patient and relatives? 自己和家人往返医院交通费、伙食费以及家人或陪护的住宿费一共花了多少钱?

1. ____RMB 元 (**EE026_1**)
2. There was no cost 没有费用

EE027 How much did you or will you eventually pay out of pocket for the total costs of hospitalization? 这次住院，您最后自己总共付了（将要付）多少钱？

1. ____RMB 元 (**EE027_1**) [softcheck upper limit: 100,000 and EE027_1 must be no more than EE024_1 上限：100,000，而且EE027_1 必须小于等于EE024_1]
2. Didn't pay anything. 没有付任何钱

EE028 Who contributes most for paying the out-of- pocket cost? 自付部分谁给您交得最多？

1. Myself 自己
2. Children 子女
3. Relatives 亲属
4. Government 政府
5. R's union 单位（包括村集体）
6. Loan 借款
7. Donate 捐赠
8. Others 其他 Specify 请注明

EE029 What was the total medication cost during this visit? 这次住院药费一共花了多少钱？

1. ____RMB 元 (**EE029_1**) [softcheck upper limit: 60,000 and EE029_1 should be no more than EE024_1 上限：60,000，而且EE029_1 应该小于等于EE024_1。]
2. Didn't receive 没有用药 Skip EE030 请跳过EE030
   [Brackets 200/500/1800/4000/8000]

EE030 How much did you pay out of pocket for medication costs during this visit? 这次住院，您自己一共付了多少钱的药费？

1. ____RMB 元 (**EE030_1**) [softcheck upper limit: 60,000 and EE030_1 should be no more than EE029_1 and EE024_1 上限：60,000，而且EE030_1 应该小于等于EE029_1 和EE024_1] [Brackets 100/300/1000/2500/5000]
2. Didn't pay anything. 没有付任何钱

[Show Card 出示卡片 14]

EE031 (Preload from EA001 or EB002) What insurance did you use or will you use? (circle all that apply) 您使用或将使用哪种医疗保险报销？（可多选）

1. Urban employee medical insurance (yi-bao) 城镇职工医疗保险（医保）
2. Urban resident medical insurance 城镇居民医疗保险

3. New cooperative medical insurance (he-zuo-yi-liao) 新型农村合作医疗保险（合作医疗）
4. Urban and rural resident medical insurance 城乡居民医疗保险（合并城镇居民和新型农村合作医疗保险）
5. Government medical insurance (gong-fei) 公费医疗
6. Medical aid 医疗救助
7. Private medical Insurance: Purchased by R's union 商业医疗保险：单位购买
8. Private medical Insurance: Purchased by Individual 商业医疗保险：个人购买
9. Urban non-employed persons's health insurance 城镇无业居民大病医疗保险
10. Other medical insurance (specify) 其他医疗保险，请注明 ____
11. Reimbursed by R's union 单位报销
12. No insurance 没有保险
13. Not revelent to me 不适用

F1：（1）“城镇职工医疗保险”指依法对城镇职工的基本医疗权利给予保障的社会医疗保险制度。
（2）“城镇居民医疗保险”指 2007 年 7 月国务院决定在全国 79 个城市实行城镇居民基本医疗保险试点。参保范围：城镇非从业居民，包括：不属于城镇职工基本医疗保险制度覆盖范围的中小学阶段的学生（包括职业高中、中专、技校学生）、少年儿童和其他非从业城镇居民。
（3）“新型农村合作医疗保险“指由政府组织、引导、支持，农民自愿参加，个人、集体和政府多方筹资，以大病统筹为主的农民医疗互助共济制度。采取个人缴费、集体扶持和政府资助的方式筹集资金。
（4）“城乡居民医疗保险”指一些地区率先将城镇居民医疗保险与新型农村合作医疗合并，推行统一的城乡居民医疗保险制度。
（5）“外来务工人员医疗保险”指在某些地区在城镇职工基本医疗保险以外，专为外来务工人员设置的一种医疗保险制度。
（6）“公费医疗”指国家为保障国家工作人员身体健康而实行的一项社会保障制度。其享受对象主要是各级国家机关、党派、团体以及文化、教育、科研、卫生、体育等事业单位的工作人员和离退休人员，二等乙级以上革命伤残军人以及在校大学生等。
（7）“医疗救助”指在政府的支持下，依靠社会力量建立的旨在向困难群体提供某些或全部基本医疗服务的制度。
（8）“商业医疗保险”是相对于社会保险而言的。商业医疗保险由商业保险公司经办，以营利为目的，企业或职工自愿参加。
（9）“其他医疗保险”如中国职工保险互助会《在职职工重大疾病互助保障计划》和《生育保险》。
（10）“医疗救助”在部分区县试点，救助标准是由各区县根据自身经济情况制定，导致救助标准有高有低。主要三种，一是提供社会医疗救助金，给救助对象以经济补偿；二是给医疗机构一定的经济补贴，使后者直接减免救助对象的部分医疗费；三是由社会医疗救助机构举办专门医疗机构，免费为救助对象提供医疗服务。
（11）“医疗保险”是指基本医疗保险，是为补偿疾病所带来的医疗费用的一种保险。职工因疾病、负伤、生育时，由社会或企业提供必要的医疗服务或物质帮助的社会保险。如中国的公费医疗、劳保医疗。
（12）“单位报销”指一些福利较好的单位可以对本单位职工发生的除基本医疗保险报销部分以外的医疗费用进行全额或一定额度的报销。
（13）城镇无业居民大病医疗保险参保范围是具有本市非农业户籍，男满 16 周岁不满 60 周岁，女满

16 周岁不满 50 周岁，未纳入城镇职工基本医疗保险覆盖范围的居民。筹资标准为每人每年 700 元，其中个人缴纳 600 元、财政补助 100 元。

EE032 Did you pay any "Red Envelopes" to the doctors for this visit? 这次住院期间有没有给医生送红包？

1. Yes 是
2. No 否

Now we'd like to know whether you have treated yourself during the past month. 下面我们想了解在过去一个月，您进行自我治疗的情况。
[Show Card 出示卡片 19]

EF001 How did you treat yourself during the past month? (circle all that apply) 过去一个月，你是否进行了以下自我治疗（注意：此处不包括凭处方取药的情况）？（可多选）[check, if choose 7 cannot choose other options 如果选择 7，就不能选其他选项。]

1. Consumed over-the-counter modern medicines 自己买非处方西药
2. Consumed prescription medicines 自己买处方西药
3. Consumed traditional herbs or traditional medicines as treatment 用传统中草药或者传统方法治疗
4. Tonic/Health supplement 吃维生素/补品/保健品
5. Use health care equipment 使用保健设备
6. Other 其他
7. None 没有 Skip to EH001 跳至EH001

**PROCEDURE 程序**：For each circled self-treatment method (1-6), ask questions EF002, EF003, EF005. 对每一种选择的 1-6 的自我治疗方式，均回答EF002，EF003，EF005。

EF002 What is the approximate total cost to [preload EF001] during the last month? 过去一个月，自己 [preload EF001] 的总成本大概是多少？

1. ____RMB 元 (**EF002_1**) [softcheck upper limits: 2,000 上限检查：2,000] [Brackets 10/30/100/200/300]
2. There was no cost. 没有费用。

**PROCEDURE 程序**：IF EF002 = 1 ASK EF003. 如果EF002 = 1，请回答EF003。

EF003 How much did you pay out-of-pocket? 除了报销的部分，您自己支付了多少？

1. ____RMB 元 (**EF003_1**) [softchecks upper limits: 2,000 and EF003_1 should be no more than EF002_1. 上限检查：2,000，而且EF003_1 应该小于等于EF002_1] [Brackets 10/30/100/200/300]
2. Didn't pay anything. 没有付任何钱 Go to EF005 请跳至EF005。

EF004 Who contribute most for paying the out-of-pocket cost? 自付部分谁给您交得最多？

1. Myself 自己
2. Children 子女
3. Relatives 亲属
4. Government 政府
5. R's union 单位（包括村集体）
6. Loan 借款
7. Donate 捐赠
8. Others 其他 Specify 请注明

[Show Card 出示卡片 14]

EF005 What insurance did you use? (circle all that apply) 您使用了哪种医疗保险报销？（可多选）

1. Urban employee medical insurance (yi-bao) 城镇职工医疗保险（医保）
2. Urban resident medical insurance 城镇居民医疗保险
3. New cooperative medical insurance (he-zuo-yi-liao) 新型农村合作医疗保险（合作医疗）
4. Urban and rural resident medical insurance 城乡居民医疗保险（合并城镇居民和新型农村合作医疗保险）
5. Government medical insurance (gong-fei) 公费医疗
6. Medical aid 医疗救助
7. Private medical Insurance: Purchased by R's union 商业医疗保险：单位购买
8. Private medical Insurance: Purchased by Individual 商业医疗保险：个人购买
9. Urban non-employed persons's health insurance 城镇无业居民大病医疗保险
10. Other medical insurance (specify) 其他医疗保险，请注明 ____
11. Reimbursed by R's union 单位报销
12. No insurance 没有保险
13. Not revelent to me 不适用

F1：（1）"城镇职工医疗保险"指依法对城镇职工的基本医疗权利给予保障的社会医疗保险制度。
(2)"城镇居民医疗保险"指 2007 年 7 月国务院决定在全国 79 个城市实行城镇居民基本医疗保险试点。参保范围：城镇非从业居民，包括：不属于城镇职工基本医疗保险制度覆盖范围的中小学阶段的学生（包括职业高中、中专、技校学生)、少年儿童和其他非从业城镇居民。
(3)"新型农村合作医疗保险"指由政府组织、引导、支持，农民自愿参加，个人、集体和政府多方筹资，以大病统筹为主的农民医疗互助共济制度。采取个人缴费、集体扶持和政府资助的方式筹集资金。
(4)"城乡居民医疗保险"指一些地区率先将城镇居民医疗保险与新型农村合作医疗合并，推行统一的城乡居民医疗保险制度。
(5)"外来务工人员医疗保险"指在某些地区在城镇职工基本医疗保险以外，专为外来务工人员设置的一种医疗保险制度。
(6)"公费医疗"指国家为保障国家工作人员身体健康而实行的一项社会保障制度。其享受对象主要是各级国家机关、党派、团体以及文化、教育、科研、卫生、体育等事业单位的工作人员和离退休人员，二等乙级以上革命伤残军人以及在校大学生等。

(7)“医疗救助”指在政府的支持下，依靠社会力量建立的旨在向困难群体提供某些或全部基本医疗服务的制度。
(8)“商业医疗保险”是相对于社会保险而言的。商业医疗保险由商业保险公司经办，以营利为目的，企业或职工自愿参加。
(9)“其他医疗保险”如中国职工保险互助会《在职职工重大疾病互助保障计划》和《生育保险》。
(10)“医疗救助”在部分区县试点，救助标准是由各区县根据自身经济情况制定，导致救助标准有高有低。主要三种，一是提供社会医疗救助金，给救助对象以经济补偿；二是给医疗机构一定的经济补贴，使后者直接减免救助对象的部分医疗费；三是由社会医疗救助机构举办专门医疗机构，免费为救助对象提供医疗服务。
(11)“医疗保险”是指基本医疗保险，是为补偿疾病所带来的医疗费用的一种保险。职工因疾病、负伤、生育时，由社会或企业提供必要的医疗服务或物质帮助的社会保险。如中国的公费医疗、劳保医疗。
(12)“单位报销”指一些福利较好的单位可以对本单位职工发生的除基本医疗保险报销部分以外的医疗费用进行全额或一定额度的报销。
(13)城镇无业居民大病医疗保险参保范围是具有本市非农业户籍，男满 16 周岁不满 60 周岁，女满 16 周岁不满 50 周岁，未纳入城镇职工基本医疗保险覆盖范围的居民。筹资标准为每人每年 700 元，其中个人缴纳 600 元、财政补助 100 元。

The following questions pertain to dental care that you have had during the past year. 下面我们想了解一下您过去一年看牙医的情况。

EH001 In the past year, have you seen a dentist for dental care, including dentures? 过去一年，您是否看过牙医？

1. Yes 有
2. No 没有 skip this part 跳过整个看牙部分

EH002 How many times have you received dental care during the past year? 过去一年，您看过几次牙医？ ____Times 次

EH003 What was the medical cost for all the dental care in the past year? 过去一年，看牙的总费用大概是多少?

1. Total cost 总费用 ____Yuan 元;
2. Didn't pay anything. 没有付任何钱。[softcheck upper limit 上限：300,000]

**PROCEDURE 程序**：IF EH003 = 1 ASK EH004

EH004

1. Self-paid part 其中自付部分 _______(**EG004_1**)Yuan 元
2. Didn't pay anything 没有付任何钱

**PROCEDURE 程序**：IF EH004 = “Didn't pay anything”，skip EH005

EH005 Who contribute most for paying the out-of-pocket cost? 自付部分谁给您交得最多？

1. Myself 自己
2. Children 子女
3. Relatives 亲属
4. Government 政府
5. R's union 单位（包括村集体）
6. Loan 借款
7. Donate 捐赠
8. Others 其他 Specify 请注明

[Show Card 出示卡片 14]

EH006 What insurance did you use? (circle all that apply) 您使用了哪种医疗保险报销？（可多选）

1. Urban employee medical insurance (yi-bao) 城镇职工医疗保险（医保）
2. Urban resident medical insurance 城镇居民医疗保险
3. New cooperative medical insurance (he-zuo-yi-liao) 新型农村合作医疗保险（合作医疗）
4. Urban and rural resident medical insurance 城乡居民医疗保险（合并城镇居民和新型农村合作医疗保险）
5. Government medical insurance (gong-fei) 公费医疗
6. Medical aid 医疗救助
7. Private medical Insurance: Purchased by R's union 商业医疗保险：单位购买
8. Private medical Insurance: Purchased by Individual 商业医疗保险：个人购买
9. Urban non-employed persons's health insurance 城镇无业居民大病医疗保险
10. Other medical insurance (specify) 其他医疗保险，请注明 ____
11. Reimbursed by R's union 单位报销
12. No insurance 没有保险
13. Not revelent to me. 不适用

F1：（1）"城镇职工医疗保险"指依法对城镇职工的基本医疗权利给予保障的社会医疗保险制度。
(2)"城镇居民医疗保险"指 2007 年 7 月国务院决定在全国 79 个城市实行城镇居民基本医疗保险试点。参保范围：城镇非从业居民，包括：不属于城镇职工基本医疗保险制度覆盖范围的中小学阶段的学生（包括职业高中、中专、技校学生)、少年儿童和其他非从业城镇居民。
(3)"新型农村合作医疗保险"指由政府组织、引导、支持，农民自愿参加，个人、集体和政府多方筹资，以大病统筹为主的农民医疗互助共济制度。采取个人缴费、集体扶持和政府资助的方式筹集资金。
(4)"城乡居民医疗保险"指一些地区率先将城镇居民医疗保险与新型农村合作医疗合并，推行统一的城乡居民医疗保险制度。
(5)"外来务工人员医疗保险"指在某些地区在城镇职工基本医疗保险以外，专为外来务工人员设置的一种医疗保险制度。
(6)"公费医疗"指国家为保障国家工作人员身体健康而实行的一项社会保障制度。其享受对象主要是各级国家机关、党派、团体以及文化、教育、科研、卫生、体育等事业单位的工作人员和离退休人员，二等乙级以上革命伤残军人以及在校大学生等。

(7)“医疗救助”指在政府的支持下，依靠社会力量建立的旨在向困难群体提供某些或全部基本医疗服务的制度。
(8)“商业医疗保险”是相对于社会保险而言的。商业医疗保险由商业保险公司经办，以营利为目的，企业或职工自愿参加。
(9)“其他医疗保险”如中国职工保险互助会《在职职工重大疾病互助保障计划》和《生育保险》。
(10)“医疗救助”在部分区县试点，救助标准是由各区县根据自身经济情况制定，导致救助标准有高有低。主要三种，一是提供社会医疗救助金，给救助对象以经济补偿；二是给医疗机构一定的经济补贴，使后者直接减免救助对象的部分医疗费；三是由社会医疗救助机构举办专门医疗机构，免费为救助对象提供医疗服务。
(11)“医疗保险”是指基本医疗保险，是为补偿疾病所带来的医疗费用的一种保险。职工因疾病、负伤、生育时，由社会或企业提供必要的医疗服务或物质帮助的社会保险。如中国的公费医疗、劳保医疗。
(12)“单位报销”指一些福利较好的单位可以对本单位职工发生的除基本医疗保险报销部分以外的医疗费用进行全额或一定额度的报销。
(13)城镇无业居民大病医疗保险参保范围是具有本市非农业户籍，男满 16 周岁不满 60 周岁，女满 16 周岁不满 50 周岁，未纳入城镇职工基本医疗保险覆盖范围的居民。筹资标准为每人每年 700 元，其中个人缴纳 600 元、财政补助 100 元。

EF006 How often did the respondent receive assistance in answering section D-Health care and insurance? 受访者填写该部分问卷时是否求助？[IWER: If it is answered by a proxy, the respondent's reaction. 访员注意：如果是协助回答，请记录受访者的反应。]

1. Never 从未
2. A few times 偶尔几次
3. Most or all of the time 大多数
4. The section was completed by a proxy respondent（the respondent is absent）受访者不在场，完全请人代填 → Skip to EF007 请跳至EF007

EF007 What is your relationship to R? 您和受访者是什么关系？[IWER: What is the proxy's relationship to R? If unknown, please ask the proxy. 访员注意：代填问卷的人和受访者是什么关系。如果不清楚，请问代填者。]

1. Spouse 配偶
2. Mother 母亲
3. Father 父亲
4. Mother-in-law 岳母/婆婆
5. Father-in-law 岳父/公公
6. Sibling 兄弟姐妹
7. Brother-in-law, sister-in-law 姐夫妹夫/嫂子弟媳
8. Child 孩子
9. Spouse of child 孩子的配偶
10. Grandchild 孙子女
11. Other relative 其他亲戚

12. Helper or other non-relative 帮忙的人或者其他非亲属

EF008 [IWER: Please record the reason for proxy 访员注意：请记录代答的原因] What is the main reason for proxy（the respondent is absent）? 受访者不在场，完全请人代填的主要原因是什么?

1. The respondent has serious physical handicaps 受访者有严重身体障碍
2. The respondent has serious mental handicaps, 受访者有严重精神障碍
3. The respondent has rejected this interview 受访者拒访
4. Other

F WORK, RETIREMENT AND PENSION 工作、退休和养老金

Type of Interview R 受访者类型		
XRType = REIW	This is a reinterview R	受访者类型为回访受访者
XRType = NEWIW	This is a new interview R	受访者类型为新受访者

FA JOB STATUS

FA001 Did you engage in agricultural work (including farming, forestry, fishing, and husbandry for your own family or others) for more than 10 days in the past year? 过去一年，您有没有从事 10 天以上的农业生产经营活动（包括为自家或其他农户种地、管理果树、采集农林产品、养鱼、打鱼、养牲畜以及去市场销售自家生产的农产品等）？

1. Yes 是 → NEWIW skips to FB001; REIW skips to FA006_w2_1 BRANCHPOINT 新受访者跳至FB001；回访受访者跳至FA006_w2_1 BRANCHPOINT
2. No 否

FA002 Did you work for at least one hour last week? We consider any of the following activities to be work: earn a wage, run your own business and unpaid family business work, et. al. Work does not include doing your own housework or doing activities without pay, such as voluntary work. 上周您工作了至少一个小时吗？挣工资工作、从事个体、私营经济活动或不拿工资为家庭经营活动帮工都算是工作，但不包括家务劳动、义务的志愿劳动。

1. Yes 是 → NEWIW skips to FB001; REIW skips to FA006_w2_1 BRANCHPOINT 新受访者跳至FB001; 回访受访者跳至FA006_w2_1 BRANCHPOINT
2. No 否

FA003 Do you have a job but are temporarily laid-off, or on sick or other leave, or in-job training? 您是不是有工作但是目前正处在临时放假、休病假，或其他假期中，或者正在在职培训？

1. Yes 是
2. No 否 → NEWIW skips to FA007; REIW skips to FA006_w2_1 BRANCHPOINT 新受访者跳至FA007; 回访受访者跳至FA006_w2_1 BRANCHPOINT

FA004 In what month and year did you leave or attend training? 您是什么时候开始休假或参加培训的？

____ 1900...2013 (**FA004_1**) year 年 __0...12 (**FA004_2**) month 月

[IWER: Mark the year using four digits. Take down the month as its actual number. For example, write January as “1” not “01”, December as “12”. If do not remember

month, fill '0'. 访员注意：用 4 位数表示年，按照实际的月份填写月。例：1 月写作"1"，而不是"01"，12 月写作"12"。如果记不住月份，请填入'0'。]

FA005 Do you expect to go back to this job at a definite time in the future or within 6 months? 您能够在确定的时间或者 6 个月以内，回到原来的工作岗位么？

1. Yes 是 → NEWIW skips to FB001; REIW skips to FA006_w2_1 BRANCHPOINT 新受访者跳至FB001; 回访受访者跳至FA006_w2_1 BRANCHPOINT
2. No 否

FA006 Do you still receive any salary from this job? 这个单位是否仍然给你发工资?

1. Yes 是 → NEWIW skips to FB001; REIW skips to FA006_w2_1 BRANCHPOINT 新受访者跳至FB001; 回访受访者跳至FA006_w2_1 BRANCHPOINT
2. No 否 → NEWIW skips to FA007; REIW skips to FA006_w2_1 BRANCHPOINT 新受访者跳至FA007; 回访受访者跳至FA006_w2_1 BRANCHPOINT

FA006_w2_1 BRANCHPOINT:

For REIW doing agricultuarl work (FA001= 1): If he/she was only doing agricultural work in the last IW (ZF1= 1), SKIP TO FA006_w2_5; If he/she was only doing non-agricultural work (ZF1= 2) or not doing any work in the last IW (ZF1= 4), SKIP TO FA006_w2_2; If he/she was doing both agricultural and non-agricultural work in the last IW(ZF1= 3), skip to FA006_w2_2a Branchpoint.

For REIW not doing agricultural work but doing non-agricultural work (FA001= 2 & FA002= 1): If he/she was only doing agricultural work in the last IW (ZF1= 1) or doing both agricultural and non-agricultural work (ZF1 = 3) in the last IW, skip to FA006_w2_1; If he/she was only doing non-agricultural work in the last IW (ZF1= 2), skip to FA006_w2_2a Branchpoint; If he/she was not doing any work in the last IW (ZF1= 4), SKIP TO FA006_w2_5;

For REIW not doing any work(XF1= 2): If he/she was only doing agricultural work in the last IW (ZF1= 1), or doing both agricultural and non-agricultural work in the last IW (ZF1= 3), skip to FA006_w2_1; If he/she was only doing non-agricultural work in the last IW (ZF1= 2), skip to FA006_w2_2a Branchpoint; If he/she was not doing any work in the last IW (ZF1= 4), skip to FA006_w2_5.

FA006_w2_1 You told us that you were doing agricultural work (ZF1= 1 or ZF1= 3) in the last IW, when did you stop doing it? 上一轮调查时 [ZIWTime] 您告诉我们您从事农业工作（ZF1= 1 或 ZF1= 3），您是什么时候停止农业工作的？

1. ____ (**FA006_w2_1_1**) year 年 ____ (**FA006_w2_1_2**) month 月
2. I was not doing agricultural work in the last IW 上一轮时我没有从事农业工作 → Skip to FA006_w2_1a Branchpoint 跳至FA006_w2_1a Branchpoint

FA006_w2_1a What was the reason for you to stop doing this job? 您停止做这份工作的原因是什么？

1. Business closed 单位破产/倒闭了

2. Quit 辞职
3. I was laid off 下岗
4. I was fired 被辞退
5. I went to school 我上学了
6. I went abroad 我出国了
7. I stopped working for health reasons 因为健康原因中断工作
8. I stopped working for family reasons 因为家庭原因中断工作
9. I was transferred to another job 工作调动
10. I started working off-farm locally 我在本地从事非农工作了
11. I went to work away from home 我外出打工了
12. Better job in local area 本地更好的新工作
13. Better job in another location 外地更好的新工作
14. I retired 我退休了
15. Other 其他

FA006_w2_1a BRANCHPOINT:

IF XRType = REIW AND HE/SHE was only doing agricultural work IN THE LAST WAVE, THEN SKIP TO FA006_w2_5.

IF XRType = REIW AND HE/SHE was doing both agricultural and non-agricultural work IN THE LAST WAVE, THEN SKIP TO FA006_w2_2a Branchpoint.

FA006_w2_2 You told us that you were not doing agricultural work in the last IW (ZF1= 2 or ZF1= 4), when did you starting doing agricultural work? 上一轮时 [ZIWTime] 您告诉我们您没有从事农业工作（ZF1= 2 或 ZF1= 4），您是什么时候开始从事农业工作的？

1. ____ (**FA006_w2_2_1**) year 年 ____ (**FA006_w2_2_2**) month 月
2. I was doing agriclutural work in the last IW 上一轮时我就从事农业工作

PROCEDURE 程序:

If XRType = REIW and he/she was only doing non-agricultural work in the last IW (ZF1= 2), skip to FA006_w2_2a Branchpoint;

If XRType = REIW and he/she was not doing any work in the last IW (ZF1= 4), skip to FA006_w2_5;

FA006_w2_2a BRANCHPOINT:

For old R who was doing employed work in the last IW (ZF5= 1): If the name of the employer is not missing in the last IW (ZF8≠ .), skip to FA006_w2_2a; If the name of the employer is missing in the last IW (ZF8= .), skip to FA006_w2_2a_2.

For old R who was doing self-employed work in the last IW (ZF6= 1): If the name of the company/business is not missing in the last IW (ZF9≠ .), skip to FA006_w2_2b; If the name of the company/business is missing in the last IW(ZF9= .), skip to FA006_w2_2b_2.

For old R who was working for unpaid family business (ZF7= 1): If the name of the family business is not missing in the last IW (ZF10≠ .), skip to FA006_w2_2c. If the name of the family business is missing in the last IW (ZF10= .), skip to FA006_w2_2c_2.

FA006_w2_2a You told us that you were working for[Preload the name of the employer from the last IW(ZF8)] in the last IW, is the name correct? 您告诉我们上一轮调查时 [ZIWTime] 您为 [下载上一轮调查时的雇主名称（ZF8）] 工作，这个名称正确吗？

1. Yes 是 → Skip to FA006_w2_2a_3 跳至FA006_w2_2a_3
2. No 否

[访员注意：如果受访者回答说上一轮不从事受雇（为别人）工作，请选择“(2) 否”]

FA006_w2_2a_1 What is the correct name? 请问正确的名称是？

[IWER: Write the name of the household head if R works for a family. 访员注意：如果雇主是家庭，工作单位请写户主的姓名。]

1. Fill in the employer's name 填写雇主名称 __________(**FA006_w2_2a_1_1**)（例 1：北京大学餐饮服务中心；例 2：北京华信服装服饰有限责任公司）Name of the department 所在部门名称：__________ （例 1：农园餐厅；例 2：纺纱生产车间）→ Skip to FA006_w2_2a_3 跳至FA006_w2_2a_3
2. Last IW, you ran your own business, fill in the firm's name 上一轮不从事受雇，而是自己开公司/做生意，请填写公司/生意名称 __________(**FA006_w2_2a_1_2**)（例 1：北京华信服装服饰有限责任公司；例 2：丽丽服装店）→ Skip to FA006_w2_5 跳至FA006_w2_5
3. Last IW, you were not employed and did work for family business without being paid, fill in the firm's name 上一轮不从事受雇，而是为家庭经营活动无偿帮工，请填写帮工的公司/生意名称 __________(**FA006_w2_2a_1_3**)（例 1：北京华信服装服饰有限责任公司；例 2：丽丽服装店）→ Skip to FA006_w2_5 跳至FA006_w2_5
4. Last IW, you were not employed and did farm work 上一轮不从事受雇，而是务农 → Skip to FA006_w2_5 跳至FA006_w2_5
5. Last IW, you did not work 上一轮不工作 → Skip to FA006_w2_5 跳至FA006_w2_5

FA006_w2_2a_2 You told us that you were working for someone else in the last IW, but you did not tell us the name of the employer in the last IW, what is the name of the employer you were working for in the last IW? 上一轮调查时 [ZIWTime] 您告诉我们您为其他人工作，但是没有告诉我们雇主的名称，请问您当时的雇主名称是？

[IWER: Write the name of the household head if R works for a family. 访员注意：如果雇主是家庭，工作单位请写户主的姓名。]

1. Fill in the employer's name 填写雇主名称 __________(**FA006_w2_2a_2_1**)（例 1：北京大学餐饮服务中心；例 2：北京华信服装服饰有限责任公司）Name of the department 所在部门名称：__________ （例 1：农园餐厅；例 2：纺纱生产车间）
2. Last IW, you ran your own business, fill in the firm's name 上一轮不从事受雇，而是自己开公司/做生意，请填写公司/生意名称 __________(**FA006_w2_2a_2_2**)（例 1：北京华信服装服饰有限责任公司；例 2：丽丽服装店）→ Skip to FA006_w2_5 跳至FA006_w2_5
3. Last IW, you were not employed and did work for family business without being paid, fill in the firm's name 上一轮不从事受雇，而是为家庭经营活动无偿帮工，请填写帮工的公司/生意名称 __________(**FA006_w2_2a_2_3**)（例 1：北京华信服装服饰有限责任公司；例 2：丽丽服装店）→ Skip to FA006_w2_5 跳至FA006_w2_5

4. Last IW, you were not employed and did farm work 上一轮不从事受雇，而是务农 → Skip to FA006_w2_5 跳至FA006_w2_5
5. Last IW, you did not work 上一轮不工作 → Skip to FA006_w2_5 跳至FA006_w2_5

FA006_w2_2a_3 Are you still working for this employer? 您还在为这个雇主工作吗？
1. Yes 是 → Skip to FA006_w2_5 跳至FA006_w2_5
2. No 否 → Skip to FA006_w2_3 跳至FA006_w2_3

FA006_w2_2b You told us that you were running ________ [Preload the name of the company/workplace from the last IW (ZF9), is the name correct? 上一轮调查时 [ZIWTime] 您告诉我们您开/做 [下载上一轮调查时公司/生意的名称（ZF9）] 公司/生意，这个名称正确吗？
1. Yes 是 → Skip to FA006_w2_2b_3 跳至FA006_w2_2b_3
2. No 否

[访员注意：如果受访者回答说上一轮不从事自雇 (自己开公司/做生意) 工作，请选择“(2) 否”]

FA006_w2_2b_1 What is the correct name? 请问正确的名称是？
1. Fill in the firm's name 填写开的公司/做的生意的名称 __________(**FA006_w2_2b_1_1**)（例 1：北京华信服装服饰有限责任公司；例 2：丽丽服装店）→ Skip to FA006_w2_2b_3 跳至FA006_w2_2b_3
2. Last IW, you were employed, fill in the firm's name 上一轮不从事自雇，而是受雇，请填写受雇的公司/生意名称 __________(**FA006_w2_2b_1_2**)（例 1：北京大学餐饮服务中心；例 2：北京华信服装服饰有限责任公司）Name of the department 所在部门名称：_____________（例 1：农园餐厅；例 2：纺纱生产车间）→ Skip to FA006_w2_5 跳至FA006_w2_5
3. Last IW, you were not self-employed and did work for family business without being paid, fill in the firm' s name 上一轮不从事自雇，而是为家庭经营活动无偿帮工，请填写帮工的公司/生意名称 _____________(**FA006_w2_2b_1_3**)（例 1：北京华信服装服饰有限责任公司；例 2：丽丽服装店）→ Skip to FA006_w2_5 跳至FA006_w2_5
4. Last IW, you were not self-employed and did farm work 上一轮不从事自雇，而是务农 → Skip to FA006_w2_5 跳至FA006_w2_5
5. Last IW, you did not work 上一轮不工作 → Skip to FA006_w2_5 跳至FA006_w2_5

FA006_w2_2b_2 You told us that you were running a company/business in the last IW, but you did not tell us the name of the company/business in the last IW, what is the name of the company/business you were running in the last IW? 上一轮调查时 [ZIWTime] 您告诉我们您在开公司/做生意，但是没有告诉我们公司/生意的名称，请问您当时的公司/生意名称是？
1. Fill in the firm's name 填写开的公司/做的生意的名称 __________(**FA006_w2_2b_2_1**)（例 1：北京华信服装服饰有限责任公司；例 2：丽丽服装店）
2. Last IW, you were employed, fill in the firm's name 上一轮不从事自雇，而是受雇，请填写受雇的公司/生意名称 __________(**FA006_w2_2b_2_2**)（例 1：北京大学餐饮服

务中心；例 2：北京华信服装服饰有限责任公司）Name of the department 所在部门名称：_____________（例 1：农园餐厅；例 2：纺纱生产车间）→ Skip to FA006_w2_5 跳至FA006_w2_5

3. Last IW, you were not self-employed and did work for family business without being paid, fill in the firm's name 上一轮不从事自雇，而是为家庭经营活动无偿帮工，请填写帮工的公司/生意名称 _____________(**FA006_w2_2b_2_3**)（例 1：北京华信服装服饰有限责任公司；例 2：丽丽服装店）→ Skip to FA006_w2_5 跳至FA006_w2_5
4. Last IW, you were not self-employed and did farm work 上一轮不从事自雇，而是务农 → Skip to FA006_w2_5 跳至FA006_w2_5
5. Last IW, you did not work 上一轮不工作 → Skip to FA006_w2_5 跳至FA006_w2_5

FA006_w2_2b_3 Are you still running this company/business? 您还在开这个公司/做这项生意吗？

1. Yes 是 → Skip to FA006_w2_5 跳至FA006_w2_5
2. No 否 → Skip to FA006_w2_3 跳至FA006_w2_3

FA006_w2_2c You told us that you were working for an unpaid family business [Preload the name of the unpaid family business from the last IW (ZF10)], is the name correct? 上一轮调查时 [ZIWTime] 您告诉我们您不拿工资为家庭经营活动 [下载上一轮无偿帮工家庭经营活动的名称（ZF10）] 帮工，这个名称正确吗？

1. Yes 是 → Skip to FA006_w2_2c_3 跳至FA006_w2_2c_3
2. No 否

[访员注意：如果受访者回答说上一轮不是为家庭经营活动无偿帮工，请选择“(2) 否”]

FA006_w2_2c_1 What is the correct name? 请问正确的名称是？

1. Fill in the firm's name 填写帮工的公司/生意的名称 __________(**FA006_w2_2c_1_1**)（例 1：北京华信服装服饰有限责任公司；例 2：丽丽服装店）→ Skip to FA006_w2_2c_3 跳至FA006_w2_2c_3
2. Last IW, you were employed, fill in the firm's name 上一轮不从事无偿帮工，而是受雇，请填写受雇的公司/生意名称 __________(**FA006_w2_2c_1_2**)（例 1：北京大学餐饮服务中心；例 2：北京华信服装服饰有限责任公司）Name of the department 所在部门名称：____________（例 1：农园餐厅；例 2：纺纱生产车间）→ Skip to FA006_w2_5 跳至FA006_w2_5
3. Last IW, you ran your own business, fill in the firm's name 上一轮不从事无偿帮工，而是自己开公司/做生意，请填写公司/生意名称 ____(**FA006_w2_2c_1_3**)（例 1：北京华信服装服饰有限责任公司；例 2：丽丽服装店）→ Skip to FA006_w2_5 跳至FA006_w2_5
4. 上一轮不从事无偿帮工，而是务农 → Skip to FA006_w2_5 跳至FA006_w2_5
5. 上一轮不工作 → Skip to FA006_w2_5 跳至FA006_w2_5

FA006_w2_2c_2 You told us that you were working for an family business without a wage in the last IW, but you did not tell us the name of the name of the family business,

what is the name of this family business you were working for without a wage in the last IW? 上一轮调查时 [ZIWTime] 您告诉我们您不拿工资为一项家庭经营活动帮工，但是没有告诉我们这项家庭经营活动的名称，请问您上一轮调查时不拿工资帮工的家庭经营活动的名称是？

1. Fill in the firm's name 填写帮工的公司/生意的名称 ________**(FA006_w2_2c_2_1)** （例 1：北京华信服装服饰有限责任公司；例 2：丽丽服装店）
2. Last IW, you were employed, fill in the firm's name 上一轮不从事无偿帮工，而是受雇，请填写受雇的公司/生意名称 ________**(FA006_w2_2c_2_2)** （例 1：北京大学餐饮服务中心；例 2：北京华信服装服饰有限责任公司）Name of the department 所在部门名称：________ （例 1：农园餐厅；例 2：纺纱生产车间）→ Skip to FA006_w2_5 跳至FA006_w2_5
3. Last IW, you ran your own business, fill in the firm's name 上一轮不从事无偿帮工，而是自己开公司/做生意，请填写公司/生意名称 ___**(FA006_w2_2c_2_3)** （例 1：北京华信服装服饰有限责任公司；例 2：丽丽服装店）→ Skip to FA006_w2_5 跳至FA006_w2_5
4. 上一轮不从事无偿帮工，而是务农 → Skip to FA006_w2_5 跳至FA006_w2_5
5. 上一轮不工作 → Skip to FA006_w2_5 跳至FA006_w2_5

FA006_w2_2c_3 Are you still working for this family business? 您还在为这项家庭经营活动帮工吗？

1. Yes 是 → Skip to FA006_w2_5 跳至FA006_w2_5
2. No 否 → Skip to FA006_w2_3 跳至FA006_w2_3

FA006_w2_3 When did you stop doing this job? 您是什么时候停止做这份工作的？ ___ **(FA006_w2_3_1**) year 年 ___ **(FA006_w2_3_2)** month 月

FA006_w2_4 What was the reason for you to stop doing this job? 您停止做这份工作的原因是什么？

1. Business closed 单位破产／倒闭了
2. Quit 辞职
3. I was laid off 下岗
4. I was fired 被辞退
5. I went to school 我上学了
6. I went abroad 我出国了
7. I stopped working for health reasons 因为健康原因中断工作
8. I stopped working for family reasons 因为家庭原因中断工作
9. I was transferred to another job 工作调动
10. I started working off-farm locally 我在本地从事非农工作了
11. I went to work away from home 我外出打工了
12. Better job in local area 本地更好的新工作
13. Better job in another location 外地更好的新工作
14. I retired 我退休了

15. Other 其他

FA006_w2_5 Starting from last IW 从上一轮调查 [ZIWTime] to the current IW 到现在，how many other jobs have you been doing? 您从事过几项其他工作？(Note: the work is defined as every work you had been working for at least one month, excluding the work you were doing in the last IW and the work you are doing in the current IW 注意：这里的工作指的是从事至少一个月的任何工作，不包括上一轮调查时的工作和现在从事的工作) ____ Skip to FA007 Branchpoint if the answer is "0" 如果回答是“0”，跳至FA007 Branchpoint

FA006_w2_6 How many months were you working on these jobs? 您在这些工作上干了几个月 __Months 月？

FA006_w2_7 While you were doing these jobs, how many weeks were you working on average per month? 您在做这些工作时，平均每个月工作几周 ___Weeks 周

FA006_w2_8 While you were doing these jobs, how many days were you working on average per week? 您在做这些工作时，平均每周工作几天？_Days 天

FA006_w2_9 While you were doing these jobs, how many hours were you working on average per day? 您在做这些工作时，平均每天工作几个小时？__Hours 小时

FA006_w2_10 While you were doing these jobs, what is the average income you earned per month (excluding income tax and insurance payment)? 您在做这些工作时，每个月平均能挣多少钱（扣除个人所得税和上缴的保险）______Yuan 元.

FA007 BRANCHPOINT:

If old R answered work history questions in the last IW (ZF12= 1), then skip to FB011 如果老受访者在上一轮调查中回答了工作史问题（ZF12= 1），请跳至FB011。

If old R did not answer work history questions in the last IW(ZF12≠ 1), and is working now(XF1= 1), then skip to FB001 如果老受访者上一轮调查时没有回答工作史问题（ZF12≠ 1），同时现在正在工作（XF1= 1），请跳至FB001。

If old R did not answer work histroy questions in the last IW(ZF12≠ 1), and was working in the last IW(ZF13= 1), then skip to FB001 如果老受访者上一轮调查时没有回答工作史问题 (ZF12≠ 1)，同时上一轮调查时有工作 (ZF13= 1)，请跳至FB001。

If old R had not worked prior to the last IW (ZF14= 1), and is not working now(XF1= 2), then skip to FA007 如果老受访者上一轮调查之前从来没有工作过 (ZF14= 1)，同时现在没有工作 (XF1= 2)，请跳至FA007。

FA007 Have you worked for at least three months during your lifetime (work includes agricultural work,earning wage work, self-employed activities, and unpaid family business work, et. al.)? 您这辈子是否曾经工作了至少三个月的时间？(工作包括务农、挣工资工作、从事个体、私营活动或不拿工资为家庭经营活动帮工等)

1. Yes 是 → Skip to FB001 请跳至FB001
2. No 否

FA008 Work includes all kinds of labour excluding doing your own housework, whether you earn wages or not. Are you sure that you didn’t work at least three months during your lifetime? 我们所说的工作指所有除家务劳动以外的所有干活的劳动，不管是不是有工资。您确定这辈子没有工作过至少三个月的时间？

1. Yes, never worked before 是的，从未工作过
2. No, ever worked. 不是，以前工作过 → Skip to FB001 请跳至FB001

FA009 What is the main reason for you not to work in your lifetime? 请问您不工作的主要原因是什么？

1. Disabled (physical or psychological) 残疾（包括生理和心理上的残疾）
2. Homemaker 操持家务
3. My family is too rich that I don’t need to work 我家太有钱了，我不需要工作
4. Taking care of siblings 照看兄弟姐妹
5. Other 其它

PROCEDURE 程序:
Skip to FN001_w2 请跳至FN001_w2

FB WORK HISTORY 工作史

FB001 At what age (or in which year) did you start working (or farming), excluding part-time job during school time? 您从多少岁或者哪年开始工作 (或者务农) 的（不包括上学期间的兼职工作）？Age 年龄 ____1···120 (**FB001_1**)Years 岁 or 或 ____1900...2013 (**FB001_2**) Year 年

[IWER: Mark the year in four digits. 访员注意：用 4 位数表示年。]

FB002 What type of work unit was your first job, A government organization, “shiye danwei,” firm, NGO, inidivdual farmer, or household enterprise? 您第一份工作的工作单位属于以下哪一种？

1. Government 政府部门
2. Institutions 事业单位
3. NGO 非营利机构（如社团、协会、学会等）
4. Firm 企业
5. Individual firm 个体户
6. Farmer 农户
7. Individual household 居民户
8. Other 其他

PROCEDURE 程序: If FB002= 4, then ask FB003, otherwise go on to FB004 . 如果FB002=4, 请提问FB003，否则请跳至FB004

[Show Card 出示卡片 20]

FB003 What is the ownership type of the business? 您单位的所有制类型是?

1. 100% State owned firm 国有企业
2. State-controlled firm 国有控股企业
3. 100% Collective-owned firm 集体所有制企业
4. Collective-controlled firm 集体控股企业
5. 100% Private firm 私营/个体
6. Private-controlled firm 私人控股企业
7. 100% foreign-owned 外商独资
8. Joint venture 中外合资
9. Other joint-ownership 其他联营企业
10. Other 其他

FB004 Where was your first workplace located? 您第一份工作的地点在哪里?

1. The same as permanent address 和目前常住地一样
2. Another village/neighborhood in permanent address's county/city/district 常住地所在县/市/区的其他村/社区 ____(**FB004_1**)village/neighborhood 村/社区
3. Other 其它: ____province_city_county/city/district 省 _ 市 _ 县/市/区, ____(**FB004_3**) village/neighborhood 村/社区
4. Abroad 国外

[IWER: 访员注意：如果记不清详细的地址名称，请在相应输入框中填入“其它”。]

**PROCEDURE 程序**: If FB002= 4 and FB003= 1/2/3/4, then skip FB005 and FB006 如果FB002= 4 而且 FB003= 1/2/3/4, 跳过 FB005 和FB006

FB005 Over your career did you ever have employment in a state owned, state controlled or collectively owned enterprise? 您一生中有没有在国有企业、国有控股企业、集体所有制企业或集体控股企业工作过?

1. Yes 有
2. No 没有 → go to FB007 跳至 FB007

FB006 Specific ownership type 请问是在哪种类型的企业工作过?

1. 100% State owned firm 国有企业
2. State-controlled firm 国有控股企业
3. 100% Collective-owned firm 集体所有制企业
4. Collective-controlled firm 集体控股企业

FB007 Not counting current non-employment or retirement, did you stop working for an extended period of time (more than 1 year once) due to reasons of family, health, school, etc.? 您是否曾经因为家庭、健康、上学等原因中断过工作（一次中断时间在 1 年以上），服兵役、灵活就业、退休后不工作或者目前失业的时间不算?

1. Yes 是
2. No 否 → skip to FB011 跳至 FB011

FB008 How long were the interruptions in all? 一共中断了多长时间? ____1...120(**FB008_1**) Years 年 ____0...11(**FB008_2**) Months 月

[IWER: Mark the month and year using integert; if 0, please fill '0'. 访员注意：用整数表示年月，如果刚好是整年，月份处填 "0"。]

FB009 When was the longest time period that you stopped working? 最长的一次中断是从什么时候到什么时候? From 从 ____1900...2013 (**FB009_1**) year 年 ____0...12 (**FB009_2**) month 月 to 到 ____1900...2013 (**FB009_3**) year 年 ____0...12 (**FB009_4**) month 月

[IWER: Mark the year using four digits. Take down the month as its actual number. For example, write January as "1" not "01", December as "12". If do not remember month, fill '0'. 访员注意: 用 4 位数表示年，按照实际的月份填写月。例：1 月写作 "1"，而不是 "01" ,12 月写作 "12"。如果记不住月份，请填入' 0'。]

[Soft Check: Reprompt for FB008 and FB009 if longest time period is greater than Total from FB008 or (FB009_3 + FB009_4/12)-(FB009_1 + FB009_2/12) > FB008_1 + FB008_2/12.]

[Another check to avoid that interruption began before starting work. FB009_1 < FB001_1 or FB009_1 < CV009_a + FB001_1]

FB010 The reason was: 中断工作的原因是:

1. Family 家庭（例如照顾家人，结婚生子）
2. Health 健康（例如生病）
3. School 上学
4. Unemployment/layoff 失业/下岗
5. Other 其他

FB011 BRANCHPOINT:

If old R had completed retirement procedure (including early retirement) or internal retirement (ZF15= 1), skip FB011 and FB012 如果老受访者已经办理了退休（包括提前退休）或者内退（ZF15= 1），跳过FB011 和FB012

FB011 Have you completed retirement procedures（including early retirement）or internal retirement（Note: Retirement from government departments, enterprises and institutions, not including retirement in the sense of getting agricultural insurance）? 您是否办理了退休手续（包括提前退休）或内退（注意：退休指从机关、事业单位、企业的退休，不包括获取农保的退休）?

1. Yes 是 → skip to FB012 跳至 FB012
2. No 否

FB012 Have you completed receding position procedures? 您是否办理了退职手续?

1. Yes 是
2. No 否

F1："退职"指职工因病残完全丧失劳动能力，但在年龄、工龄或个人缴费年限方面又不具备退休条件的，经医院证明并经劳动鉴定委员会确认、组织批准后退出生产或工作岗位，并按国家有关规定给予一定的物质帮助和被补偿，进行休养。或者是已到退休年龄，但工龄不够。

FB012 BRANCHPOIN:

If new R is not working but has worked before (FA007= 1 or FA008= 2), skip to FK001. 如果新受访者现在没有工作但是曾经工作过（FA007= 1 或FA008= 2)），跳至FK001.

If old R is not working (XF1= 2) but worked before (FA006_w2_5> 0 or ZF13= 1 or ZF14= 2), skip to FK001. 如果老受访者现在没有工作（XF1= 2）但是曾经工作过（FA006_w2_5> 0 或 ZF13= 1 或 ZF14= 2），跳至FK001

FC CURRENT PRIMARY JOB/OCCUPATION 当前、主要工作

FC001 BRANCHPOINT]:

If R (including new R and old R) did farming last year (FA001= 1), ask FC001 如果受访者（包括新受访者和老受访者）过去一年从事过农业工作（FA001= 1 ），询问FC001

if R did not do farming last year, but did non-agricultural work last week (XF2= 1), ask FC019. 如果受访者过去一年没有从事农业工作，但是上周从事过非农工作（XF2= 1），询问FC019.

FARM EMPLOYED 农业打工

FC001 Did you work for other famers in wage for at least ten days in the past year?（Agricultural work in wages）过去一年，您有没有为其他农户打工至少 10 天？（指从事农业劳动挣钱）

1. Yes 是
2. No 否 → skip to FC008 跳至 FC008

F1：“为其他农户打工”指为其他农户从事农业生产劳动挣钱。

FC002 How many employers did you work for in the past month? 过去一个月，您一共为多少个农户打工？ ____

FC003 Where is your workplace for most time? 大多数时间里，您工作的地点是？

1. The same as permanent address 和目前常住地一样
2. Another village/neighborhood in permanent address's county/city/district 常住地所在县/市/区的其他村/社区 ____(**FC003_1**) village/neighborhood 村/社区
3. Other 其它： ____(**FC003_2**) province_city_county/city/district 省 _ 市 _ 县/市/区，____(**FC003_3**) village/neighborhood 村/社区
4. Abroad 国外

[IWER: 访员注意：如果记不清详细的地址名称，请在相应输入框中填入“其它”。]

FC004 How many months did you work on cropping (forestry), livestock, and fishing in wage for other famers in the past year? 过去一年中，您有几个月在为其他农户从事 [种植业及林业（包括种地、管理果树、采集农林产品）] 或 [畜牧业 (饲养牲畜)] 或 [渔业（养殖水产品）]? ____0...12 Months 月？

FC005 How many days did you work in wage for other famers per week on average during a normal work month in the past year? 过去一年中，在您为其他农户从事 [种植业及林业

(包括种地、管理果树、采集农林产品)] 或 [畜牧业 (饲养牲畜)] 或 [渔业 (养殖水产品)] 的月份里，您一般每周干几天？ ____0...7 Days 天

FC006 How many hours did you usually work in wage for other famers per day during a normal work day in the past year? 过去一年中，在您为其他农户从事 [种植业及林业 (包括种地、管理果树、采集农林产品)] 或 [畜牧业 (饲养牲畜)] 或 [渔业 (养殖水产品)] 的日子里，您一般每天要干几个小时？ ____0...16 Hours 小时
[Soft Check: Verify if number of hours per day is unreasonable, e.g., FC006 >16]

FC007 What is the average monthly wage did you get in your working months in the past year? 过去一年里，您工作的月份里平均每个月挣多少钱？ ____Yuan 元

HOUSEHOLD AGRICULTURAL WORK 自家农业生产活动

FC008 Did you work for your own household for at least ten days in the past year? 过去一年，您有没有为自家从事农业生产经营活动至少 10 天？
1. Yes 是
2. No 否 → skip to FC013 跳至 FC013

FC009 How many months did you work on [cropping (forestry), livestock, and fishing] for your own householdin the past year? 过去一年中，您有几个月为自家从事 [种植业及林业 (包括种地、管理果树、采集农林产品)] 或 [畜牧业 (饲养牲畜)] 或 [渔业 (养殖水产品)]? ____ 0...12 Months 月

FC010 How many days did you work for your own household per week on average during a normal work month in the past year? 过去一年中，在您为自家从事 [种植业及林业 (包括种地、管理果树、采集农林产品)] 或 [畜牧业 (饲养牲畜)] 或 [渔业 (养殖水产品)] 的月份里，您一般每周干几天？ ____ 0...7 Days 天

FC011 How many hours did you usually work for your own household per day during a normal work day in the past year? 过去一年中，在您为自家从事 [种植业及林业 (包括种地、管理果树、采集农林产品)] 或 [畜牧业 (饲养牲畜)] 或 [渔业 (养殖水产品)] 的日子里，您一般每天要干几个小时？ __ 0...24 Hours 小时
[A check similar to the one for FC006. FC011 > 16 is unreasonable]

FC012 Where is your workplace for most time? 大多数时间里，您工作的地点是？ [preload sampling community ID]
1. The same as permanent address 和目前常住地一样
2. Another village/neighborhood in permanent address's county/city/district 常住地所在县/市/区的其他村/社区 ____(**FC012_1**) village/neighborhood 村/社区
3. Other 其它： ____(**FC012_2**) province_city_county/city/district 省 _ 市 _ 县/市/区， _____ (**FC012_3**) village/neighborhood 村/社区
4. Abroad 国外

[IWER: 访员注意：如果记不清详细的地址名称，请在相应输入框中填入“其它”。]

FC013 How many days of work did you miss last year due to health problems? 过去一年您有多少天因为健康原因干不了活？ ____0...366 Days 天
[IWER: Mark 0 if you didn't miss any work days. 访员注意：如果没有，请注明“0”。]

FC014 Besides agricultural work, did you work for at least one hour last week in wage or self-employed work or umpaid family business? 除农业生产经营活动外，上周您有没有工作（包括挣工资工作、从事个体、私营活动或不拿工资为家庭经营活动帮工等）至少一个小时？

1. Yes 是 → skip to FC019 跳至 FC019
2. No 否

FC015 Do you have wage or self-employed work but are temporarily laid-off or are on sick, seasonal, or other leave or in-job training? 您是否有其他工作（包括挣工资工作、从事个体、私营活动或不拿工资为家庭经营活动帮工等），但是目前正暂时放假、由于生病或者季节原因休假、歇业或者处于其他假期中或正在在职培训？

1. Yes 是 → skip to FC018 跳至 FC018
2. No 否

FC016 In what month and year did leave or attend training? 您是什么时候开始休假/歇业/在职培训的？ ____1900...2013 (**FC016_1**) Year 年 __0...12 (**FC016_2**) Month 月
[IWER: Mark the year using four digits. Take down the month as its actual number. For example, write January as “1” not “01”, December as “12”. If do not remember month, fill ‘0’. 访员注意：用 4 位数表示年，按照实际的月份填写月。例：1 月写作“1”，而不是“01”，12 月写作“12”。如果记不住月份，请填入’0’。]
[Same check as the one for FA004 . (Interview Year –FC016_1 + (Interview month – FC016_2)/12 > 1?]

FC017 Do you expect to go back to this job at a definite time in the future or within 6 months? 您能够在确定的时间或者 6 个月以内，回到原来的工作岗位么？

1. Yes 是 → skip to FC019 跳至 FC019
2. No 否

FC018 At what age do you plan to stop working? Stopping work in this context shall refer to having stopped all income-related activities, unpaid family business and having no intention of engaging in anything more serious than small pastime work. 您计划在多大年龄时停止工作，即停止从事一切以挣钱为目的的活动，也不再为家庭经营活动帮工，将来也不打算从事比消遣性工作更劳累的工作?____ 1⋯120 Years old 岁
[IWER: Please tell me the approximate age. Mark 0 if you plan to keep working until you are physically able. 访员注意：请告知大概年龄。如果您计划只要健康允许，就一直工作，请标明“0”。]
F1：“消遣性工作”指不是以挣钱为目的的活动，以及不再为家庭经营活动帮工的工作。

**FC018 BRANCHPOINT**:
Skip to pension and social security section (FN001_w2) if have not processed retirement and receding (FB011= 2 and FB012= 2) 如果还没有办理退休或退职（FB011= 2 且 FB012= 2），跳至养老保险部分（FN001_w2）<br>Skip to retirement section (FM001) if processed retirement or receding (FB011= 1 and FB012= 1 or processed retirement or receding in the last IW (ZF15= 1 or ZF16= 1) 如果已经办理退休或退职（FB011= 1 或 FB012= 1 或上一轮时办理了退休或退职 (ZF15= 1 or ZF16= 1)，跳至退休和退职模块（FM001）

FC019 Besides agricultural work, do you currently hold more than one non-agricultural job? 除农业生产经营外，您目前有两份（及以上）的非农业工作吗？
[IWER: non-agricultural job includes paid jobs, self-employed activities, unpaid family business work, et. al. Activities without pay, such as voluntary work, are not included. 访员注意：工作包括挣工资工作、从事个体、私营活动或不拿工资为家庭经营活动帮工等，但没有工资的比如志愿者之类的工作不包括在内；此处务农不算工作，即如果一个人除了务农外只有一份工作，此处选 2。]
1. Yes 是 → skip to FC020 跳至 FC020
2. No 否 → skip to FC021 跳至 FC021

FC020 Among all your jobs, which one is your main job? [Main job is defined as the job at which you work the longest hours] Do you earn a wage or do you run your own business or work for unpaid family business? 在这几份工作中，您目前的主要工作 [主要工作的定义：工作时间最长的工作] 是什么？是挣工资工作，从事个体或者私营经济活动，还是不拿工资为家庭经营活动帮工？
1. Employed 挣工资工作 → skip to FD001 跳至 FD001
2. Self-employed 从事个体或者私营经济活动 → skip to FH001 跳至 FH001
3. unpaid family business 不拿工资为家庭经营活动帮工 → skip to FH001 跳至 FH001

FC021 How do you describe your non-agricultural job? Do you earn a wage or do you run your own business or work for unpaid family business? 您怎么描述您现在的工作？是挣工资工作，从事个体或者私营经济活动还是不拿工资为家庭经营活动帮工？
1. Employed 挣工资工作 → skip to FD001 跳至 FD001
2. Self-employed 从事个体或者私营经济活动 → skip to FH001 跳至 FH001
3. unpaid family business 不拿工资为家庭经营活动帮工 → skip to FH001 跳至 FH001

FD EMPLOYED 受雇

FD001 Do you receive wages from your current workplace or receive them from a dispatch/contract company? 您的工资是从工作单位直接拿到还是要通过某个派遣单位拿？
1. Place of work 工作单位
2. Labor dispatch company 劳务派遣单位

F1：“工作单位”是指受访者当前从事劳动的单位。“派遣单位”是指劳动者签订合同，或者人事所在的单位，比如劳务输出公司。

[CAPI: For dispatched/contract workers (FD001= 2), mention the following for questions FD002 -FD016. 对派遣工人 (FD001= 2), 对 FD002-FD016 均提示] The next few questions pertain to the situation at your current workplace, and not to the company that has dispatched/contracted you out. 下面几个问题是关于您为之工作的单位的情况，而不是派遣单位。

FD002 Do you work for a government organization, institution, firm, NGO, individual farmer, or resident household? 您是为政府机构、事业单位、企业单位、非赢利结构（如社团、协会、学会等）、个体户、农户，还是居民户工作？

1. Government 政府部门
2. Institutions 事业单位
3. NGO 非营利机构（如社团、协会、学会等）
4. Firm 企业
5. Individual firm 个体户
6. Farmer 农户
7. Individual household 居民户
8. Other 其他

FD003 BRANCHPOINT:
For old R: if old R is working for the same employer (FA006_w2_2a_3= 1), skip FD003.
老受访者：如果老受访者为同一个雇主工作（FA006_w2_2a_3= 1），跳过FD003。

[INTRO: We will ask your work history later.In order to distinguish the work units, we need the full name and address of your employer. 我们在后面的问卷还会问有关工作史的问题，为了便于区分不同的工作单位，我们需要单位名称。]

FD003 What is the name of your workplace/employer? Please state specifically the name of your company or institution. 您的工作单位或者雇主是什么名称？（请标明详细的单位名称）

工作单位名称：______(**FD003_1**)（例 1：北京大学餐饮服务中心；例 2：北京华信服装服饰有限责任公司）

所在部门名称：________(**FD003_2**)（例 1：农园餐厅；例 2：纺纱生产车间）

[IWER: Write the name of the household head if R works for a family. 访员注意：如果雇主是家庭，工作单位请写户主的姓名。]

FD004 Where is your workplace located? 您工作单位的地点是？(preload sampling community ID)

1. The same as permanent address 和目前常住地一样
2. Another village/neighborhood in permanent address's county/city/district 常住地所在县/市/区的其他村/社区 ____(**FD004_1**) village/neighborhood 村/社区
3. Other 其它：____(**FD004_2**) province_city_county/city/district 省 _ 市 _ 县/市/区，____(**FD004_3**) village/neighborhood 村/社区
4. Abroad 国外

[IWER: 访员注意：如果记不清详细的地址名称，请在相应输入框中填入“其它”。]

PROCEDURE 程序：

If R is working for individual farmer or resident household (FD002= 6, 7), skip to FD011. 如果受访者为农户、居民户工作（FD002= 6, 7），请跳至FD011。

FD005 BRANCHPOINT:

If old R is working for a different employer (FA006_w2_2a_3= 2),skip to FD005 如果老受访者为不同的雇主工作（FA006_w2_2a_3= 2），跳至FD005

If old R is working for the same employer but the information of the type of business is missing in the last IW (FA006_w2_2a_3= 1 & ZF8_1= .), skip to FD005_w2_2. 如果老受访者为同一个雇主工作但是单位的产业或行业类型缺失（FA006_w2_2a_3= 1 & ZF8_1= .），跳至FD005_w2_2

If old R is working for the same employer and the information of the type of business is not missing (FA006_w2_2a_3= 1 & ZF8_1= .), skip to FD005_w2_1. 如果老受访者为同一个雇主工作并且单位的产业或行业类型没有缺失（FA006_w2_2a_3= 1 & ZF8_1= .），跳至FD005_w2_1

New R please answer FD005. 新受访者，回答FD005

FD005 What kind of business or industry do you work in—that is, what does your workplace primarily make or do? 该单位主要是做什么的，也就是，他们制造什么产品或者从事什么活动？ ___________ （例 1：为校内提供餐饮服务；例 2：制造纱质布料）

[IWER: Type of business 访员注意：产业或行业类型。注意：回答中请不要用逗号。]

PROCEDURE 程序：

Skip to FD006. 跳至FD006.

FD005_w2_1 You told us in the last IW that your workplace did or made 上一轮调查时 [ZIWTime] 您告诉我们您的单位是做 [preload information of the type of business of workplace from last IW 下载上一轮调查时单位的产业或行业类型 (ZF8_1)], is the information correct? 是这样的吗？

1. Yes, it is correct 是，信息正确 → skip to FD005_w2_3 跳至 FD005_w2_3
2. No, it is not correct 否，信息不正确

FD005_w2_2 What did your workplace do or make in the last IW? 上一轮调查时 [ZIWTime] 您的单位是做什么？ ___________ 例 1：为校内提供餐饮服务；例 2：制造纱质布料）

[IWER: Type of business 访员注意：产业或行业类型。注意：回答中请不要用逗号。]

FD005_w2_3 Is your workplace still doing or making it? 您的单位现在还是做这个吗？

1. Yes 是 → skip to FD006 跳至 FD006
2. No 否

FD005_w2_4 What is your workplace doing or making now? 您的单位现在做什么？___________ 例 1：为校内提供餐饮服务；例 2：制造纱质布料）

[IWER: Type of business 访员注意：产业或行业类型。注意：回答中请不要用逗号。]

FD005_w2_5 When did your workplace start doing this? 您的单位是从什么时候开始做这个的？
____ (**FD005_w2_5_1**) year 年 ____ (**FD005_w2_5_2**) month 月

PROCEDURE 程序：
If government employee (FD002= 1), ask FD006 to FD007, then skip to FD011. 如果是政府雇员，问FD006 to FD007，然后跳转到 FD011。

FD006 Are you a civil servant? 您是公务员吗？
1. Yes 是
2. No 否

FD007 Are you a formal employee of an establishment? 您是正式编制内的员工吗？
1. Yes 是
2. No 否

PROCEDURE 程序：
If institution (FD002 = 2), ask FD008 to FD009, then skip to FD011. 如果是事业单位，问FD008 to FD009，然后跳转到 FD011。

FD008 Is your institution operated as a firm or as a government unit? 您这个单位是比照企业还是政府机构运行？
1. As a firm 比照企业
2. As a government unit 比照政府机构

FD009 Are you a formal employee of an establishment? 您是正式编制内的员工吗？
1. Yes 是
2. No 否

PROCEDURE 程序：
If firm (FD002= 4), ask from FD010, 如果是企业，从 FD010 开始问

[Show Card 出示卡片 20]

FD010 What is the ownership type of the business? 您单位的所有制类型是？
1. 100% State owned firm 国有企业
2. State-controlled firm 国有控股企业
3. 100% Collective-owned firm 集体所有制企业
4. Collective-controlled firm 集体控股企业
5. 100% Private firm 私营/个体
6. Private-controlled firm 私人控股企业
7. 100% foreign-owned 外商独资
8. Joint venture 中外合资
9. Other joint-ownership 其他联营企业

10. Other 其他

FD011 When did you start working for this employer? 您什么时候开始在这个单位工作的？ ____1900...2013 (**FD011_1**) Year 年 ____0...12 (**FD011_2**) Month 月

[IWER: Mark the year using four digits. Take down the month as its actual number. For example, write January as "1" not "01", December as "12". If do not remember month, fill '0'. 访员注意：用 4 位数表示年，按照实际的月份填写月。例：1 月写作 "1"，而不是 "01" ,12 月写作 "12"。如果记不住月份，请填入' 0'。]

[Soft Check: Consistency of employment start date. Make Sure that the individual was at least a minimum age when he/she started working for this employer (e.g., prompt to check if under 16?). Specifically (FD011_1+FD011_2/12)-(CV009_a+CV009_b/12)<16 prompts a soft check.]

[Replace 16 in the above soft check with FB001. (FD011_1 + FD011_2/12)-(CV009_a + CV009_b/12) < FB001_1 or FD011_1 < FB001_2]

PROCEDURE 程序:

如果受访者上一轮调查时不工作 (ZF1 = 4)，但是本轮 FD011 的回答FD011_1) 和FD011_2早于上一轮的调查时间 [ZIWTime]，询问 FD011_w2_1 和FD011_w2_2

FD011_w2_2 When did you begin working in this unit? 您开始在现在这个单位工作的准确时间是？ ____1900...2013 (**FD011_w2_2_1**) Year 年 ____0...12 (**FD011_w2_2_2**) Month 月

[FD011_w2_2回答的时间需在上一轮调查（ZIWTime）与本轮调查时间之间]

FD012 BRANCHPOINT :

If old R is working for a different employer (FA006_w2_2a_3= 2), skip to FD012 如果老受访者为不同的雇主工作（FA006_w2_2a_3= 2），跳至 FD012

If old R is working for the same employer but the information of the type of business is missing in the last IW (FA006_w2_2a_3= 1 & ZF17= .), skip to FD012_w2_2. 如果老受访者为同一个雇主工作但是单位的产业或行业类型缺失（FA006_w2_2a_3= 1 &ZF17= .），跳至FD012_w2_2

If old R is working for the same employer and the information of the type of business is not missing (FA006_w2_2a_3= 1 & ZF17≠ .), skip to FD012_w2_1. 如果老受访者为同一个雇主工作并且单位的产业或行业类型没有缺失（FA006_w2_2a_3= 1 & ZF17≠ .），跳至FD012_w2_1

New R please answer FD012. 新受访者，回答FD012

FD012 What sort of work do you do? 您具体从事什么工作？ __________(**FD012_1**) 例 1：餐厅面点厨师；例 2：生产线绕线工人）是否有相关的职业资格证书？ __________(**FD012_2**)（例 1：有高级中式烹调师证；例 2：没有职业资格证书）→ Skip to FD013. 跳至FD013

[IWER: Ask about the specific work that R does. 访员注意：请详细填写具体工作内容。注意：回答中请不要用逗号。]

FD012_w2_1 You told us in the last IW that you were doing 上一轮调查时 [ZIWTime] 您告诉我们您在做 [preload information of the work from last IW 下载上一轮调查的工作信息 (ZF17)]，is the information correct? 是这样的吗？

1. Yes, it is correct 是，信息正确 → skip to FD012_w2_3 跳至FD012_w2_3
2. No, it is not correct 否，信息不正确

FD012_w2_2 What were you doing in the last IW? 上一轮调查时 [ZIWTime] 您在做什么？ __________(**FD012_w2_2_1**) 是否有相关的职业资格证书？ __________(**FD012_w2_2_2**)（例 1：有高级中式烹调师证；例 2：没有职业资格证书）
[IWER: Ask about the specific work that R does. 访员注意：请详细填写具体工作内容。注意：回答中请不要用逗号。]

FD012_w2_3 Are you still doing this work? 您还在做这项工作吗？
1. Yes，I am 是，还在做 → skip to FD013 branchpoint 跳至FD013 branchpoint
2. No，I am not 否，不做了

FD012_w2_4 What sort of work are you doing now? 您现在具体从事什么工作？ __________ (**FD012_w2_4_1**)（例 1：餐厅面点厨师；例 2：生产线绕线工人）是否有相关的职业资格证书？ __________(**FD012_w2_4_2**)（例 1：有高级中式烹调师证；例 2：没有职业资格证书）
[IWER: Ask about the specific work that R does. 访员注意：请详细填写具体工作内容。注意：回答中请不要用逗号。]

FD012_w2_5 When did you start doing your current work? 您是从什么时候开始做您现在这个工作的？ ____ (**FD012_w2_5_1**) year 年 ____ (**FD012_w2_5_2**) month 月

FD013 BRANCHPOINT :
If old R is working for a different employer (FA006_w2_2a_3= 2),skip to FD013 如果老受访者为不同的雇主工作（FA006_w2_2a_3= 2），跳至 FD013
If old R is working for the same employer but the information of the type of business is missing in the last IW (FA006_w2_2a_3= 1 & ZF18= .), skip to FD013_w2_2. 如果老受访者为同一个雇主工作但是单位的产业或行业类型缺失（FA006_w2_2a_3= 1 &ZF18= .），跳至FD013_w2_2
If old R is working for the same employer and the information of the type of business is not missing (FA006_w2_2a_3= 1 & ZF18≠ .), skip to FD013_w2_1. 如果老受访者为同一个雇主工作并且单位的产业或行业类型没有缺失（FA006_w2_2a_3= 1 & ZF18≠ .），跳至FD013_w2_1
New R please answer FD013. 新受访者，回答FD013

FD013 What is your current position? 您目前的职务是什么？
1. Clerk/worker 普通职工
2. Team Leader 组长（股长）
3. Section Chief 科长
4. Director of a division 处长
5. Director-General of a bureau and above 局长及以上
6. Village Leader 村干部
7. Township Leader 乡镇干部

8. Division manager 单位部门经理
9. Overall/General manager 单位总经理
10. Others 其他

PROCEDURE 程序:
Skip to FD014. 跳至FD014.

FD013_w2_1 You told us that in the last IW you were 上一轮调查时 [ZIWTime] 您告诉我们您是 ______ [Preload information from the last IW 下载上一轮调查时的信息 (ZF18)], is the information correct? 是这样的吗?

1. Yes 是 → skip to FD013_w2_3 跳至FD013_w2_3
2. No 否

FD013_w2_2 What were your position in the last IW? 上一轮调查时 [ZIWTime] 您的职务是? ______

1. Clerk/worker 普通职工
2. Team Leader 组长（股长）
3. Section Chief 科长
4. Director of a division 处长
5. Director-General of a bureau and above 局长及以上
6. Village Leader 村干部
7. Township Leader 乡镇干部
8. Division manager 单位部门经理
9. Overall/General manager 单位总经理
10. Others 其他

FD013_w2_3 Are you still holding this position? 您现在还是这个职务吗?

1. Yes 是 → skip to FD014 branchpoint 跳至FD014 branchpoint
2. No 否

FD013_w2_4 What is your current position? 您目前的职务是什么?

1. Clerk/worker 普通职工
2. Team Leader 组长（股长）
3. Section Chief 科长
4. Director of a division 处长
5. Director-General of a bureau and above 局长及以上
6. Village Leader 村干部
7. Township Leader 乡镇干部
8. Division manager 单位部门经理
9. Overall/General manager 单位总经理
10. Others 其他

FD013_w2_5 When did you start doing your current work? 您是从什么时候开始做您现在这个工作的? ____ (**FD013_w2_5_1**) year 年 ____ (**FD013_w2_5_2**) month 月

FD014 BRANCHPOINT :

If old R is working for a different employer (FA006_w2_2a_3= 2),skip to FD014 如果老受访者为不同的雇主工作（FA006_w2_2a_3= 2），跳至FD014

If old R is working for the same employer but the information of the type of business is missing in the last IW (FA006_w2_2a_3= 1 & ZF19_1= .), skip to FD014_w2_2. 如果老受访者为同一个雇主工作但是单位的产业或行业类型缺失（FA006_w2_2a_3= 1 &ZF19_1= .），跳至FD014_w2_2

If old R is working for the same employer and the information of the type of business is not missing (FA006_w2_2a_3= 1 & ZF19≠= .), skip to FD014_w2_1. 如果老受访者为同一个雇主工作并且单位的产业或行业类型没有缺失（FA006_w2_2a_3= 1 & ZF19≠= .），跳至FD014_w2_1

New R please answer FD014. 新受访者，回答FD014

FD014 What is your current professional/technical level? 你目前的专业/技术职称是什么？

1. Technician 技术员
2. Primary level 初级职称
3. Intermediate level 中级职称
4. Advanced level 高级职称
5. No professional/technical level 无职称

F1：“专业/技术职称”指专业技术人员的专业技术与学识水平和工作能力的等级称号。

PROCEDURE 程序：

Skip to Procedure prior FD015. 跳至FD015前的程序控制.

FD014_w2_1 You told us in the last IW that your professional/technical level was 上一轮调查时 [ZIWTime] 您告诉我们你的专业/技术职称是 ______[preload the information of professional/technical level from the last IW 下载上一轮调查时的专业/技术职称信息 (ZF19)], is the information correct? 是这样的吗？

1. Yes 是 → skip to FD014_w2_3 跳至FD014_w2_3
2. No 否

FD014_w2_2 What was your professional/technical level in the last IW? 上一轮调查时 [ZIWTime] 您的专业/技术职称是？

1. Technician 技术员
2. Primary level 初级职称
3. Intermediate level 中级职称
4. Advanced level 高级职称
5. No professional/technical level 无职称

F1：“专业/技术职称”指专业技术人员的专业技术与学识水平和工作能力的等级称号。

FD014_w2_3 Is it still your professional/technical level? 您现在还是这个专业/技术职称吗？

1. Yes, it is 是，还是 → Skip to Procedure prior FD015. 跳至FD015 前的程序控制.
2. No，it is not 否，不是了

FD014_w2_4 What is your current professional/technical level? 你目前的专业/技术职称是什么?

1. Technician 技术员
2. Primary level 初级职称
3. Intermediate level 中级职称
4. Advanced level 高级职称
5. No professional/technical level 无职称

F1:“专业/技术职称”指专业技术人员的专业技术与学识水平和工作能力的等级称号。

FD014_w2_5 When did you start having the current professional/technical level? 您是什么时候开始有现在的专业/技术职称的? ____ (**FD014_w2_5_1**) year 年 ____ (**FD014_w2_5_2**) month 月

PROCEDURE 程序:

For new R: Skip FD015-FD016 if answer to FD013 is 2-9. 新受访者:如果FD013选择2-9,跳过FD015和FD016。

For old R: Skip FD015-FD016 if old R's position in the last IW (ZF18) is 2-9 & FD013_w2_1$=1$ & FD013_w2_3$=1$ or if FD013_w2_2$=2-9$ or if FD013_w2_4$=2-9$. 老受访者:如果老受访者上一轮调查时的职务(ZF18)是 2-9 并且 FD013_w2_1$=1$ 且FD013_w2_3$=1$,或者如果FD013_w2_2$=2-9$,或者如果FD013_w2_4$=2-9$,跳过FD015-FD016。

FD015 Are you in a position to supervise others? 您是否管理别人?

1. Yes 是
2. No 否 → Skip to FD017. 跳至FD017

FD016 How many people are there under your supervision? 您管着多少人?

1. 1~5 people 1~5 人
2. 6~10 people 6~10 人
3. 11~15 people 11~15 人
4. 16~30 people 16~30 人
5. 31~99 people 31~99 人
6. More than 100 people 超过 100 人

[CAPI: For dispatched worker(FD001 $=2$), prompt for FD017-FD030 对派遣工人 (FD001$=2$), 对FD017-FD030 均提示:

The next few questions are about your dispatch work unit. 下面几个问题是关于您派遣单位的情况。]

PROCEDURE 程序:

For new R: Do not ask FD017 to FD019 if FD013= 3 – 5 (government officials). 新受访者：如果FD013= 3 – 5（政府官员），跳过FD017 , FD018 , FD019.

For old R: Do not ask FD017 to FD019 if old R's position in the last IW (ZF18) is 3-5 & FD013_w2_1= 1 & FD013_w2_3= 1 or if FD013_w2_2= 3 – 5 or if FD013_w2_4= 3 – 5. 老受访者：如果老受访者上一轮调查时的职务（ZF18）是 3-5 并且 FD013_w2_1= 1 且 FD013_w2_3= 1，或者如果 FD013_w2_2= 3 – 5，或者如果 FD013_w2_4= 3 – 5，跳过 FD017 , FD018 , FD019.

FD017 What is your employment type at your current workplace? 您目前的受聘类型是？

1. Regular worker 劳务派遣工
2. Contract worker 合同制员工
3. Casual/Part-time worker 日工/钟点工

FD018 Do you have a personnel file? 您有档案吗？

1. Yes 是
2. No 否 → Skip to FD020. 跳至FD020

FD019 Where is your personnel file kept? 您的档案存放在哪里？

1. With my current employer 当前雇主处
2. Other work unit but not current employer 其他工作单位 (非当前雇主处)
3. With the Job Service Center in this city 本县/市人才服务中心
4. HuKou place in other city 户口所在地
5. Other city 其他城市

FD020 Did you receive a labor contract (or employment contract) in written form from your current workplace(or labor dispatch company)? 您与当前单位/劳务派遣单位签了书面的劳动合同吗？

1. Yes 是
2. No 否 → Skip to FD024. 跳至FD024

FD021 What is the agreed period of employment (labor contract period)? 您的劳动合同的期限是多长？

1. Defined period 固定期限 ____0...100 (**FD021_1**) Years 年 __0...11 (**FD021_2**) Months 月

   [IWER: if do not remember months, please fill '0'. 访员注意：如果记不住多少个月，请填"0"。]
2. Not defined 无固定期限 → Skip to FD024. 跳至 FD024
3. Same as the term of the project 以完成一定工作任务为期限的劳动合同

FD022 Has the current employment contract ever been renewed? 现在的劳动合同是续签过的吗？

1. Yes 是

2. No 否 → Skip to FD024. 跳至FD024

FD023 How many times has the contract been renewed? 您的劳动合同是第几次续签? _____
1...50 times 次

FD024 How long do you expect to work at your current workplace? 您预期还会在现在的单位干多久?

1. Less than one year 不到 1 年
2. One to two years 1-2 年
3. Two to three years 2-3 年 → Skip FD025. 请跳过FD025
4. More than three years 超过 3 年 → Skip FD025. 请跳过FD025

FD025 Why do you expect so? 你为什么这么觉得呢?

1. Because the predefined contract period will expire 因为之前签的合同快到期了
2. Because typically the contract expires (although there' s no written contract) 因为之前约定的时间快到了 (尽管没有书面合同)
3. Because I was hired under the condition that I would resign upon the request of my employer 因为估计雇主会让我辞职
4. Because the current job/project will be completed 因为目前的工作/项目即将结束
5. Because the person I am substituting/replacing will return to work 因为我顶替的那个人要回来了
6. Because I can only work during certain seasons 因为我只能在某些时间段工作
7. Because I plan to find another job that better suits my job aptitude, abilities, and preferences 因为我想找一份更适合我的工作
8. Because I will reach retirement age as set by regulations/practice 因为我快到退休年龄了
9. Because of family care responsibilities, poor health, etc. 因为家庭或健康方面的原因
10. Other 其他原因

PROCEDURE 程序:
If R has not processed retirement (FB011= 2) ask: 如果被访者还没有办理退休手续, 问:

FD026 Is this work unit going to process retirement for you? 您目前的工作单位将给您办理退休手续吗?

1. Yes 是
2. No 否 → Skip to FD029. 跳至FD029

FD027 At what age will you process retirement? 您将在多少岁办理退休手续? __ 45...120 Years old 岁 → Skip to FD029. 跳至 FD029
[Soft Check for Reasonable Age: If (FD027< 50 & CV004== 2) or (FD027< 55 & CV004== 1) prompt for verification.] [Add another check to see if the reported age above is smaller than actual age. To be specific, FD027<(Interview Year – CV009_a)]

PROCEDURE 程序:
If R has processed retirement (FB011= 1 or ZF15= 1) ask: 如果被访者已经办理退休手续，问:

FD028 Did you process retirement through this work unit? 您是在这个单位办的退休手续吗？
1. Yes 是
2. No 否

FD029 Except for national/public holidays, how many days of paid vacation do you have this year at your current workplace? 除国家法定节假日以外，今年您可以享受几天的带薪休假？___ 0...366 Days 天
[IWER: Mark 0 if there is no paid vacation. 访员注意：如果没有带薪休假，请标明“0”。]
[Soft Check for reasonable range: If FD029> 30?, prompt for verification]

FD030 How many days of work did you miss at this current job in the past year due to health problems? 在过去的一年中，您由于健康原因，在这个单位请假多少天？___ 0...366 Days 天
[IWER: Mark 0 if you didn't miss work. 访员注意：如果您没有不在职的情况，请标明“0”]

PROCEDURE 程序:
If FD030= 0, skip FD031. 程序：如果FD030= 0，跳过FD031。

FD031 In these days, how many did not deduct wage or bonus? 在这些天里，有多少天是不扣工资或奖金的？___ 0...366 Days 天
[Soft Check for reasonable range: If FD031>FD030?, prompt for verification]

FE QUESTIONS ABOUT LABOR SUPPLY 劳动力供给

[CAPI: For dispatched worker(FD001= 2), prompt for FE001-FE003 对派遣工人 (FD001= 2), 对FE001-FE003 均提示] The next few questions about labor supply are about the situation of your work place, not dispatch work unit. 下面几个关于劳动力供给的问题是指您工作单位的情况，而不是派遣单位。

FE001 Counting paid vacations and sick leave not deducting wage as work, how many months did you work in the past year? 过去一年中，您工作了几个月（带工资的假期和不扣工资的病假算作工作时间）？__ 0...12 Months 月

FE002 How many days a week did you work on average in the past year? 过去一年中，您一般每周工作几天？__0...7 Days 天

FE003 How many hours did you work per day on average in the past year, excluding meal breaks but including any paid or unpaid overtime? 过去一年中，您一般每天工作多少个小时？工作时间不包括午休时间，但包括加班时间（不管是否有报酬）__0...24 Hours 小时
[Soft Check: Verify if number of hours per day is unreasonable, e.g., FE003 >16]

FF QUESTIONS ABOUT WAGES 工资问题

[CAPI: For dispatched worker(FD001= 2), prompt for FF001-FG014 对派遣工人 (FD001= 2), 对 FF001-FG014 均提示] The following questions about salary and benefits refer to what you receive from the dispatch company. 下面几个问题是关于您从派遣单位获得工资和福利的情况。

FF001 How is your wage paid mainly? Is it regularly paid, contract-based, performance-based, or other? If it is regularly paid, please tell me how often you receive your wages. Do you have a yearly contract, monthly, weekly, daily, or hourly? Please select one. 您的工资主要是怎么支付的？是定期支付、按项目支付、按绩效支付还是其他？如果是定期支付，请回答您是多久领一次工资？您是否按年、月、周、日还是小时领取工资？请选择一个

1. Yearly salary 年薪
2. Monthly salary 月薪 → Skip to FF004. 跳至FF004
3. Weekly salary 周薪 → Skip to FF006. 跳至FF006
4. Daily salary 日薪 → Skip to FF008. 跳至FF008
5. Hourly salary 小时工资 → Skip to FF010. 跳至FF010
6. Contract-based 按项目 → Skip to FF012. 跳至FF012
7. Performance-based 按绩效 → Skip to FF012. 跳至FF012
8. Other 其他 → Skip to FF012. 跳至FF012

FF002 What is the after-tax salary including bonus in the last year? 把奖金等各种收入都算在内，您过去一年从单位拿到多少钱（税后）？ ____(**FF002_1**) Yuan 元其中，奖金有多少？ ____(**FF002_2**) Yuan 元或 __(**FF002_3**)%

[Soft Check: Prompt for clarification if under a low threshold, e.g. under 1200 RMB annual. Specifically, prompt to clarify if FF002< 1200 RMB.]

FF003 [IWER: If R is unwilling to answer or does not remember, ask unfolding bracket questions here. 访员注意：如果受访者不愿回答或者忘记了，在此处分级展开提问] 把奖金等各种收入都算在内，您过去一年从单位拿到多少钱（税后）？ 10,000 /30,000 /50,000 /100,000 /200,000 yuan 元

FF003_w2 [IWER: If R is unwilling to answer or does not remember, ask unfolding bracket questions here. 访员注意：如果受访者不愿回答或者忘记了，在此处分级展开提问] 其中，奖金有多少？ 10,000 /30,000 /50,000 /100,000 /200,000 yuan 元

PROCEDURE 程序:
Skip to FF014. 跳至 FF014

FF004 What is the after-tax salary including bonus in the last month? 把奖金等各种收入都算在内，您上个月从单位拿到多少钱（税后）？ ____(**FF004_1**) Yuan 元其中，奖金有多少？ ____(**FF004_2**) Yuan 元或 __(**FF004_3**)%

[Soft Check: Prompt for clarification if under a low threshold, e.g. under 100 RMB per month. Specifically, prompt to clarify if FF004 < 100 RMB.]

FF005 [IWER: If R is unwilling to answer or does not remember, ask unfolding bracket questions here. 访员注意：如果受访者不愿回答或者忘记了，在此处分级展开提问] 把奖金等各种收入都算在内，您上个月从单位拿到多少钱（税后）？500 /1,000 /2,500 /5,000 /10,000 yuan 元

FF005_w2 [IWER: If R is unwilling to answer or does not remember, ask unfolding bracket questions here. 访员注意：如果受访者不愿回答或者忘记了，在此处分级展开提问] 其中，奖金有多少？500 /1,000 /2,500 /5,000 /10,000 yuan 元

**PROCEDURE 程序:**
Skip to FF014. 跳至 FF014

FF006 What is the wage including bonus last week? 把奖金等各种收入都算在内，您上周从单位拿到多少钱（税后）？ ____Yuan 元

[Soft Check: Prompt for clarification if under a low threshold, e.g. under 25 RMB per week. Specifically, prompt to clarify if FF006 $<$ 25 RMB.]

**PROCEDURE 程序:**
Skip to FF012. 跳至 FF012

FF008 What is the usual daily wage? 您一般每天挣多少钱？ ____Yuan 元

[Soft Check: Prompt for clarification if under a low threshold, e.g. under 5 RMB per day. Specifically, prompt to clarify if FF008 $<$ 5 RMB.]

FF009 [IWER: If R is unwilling to answer or does not remember, ask unfolding bracket questions here. 访员注意：如果受访者不愿回答或者忘记了，在此处分级展开提问] 20 /50 /100 /200 /500 yuan 元

**PROCEDURE 程序:**
Skip to FF012. 跳至 FF012

FF010 What is your hourly wage? 您每小时的工资是多少？ ____Yuan 元

[Soft Check: Prompt for clarification if under a low threshold, e.g. under 1 RMB per hour. Specifically, prompt to clarify if FF010 $<$ 1 RMB.]

**PROCEDURE 程序:**
Skip to FF012. 跳至 FF012

FF012 How much on average do you receive last month after taxes (including bonus)? 把奖金等各种收入都算在内，您上个月税后拿到多少钱？ ____(**FF012_1**) Yuan 元其中，奖金有多少？ ____(**FF012_2**) Yuan 元或 __(**FF012_3**)%

[Soft Check: Prompt for clarification if under a low threshold, e.g. under 100 RMB per month. Specifically, prompt to clarify if FF012 $<$ 100 RMB.]

FF013 [IWER: If R is unwilling to answer or does not remember, ask unfolding bracket questions here. 访员注意：如果受访者不愿回答或者忘记了，在此处分级展开提问] 把奖金等各种收入都算在内，您上个月税后拿到多少钱？1,000 /3,000 /5,000 /10,000 /20,000 yuan 元

FF013_w2 [IWER: If R is unwilling to answer or does not remember, ask unfolding bracket questions here. 访员注意：如果受访者不愿回答或者忘记了，在此处分级展开提问] 其中，奖金有多少？1,000 /3,000 /5,000 /10,000 /20,000 yuan 元

FF014 What is the value of all other bonuses (not paid at same time as regular wage) received in the past year? 过去一年中，您从单位拿到的其他所有奖金（不和薪酬一起按期支付，比如年终奖等）一共有多少钱（税后）？____Yuan 元

[Soft Check: Prompt on bonus and monthly earnings if bonus is more than five times monthly net income]

FF015 [IWER: If R is unwilling to answer or does not remember, ask unfolding bracket questions here. 访员注意：如果受访者不愿回答或者忘记了，在此处分级展开提问] 1,000 /3,000 /5,000 /10,000 /20,000 yuan 元

FG Fringe Benefits 单位福利

[Show Card 出示卡片 21]

FG001 The following are fringe benefits which may be provided by a company. Please answer if the following are provided by your current workplace and whether you benefit from the following. (Check all that apply) 下面我们将列举一些福利。请回答您目前公司的福利情况以及您是否享受以下福利？(可多选)

1. Free lunch 免费午餐
2. Free breakfast 免费早餐
3. Free dinner 免费晚餐
4. Meal cash subsidy 餐费补贴
5. Transportation cash subsidizations 交通费补贴
6. Free housing 免费住宿
7. Subsidization of housing 住房补贴
8. Company car 单位配车
9. Company bus 单位班车
10. Other subsidies 其他补贴（包括实物折合）
11. None 没有 → Skip to FG003 请跳至 FG003

PROCEDURE 程序:
For each choice of FG001, ask FG002. 对上题的每一个答案，都分别提问 FG002

FG002 How much is the value of the subsidy per month? 每个月这种补助有多少（值多少钱）？____Yuan 元

PROCEDURE 程序:
If FD006 = 1 or FD007 = 1 or FD009 = 1 , then skip FD003-FD014 如果是公务员、政府机构的其他正式编制的员工、或事业单位的正式编制的员工FD006 = 1 或者FD007 = 1 或者FD009 = 1 , 跳过 FD003-FD014

PROCEDURE 程序: If R has not processed retirement(FB011= 2), ask FD003-FD014 程序: 如果受访者没有退休（FB011= 2), 问: FD003-FD014

[Show Card 出示卡片 22]

FG003 Does your employer provide pension insurance, health insurance, unemployment insurance, worker' s injury insurance and maternity insurance ? (Choose all that apply) 您的雇主是否提供养老保险、医疗保险、失业保险、工伤保险和生育保险？(可多选)

1. Pension 养老保险 → Skip to FG009 请跳至 FG009
2. Health insurance 医疗保险 → Skip to FG009 请跳至 FG009
3. Unemployment insurance 失业保险 → Skip to FG009 请跳至 FG009
4. Worker' s injury insurance 工伤保险 → Skip to FG009 请跳至 FG009
5. Maternity insurance 生育保险 → Skip to FG009 请跳至 FG009
6. None 没有

FG004 Why are you not covered by the above mentioned social insurance through your employer?（Choose all that apply）? 单位为什么没有给你上社会保险（包括养老保险、医疗保险、失业保险、工伤保险、生育保险）？(可多选)

1. There is no social insurance through employment in my local area 本地没有职工社会保险 → Skip to FG014 请跳至 FG014
2. Social insurance through work is available in my local area but my employer does not provide it to me 本地有这些项目但是我的单位不给我上保险 → Skip to FG014 请跳至 FG014
3. My employer offers it but I am unwilling to join 单位虽然提供但是我不愿意参加 → Skip to FG014 请跳至 FG014
4. I am in the trial period 我在试用期中间 → Skip to FG014 请跳至 FG014
5. I have social insurance from elsewhere 我在其他地方上了保险 → Skip to FG005 请跳至 FG005
6. I have passed retirement age 我已经过了退休年龄 → Skip to FG014 请跳至 FG014

FG005 From where do you have/receive the above mentioned social insurance? 您在哪儿上的社会保险（包括养老保险、医疗保险、失业保险、工伤保险、生育保险)?

1. I am covered through another work unit 通过另一个工作单位 → Skip to FG006 请跳至 FG006
2. I contribute through the job service center 通过人才交流中心 → Skip to FG008 请跳至 FG008

FG006 Why do you contribute the above mentioned social insurance fees through another work unit? 您为什么要通过另一个工作单位缴纳社会保险（包括养老保险、医疗保险、失业保险、工伤保险、生育保险）费?

1. I was laid off by that work unit but my employment contract has not terminated 我在那家工作单位下岗，但劳动关系没有终止。
2. I am on leave from that employer 我从这那家单位请假。
3. I keep my position but do not receive pay from that work unit 我在那家工作单位办了停薪留职。
4. A friend in that work unit is helping me this way 我在那家单位有个朋友帮我。
5. Other 其他

FG007 Where is this work unit located? 这个单位在哪?

1. Same city 就在这个城市
2. Different city, same province 省内的其他城市
3. Different province 其他省

**PROCEDURE 程序**:
Skip to FF009. 跳至 FF009

FG008 Who pays for your contribution through the job service center? 您通过人才交流中心上社会保险（包括养老保险、医疗保险、失业保险、工伤保险、生育保险），请问由谁出钱?

1. My employer 雇主
2. Myself 我自己
3. My employer and myself 雇主和我各负担一部分

**PROCEDURE 程序**:
All those paying social insurance through whatever means, ask(FG003 = 1 − 4 or FG004 = 5). 只要上了社会保险的人，不管通过什么渠道上的，都请回答：FG009 – FG013

FG009 Is the salary you told me about earlier the net amount after paying for the above benefits? 您刚才告诉我的您的收入，扣除了这些上交的保险金额吗?

1. Yes 是
2. No 否

FG010 On what income base is the contribution to the above mentioned social insurance determined? 请问您缴纳社会保险（包括养老保险、医疗保险、失业保险、工伤保险、生育保险）费时是根据多少工资计算的? ____ Yuan/month 元/月

FG011 Do you know how much you or your employer (another work unit) contributes to the above benefits? 您知道您或者您的雇主（另一个工作单位）给您缴纳了多少社会保险（包括养老保险、医疗保险、失业保险、工伤保险、生育保险）吗?

1. I know the amount by myself and the employer 我知道我和雇主各缴了多少
2. I know the amount by myself but not the employer 我只知道我自己缴纳了多少，但不知道雇主缴了多少。→ Skip to FG013 请跳至 FG013

3. I know neither my contribution nor my employer's contribution. 我既不知道我缴了多少，也不知道雇主缴了多少。→ Skip to FG014 请跳至 FG014

FG012 How much is your employer's contribution in total? 您的雇主每月给您缴了多少？____Yuan/month 元/月
Soft Check Income Report and FG012 if Contribution is Greater than Monthly Income

FG013 How much is your own contribution in total? 您个人每个月缴纳了多少社会保险（包括养老保险、医疗保险、失业保险、工伤保险、生育保险）？____Yuan/month 元/月 Soft Check Income Report and FG013 if Contribution is Greater than Monthly Income

FG014 Does your employer provide funding for public housing? 您的雇主是否提供住房公积金？

1. Yes 是
2. No 否

F1："住房公积金"指单位及其在职职工缴存的长期住房储金，是住房分配货币化、社会化和法制化的主要形式。Soft Check: Verify if FG014 > than some threshold.

FG015 At what age do you plan to stop working? Stopping work in this context shall refer to having stopped all income-related activities and unpaid family business and having no intention of engaging in anything more serious than small pastime work. 您计划在多大年龄时停止工作，即停止从事一切以挣钱为目的的活动以及不再为家庭经营活动帮工，将来也不打算从事比消遣性工作更劳累的工作?____0...120 Years old 岁
F1："消遣性工作"指不是以挣钱为目的的活动，以及不再为家庭经营活动帮工的工作。Soft Check: Prompt for correction if current age is greater than response, e.g. (Interview Year+ Interview month/12) – (CV009_a+CV009_b/12) > FG015

PROCEDURE 程序:
Skip to FJ001. 跳至 FJ001

FH NON-FARM SELF-EMPLOYED AND UNPAID FAMILY BUSINESS 非农自雇和为家庭经营活动帮工

FH001 How many months did you work in the past year? 过去一年中，您工作了几个月？__ 0...12 Months 月

FH002 How many days did you work per week on average in the past year? 过去一年中，您一般每周工作几天？__ 0...7 Days 天

FH003 How many hours did you work per day on average in the past year, excluding meal breaks but including any paid or unpaid overtime on a normal work month? 过去一年中，您一般每天工作多少个小时？不包括午餐和其他休息时间，但是包括加班时间，不管是否有报酬 __ 0...24 hours 小时
Soft Check: Verify if number of hours per day is unreasonable, e.g., FH003 >16

FH004 How many days of work did you miss in the past year due to health problems? 过去一年中，您由于健康原因，有多少天没有干活？____ 0...366 Days 天
[IWER: Mark 0 if you didn't miss any work days. 访员注意：如果没有，请注明“0”。]

[Next are some questions about the main self-employed work. 下面是关于您从事的主要个体或者私营经济活动的问题]

PROCEDURE 程序:
IF FC020= 2 or FC021= 2[Self-employed], ask 如果FC020= 2 或者 FC021= 2，受访者自雇，询问：
If old R is running the same company or workplace (FA006_w2_2b_3= 1), skipFH005 如果老受访者开同一家公司/做同一个生意，跳过FH005

FH005 What is the name of your company or workplace? 您的企业或者生意的名称是？(请标明详细的单位名称) ____________ (例 1：北京华信服装服饰有限责任公司；例 2：丽丽服装店)
[IWER: If there is more than one company, ask about the main one. Mark 0 if there is no name. 访员注意：如果在几个企业工作或者做了多项生意，问主要工作的企业或者主要从事的生意。如果没有名称，请标明“0”。]

FH006 Where is your company or workplace located? 您的公司/生意的地址是？

1. The same as permanent address 和目前常住地一样
2. Another village/neighborhood in permanent address's county/city/district 常住地所在县/市/区的其他村/社区 ____(**FH006_1**)village/neighborhood 村/社区
3. Other 其它：____(**FH006_2**) province_city_county/city/district 省 _ 市 _ 县/市/区，____(**FH006_3**) village/neighborhood 村/社区
4. Abroad 国外

[IWER: 访员注意：如果记不清详细的地址名称，请在相应输入框中填入“其它”。]

FH007 BRANCHPOINT]:
If old R is running the same company or workplace (FA006_w2_2b_3= 1) and the information of the type of business is not missing, skip to FH007_w2_1 如果老受访者开同一家公司/做同一个生意且生意类型没有缺失，跳至FH007_w2_1
If old R is running the same company or workplace (FA006_w2_2b_3= 1 & ZF9_1= .) and the information of the type of business is missing, skip to FH007_w2_2 . 如果老受访者开同一家公司/做同一个生意但是生意信息缺失，跳至 FH007_w2_2。
If old R is running the different company or workplace (FA006_w2_2b_3= 1 & ZF9_1≠= .), skip to FH007. 如果老受访者开不同的公司/做不同的生意，跳至 FH007。
New R please answerFH007. 新受访者，请回答FH007。

FH007 What kind of business or industry do you work in—that is, what does your company do or make? 您主要做什么生意，即生产什么产品或者从事什么经营活动？____________ (例 1：制造纱质布料；例 2：销售服装)
[IWER: Type of business 产业或行业类型。注意：回答中请不要用逗号。]

PROCEDURE 程序:
Skip to FH008. 跳至 FH008

FH007_w2_1 In the last IW, you told us that your company did or made 上一轮调查时 [ZIWTime]，您告诉我们您的公司/生意是做 ____________[preload information of type of business of company or business (ZF9_1) 下载上一轮调查时公司/生意的产业或行业类型信息], is the information correct? 是这样的吗？

1. Yes 是 → skip to FH007_w2_3 请跳至 FH007_w2_3
2. No 否

FH007_w2_2 What did your company do or make in the last IW? 上一轮调查时 [ZIWTime] 您的公司/生意是做什么？ _________.（例 1：制造纱质布料；例 2：销售服装）
[IWER: Type of business 产业或行业类型。注意：回答中请不要用逗号。]

FH007_w2_3 Is your company still doing or making it? 您的公司/生意现在还是做这个吗？

1. Yes 是 → skip to FH009 请跳至 FH009
2. No 否

FH007_w2_4 What is your company doing or making now? 您的公司/生意现在是做什么？ _________.（例 1：制造纱质布料；例 2：销售服装）
[IWER: Type of business 产业或行业类型。注意：回答中请不要用逗号。]

PROCEDURE 程序:
If old R is running the same company or business(FA006_w2_2b_3= 1), skipFH008 如果老受访者开同一家公司/做同一个生意，跳过FH008

FH008 When did you start working at the current company or workplace? 您什么时候开始经营这个企业/这项生意？ ____1900...2013 (**FH008_1**) Year 年 __0...12 (**FH008_2**) Month 月
[IWER: Mark the year using four digits. Take down the month as its actual number. For example, write January as “1” not “01”, December as “12”. If do not remember month, fill ‘0’. 访员注意：用 4 位数表示年，按照实际的月份填写月。例：1 月写作“1”，而不是“01”，12 月写作“12”。如果记不住月份，请填入’0’。]
[A check similar to the one for FD011. (FD011_1 + FD011_2 /12)- (CV009_a + CV009_b/12) < 16 or (FD011_1 + FD011_2 /12)- (CV009_a + CV009_b/12) < FB001_1 or FH008_1 < FB001_2]

FH009 Do any other household members work in the same self-employed activity? 您是否还有其他家人也从事同样的个体或者私营经济活动？

1. Yes 是 → skip to FI001 请跳至 FI001
2. No 否

[IWER: Income from self-employment if R is the single operator（FH009= 2）. If other family member involved, income is asked in household section. 访员注意：如果受访者是自己一个人开公司、做生意等，那么接下来问收入；如果还有其他家人一起做事，那么跳至FI001，在家庭问卷中问收入。]

FH010 Not including spending on fixed capital, what is your best estimate of net income earned from this activity in the last year? Remember to consider the following types of costs: energy, housing or equipment rental, raw materials, transportation, marketing, wages, taxes, and other fees. 不包括在固定资本上的投入，您能否估计一下过去一年您开这个公司/做这项生意挣了多少钱？我们指的是刨除能源、住房和设备租用费、原材料、交通费、营销、工资、税收和杂费之后的净收入。____ Yuan 元

Soft Check: Prompt for verification if net income is less than 1200 RMB / year. Or, prompt if FH010 <1200 RMB

FH011 [IWER: If R is unwilling to answer or does not remember, ask unfolding bracket questions here. 访员注意：如果受访者不愿回答或者忘记了，在此处分级展开提问] 5,000 /10,000 /50,000 /100,000 /200,000 yuan 元

**PROCEDURE 程序:**
Skip to FI001 请跳至 FI001

Next are some questions about your unpaid family business. 下面是关于您不拿工资为家庭经营活动帮工的问题

FH012 What is the name of company or workplace that you work in without wage? 您无偿帮忙的企业或者生意的名称是？_________ （例 1：北京华信服装服饰有限责任公司；例 2：丽丽服装店）

[IWER:Mark 0 if there is no name. 如果没有名称，请标明“0”.]

FH013 Where is this company or workplace loacted? 这个公司/生意的地址是？

1. The same as permanent address 和目前常住地一样
2. Another village/neighborhood in permanent address's county/city/district 常住地所在县/市/区的其他村/社区 ____(**FH013_1**)village/neighborhood 村/社区
3. Other 其它：____(**FH013_2**) province_city_county/city/district 省 _ 市 _ 县/市/区，____(**FH013_3**) village/neighborhood 村/社区
4. Abroad 国外

[IWER: 访员注意：如果记不清详细的地址名称，请在相应输入框中填入“其它”。]

FH014 BRANCHPOINT]:

If old R is working in a different unpaid family business(FA006_w2_2c_3= 2) skip to FH014 如果老受访者为不同的家庭经营活动无偿帮工，跳至FH014

If old R is working in a same unpaid family business but the information of the type of business is missing in the last IW(FA006_w2_2c_3= 1 & ZF10_1= .) skip to FH014_w2_2. 如果老受访者为同一个家庭经营活动无偿帮工，但是上一轮调查时家庭经营活动的产业或行业类型缺失，跳至FH014_w2_2。

If old R is working in a same unpaid family business and the information of the type of business is not missing in the last IW (FA006_w2_2c_3= 1 & ZF10_1/ = .), skip to FH014_w2_1. 如果老受访者开不同的公司/做不同的生意，跳至 FH014_w2_1。

New R please answerFH014. 新受访者，请回答FH014。

FH014 What kind of business or industry do you work in—that is, what does this company do or make? 他们主要做什么生意，即生产什么产品或者从事什么经营活动？ _________. （例 1：制造纱质布料；例 2：销售服装）

[IWER: Type of business 产业或行业类型。注意：回答中请不要用逗号。]

PROCEDURE 程序:

Skip to FH015 请跳至 FH015

FH014_w2_1 You told us in the last IW that the type of business of company or workplace you worked without wage did or made 上一轮调查时 [ZIWTime] 您告诉我们您无偿帮工的公司/生意是做 _________[preload information of the type of business from last IW (ZF10_1) 下载上一轮调查时不拿工资帮工的公司/生意的产业或行业类型]，is the information correct? 是这样的吗？

1. Yes, it is correct 是，信息正确 → skip to FH014_w2_3 请跳至 FH014_w2_3
2. No, it is not correct 否，信息不正确

FH014_w2_2 What did the company or workplace do or make in the last IW? 上一轮调查时 [ZIWTime] 这个公司/生意是做什么的？ _________. （例 1：制造纱质布料；例 2：销售服装）

[IWER: Type of business 产业或行业类型。注意：回答中请不要用逗号。]

FH014_w2_3 Is the company or workplace still doing or making it? 这个公司/生意现在还是做这个吗？

1. Yes 是 → skip to FH015 Branchpoint 请跳至 FH015 Branchpoint
2. No 否

FH014_w2_4 What is the company or workplace doing or making now? 这个公司/生意现在做什么？ _________. （例 1：制造纱质布料；例 2：销售服装）

[IWER: Type of business 产业或行业类型。注意：回答中请不要用逗号。]

FH014_w2_5 When did the company or workplace start doing this? 这个公司/生意是从什么时候开始做这个的？ ____ (**FB014_w2_5_1**) Years 年 _____(**FB014_w2_5_2**) Months 月

FH015 BRANCHPOINT :

If old R is working in a different unpaid family business FA006_w2_2c_3= 2) skip to FH015 如果老受访者为不同的家庭经营活动无偿帮工，跳至FH015

If old R is working in a same unpaid family business but the information of the work is missing in the last IW (FA006_w2_2c_3= 1 & ZF22_1= .) skip to FH015_w2_2. 如果老受访者为同一个家庭经营活动无偿帮工，但是上一轮调查时的工作信息缺失，跳至FH015_w2_2。

If old R is working in a same unpaid family business and the information of the work is not missing in the last IW (FA006_w2_2c_3= 1 & ZF22_1≠= .), skip to FH015_w2_1. 如果老受访者为同一个家庭经营活动无偿帮工，且上一轮调查时的工作信息没有缺失，跳至FH015_w2_1。

New R please answerFH015. 新受访者，请回答FH015。

FH015 What sort of work did you do? 您具体从事什么工作？ ________. （例 1：会计；例 2：帮助店主销售服装）

[IWER: Ask the specific work. 访员注意：请详细填写具体工作内容。注意：回答中请不要用逗号。]

PROCEDURE 程序:

Skip to FH016 请跳至 FH016

FH015_w2_1 You told us in the last IW that you were doing 上一轮调查时 [ZIWTime] 您告诉我们您是做 ________[preload information of the work from last IW (ZF22) 下载上一轮调查时的工作信息]，is it correct? 是这样的吗？

1. Yes 是 → skip to FH015_w2_3 请跳至 FH015_w2_3
2. No 否

FH015_w2_2 What were you doing in the last IW? 上一轮调查时 [ZIWTime] 您做什么工作？ ________. （例 1：会计；例 2：帮助店主销售服装）

[IWER: Ask the specific work. 访员注意：请详细填写具体工作内容。注意：回答中请不要用逗号。]

FH015_w2_3 Are you still doing this work? 您还在做这项工作吗？

1. Yes 是 → skip to FH016 请跳至 FH016
2. No 否

FH015_w2_4 What sort of work are you doing now? 您现在具体从事什么工作？ ________. （例 1：会计；例 2：帮助店主销售服装）

[IWER: Ask about the specific work that R does. 访员注意：请详细填写具体工作内容。注意：回答中请不要用逗号。]

FH015_w2_5 When did you start doing your current work? 您是从什么时候开始做您现在这个工作的？ ____ (**FB015_w2_5_1**) Years 年 ____(**FB015_w2_5_2**) Months 月

FH016 How many family members, relatives or friends who work without payment are there including yourself? 您有多少家人、亲戚或朋友在这个公司/生意不拿工资做事的？(包括您本人）__ People 人

FH017 When did this company or workplace start operation? 这个企业/生意是什么时候开办/经营的？____1900...2013 (**FH017_1**) Year 年 __0...12 (**FH017_2**) Month 月

FH018 When did you start working at the current company or workplace? 您什么时候开始在这个企业/生意帮忙的？____1900...2013 (**FH018_1**) Year 年 __0...12 (**FH018_2**) Month 月

FH019 Do any other household members work in the same company or workplace? 您是否还有其他家人也在这个企业/生意工作？

1. Yes 是 → skip to FI001 请跳至 FI001
2. No 否

[IWER: Income from self-employment if no other household members work in（FH019= 2）. If other family member involved, income is asked in household section. 访员注意：如果没有其他家人在企业/生意工作，那么接下来问收入；如果还有其他家人一起做事，那么跳至FI001，在家庭问卷中问收入。]

FH020 Not including spending on fixed capital, what is your best estimate of net income earned from this activity in the last year? Remember to consider the following types of costs: energy, housing or equipment rental, raw materials, transportation, marketing, wages, taxes, and other fees. 不包括在固定资本上的投入，您能否估计一下过去一年这个公司/做这项生意挣了多少钱？我们指的是刨除能源、住房和设备租用费、原材料、交通费、营销、工资、税收和杂费之后的净收入。____ Yuan 元

Soft Check: Prompt for verification if net income is less than 1200 RMB / year. Or, prompt if FH020 $<$ 1200 RMB

FH020_bracket [IWER: If R is unwilling to answer or does not remember, ask unfolding bracket questions here. 访员注意：如果受访者不愿回答或者忘记了，在此处分级展开提问]
5,000 /10,000 /50,000 /100,000 /200,000 yuan 元

FI Social insurance questions for the self-employed. 自雇部分中的社会保险问题

**PROCEDURE 程序**: If R has not processed retirement(FB011 = 2), ask: FI001-FI011 . 如果受访者没有退休（FB011 = 2），问：FI001-FI011。

[Show Card 出示卡片 22]

FI001 Next are questions about your social insurance. Are you covered by pension insurance, health insurance, unemployment insurance, worker’s injury insurance or maternity insurance through your own self-employment? (Choose all that apply) 下面是一些关于社会保险的问题。您是否以个体或者私营业主的身份缴纳了养老保险、医疗保险、失业保险、工伤保险或生育保险？(可多选)

1. Pension 养老保险 → skip to FI005 请跳至 FI005
2. Health insurance 医疗保险 → skip to FI005 请跳至 FI005
3. Unemployment insurance 失业保险 → skip to FI005 请跳至 FI005
4. Worker’s injury insurance 工伤保险 → skip to FI005 请跳至 FI005
5. Maternity Insurance 生育保险 → skip to FI005 请跳至 FI005
6. None 没有

FI002 Why are you not covered by the above mentioned social insurance through self-employment? 您为什么没有给自己上社会保险（包括养老保险、医疗保险、失业保险、工伤保险、生育保险）？

1. It is not possible for the self-employed to participate in social insurance in my local area 在本地自雇经营的人没有办法上社会保险 → skip to FI012 请跳至 FI012
2. Social insurance is available to the self-employed but I do not participate 我可以上保险但是我不愿意参加 → skip to FI012 请跳至 FI012
3. I have social insurance from elsewhere 我在其他地方上了保险
4. I have passed retirement age 我已经过了退休年龄 → skip to FI012 请跳至 FI012
5. I don’t know how to participate in social insurance 不知道怎么上社会保险 → skip to FI012 请跳至 FI012
6. Other 其他 → Skip to FI012 → skip to FI012 请跳至 FI012

FI003 From where do you have the above mentioned social insurance? 您在哪上的社会保险(包括养老保险、医疗保险、失业保险、工伤保险、生育保险)?

1. I am covered through another work unit 我通过另一个工作单位上的
2. I contribute through job service center 我通过人才交流中心上的 → skip to FI005 请跳至 FI005

FI004 You contribute through another work unit, why? 请问您为什么通过另一个工作单位缴纳社会保险（包括养老保险、医疗保险、失业保险、工伤保险、生育保险）费?

1. I was laid-off by that work unit but employment contract is not terminated 我在那家工作单位下岗，但工作关系没有中止
2. I am on leave from that employer 我从那家单位请假，工作关系没有变动
3. I keep my position but do not receive pay from that work unit 我在那家工作单位办了停薪留职，没有转工作关系
4. A friend in that work unit is helping me this way 我在那家单位有个朋友帮我
5. Other 其他

**PROCEDURE 程序**: If R has social insurance, either from self-employment or by job service center, ask: FI005–FI011 . 如果受访者上了社会保险，无论是通过自雇还是人才交流中心，请提问: FI005–FI011

FI005 How much is your own contribution to the above mentioned social insurance last year? 您过去一年上社会保险（包括养老保险、医疗保险、失业保险、工伤保险、生育保险）个人缴纳多少钱？____ Yuan 元

Soft Check: Prompt if FI005 = 0 or FI005>FI010/2.

**PROCEDURE 程序**:
If FI009 = 2，skip FI006．如果FI009= 2，跳过FI006

FI006 Is the income you told us about earlier net after paying for the above benefits? 您刚才告诉我们的净收入，扣除了这些上交的保险金额吗？

1. Yes 是
2. No 否

FI007 On what income base is the contribution to the above mentioned social insurance determined? 请问您缴纳社会保险（包括养老保险、医疗保险、失业保险、工伤保险、生育保险）费时是根据多少收入计算的？____ Yuan/month 元/月

**PROCEDURE 程序**: If R participates in social insurance through another work unit (FI003 = 1), ask FI008–FI010．如果被访者通过另一个单位缴纳社会保险（FI003 = 1），提问：FI008–FI010

FI008 Do you know how much you or that work unit contributes to the above benefits? 您知道您或者那个工作单位给您缴纳了多少社会保险（包括养老保险、医疗保险、失业保险、工伤保险、生育保险）吗？

1. I know the amount by myself and the work unit 我知道我和单位各缴了多少 → skip to FI009 请跳至 FI009
2. I know the amount by myself but not the work unit 我只知道我自己缴纳了多少，但不知道单位缴了多少。→ skip to FI010 请跳至 FI010
3. I do not know either my contribution or the work unit’s contribution at all. 我既不知道我缴了多少，也不知道单位缴了多少。→ skip to FI011 请跳至 FI011

FI009 How much is work unit’s contribution in total? 单位每月给您缴了多少？__ Yuan/month 元/月

Soft Check: Prompt for Verification if Contribution is Greater than Monthly Earnings, e.g., FI009 >FH010

FI010 How much is your own contribution to pension in total? 您个人每月缴多少社会保险(包括养老保险、医疗保险、失业保险、工伤保险、生育保险)？____ Yuan/month 元/月

Soft Check: Prompt for Verification if Contribution is Greater than Monthly Earnings, e.g., FI010 > FH010

FI011 Do you expect to process retirement at this business/organization? 您预期将来在这个单位办理退休手续吗？

1. Yes 是
2. No 否

FI012 At what age do you plan to stop working? Stopping work in this context shall refer to having stopped all income-related activities and unpaid family business and having no intention of engaging in anything more serious than small pastime work. 您计划在多大年龄停止工作？即停止从事一切以挣钱为目的的活动以及不再为家庭经营活动帮工，将来也不打算从事比消遣性工作更劳累的工作?____ 0...120 Years old 岁
F1："消遣性工作"指不是以挣钱为目的的活动，以及不再为家庭经营活动帮工的工作。

[IWER: Please tell me the approximate age. Mark 0 if you plan to keep working until you are physically able. 访员注意：请告知大概年龄。如果您计划只要健康允许，就一直工作，请标明"0"。] Soft Check: Prompt for correction if current age is greater than response, e.g. (Interview Year+ Interview month/12) – (CV009_a+CV009_b/12) > FI012

FJ SIDE JOB (EMPLOYED OR SELF-EMPLOYED) 非主要职业(受雇或自雇)

If FC019 = 1(more than one job), proceed with the following section. 如果 FC019 = 1(工作超过一份）问这部分

FJ001 How many jobs do you currently hold, excluding your main job? 除了主要工作，您目前还从事其他几份工作？__ 1...20 份

FJ002 How many hours a week do you work on average at your side job(s), not considering your main job? 除了主要工作，您每周在其他工作上平均工作多少个小时？____ 0...168 Hours per week 小时/周

FJ003 What is the average monthly income or wage that you get from side job(s) other than your main job? 除了主要工作的收入外，您从事其他工作每月的平均收入是？____ Yuan per month 元/月

FJ004 [IWER: If R is unwilling to answer or does not remember, ask unfolding bracket questions here. 访员注意：如果受访者不愿回答或者忘记了，在此处分级展开提问:] 500 /1,000 /2,500 /5,000 /10,000 yuan 元

FK UNEMPLOYMENT AND JOB SEARCH ACTIVITIES 失业，求职经历

PROCEDURE 程序:

- If new R is not working but has worked before (FA007= 1 or FA008= 2), answer FK. 如果新受访者现在没有工作但是曾经工作过，询问 FK 部分。
- If old R is not working (XF1= 2) but worked before (FA006_w2_5> 0 or ZF13= 1 or ZF14= 2), answer FK 如果老受访者现在没有工作但是曾经工作过，询问 FK 部分。

FK001 Next are some questions about circumstances about your non-employment and job search activities. In what month and year did you last work? 下面是一些关于您停止工作前后的一些情况和找工作的问题。您什么时候开始不工作的？ ____ 1900...2013 (**FK001_1**) Year 年 __0...12 (**FK002_2**) Month 月

[IWER: Mark the year using four digits. Take down the month as its actual number. For example, write January as "1" not "01", December as "12". If do not remember month, fill '0'. 访员注意：用 4 位数表示年，按照实际的月份填写月。例：1 月写作"1"，而不是"01"，12 月写作"12"。如果记不住月份，请填入' 0'。]

FK002 Did you search for a new job during the last month? 过去一个月您是否找过工作？

1. Yes 是
2. No 否 → Skip to FL001 请跳至 FL001

FK003 At what age do you plan to stop working? Stopping work in this context shall refer to having stopped all income-related activities and unpaid family business and having no intention of engaging in anything more serious than small pastime work. 您计划在多大年龄停止工作？即停止从事一切以挣钱为目的的活动以及不再为家庭经营活动帮工，将来也不打算从事比消遣性工作更劳累的工作? ___0...120 Years old 岁

[IWER: Please tell me the approximate age. Mark 0 if you plan on working until you are physically capable. 访员注意：请告知退休的大约年纪。如果您计划只要身体健康就一直工作，请注明"0"。]

FL LAST JOB 最近一份工作

FL001 BRANCHPOINT :

For Old R: 老受访者：If old R' s last job is not missing (ZF22_1 = 1), skip FL. 如果老受访者最近一份工作没有缺失，跳过 FL 部分。If old R' s last job is missing (ZF22_1 = 2), please skip to procedure before FL001. 如果老受访者最近一份工作缺失，请跳至FL001前的程序控制。

For new R: 新受访者：Please skip to procedure before FL001. 新受访者，请跳至FL001前的程序控制。

PROCEDURE 程序:

RESPONDENT NOT CURRENTLY WORKING 目前没有工作的人，但曾经工作过的人回答

Ask if FA007 = 1 or FA008 = 2

[IWER: The next questions are about the last main job you had, which could be farming, earning a wage, running your own business or working for unpaid family business. It does not include doing your own housework or doing activities without pay, such as voluntary work. If you have more than one job, we are interested in the job at which you worked the longest hours. We' re interested in your situation near the termination of this job. 下面的问题是关于您最近一份工作的情况，可以是务农、挣工资工作、从事个体、私营经济活动、或者不拿工

资为家庭经营活动帮工，但不包括家务劳动或者志愿者劳动等没有报酬的工作。如果您之前同时从事不止一份工作，我们想了解您的主要工作，即占用您时间最多的那份工作。]

FL001 Did you work for someone else(including work for unpaid family business), were you self-employed, did you farm, or were you otherwise employed? 您最近一份工作是为别人工作（包括不拿工资为家庭经营活动帮工）还是自己从事个体或者私营经济活动，或者务农？

1. Employed 受雇（包括非农打工和在外地的农业打工等）
2. Self-employed 自雇（从事个体或者私营经济活动）
3. Unpaid family business 不拿工资为家庭经营活动帮工
4. Farming 农业生产活动（农业生产经营活动，包括为自家或其他农户种地、管理果树、采集农林产品、养鱼、打鱼、养牲畜以及去市场销售自家生产的农产品等；但不包括在外地的农业打工）

FL002 In which year and month did you start working at that job? 您从哪年哪月开始做这份工作的？ ____ 1900...2013 (**FL002_1**) Year 年 __0...12 (**FL002_2**) Month 月

[IWER: Mark the year using four digits. Take down the month as its actual number. For example, write January as “1” not “01”, December as “12”. If do not remember month, fill ‘0’. 访员注意：用 4 位数表示年，按照实际的月份填写月。例：1 月写作“1”，而不是“01”,12 月写作“12”。如果记不住月份，请填入’0’。] [Soft Check: Prompt for Verification/Clarification if the Respondent was Less than 16 at time of starting this job, e.g. Prompt if (FL002_1+FL002_2 /12)-(CV009_a+CV009_b/12)<16] [Replace 16 in the above check with FB001 . FL002_1 < FB001_2 or FL002_1 < (CV009_a + FB001_1)]

FL003 In which year and month did you stop working at that job? 您什么时候停止做这份工作的？ ____ 1900...2013 (**FL003_1**) Year 年 __0...12 (**FL003_2**) Month 月

[IWER: Mark the year using four digits. Take down the month as its actual number. For example, write January as “1” not “01”, December as “12”. If do not remember month, fill ‘0’. 访员注意：用 4 位数表示年，按照实际的月份填写月。例：1 月写作“1”，而不是“01”,12 月写作“12”。如果记不住月份，请填入’0’。] [Soft Check: Prompt for Verification/clarification if End Date is Before the Start Date, e.g. FL003_1 < FL002_1]

FL004 BRANCHPOINT：

For old R, if FL003_1<Last IW Year（ZIWYear）or FL003_1=Last IW Year（ZIWYear）and FL003_2<Last IW Month（ZIWMonth），then skip to Section FM. 对于老受访者，如果FL003_1< 上一轮调查年（ZIWYear）或者 FL003_1= 上一轮调查年（ZIWYear）并且 FL003_2< 上一轮调查月（ZIWMonth），请跳至 FM 部分。

FL004 Where was the job located? 工作地点在哪里？

1. The same as permanent address 和目前常住地一样
2. Another village/neighborhood in permanent address’s county/city/district 常住地所在县/市/区的其他村/社区 ____(**FL004_1**) village/neighborhood 村/社区

3. Other 其它：____(**FL004_2**) province_city_county/city/district 省 _ 市 _ 县/市/区，____(**FL004_3**) village/neighborhood 村/社区
4. Abroad 国外

[IWER: 访员注意：如果记不清详细的地址名称，请在相应输入框中填入“其它”。]

PROCEDURE 程序:
If FL001 = 4 Skip to FL020 如果FL001 = 4，跳至FL020
If non-farmer (FL001= 1/2/3), ask FL005 to FL008 . 如果不是农民（FL001= 1/2/3），问FL005 to FL008。

FL005 What was the name of your workplace/employer? Please state specifically the name of your company or business. 您的工作单位或者雇主是什么名称？（请详细标明名称）工作单位名称：________________ (**FL005_1**)（例 1：北京大学餐饮服务中心；例 2：北京华信服装服饰有限责任公司），所在部门名称：____________ (**FL005_2**)（例 1：农园餐厅；例 2：纺纱生产车间）

FL006 What kind of business or industry was it—that is, what did they make or do at the place where you worked? 该单位主要是做什么的，也就是，他们制造什么产品或者从事什么活动？__________（例 1：为校内提供餐饮服务；例 2：制造纱质布料）
[IWER: Type of business 访员注意：产业或行业类型。注意：回答中请不要用逗号。]

FL007 Is this employer still in existence? 这个单位还存在吗？
1. Yes 是
2. No 否

FL008 How many hours a week did you usually work [for this employer/in this business]? 您当时一般每周工作几个小时？[做这份工作 ___0...168 Hours per week 小时/周
[Soft Check: Prompt for Verification if FL008 > 80]

PROCEDURE 程序:
Ask only if employed. 如果是受雇（FL001= 1），问：

FL009 What were the monthly wages, bonuses, and subsidies from this job，before you stopped working at this job? 您停止做这份工作前，平均每月总共挣多少钱（包括工资、奖金和补贴）？_____Yuan 元
[IWER: Mark 0 if there is no net income, and mark 999997 if running a deficit. 访员注意：如果没有净收入请标注 0，如果是亏损请标注 999997.] [Soft Check: Soft Check: Prompt for Verification if FL009 < 100]

FL010 [IWER: If R is unwilling to answer or does not remember, ask unfolding bracket questions here. 访员注意：如果受访者不愿回答或者忘记了，在此处分级展开提问:] 500 /1,000 /2,500 /5,000 /10,000 yuan 元

FL011 What was the value of other bonuses not paid with regular wages each year? 您从单位拿到的其他所有奖金（不和薪酬一起按期支付，比如年终奖等）每年一共有多少钱?
______Yuan 元

FL011_bracket [IWER: If R is unwilling to answer or does not remember, ask unfolding bracket questions here. 访员注意：如果受访者不愿回答或者忘记了，在此处分级展开提问:]
500 /1,000 /2,500 /5,000 /10,000 yuan 元

PROCEDURE 程序:
Ask FL012 if self-employed (FL001 = 2). 如果是自雇（FL001 = 2），问 FL012。
Otherwise skip to FL013. 否则跳至FL013

FL012 Do you have employees? 您有雇员吗?
1. Self-employed with employees 有
2. Self-employed without employees 没有
3. Family business worker without pay 家里人一起做，不付工资

PROCEDURE 程序:
Ask FL013–FL017 only if employed (FL001 = 1). 对于问题FL013–FL017，只向受雇人群提问（FL001 = 1）。
Otherwise skip to FL020. 否则跳至FL020。

FL013 Were you a regular worker, a temporary worker, or a casual worker? 您是正式员工、合同制员工、临时工，还是小时工?
1. Regular wage worker 正式员工
2. Contract worker 合同制员工
3. Temporary wage worker 临时工
4. Casual wage worker 小时工

FL014 Did you work for the government, institution, firm, NGO, individual farmer or a resident household? 您是为政府机构、事业单位、企业单位、个体户、非赢利机构（如社团、协会、学会等)、农户，还是居民户工作?
1. Government 政府部门
2. Institutions 事业单位 → Skip to FL017 跳至FL017
3. NGO 非营利机构（如社团、协会、学会等）→ Skip to FL017 跳至FL017
4. Firm 企业 Skip to → FL016 跳至FL016
5. Individual firm 个体户 → Skip to FL017 跳至FL017
6. Individual farmer 农户 → Skip to FL017 跳至FL017
7. Individual household 居民户 → Skip to FL017 跳至FL017
8. Other 其他 → Skip to FL017 跳至FL017

PROCEDURE 程序:
If government employee (FL014= 1), ask FL015. 如果是政府雇员，问FL015

FL015 Were you a civil servant? 您是公务员吗?

1. Yes 是
2. No 否

**PROCEDURE 程序**:
If firm (FL014= 1), ask FL016. 如果在企业工作 (FL014= 1), 问FL016<br>Otherwise skip to FL017. 否则跳至FL017

[Show Card 出示卡片 20]

FL016 What was the ownership type of the business? 您的企业的所有制类型是?

1. 100% State owned firm 国有企业
2. State-controlled firm 国有控股企业
3. 100% Collective-owned firm 集体所有制企业
4. Collective-controlled firm 集体控股企业
5. 100% Private firm 私营/个体
6. Private-controlled firm 私人控股企业
7. 100% foreign-owned 外商独资
8. Joint venture 中外合资
9. Other joint-ownership 其他联营企业
10. Other 其他

FL017 What sort of work did you do? 您自己具体从事什么工作? ______(**FL017_1**)(例 1:餐厅面点厨师;例 2:生产线绕线工人)是否有相关的职业资格证书? ____**FL017_2**)(例 1:有高级中式烹调师证;例 2:没有职业资格证书)

[IWER: Ask the specific work. 访员注意:请详细填写具体工作内容。注意:回答中请不要用逗号。]

**PROCEDURE 程序**:
If R has processed retirement [FB011= 1], ask FL018. 访员注意:如果受访者办理了退休手续,请回答FL018]

FL018 Is this the business/organization where you processed retirement? 您是在这个单位办的退休手续吗?

1. Yes 是 → Skip to FL021 跳至FL021
2. No 否 → Skip to FL020 跳至FL020

**PROCEDURE 程序**:
If R has not processed retirement [FB011= 2], ask FL019. 访员注意:如果受访者没有办理退休手续,请回答FL019]

FL019 Do you expect to process retirement from this business/organization? 您预期将来从这个单位或以自营职业的身份办理退休手续吗?

1. Yes 是
2. No 否

FL020 Why did you leave that employer? 您为什么离开这个工作单位？或者您为什么不继续您的生意或务农了，或者您为什么不再为家庭经营活动帮工了呢？
[IWER: Do not probe but check all that apply. 访员注意：不用深究，请选择全部适用项。]
1. Business closed 单位破产／倒闭了 (**FL020s1**)
2. Quit 辞职 (**FL020s2**)
3. I was laid off 下岗 (**FL020s3**)
4. I was fired 被辞退 (**FL020s4**)
5. I went to school 我上学了 (**FL020s5**)
6. I went abroad 我出国了 (**FL020s6**)
7. I stopped working for health reasons 因为健康原因中断工作 (**FL020s7**)
8. I stopped working for family reasons 因为家庭原因中断工作 (**FL020s8**)
9. I was transferred to another job 工作调动 (**FL020s9**)
10. I was sent down to the countryside to do manual labor 上山下乡 (**FL020s10**)
11. I started working off-farm locally 我在本地从事非农工作了 (**FL020s11**)
12. I went to work away from home 我外出打工了 (**FL020s12**)
13. Better job in local area 本地更好的新工作 (**FL020s13**)
14. Better job in another location 外地更好的新工作 (**FL020s14**)
15. I retired 我退休了 (**FL020s15**)
16. Other 其他 (**FL020s16**)

FL021 Did you receive any payments other than the legal retirement allowance upon leaving your last job? (For example, condolence payment，workers compensation, etc.) 上一份工作结束时，除去退休工资，您是否还得到了其他补偿（如买断工龄，伤残津贴等）？
1. Yes 是
2. No 否

F1：“买断工龄”指改革开放初期我国一些国有企业在改革过程中安置富余人员的一种办法，即参照员工在企业的工作年限、工资水平、工作岗位等条件，结合企业的实际情况，经企业与员工双方协商，报有关部门批准，由企业一次性支付给员工一定数额的货币，从而解除企业和富余员工之间的劳动关系，把员工推向社会的一种形式。

PROCEDURE 程序:
If FL021 = 1, ask FL022. 如果 FL021= 1，问 FL022

FL022 How much was the compensation and for how many years of work?____Yuan 元 ____years 您得到了多少补偿?____ (**FL022_1**) 元；是按照多少工龄算的？ ____0...120 (**FL022_2**) 年

FL022_bracket [IWER: If R is unwilling to answer or does not remember, ask unfolding bracket questions here. 访员注意：如果受访者不愿回答或者忘记了，在此处分级展开提问]: 1000 /2000 /5000 /10,000 /20,000 yuan 元

FM RETIREMENT 退休与退职

PROCEDURE 程序:

If R has processed retirement [FB011 = 1 or FB012 = 1 or ZF15 = 1 or ZF16 = 1], ask FM001 –FM059 . 如果受访者已经办了退休手续 [FB011 = 1 或 ZF15 = 1] 或者退职手续 [FB012 = 1 或 ZF16 = 1]，提示受访者，您刚才告诉我们您已经办理了退休/退职手续，下面是关于您的退休/退职手续的一些问题，并提问FM001 –FM059

FM001_1 BRANCHPOINT :

如果老受访者上一轮调查时已经办理了退职（ZF16= 1），依次检查 ZF25_1 ZF25_2 ZF25_3，如果 ZF25_1= 1，则询问FM004，如果 ZF25_2= 1，则询问FM005，如果 ZF25_3= 1，则询问FM007。之后请跳至FM001_2 BRANCHPOINT

如果老受访者上一轮调查时已经办理了 [正式/提前] 退休（ZF25_4= 1），依次检查 ZF25_1 ZF25_5 ZF25_6，如果 ZF25_1= 1，则询问FM004，如果 ZF25_5= 1，则询问FM014，如果 ZF25_6= 1，则询问FM016-FM017。之后请跳至FM001_2 BRANCHPOINT

如果老受访者上一轮调查时已经办理了内退（ZF25_8= 1），依次检查 ZF25_1 ZF25_9 ZF25_10 ZF25_11，如果 ZF25_1= 1，则询问FM004，如果 ZF25_9= 1，则询问FM025，如果 ZF25_10= 1，则询问FM027，如果 ZF25_11= 1，则询问FM028-FM029。之后请跳至FM001_2 BRANCHPOINT

FM001_2 BRANCHPOINT :

If old R had completed receding position procedure prior to the last IW (ZF16= 1), or had completed regular retirement in the last IW (ZF16= 1), skip to FM052 BRANCHPOINT. 如果老受访者上一轮调查时已经办理了退职，或上一轮调查时已经办理了正式退休，跳至FM052 BRANCHPOINT.

If old R had completed internal retirement but not yet regular retirement prior to the last IW（ZF23= 1），skip to FM037_w2 BRANCHPOINT. 如果老受访者上一轮调查时已经办理了内退但是还没有办理正式退休，跳至FM037_w2 BRANCHPOINT。

If old R had not completed retirment procedure or receding position procedure prior to the last IW (ZF24= 1), continue with FM001 . 如果老受访者上一轮调查时还没有办理退休或退职手续，询问FM001 .

All new R continues with FM001 所有的新受访者，询问FM001 。

FM001 Which of the following is the work unit that processed your [preload: retirement /receding position] 给您办理 [退休/退职] 手续的单位是下面的哪一个？

[IWER: If R has no work unit (no answer to FD003 or FL027), please choose “(3) None of the above”. 访员注意：如果此受访者没有工作单位，请选择“(3）以上都不是”，并继续]

1. Current work unit 现在的工作单位 [for new R: preload FD003; for old R: ZF8 if FA006_w2_2a= 1 & FA006_w2_2a_3= 1, or FA006_w2_2a_1= 1 if FA006_w2_2a= 2 & FA006_w2_2a_3= 1, or FM003 if FA006_w2_2a_3= 2] → Skip to procedure before FM005 请跳至FM005 前的程序控制
2. Last work unit [preload FL005 or ZF25] FL005 中提到的上一份工作的单位 [下载FL005 or ZF25] → Skip to procedure before FM005 请跳至FM005 前的程序控制

3. None of the above 以上都不是

PROCEDURE 程序:
If answer to FM001is "(3) None of the above", then ask FM002-FM004 : 如果 FM001回答" (3) 以上都不是", 问FM002-FM004。

FM002 What is the name of the employer that processed your [preload: retirement /receding position]? 给您办理 [退休/退职] 手续的单位的名称是什么? ________

FM003 What was the type of your work unit at [preload: retirement /receding position]? 您办理 [退休/退职] 的单位是哪种类型的?

1. Government 政府部门
2. Institutions 事业单位
3. NGO 非营利机构（如社团、协会、学会等）
4. Firm 企业
5. Individual firm 个体户
6. Farmer 农户
7. Individual household 居民户
8. Other 其他

FM004 Where is this work unit located? 这个单位的地址是?

1. The same as permanent address 和目前常住地一样
2. Another village/neighborhood in permanent address's county/city/district 常住地所在县/市/区的其他村/社区 ____ (**FB004_1**) village/neighborhood 村/社区
3. Other 其它: ____(**FB004_2**) province_city_county/city/district 省 _ 市 _ 县/市/区, ____(**FB004_3**) village/neighborhood 村/社区
4. Abroad 国外

[IWER: 访员注意: 如果记不清详细的地址名称, 请在相应输入框中填入"其它"。]

PROCEDURE 程序:
If FB012= 1 (Receding), ask FM005-FM009 如果FB012= 1（受访者退职），询问 FM005-FM009

FM005 In what month and year did you recede from your position? 您是在哪年哪月办理的退职? ____ (**FM005_1**) 1900...2013 year 年 __ (**FM005_2**) 0...12 month 月

[IWER: Mark the year using four digits. Take down the month as its actual number. For example, write January as "1" not "01", December as "12". If do not remember month, fill '0'. 访员注意: 用 4 位数表示年, 按照实际的月份填写月。例: 1 月写作 "1", 而不是 "01" ,12 月写作 "12"。如果记不住月份, 请填入' 0'。]

FM006 What was the main reason you receded from your position? 您办退职的主要原因是?

1. Due to poor health, I couldn't continue my work any more, at the same time I wasn't eligible for retirement 身体原因不能继续工作，而又不符合退休条件

2. Years of eligible work are less than three, and time of stopping work due to diseases or injures not related to work are more than one year 连续工龄不满三年，因病或非因工负伤而停止工作的时间满一年
3. I'm recruit worker within 6 months, but I had serious chronic disease once and can't tstick to work any more 录用后在六个月以内，发现原来有严重慢性疾病，不能坚持工作的
4. I receded from my position voluntary 自愿退职
5. Reach retirement age, but not eligible working age. 到了退休年龄，但工龄不够
6. Other 其他

FM007 Your pre-receding total salary was____ Yuan a month (including basic wage, bonus, et. al). 您退职前的总工资是每月多少钱？ ____ 元/月，包括基本工资和奖金等。

FM007_bracket [IWER: If R is unwilling to answer or does not remember, ask unfolding bracket questions here. 访员注意：如果受访者不愿回答或者忘记了，在此处分级展开提问]: 500 /1,000 /2,000 /3,500 /5,000 yuan 元

FM008 Did you receive any payments for leaving your job? 您是否得到了退职补偿？

1. Yes 是
2. No 否

PROCEDURE 程序:

If FM008 = 1, ask FM009. 如果 FM008 = 1，问 FM009

FM009 How much was the compensation?____ Yuan 元您得到了多少补偿?____ 元;

FM009_bracket [IWER: If R is unwilling to answer or does not remember, ask unfolding bracket questions here. 访员注意：如果受访者不愿回答或者忘记了，在此处分级展开提问]: 500 /1,000 /2,000 /3,500 /5,000 yuan 元

PROCEDURE 程序:

If FB011 = 1 (Retirement), ask FM011-FM041. 如果 FB011= 1（受访者退休），询问FM011-FM041。

Otherwise skip to FM042, 否则跳至FM042

FM011 Was your retirement normal retirement; early retirement; or internal retirement initially, followed by regular retirement? 您办的是正常退休，提前退休还是先内退然后办的正式退休或者将来再办正式退休？

1. Normal retirement 正常退休
2. Early retirement 提前退休
3. Internal retirement first, then regular retirement 先内退然后又正式退休
4. Internal retirement, but not yet regular retirement 内退但目前还没有办理正式退休

FM012 Did you retire as a worker or as a cadre? 您退休时的身份是干部还是工人？

1. Worker 工人
2. Cadre 干部

PROCEDURE 程序:
If year of first job was before 1952 (compute from FB001), then ask FM013. 如果第一份工作是在 1952 年之前，问FM013。

FM013 Are you an ordinary retiree or revolutionary retiree? 您现在是退休还是离休？
1. Ordinary retiree 退休
2. Revolutionary retiree 离休

F1："离休"指对建国前参加中国共产党所领导的革命战争、脱产享受供给制待遇的和从事地下革命工作的老干部，达到离职休养年龄的，实行离职休养的制度。已经退休的干部，符合上述条件的，应当该为离休。

PROCEDURE 程序:
If FM011 = 1 or FM011 = 2 , ask FM014-FM024. 如果 FM011 = 1/2，问 FM014-FM024。

FM014 In what month and year did you take [preload: normal/early] retirement? 您办理 [正式/提前] 退休手续是在哪年哪月？____ 1900...2013 (**FM014_1**) year 年 __0...12 (**FM014_2**) month 月

[IWER: Mark the year using four digits. Take down the month as its actual number. For example, write January as "1" not "01", December as "12". If do not remember month, fill '0'. 访员注意：用 4 位数表示年，按照实际的月份填写月。例：1 月写作"1"，而不是"01"，12 月写作"12"。如果记不住月份，请填入'0'。]

Soft Check: Prompt for Verification/Correction if Age of Early Retirement is Young, e.g. Prompt if ((FM014_1 +FM014_2 /12)-(CV009_a+CV009_b/12) < 45 & CV004==2) | ((FM014_1 +FM014_1 /12)-(CV009_a+CV009_b/12) <50 & CV004== 1)

PROCEDURE 程序:
Ask FM015 if FM011 = 2 (early retirement). 如果提前退休，问FM015

FM015 What was the main reason you processed early retirement? 您办提前退休的主要原因是？
1. I have 30 years job experience, which is enough for early retirement. 我的工龄达到了 30 年，可以提前退休。
2. My work unit belonged to the category of high-risk and hard manual labor and thus was eligible for offering early retirement 单位是高风险、高强度的工作，允许提前退休
3. My work unit was restructuring/bankrupt, so it offered early retirement 我的工作单位要破产、已经破产或者正在重组因此允许提前退休
4. Due to poor health 健康原因
5. Due to family reason 家庭原因
6. Other 其他

FM016 Your pre-retirement salary was____ Yuan a month (including bonus and subsidy, et. al). 您退休前的工资，包括奖金和各种补贴每月多少钱？____ 元/月。

FM017 [IWER: If R is unwilling to answer or does not remember, ask unfolding bracket questions here. 访员注意：如果受访者不愿回答或者忘记了，在此处分级展开提问]: 500 /1,000 /2,500 /5,000 /10,000 yuan 元

FM024 How many years of eligible work did you have at the time of retirement? 您退休时按多少年工龄计算？____0.00...100.00 Years 年

**PROCEDURE 程序**:<br>Ask FM025-FM029 if FM011 = 3or FM011 = 4 (internal retirement). 如果 FM011=3/4, 如果内退，问FM025-FM029<br>Otherwise skip to FM042, 否则跳至 FM042

FM025 In what month and year did you take internal retirement? 您是在哪年哪月办理的内退？____ 1900...2013 (**FM025_1**) year 年 __0...12 (**FM025_2**) month 月

[IWER: Mark the year using four digits. Take down the month as its actual number. For example, write January as "1" not "01", December as "12". If do not remember month, fill '0'. 访员注意：用 4 位数表示年，按照实际的月份填写月。例：1 月写作 "1"，而不是 "01" ,12 月写作 "12"。如果记不住月份，请填入' 0'。]

Soft Check: Prompt for Verification/Correction if Age of Retirement is Young, e.g. Prompt if (FM030_1 +FM030_2 /12)-(CV009_a+CV009_b/12) <45 & CV004==2) | ((FM030_1 + FM030_2 /12)-(CV009_a+CV009_b/12) <50 & CV004==1)

FM026 What was the main reason you processed internal retirement? 您办理内退的主要原因是？

1. 5years less than the legal retirement age 距法定退休年龄不足 5 年。
2. My work unit was restructuring/bankrupt 我的工作单位要破产、已经破产或者正在重组
3. Due to poor health 健康原因
4. Due to family reason 家庭原因
5. Other 其他

FM027 Your pre-internal retirement salary was ____ Yuan a month everything included. 您内退前的工资、奖金全部加起来是平均每月多少钱?____ 元/月。

FM027_bracket [IWER: If R is unwilling to answer or does not remember, ask unfolding bracket questions here. 访员注意：如果受访者不愿回答或者忘记了，在此处分级展开提问]: 500 /1,000 /2,000 /3,500 /5,000 yuan 元

FM028 How much was the internal retirement wage (everything included) when you processed internal retirement? 您内退的时候，工资、奖金全部加起来每个月平均能拿到多少？____ 元/月。

FM029 [IWER: If R is unwilling to answer or does not remember, ask unfolding bracket questions here. 访员注意：如果受访者不愿回答或者忘记了，在此处分级展开提问]: 500 /1,000 /2,000 /3,500 /5,000 yuan 元

PROCEDURE 程序:
Ask FM030–FM036 if FM011 = 3. 如果 FM011= 3, 问FM030–FM036

FM030 In what month and year did you process formal retirement? 您在哪年哪月办理的正式退休手续? ____ 1900...2013 (**FM030_1**) year 年 __0...12 (**FM030_2**) month 月
[IWER: Mark the year using four digits. Take down the month as its actual number. For example, write January as "1" not "01", December as "12". If do not remember month, fill '0'. 访员注意: 用 4 位数表示年，按照实际的月份填写月。例：1 月写作"1"，而不是"01" ,12 月写作"12"。如果记不住月份，请填入' 0'。]
Soft Check: Prompt for Verification/Correction if Age of Retirement is Young, e.g. Prompt if ((FM030_1 +FM030_2 /12)-(CV009_a+CV009_b/12) <45 & CV004==2) | ((FM030_1 +FM030_2 /12)-(CV009_a+CV009_b/12) <50 & CV004==1)

FM036 How many years of eligible work did you have at the time of formal retirement? 您正式退休时计算了多少年工龄? ____0.00...100.00 Years 年

PROCEDURE 程序:
Skip to FM042 跳至FM042

FM037_w2 BRANCHPOINT :
If R had completed internal retirement but not yet regular retirement prio to the last IW (ZF23= 1), then ask FM037_w2- FM040_w2. 如果上一轮调查时受访者已经办理了内退但是还没有办理正式退休，询问 FM037_w2至FM040_w2.。

FM037_w2 Did you proceed formal retirement? 您是否已经办理正式退休手续?
1. Yes 是
2. No 否 → Skip to FM037 跳至FM037

FM038_w2 In what month and year did you process formal retirement? 您在哪年哪月办理的正式退休手续? ____ 1900...2013 (**FM038_w2_1_1**) year 年 __0...12 (**FM038_w2_1_2**) month 月
[IWER: Mark the year using four digits. Take down the month as its actual number. For example, write January as "1" not "01", December as "12". If do not remember month, fill '0'. 访员注意: 用 4 位数表示年，按照实际的月份填写月。例：1 月写作"1"，而不是"01" ,12 月写作"12"。如果记不住月份，请填入' 0'。]
Soft Check: Prompt for Verification/Correction if Age of Retirement is Young, e.g. Prompt if ((FM030_1 +FM030_2 /12)-(CV009_a+CV009_b/12) <45 & CV004==2) | ((FM030_1 +FM030_2 /12)-(CV009_a+CV009_b/12) <50 & CV004==1)

FM040_w2 How many years of eligible work did you have at the time of formal retirement? 您正式退休时计算了多少年工龄？ ____0.00...100.00 Years 年

Skip to FM042 跳至FM042

> **PROCEDURE 程序:**
> Ask FM037–FM040 if FM011 = 4 or FM037_w2 = 2. 如果FM011 = 4 或者 FM037_w2 = 2，问FM037–FM040
> Otherwise Skip to FM042. 否则跳至FM042。

FM037 In what month and year are you going to process formal retirement? 您将会在哪年哪月办理正式退休手续？ ____ 1900...2013 (**FM037_1**) year 年 __0...12 (**FM030_2**) month 月

[IWER: Mark the year using four digits. Take down the month as its actual number. For example, write January as "1" not "01", December as "12". If do not remember month, fill '0'. 访员注意：用 4 位数表示年，按照实际的月份填写月。例：1 月写作"1"，而不是"01" ,12 月写作"12"。如果记不住月份，请填入' 0'。]

Soft Check: Verify if Age of Respondent will be outside the legal retirement range, e.g., Prompt for verification if ((FM037_1 + FM037_2/12) - (CV009_a + CV009_b/12) < 50 | (FM037_1 + FM037_2/12) - (CV009_a + CV009_b/12) > 55) & CV004 == 2) | (FM037_1 + FM037_2 /12) - (CV009_a + CV009_b/12) < 55 | (FM037_1 + FM037_2/12) - (CV009_a + CV009_b/12) > 60) & CV004 ==1)

FM040 How many years of eligible work will you have at the time of retirement? 您正式退休时预计将计算多少年工龄？ ____0.00...100.00 Years 年

FM041 How many years of eligible work do you currently have? 您现在已经有多少年工龄了？ ____0.00...100.00 Years 年 [FM041 > FM040]

> **PROCEDURE 程序:**
> Skip (FM042–FM051) if FM037_w2 = 2. 如果FM037_w2 = 2，跳过FM042–FM051

FM042 Did you have a spouse when you processed [preload: normal retirement / early retirement / internal retirement / receding position]? 您办理手续时是否有配偶？

[正式退休/提前退休/内退/退职 (注：从FM011处加载。如果 FM037_w2 = 1，则此处加载"正式退休"。下同)]

1. Yes 是
2. No 否

FM043 How was your health at the time of your [preload: normal retirement / early retirement / internal retirement / receding position], excellent, very good, good, fair or poor? 您 [正式退休/提前退休/内退/退职] 时您的身体状况如何？是极好、很好、好、一般还是不好?

1. Excellent 极好
2. Very good 很好

3. Good 好
4. Fair 一般
5. Poor 不好

PROCEDURE 程序:
Skip FM044–FM046 if FM042 = 2. 如果 FM042 = 2，跳过 FM044–FM046

FM044 Had your spouse already processed retirement when you processed [preload: normal retirement / early retirement / internal retirement / receding position]? 您办理 [正式退休/提前退休/内退/退职] 时配偶是否已经办理了退休手续？
1. Yes 是
2. No 否

FM045 What kind of economic activities was your spouse engaged in at the time of your [preload: retirement / receding position]? 您 [退休/退职] 时配偶在从事哪种经济活动？
1. Employed by another person or company and received a wage 受雇于某公司或个人，领取工资
2. Ran own business 从事个体或者私营经济活动
3. Non-employed and looking for a job 没有工作但是在找工作
4. Non-employed and not looking for a job，or only doing household work 没有工作也没有找工作，或仅仅在家作家务
5. Farming 务农

FM046 How was your spouse's health at the time of your [preload: normal retirement / early retirement / internal retirement / receding position], excellent, very good, good, fair or poor? 您 [正式退休/提前退休/内退/退职] 时您配偶的身体状况如何？是极好、很好、好、一般还是不好?
1. Excellent 极好
2. Very good 很好
3. Good 好
4. Fair 一般
5. Poor 不好

FM047 Was your father alive at the time of your [preload: normal retirement / early retirement / internal retirement / receding position]? 您 [正式退休/提前退休/内退/退职] 时，您父亲还健在吗？
1. Yes 是
2. No 否 → Skip to FM049 跳至FM049

FM048 How about the health of your father at the time of your [preload: normal retirement / early retirement / internal retirement / receding position], excellent, very good, good, fair or poor? 您 [正式退休/提前退休/内退/退职] 时您父亲的身体状况如何？是极好、很好、好、一般还是不好?

1. Excellent 极好
2. Very good 很好
3. Good 好
4. Fair 一般
5. Poor 不好

FM049 Did your mother alive when your [preload: normal retirement / early retirement / internal retirement / receding position]? 您 [正式退休/提前退休/内退/退职] 时，您母亲还健在吗？

1. Yes 是
2. No 否 → Skip to FM051 跳至FM051

FM050 How about the health of your mother at the time of your [preload: normal retirement / early retirement / internal retirement / receding position], excellent, very good, good, fair or poor? 您 [正式退休/提前退休/内退/退职] 时您母亲的身体状况如何？是极好、很好、好、一般还是不好?

1. Excellent 极好
2. Very good 很好
3. Good 好
4. Fair 一般
5. Poor 不好

FM051 How many grandchildren below age 6 did you have at the time of your [preload: normal retirement / early retirement / internal retirement / receding position]? 您 [正式退休/提前退休/内退/退职] 时有几个六岁以下的孙子女或者外孙子女？ ____0...50 persons 人

[IWER: if none, fill '0'. 访员注意：如果没有请填“0”]

FM052 BRANCHPOINT:

For old R who had processed receding position procedure in the last IW (ZF16 = 1) or had processed internal but not regular retirement in the last IW (ZF23 = 1), or had processed regular retirement in the last IW (ZF26 = 1): if old R had not worked after processing retirement/receding position procedure until the last IW (ZF26 = 1) and is currently not working (XF1 = 2), ask FM052_w2; if old R had not worked after processing retirement/receding position procedure until the last IW (ZF27 = 1) and is currently working (XF1 = 1), ask FM053. 上一轮时已经办理退职、或者已经办理内退但是还没有办理正式退休、或者已经办理正式退休手续的老受访者：如果老受访者办理退休/退职后到上一轮调查时没有工作过并且现在不工作，询问FM052_w2；如果老受访者办理退休/退职后到上一轮调查时没有工作过但是现在在工作，询问FM053。其他老受访者，询问FM052_w2。
New R 新受访者：If R is currently not working (FA001 = 2 & FA002 = 2 & FA003 = 2), ask FM052. 如果受访者现在不在工作（FA001 = 2 & FA002 = 2 & FA003 = 2），问：FM052。If FA052 = 1 or If R is currently working, ask FM053. 如果受访者退休后工作过或者现在仍在工作（FA052 = 1 or FA001 = 1 or FA002 = 1 or FA003 = 1），跳至FM053。

FM052 Did you work after you processed [preload: normal retirement / early retirement / internal retirement / receding position]? We consider any of the following activities to be work: agricultural work, earn a wage, run your own business and unpaid family business work, et. al. Work does not include doing your own housework or doing activities without pay, such as voluntary work. 在您办理了 [正式退休/提前退休/内退/退职] 之后，您是否工作过？务农、挣工资工作、从事个体、私营经济活动或不拿工资为家庭经营活动帮工都算是工作，但不包括家务劳动、义务的志愿劳动。

1. Yes 是 → Skip to FM053 跳至 FM053
2. No 否 → Skip to FM054 跳至FM054

FM052_w2 Did you work in the last two years? We consider any of the following activities to be work: agricultural work, earn a wage, run your own business and unpaid family business work, et.al. Work does not include doing your own householdwork or doing activities without pay, such as voluntary work. 过去两年您工作过吗？挣工资工作、从事个体、私营经济活动或不拿工资为家庭经营活动帮工都算是工作，但不包括家务劳动、义务的志愿劳动

1. Yes 是
2. No 否 → Skip to FM054 跳至FM054

FM053 After you processed [preload: normal retirement / early retirement / internal retirement / receding position] How long did you start to work again? 在您办理了 [正式退休/提前退休/内退/退职] 之后，您过了多长时间又开始工作的？ ____0.00...100.00 Years 年 (allow for decimal points 允许小数点)

**PROCEDURE 程序**:<br>Skip to FN001_w2 跳至FN001_w2

FM054 Are you currently engaged in paid small pastime work? 您目前是否从事小的消遣性的工作但是也有收入？

1. Yes 是
2. No 否 → Skip to FN001_w2 跳至FN001_w2

FM055 What kind of pastime job are you engaged in? 您从事哪种消遣性的工作?__________
[IWER: Ask the specific work. 访员注意：请详细填写具体工作内容。注意：回答中请不要用逗号。]

FM056 When did you start this job? 您从什么时候开始做这项工作的？____ 1900...2013 (**FM056_1**) year 年 __0...12 (**FM056_2**) month 月
[IWER: Mark the year using four digits. Take down the month as its actual number. For example, write January as "1" not "01", December as "12". If do not remember month, fill '0' . 访员注意：用 4 位数表示年，按照实际的月份填写月。例：1 月写作 "1"，而不是 "01"，12 月写作 "12"。如果记不住月份，请填入' 0'。]

FM057 How many days per week do you usually work for your pastime job? 您一般每周做几天？An average of __ days per week 平均每周 __0...7 天

FM058 How many hours per week do you usually work at your pastime job? 您一般每周做几个小时？An average of ____ hours per week 平均每周 ____0.00...168.00 小时

FM059 What is your monthly income from the pastime work? 您每月能挣多少钱？____ YUAN 元
[IWER: Mark 0 if there is no net income, and mark 999997 if running a deficit. 访员注意：如果没有净收入填 0，如果每月亏损填 999997。]

FM059_bracket [IWER: If R is unwilling to answer or does not remember, ask unfolding bracket questions here. 访员注意：如果受访者不愿回答或者忘记了，在此处分级展开提问]: 500 /1,000 /2,000 /3,500 /5,000 yuan 元

FN PENSION INSURANCE 养老保险

[Intro: Next we' ll ask you some questions about your pension insurance. It' s important to assess existing pension policy and revise it in the future. 引语：下面我们将问一些有关您的养老保险的问题，这些问题对于评估现有的养老政策以及制定将来的养老政策有着重要意义。]

[Show Card 出示卡片 23]

FN001_w2 Are you currently participating in or receiving at least one kind of pension as followings? Pension here refers to income from such pension programs as supplemental pension insurance of the firms, residents' pension insurance, rural pension insurance, Urban residents' pension and commercial pension insurance, and pension subsidy for the oldest old and so on? (choose all that apply) 您现在参加或领取以下一种或几种退休金/养老金吗？（可多选）
[IWER: Read out all the choices. 访员注意：读出所有选项。]

1. 政府机关和事业单位退休金
2. 企业职工基本养老保险
3. Supplemental pension insurance of the firm 企业补充养老保险（企业年金）
4. Commercial pension 商业养老保险
5. 人寿保险
6. Rural pension 农村养老保险（老农保）
7. Residents' pension 城乡居民社会养老保险
8. Urban residents' pension 城镇居民养老保险
9. New Rural Social Pension Insurance 新型农村社会养老保险（新农保）
10. 征地养老保险（失地养老保险）
11. Pension subsidy to the oldest old 高龄老人养老补助（补贴）
12. Other 其他
13. 没有参加或领取任何养老金

[F1：(1)“政府机关和事业单位退休金”指受访者从政府和事业单位领取的退休金。目前，我国大部分政府机构和事业单位，都还没有实行社会养老保险制度，仍实行退休养老制度。这一制度下，一般不需要个人在退休前缴纳养老保险金。(2)“企业职工基本养老保险”是指受访者参加社会基本养老保险制度，达到退休年龄并符合领取条件后，领取到社会养老保险金。(3)“企业补充养老保险”也叫企业年金，是指企业及其职工在依法参加基本养老保险的基础上，自愿建立的补充养老保险制度。(4)“商业养老保险”是指受访者直接向商业保险公司投保，定期缴纳保险费，从合同约定年龄开始持续、定期地领取养老金的人寿保险，能有效地满足客户的养老需要。(5)“农村养老保险”(老农保)是指农村社会养老保险。1992 年民政部颁布了《县级农村社会养老保险基本方案》，确定以县为基本单位开展农村社会养老保险。(6)“城乡居民社会养老保险”指为推进城乡一体化，某些地区将城镇居民社会养老保险和新型农村社会养老保险统筹安排、合并实施，称为“城乡居民社会养老保障制度”。(7)“城镇居民养老保险”指通过建立城镇居民养老保险个人账户，实行个人缴费和财政补贴相结合的筹资模式，为目前未享受定期养老待遇或其他相关定期养老待遇的城镇居民提供的养老保险。(8)“新型农村社会养老保险”(新农保)采取个人缴费、集体补助和政府补贴相结合，其中中央财政将对地方进行补助，并且会直接补贴到农民头上。(9)“高龄老人养老补助（补贴)”是指 60 岁以上老人享有政策性的优惠，如乘车免费、挂号费免费、参观等免费、部分社区活动免费，如注射流感疫苗等，百岁以上老人才享有月补助金。]

Part 1 政府机关、事业单位退休金及企业职工基本养老保险

Ask all R 所有人回答：

FN002_w2 Are you currently receiving pension as follows (Check all that apply)? 您现在是否领取以下退休金/养老保险？(可多选)

1. 政府机关（公务员）退休金
2. 事业编制职工退休金
3. 企业职工基本养老保险
4. 以上都没有领取 → Skip to FN006_w2 跳至 FN006_w2

**PROCEDURE 程序**:
如果FN002_w2多选，则针对每一个选项，循环询问FN003_w2-FN005_w2_bracket.

FN003_w2 In what month and year did you start to receive you pension benefits? 您从什么哪年哪月开始领取这一养老金 (退休金) 的？____ 1900...2013 (**FN003_w2_1**) year 年 __0...12 (**FN003_w2_2**) month 月

[IWER: Mark the year using four digits. Take down the month as its actual number. For example, write January as “1” not “01”, December as “12”. If do not remember month, fill ‘0’. 访员注意：用 4 位数表示年，按照实际的月份填写月。例：1 月写作“1”，而不是“01”，12 月写作“12”。如果记不住月份，请填入’0’。]

FN004_w2 How much were the benefits (including subsidy) when you retired? 您办理正式退休的时候，退休金/养老金 (包括各种补贴) 是多少？____ Yuan per month 元/月。

FN005_w2 What is your monthly pension (including subsidy)? 您现在每月领多少退休金/养老金 (包括各种补贴)? _____ Yuan 元

[Soft Check: Verify if monthly benefits are low or high, e.g., prompt if FN005_w2 <200 yuan/month]

PROCEDURE 程序:

Skip to FN030_w2 跳至FN030_w2

FN006_w2 Are you currently enrolled in pension program of the government and institutions or basic pension of the firms? 您将来是否能够享受政府和事业单位的退休金待遇? 您目前是否参加了企业职工基本养老保险?

1. Yes, pension program of the government and institutions 是的，将来享受政府机关和事业单位的退休金待遇
2. Yes, basic pension insurance of the firms 是的，目前参加了企业职工基本养老保险
3. Yes, both of them. 是的，两个都在参加/能够享受。
4. No 否，都没有参加/不能享受 → Skip to FN022_w2 跳至 FN022_w2

[F1：（1）“政府机关和事业单位退休金”指受访者从政府和事业单位领取的退休金。目前，我国大部分政府机构和事业单位，都还没有实行社会养老保险制度，仍实行退休养老制度。这一制度下，一般不需要个人在退休前缴纳养老保险金。（2）“企业职工基本养老保险”是指受访者参加社会基本养老保险制度，达到退休年龄并符合领取条件后，领取到社会养老保险金。]

PROCEDURE 程序:

如果FN006_w2 = 3，循环询问FN007_w2–FN014_w2_bracket.

FN007_w2 From which of the following work units did you get the pension insurance you just told us? 将来给您提供的单位是下面的哪一个?

[preload “pension program of government and institutions” if FN006_w2 = 1; or preload “Basic pension insurance of the firms” if FN006_w2 = 2] [如果FN006_w2 = 1，下载“政府和事业单位退休金待遇”；如果FN006_w2 = 2，下载“企业职工基本养老保险”] [IWER: Choose “(4) None of the above” if the respondent has no work unit 访员注意：如果受访者没有单位，请选择“(4) 都不是”并继续]

1. Current work unit 现在的工作单位 [for new R: preload FD003; for old R: ZF8 if FA006_w2_2a = 1 & FA006_w2_2a_3= 1, or FA006_w2_2a_1 if FA006_w2_2a= 2 & FA006_w2_3= 1, or FD003 if FA006_w2_2a_3= 2] → Skip to FN011_w2 跳至 FN011_w2
2. Last work unit [preload FL005 or ZF25] FL005中提到的上一份工作的单位 [下载FL005 or ZF25] → Skip to FN011_w2 跳至 FN011_w2
3. The work unit that processed retirement for the respondent [preload FM002] FM002 中提到的办理退休手续的单位 [下载FM002] → Skip to FN011_w2 跳至 FN011_w2
4. None of the above 都不是

PROCEDURE 程序:

If answer to FN007_w2 = 4 (None of the above), then ask FN008_w2 – FN010_w2. 如果FN007_w2 = 4 (回答“都不是”)，请问 FN008_w2 – FN010_w2.

FN008_w2 What is the name of the unit that provides you this pension insurance? 将来给您提供这份退休金/养老保险的单位的名称是什么? ____________

FN009_w2 What was the type of the unit that provides you the pension insurance? 这个给您提供养老保险的单位是哪种类型的?

1. Government 政府部门
2. Institutions 事业单位
3. NGO 非营利机构（如社团、协会、学会等）
4. Firm 企业
5. Individual 个体或者私营企业主
6. Other 其他

FN010_w2 Where is this work unit located? 这个给您提供养老保险的单位在哪?

1. The same as permanent address 和目前常住地一样
2. Another village/neighborhood in permanent address's county/city/district 常住地所在县/市/区的其他村/社区 ____ (**FN010_1**) village/neighborhood 村/社区
3. Other 其它: ____ (**FN010_2**) province_city_county/city/district 省 _ 市 _ 县/市/区, ____ (**FN010_3**) village/neighborhood 村/社区
4. Abroad 国外

[IWER: 访员注意：如果记不清详细的地址名称，请在相应输入框中填入"其它"。]

PROCEDURE 程序:
如果当前询问的是政府机关和事业单位的退休金，请跳过FN011_w2

FN011_w2 In what month and year did you start to participate in the basic pension insurance of the firms through this work unit? 您是从哪年哪月开始在这个单位参加基本养老保险的? ____ 1900...2013 (**FN011_w2_1**) year 年 __0...12 (**FN011_w2_2**) month 月
[IWER: Mark the year using four digits. Take down the month as its actual number. For example, write January as "1" not "01", December as "12". If do not remember month, fill '0'. 访员注意: 用 4 位数表示年，按照实际的月份填写月。例: 1 月写作"1"，而不是"01"，12 月写作"12"。如果记不住月份，请填入'0'。]

FN012_w2 Do you need to pay the premium by yourself? 您参加的这份养老保险需要您个人或单位缴费吗?

1. Yes 需要
2. No 不需要 → Skip to FN015_w2 跳至 FN015_w2

FN013_w2 How much is the premium you paid? 您个人现在缴纳的保险费是多少? ____ (**FN013_w2_1**) Yuan per month 元/月 Or 或 ____ 0.00...100.00 (**FN013_w2_2**) percent 工资的比例

FN014_w2 How much the premium your unit paid? 单位现在为您缴纳的保险费是多少? ____ (**FN014_w2_1**) Yuan per month 元/月 Or 或 ____0.00...100.00 (**FN014_w2_2**) 工资的比例

PROCEDURE 程序:
Skip FN015_w2 -FN016_w2 if FN006_w2 = 1. 如果FN006_w2 = 1, 请跳过FN015_w2 - FN016_w2

FN015_w2 Before the above mentioned time, have you ever participated in basic pension insurance of the firms through other work unit for more than ten years? 在此之前，您有没有在其他地方参加企业职工基本养老保险超过 10 年?

1. Yes 有
2. No 没有 → Skip to FN017_w2 跳至 FN017_w2

FN016_w2 Among all of work units that you have participated in basic pension insurance of the firms for more than 10 years, in which province was the lastest work unit located? 在您之前参加的所有超过 10 年的企业职工基本养老保险中，最近的一次是在哪个省参加的? ____ Province 省/自治区/直辖市

FN017_w2 For how many years altogether have you been included in this program? [Include years with other employers if the same plan.] 到现在为止您可以用来计算养老金（退休金）的工龄一共有多少年? [不管雇主是否相同，只要能够累积计算工龄，都应算进去。]____0.00...100.00 Years 年

FN018_w2 For how many years altogether will you have been included in this program when you retire? [Include years with other employers if the same plan.] 您估计，到您退休时您可以用来计算养老金（退休金）的工龄一共有多少年? [不管雇主是否相同，只要能够累积计算工龄，都应算进去。]____0.00...100.00 Years 年

FN019_w2 Will this be enough years to receive pension? 这工龄足够您从上述养老保险项目中领取养老金吗?

1. Yes 是 → Skip to FN021_w2 跳至 FN021_w2
2. No 否

FN020_w2 What do you plan to do? 那您计划怎么办?

1. I will pay the remaining premiums all in one payment at retirement to qualify for pension 退休的时候，我会一次性支付剩余年限的保险金，这样就能领取养老金了。
2. I will receive a one-time payment at retirement and not get pension 退休的时候，我会得到一次性的补偿，以后就没有养老金了。→ Skip to FN030_w2 跳至 FN030_w2
3. I will not receive pension 我将拿不到养老金。→ Skip to FN030_w2 跳至 FN030_w2

PROCEDURE 程序:
If R answered yes to pension [FN019_w2 = 1 or FN020_w2 = 1], ask FN021_w2 - FN019_w2_bracket, then skip to FN030_w2. 如果被访者退休的时候，能领养老金 [FN019_w2= 1 or FN020_w2= 1], 请回答FN021_w2 - FN019_w2_bracket, 然后跳至 FN030_w2

FN021_w2 About how much do you expect your benefits to be? (as a percentage of your pay at retirement, or as an amount per month or year?) 您预期领多少钱的养老金？可以是退休前工资的百分比，也可以是每月多少钱。____ (**FN021_w2_1**) Yuan per month 元/月 Or 或 ____0.00..100.00 (**FN021_w2_2**)

FN021_w2_bracket [IWER: If R is unwilling to answer or does not remember, ask unfolding bracket questions here. 访员注意：如果受访者不愿回答或者忘记了，在此处分级展开提问]: 500 /1,000 /2,000 /3,500 /5,000 yuan 元

FN022_w2 Are you ever enrolled in pension program of the government and institutions or basic pension of the firms? 您刚刚说现在没有参加政府和事业单位的养老保险或者企业基本养老保险，请问您之前是否曾经参加过这两类保险？

1. Yes, pension program of the government and institutions 是的，曾经参加过政府和事业单位的养老保险
2. Yes, basic pension insurance of the firms 是的，曾经参加过企业职工基本养老保险
3. Yes 是的，两种都曾经参加过
4. No 否，两种都没有参加过 → Skip to FN030_w2 跳至FN030_w2

F1：“政府和事业单位的养老保险”指受访者从政府和事业单位领取的退休金。目前，我国大部分政府机构和事业单位，都还没有实行社会养老保险制度，仍实行退休养老制度。

FN023_w2 From which of the following work units did you get the pension insurance you just told us[preload “pension program of government and institutions” 您当时参加的单位是下面的哪一个？

Preload ”pension program of government and institutions” if FN022_w2 = 1; or preload “Basic pension insurance of the firms” if FN022_w2 = 2; 如果FN022_w2 = 1，下载“政府和事业单位养老保险”；如果FN022_w2 = 2，下载“企业职工基本养老保险”]

[IWER: Choose “(4) None of the above” if the respondent has no work unit 访员注意：如果受访者曾经多次参加过上述两类保险，请其填写参保时间最长的那次时所在的单位；如果受访者没有单位，请选择“(4) 都不是”并继续]

1. Current work unit 现在的工作单位 [for new R: preload FD003; for old R: ZF8 if FA006_w2_2a = 1 & FA006_w2_3 = 1, or FA006_w2_2a_1 if FA006_w2_2a = 2 & FA006_w2_3 = 1, or FD003 if FA006_w2_2a_3 = 2] → Skip to FN027_w2 跳至FN027_w2
2. Last work unit [preload FL005 or ZF25] FL005 中提到的上一份工作的单位 [下载FL005 or ZF25] → Skip to FN027_w2 跳至FN027_w2
3. The work unit that processed retirement for the respondent [preload FM002] FM002 中提到的办理退休手续的单位 [下载FM002] → Skip to FN027_w2 跳至FN027_w2
4. None of the above 都不是

PROCEDURE 程序:

If answer to FN023_w2 = 4 (None of the above), then ask FN024_w2 – FN026_w2. 如果FN023_w2 = 4 (回答“都不是”)，请问 FN024_w2 – FN026_w2

FN024_w2 What is the name of the unit that provides you this pension insurance? 给您提供这份养老保险的单位的名称是什么? ____________

FN025_w2 What was the type of the unit that provides you the pension insurance? 这个给您提供养老保险的单位是哪种类型的？

1. Government 政府部门
2. Institutions 事业单位
3. NGO 非营利机构（如社团、协会、学会等）
4. Firm 企业
5. Individual 个体或者私营企业主
6. Other 其他

FN026_w2 Where is this work unit located? 这个给您提供养老保险的单位在哪？

1. The same as permanent address 和目前常住地一样
2. Another village/neighborhood in permanent address's county/city/district 常住地所在县/市/区的其他村/社区 ____ (**FN026_w2_1**) village/neighborhood 村/社区
3. Other 其它: ____ (**FN026_w2_2**) province_city_county/city/district 省_市_县/市/区, ____ (**FN026_w2_3**) village/neighborhood 村/社区
4. Abroad 国外

[IWER: 访员注意：如果记不清详细的地址名称，请在相应输入框中填入“其它”。]

FN027_w2 In what month and year did you start to participate in the basic pension insurance of the firms through this work unit? 您是从哪年哪月开始在这个单位参加养老保险的？ ____ 1900...2013 (**FN027_w2_1**) year 年 __0...12 (**FN027_w2_2**) month 月

[IWER: Mark the year using four digits. Take down the month as its actual number. For example, write January as “1” not “01”, December as “12”. If do not remember month, fill ‘0’. 访员注意: 用 4 位数表示年，按照实际的月份填写月。例：1 月写作“1”，而不是“01”，12 月写作“12”。如果记不住月份，请填入’0’。]

FN028_w2 In what month and year did you start to participate in the basic pension insurance of the firms through this work unit? 您是在哪年哪月停止参加这份养老保险的？ ____ 1900...2013 (**FN028_w2_1**) year 年 __0...12 (**FN028_w2_2**) month 月

[IWER: Mark the year using four digits. Take down the month as its actual number. For example, write January as “1” not “01”, December as “12”. If do not remember month, fill ‘0’. 访员注意: 用 4 位数表示年，按照实际的月份填写月。例：1 月写作“1”，而不是“01”，12 月写作“12”。如果记不住月份，请填入’0’。]

FN029_w2 What was the reason for you to stop participating in this pension? 您停止参加这份养老保险的原因是？（Multiple choice 可多选）

1. Business closed 单位倒闭了
2. I changed job/I was fired 换工作了/被辞退了
3. I moved to other city, pension can not be transferred 更换工作城市，无法转移保险
4. I thought it was not worth and exited 感觉不划算，自愿退保

Part 2 企业补充养老保险

FN030_w2 Did you participate in supplemental pension insurance of the firm or receive the pension? 您是否参加了企业补充养老保险，或者正在领取企业补充养老保险金？

1. Yes, but I don't receive it 参加了，还未领取
2. Yes, I receive it 参加了，现在在领取
3. No 没有参加或领取企业补充养老保险 → Skip to FN043_w2 跳至FN043_w2

FN031_w2 Where did you participate in the insurance? 您在哪儿参加的这份保险？____ (**FN031_w2_1**) Province 省/自治区/直辖市 ____ (**FN031_w2_2**) city 市 ____ (**FN031_w2_3**) county 县

FN032_w2 What type of retirement pension plan is/was your employer's supplement pension? 您的单位选择的补充养老金属于哪种类型？

1. Defined Benefit (DB) Retirement Pension 收益确定型退休金
2. Defined Contribution (DC) Retirement Pension 缴费确定型退休金

DB Retirement Pension Plan: A worker's retirement pension is determined in advance and the amount paid by the user shall change based on how well the savings are managed. 收益确定性退休金计划：预先决定员工的养老金，使用者所支付的金额根据储蓄管理情况而定。
DC Retirement Pension Plan: The amount paid by the user is determined in advance and the retirement pension paid to a worker shall change based on how well the savings are managed. 缴费确定性退休金计划：预先决定使用者支付的金额，员工领取的退休金根据储蓄管理情况而定。

PROCEDURE 程序:

Skip FN033_w2 - FN040_w2_bracket if FN030_w2 = 2. 如果FN030_w2 = 2，跳过FN033_w2-FN040_w2_bracket

FN033_w2 For how many years altogether have you been included in this plan? [Include years with other employers if the same plan.] 您参加该保障制度多少年了？[包括受雇不同雇主但享受同一计划的年度。]____ 0.00...100.00 Years 年

FN034_w2 At what age do you expect to start receiving benefits from this plan? 您预期什么时候开始从这份保险中领取保险金？

1. At age 在 ____ 0...120 (**FN034_w2_1**) 岁 or in 或在 ____0...120 (**FN034_w2_2**) years 年之后
2. I do not expect receiving these benefits because I have received cash settlements 领取不到保险金，因为已被买断 → Skip to FN040_w2 跳至FN040_w2
3. I do not expect receiving these benefits because I have lost benefits 领取不到保险金，因为保险已经中断。→ Skip to FN043_w2 跳至FN043_w2
4. Other 其他 → Skip to FN043_w2 跳至FN043_w2

FN035_w2 What is the combined monthly contribution from you and your employer? Of which, how much do you pay? 每个月您和雇主一共缴纳多少钱？____ （**FN035_w2_1**） Yuan 元；其中，您个人支付部分是多少？____ 0.00...100.00 (**FN035_w2_2**) %

FN035_w2_bracket [IWER: If R is unwilling to answer or does not remember, ask unfolding bracket questions here. 访员注意：如果受访者不愿回答或者忘记了，在此处分级展开提问]: 500 /1,000 /2,000 /3,500 /5,000 yuan 元

**PROCEDURE 程序**:
If FN032_w2 = 1 ask: 如果 FN032_w2 = 1，问：

FN036_w2 For a DB plan, do you know how much you are entitled to at (age in FN034_w2)? 如果是收益确定性退休金计划，[开始领退休金的年龄，preload FN034_w2] 岁时，您知道每月可以领取多少钱？____ （**FN036_w2_1**） Yuan per month 元/月 Or 或 ____0.00...100.00 （**FN036_w2_2**） Or 或 ____ （**FN036_w2_3**） Yuan 元（Lump sum amount 一次结清）

FN036_w2_bracket [IWER: If R is unwilling to answer or does not remember, ask unfolding bracket questions here. 访员注意：如果受访者不愿回答或者忘记了，在此处分级展开提问]: 500 /1,000 /2,000 /3,500 /5,000 yuan 元

**PROCEDURE 程序**:
Skip to FN043_w2 跳至FN043_w2

**PROCEDURE 程序**:
If FN032_w2 = 2 ask: 如果FN032_w2 = 2，问：

FN037_w2 For a DC plan, have you ever checked your account balance? 如果是缴费确定型退休金计划，您有没有查看过账户余额？

1. Yes 有，____ （**FN037_w2_1**） Yuan 元 in____ （1900...2013（**FN037_w2_2**） Year 年 ____ （0...12 （**FN037_w2_3**） Month 月
   [IWER: Mark the year using four digits. Take down the month as its actual number. For example, write January as “1” not “01”, December as “12”. If do not remember month, fill ‘0’. 访员注意：用 4 位数表示年，按照实际的月份填写月。例：1 月写作“1”，而不是“01”，12 月写作“12”。如果记不住月份，请填入’0’。]
2. No 没有

FN038_w2 What is the earliest age at which you could leave this employer and start to receive pension benefits? 您最早可以在多大年纪时离开这家单位，并开始领取这项养老金？ ____45...120 Years old 岁

FN039_w2 By how much would your pension be reduced from full benefits if you left this job at (AGE IN FN038_w2)? 如果您 [preload FN038_w2] 岁时离开现在的工作，您可领取的养老金会比预期的全额养老金减少多少？By ____0.00...100.00 (**FN039_w2_1**) % or 或 ____ (**FN039_w2_2**) Yuan 元

FN039_w2_bracket [IWER: If R is unwilling to answer or does not remember, ask unfolding bracket questions here. 访员注意：如果受访者不愿回答或者忘记了，在此处分级展开提问]: 500 /1,000 /2,000 /3,500 /5,000 yuan 元

**PROCEDURE 程序**:
Skip to FN043_w2 跳至FN043_w2

FN040_w2 How much cash settlements did you receive? 您买断时拿了多少钱？ _______ Yuan 元

**PROCEDURE 程序**:
Skip to FN043_w2 跳至FN043_w2

FN041_w2 In what month and year did you start to receive pension benefits from supplement pension insurance of the firms? 您是从哪年哪月开始领企业补充养老保险金的？ ____ 1900...2013 (**FN041_w2_1**) year 年 __0...12 (**FN041_w2_2**) month 月 [F1：“企业补充养老保险”也叫企业年金，是指企业及其职工在依法参加基本养老保险的基础上，自愿建立的补充养老保险制度。] [IWER: Mark the year using four digits. Take down the month as its actual number. For example, write January as “1” not “01”, December as “12”. If do not remember month, fill ‘0’. 访员注意：用 4 位数表示年，按照实际的月份填写月。例：1 月写作“1”，而不是“01”，12 月写作“12”。如果记不住月份，请填入’0’。]

FN042_w2 What are your monthly benefits? 您每月领多少钱？ ____ Yuan per month 元/月

Part 3 商业养老保险

FN043_w2 Did you participate in commercial pension insurance, did someone buy commercial pension insurance for you, or did you get commercial pension insurance? 您是否参加了/别人给买了商业养老保险，或者正在领取商业养老保险金？

1. Yes, but I don’t receive it 参加了/别人帮忙买了，但是还未领取
2. Yes, I receive it 现在在领取商业养老保险金
3. No 没有参加或领取商业养老保险 → Skip to FN057_w2 跳至 FN057_w2

FN044_w2 Where did you participate in this insurance? 您在哪儿参加的这份保险？

1. The same as permanent address 和目前常住地一样
2. Another village/neighborhood in permanent address’s county/city/district 常住地所在县/市/区的其他村/社区 ____ (**FN044_w2_1**) village/neighborhood 村/社区
3. Other 其它: ____ (**FN044_w2_2**) province_city_county/city/district 省 _ 市 _ 县/市/区, ____ (**FN044_w2_3**) village/neighborhood 村/社区
4. Abroad 国外 [IWER: 访员注意：如果记不清详细的地址名称，请在相应输入框中填入“其它”。]

FN045_w2 Who paid for the commercial pension insurance? 谁给您买的商业养老保险？

1. Myself 我自己
2. My employer 我的雇主
3. My family or relative 我的家人或亲戚
4. Other person 其他人

F1:“商业养老保险”是指受访者直接向商业保险公司投保，定期缴纳保险费，从合同约定年龄开始持续、定期地领取养老金的人寿保险，能有效地满足客户的养老需要。

> **PROCEDURE 程序:**
> Skip FN046_w2-FN054_w2_bracket if FN043_w2 = 2. 如果FN043_w2 = 2，跳过FN046_w2-FN054_w2_bracket

FN046_w2 When did you start paying for the commercial pension? 您什么时候开始购买这商业养老保险的? ____ 1900...2013 (**FN046_w2_1**) year 年 __0...12 (**FN046_w2_2**) month 月

F1:“商业养老保险”是指受访者直接向商业保险公司投保，定期缴纳保险费，从合同约定年龄开始持续、定期地领取养老金的人寿保险，能有效地满足客户的养老需要。[IWER: Mark the year using four digits. Take down the month as its actual number. For example, write January as “1” not “01”, December as “12”. If do not remember month, fill ‘0’. 访员注意：用 4 位数表示年，按照实际的月份填写月。例：1 月写作“1”，而不是“01”，12 月写作“12”。如果记不住月份，请填入’0’。]

FN047_w2 How do you contribute to the commercial pension? 以什么样的形式交纳保险费?
1. Annual payment 按年
2. Lump sum amount 一次性缴费 → Skip to FN050_w2 跳至 FN050_w2

FN048_w2 You contribute ____ yuan/ year to the commercial insurance ? 您每年需要交纳多少保险费? ____ Yuan/ year 元/年

FN048_w2_bracket [IWER: If R is unwilling to answer or does not remember, ask unfolding bracket questions here. 访员注意：如果受访者不愿回答或者忘记了，在此处分级展开提问]: 500 /1,000 /2,000 /3,500 /5,000 yuan 元

FN049_w2 How many years do you need to pay? 您需要缴几年保险费? ____ Years 年
Soft Check: Prompt for verification if greater than a legal maximum

> **PROCEDURE 程序:**
> Skip FN050_w2-FN050_w2_bracket if FN047_w2 = 2. 如果FN047_w2 = 2，跳过FN050_w2-FN050_w2_bracket

FN050_w2 How much premium do you need to pay in total? 您一共需要交纳多少保险费? ____Yuan 元

FN050_w2_bracket [IWER: If R is unwilling to answer or does not remember, ask unfolding bracket questions here. 访员注意：如果受访者不愿回答或者忘记了，在此处分级展开提问]: 500 /1,000 /2,000 /3,500 /5,000 yuan 元

FN051_w2 How do you receive the pension? 保险费的领取方式?

1. Lump sum amount 一次性领取 → Skip FN052_w2-FN053_w2_bracket 请跳过FN052_w2-FN053_w2_bracket
2. Yearly 年领
3. Monthly 月领

PROCEDURE 程序:
If FN051_w2 = 2 ask FN052_w2 and FN052_w2_bracket

FN052_w2 How much do you expect to receive____ yuan/ year after your retirement? 您预期退休后每年能领取多少保险费? ____ Yuan/year 元/年
Soft Check: Prompt for verification if low or high, e.g. FN052_w2 < 1200 per year or FN052_w2 > 60000 per year

FN052_w2_bracket [IWER: If R is unwilling to answer or does not remember, ask unfolding bracket questions here. 访员注意: 如果受访者不愿回答或者忘记了, 在此处分级展开提问]: 500 /1,000 /2,000 /3,500 /5,000 yuan 元

PROCEDURE 程序:
If FN051_w2 = 3 ask FN053_w2 and FN053_w2_bracket

FN053_w2 How much do you expect to receive ____ yuan/month in the future? 您预期以后每月能领取多少养老金? ____ Yuan/ month 元/月
Soft Check: Prompt for verification if low or high, e.g. FN053_w2 < 100 per month or FN053_w2 >5000 per year

FN053_w2_bracket [IWER: If R is unwilling to answer or does not remember, ask unfolding bracket questions here. 访员注意: 如果受访者不愿回答或者忘记了, 在此处分级展开提问]: 500 /1,000 /2,000 /3,500 /5,000 yuan 元

FN054_w2 How much do you expect to receive ____ yuan in total? 您预期领取的养老金总额能达到多少? ____ Yuan 元

FN054_w2_bracket [IWER: If R is unwilling to answer or does not remember, ask unfolding bracket questions here. 访员注意: 如果受访者不愿回答或者忘记了, 在此处分级展开提问]: 500 /1,000 /2,000 /3,500 /5,000 yuan 元

PROCEDURE 程序:
Skip to FN057_w2. 跳至 FN057_w2

FN055_w2 In what month and year did you start to receive commercial pension benefits? 您是从哪年哪月开始领商业养老金的? ____ 1900...2013 (**FN055_w2_1**) year 年 __0...12 (**FN055_w2_2**) month 月
[IWER: Mark the year using four digits. Take down the month as its actual number. For example, write January as "1" not "01", December as "12". If do not remember month, fill '0'. 访员注意: 用 4 位数表示年, 按照实际的月份填写月。例: 1 月写作 "1", 而不是 "01", 12 月写作 "12"。如果记不住月份, 请填入' 0'。]

FN056_w2 What is your monthly benefit? 您每月领多少钱? ____ Yuan per month 元/月

FN056_w2_bracket [IWER: If R is unwilling to answer or does not remember, ask unfolding bracket questions here. 访员注意：如果受访者不愿回答或者忘记了，在此处分级展开提问]: 500 /1,000 /2,000 /3,500 /5,000 yuan 元

Part 4 人寿保险

FN056_w2_1 Did you participate in life insurance? 你是否购买过人寿保险？

1. Yes 是
2. No 否 → Skip to FN057_w2 跳至 FN057_w2

FFN056_w2_2 When did you start to participate in life insurance? 您什么时候购买人寿保险的？ ____ 1900...2013 (**FN056_w2_2_1**) year 年 __0...12 (**FN056_w2_2_2**) month 月

FN056_w2_3 What kind of life insurance did you participate in? 你购买的人寿保险是哪一种？

1. Term life insurance 定期寿险
2. Whole life insurance 终身寿险
3. Survivorship insurance 生存保险
4. Endowment insurance 生死两全保险
5. Other, please specify 其它（请填写具体名称 ____）(**FN056_w2_3_1**)

FN056_w2_4 Who paid for your life insurance? 谁给您的人寿保险缴费？

1. Myself 我自己
2. My children 我的孩子
3. Other family member or relative 其他家人或亲戚
4. Others 其他人

FN056_w2_5 How to pay the insurance premium? 保险费的缴纳方式是？

1. Monthly 月交
2. Quaterly 季交
3. Half a year 半年交
4. Annually 年交

FN056_w2_6 How much is the payment amount? 缴费金额是多少？

1. If pay it monthly 如果月交，____Yuan/Month 元/月 (**FN056_w2_6_1**)
2. If pay it quarterly 如果季交，____Yuan/Quarter 元/季 (**FN056_w2_6_2**)
3. If pay it every six months 如果半年交，____Yuan/Half a year 元/半年 (**FN056_w2_6_3**)
4. If pay it annually 如果年交，____Yuan/Year 元/年 (**FN056_w2_6_4**)

FN056_w2_7 Did you receive reimbursement from your life insurance? 你是否从人寿保险中领取过赔付金？

1. Yes 是
2. No 否 → Skip to FN056_w2_9 跳至 FN056_w2_9

FN056_w2_8 How much did you get? 领取过多少钱？ ____Yuan 元

FN056_w2_9 How much do you expect to receive? 你预计将来可能从人寿保险中领取多少钱？ ____ (**FN056_w2_9_1**) Yuan/Month 元/月 Or 或 ____ (**FN056_w2_9_2**) Yuan 元（Lump sum amount 一次结清）

FN057_w2 What kind of pension did you participate in or receive? (multiple choice) 您是否参加了/正在领取以下养老保险？（可多选）

1. Rural pension 农村养老保险（老农保）
2. Residents' pension 城乡居民养老保险
3. Urban residents' pension 城镇居民养老保险
4. None of above 以上都没有 → Skip to FN069_w2 跳至 FN069_w2

PROCEDURE 程序:
If FN057_w2 is multiple choiced, ask FN058_w2-FN068_w2_bracket for every answer
如果FN057_w2多选，则针对每个答案分别询问 FN058_w2-FN068_w2_bracket

FN058_w2 For every answers of FN057_w2, do you participate in or receive the pension? 对于 [下载FN057_w2的答案]，您现在是参加保险（正在缴费）、还是正在领取保险金？

1. Participate in 参加保险/正在缴费
2. Receive the pension 正在领取养老保险金

FN059_w2 Where did you participate in the pension? 您在哪儿参加的 [下载FN057_w2的答案]？

1. The same as permanent address 和目前常住地一样
2. Another village/neighborhood in permanent address's county/city/district 常住地所在县/市/区的其他村/社区 ____ (**FN059_w2_1**) village/neighborhood 村/社区
3. Other 其它: ____ (**FN059_w2_2**) province_city_county/city/district 省_市_县/市/区, ____ (**FN059_w2_3**) village/neighborhood 村/社区
4. Abroad 国外

[IWER: 访员注意：如果记不清详细的地址名称，请在相应输入框中填入"其它"。]

PROCEDURE 程序:
Skip FN060_w2-FN066_w2_bracket if FN058_w2 = 2. 如果FN058_w2 = 2，跳过FN060_w2-FN066_w2_bracket

FN060_w2 Have you ever contributed to your [preload FN057_w2]? 您参加 [下载FN057_w2的答案] 缴过费吗？
[CAPI: (1) 如果是家人或亲戚替自己缴费，也算缴费 (2) If FN057_w2 = 2, prompt: If your residents' pension was transferred from other pension programs, your contribution to these other programs also counts. 如果FN057_w2 = 2，提示：如果您的城乡居民养老保险是从其他保险转过来的，为其他养老保险缴的费用也算]

1. Yes 是，缴过
2. No 否，没有 → Skip FN061_w2 and FN062_w2 跳过 FN061_w2 and FN062_w2

FN061_w2 In what month and year did you start to pay for your [preload FN057_w2]? 您或其他人是从哪年哪月开始为 [下载FN057_w2 的答案] 缴费的？____ 1900...2013 (**FN061_w2_1**) year 年 __0...12 (**FN061_w2_2**) month 月

FN062_w2 Your annual contribution was ____ Yuan, annual subsidy from the collective was ____ yuan, annual subsidy from the government was ____ Yuan; or your lump sum contribution was ____ Yuan, lump sum subsidy from the collective was ____ Yuan, lump sum subsidy from the government was ____ Yuan. 您每年缴费 ____ (**FN062_w2_1**) 元，集体每年补贴 ____ (**FN062_w2_2**) 元，政府每年补贴 ____ (**FN062_w2_3**) 元。或者您总共缴费 ____ (**FN062_w2_4**) 元，集体总共补贴 ____ (**FN062_w2_5**) 元，政府总共补贴 ____ (**FN062_w2_6**) 元。

PROCEDURE 程序:
Ask FN063_w2 and FN064_w2 if the respondent is enrolled in residents' pension FN057_w2 = 2. 如果受访者参加了城乡居民养老保险 FN057_w2 = 2，请回答FN063_w2和FN064_w2

FN063_w2 Was your residents' pension transferred from other pension insurances like rural pension and basic pension of the firms? 您参加的城乡居民养老保险是从农村养老保险、企业基本养老保险或其他养老保险转过来的吗？

1. Yes 是的
2. No 不是 → Skip FN064_w2 跳过 FN064_w2

F1：（1）“农村养老保险”是指农村社会养老保险。1992 年民政部颁布了《县级农村社会养老保险基本方案》，确定以县为基本单位开展农村社会养老保险。（2）“企业基本养老保险”：是指受访者参加社会基本养老保险制度，达到退休年龄并符合领取条件后，领取到社会养老保险金。

FN064_w2 Which of the following pension program was your residents' pension transferred from? 您参加的城乡居民养老保险是从以下哪种养老保险转过来的？

1. Rural pension 农村养老保险
2. Basic pension insurance of the firms 企业基本养老保险
3. Other 其他 ____(**FN064_w2_1**)

F1：（1）“农村养老保险”是指农村社会养老保险。1992 年民政部颁布了《县级农村社会养老保险基本方案》，确定以县为基本单位开展农村社会养老保险。（2）“企业基本养老保险”：是指受访者参加社会基本养老保险制度，达到退休年龄并符合领取条件后，领取到社会养老保险金。

PROCEDURE 程序:
If FN058_w2 = 1 ask FN065_w2-FN066_w2_bracket.

FN065_w2 When do you expect to receive pension? 您预期在多大年龄享用该保险？At age 在 ____45...120 (**FN065_w2_1**) years old 岁 or in 或在 ____ 0.00...100.00 (**FN065_w2_2**) years 年之后

Soft Check: Prompt or Verify if FN065_w2_1 <50 or FN065_w2_2 >60

FN066_w2 About how much do you expect your benefits to be? (as an amount per month or year or a lump sum?) 您预期自己能领多少钱？____ (**FN066_w2_1**) Yuan per month 元/月 Or 或 ____ (**FN066_w2_2**) Yuan 元（Lump sum amount 一次结清）
[Soft Check: Prompt or Verify if These Benefits are Low or High, e.g., FN066_w2_1 <100 | FN066_w2_1 >5000 per month.]

PROCEDURE 程序:
If FN058_w2 = 2 ask FN067_w2-FN068_w2_bracket
Otherwise skip to FN069_w2. 跳至 FN069_w2

FN067_w2 In what month and year did you start to receive your [preload FN002]? 您是从哪年哪月开始领取 [下载 FN057_w2]? ____ 1900...2013 (**FN067_w2_1**) year 年 __0...12 (**FN067_w2_2**) month 月
[IWER: Mark the year using four digits. Take down the month as its actual number. For example, write January as "1" not "01", December as "12". If do not remember month, fill '0'. 访员注意：用 4 位数表示年，按照实际的月份填写月。例：1 月写作"1"，而不是"01"，12 月写作"12"。如果记不住月份，请填入'0'。]

FN068_w2 How much do you receive now? (as an amount per month?) 您现在每月领多少钱？____ Yuan per month 元/月

Part 6 New Rural Social Pension Insurance 新型农村社会养老保险

FN069_w2 Do you currently participate in the New Rural Social Pension Insurance program? 您现在是否参加了新型农村社会养老保险？
1. Yes 是的 → Skip to FN071_w2 请跳至 FN071_w2
2. No 否

FN070_w2 Why don't you participate in the New Rural Social Pension Insurance program? (Circle all that applies) 您没有参加新型农村社会养老保险的原因是？（可多选）
1. The New Rural Social Pension has not been introduced in my local area 本地还没有开展新型农村社会养老保险 (**FN070_w2_1**)
2. I lack money 没有钱 (**FN070_w2_2**)
3. I am not satisfied with the benefits because it isn't cost-effective. 我对待遇水平不满意，缴费参加该保险是不划算的。(**FN070_w2_3**)
4. The benefits are poor, and mean nothing to my life. 待遇水平低，对我的生活没有什么意义 (**FN070_w2_4**)
5. Application and payment arrangements make it inconvenient to participate 申请办理或缴费不方便 (**FN070_w2_5**)
6. The mechanism for making contributions is not justifiable. 缴费设计不合理 (**FN070_w2_6**)
7. I do not have a local Hukou. 我没有本地户口 (**FN070_w2_7**)
8. I am already covered by other social pension insurance. 我已经参加了其他社会养老保险项目，不能重复参加 (**FN070_w2_8**)

9. Other 其他 (**FN070_w2_9**)

PROCEDURE 程序:
Skip to FN080_w2 跳至 FN080_w2

FN071_w2 In what month and year were you first covered by New Rural Social Pension Insurance? 您是从什么时候加入新型农村社会养老保险（新农保）的？____ 2008...2013 (**FN071_w2_1**) year 年 __0...12 (**FN071_w2_2**) month 月
[IWER: Mark the year using four digits. Take down the month as its actual number. For example, write January as “1” not “01”, December as “12”. If do not remember month, fill ‘0’. 访员注意：用 4 位数表示年，按照实际的月份填写月。例：1 月写作 “1”，而不是 “01”，12 月写作 “12”。如果记不住月份，请填入’0’。]

FN072_w2 How do you contribute to New Rural Pension Insurance? 您是以什么方式缴费的？
1. Annual payment 按年 ____ (**FN072_w2_1**) Yuan/Year 元/年
2. Lump sum amount 一次性缴费 ____ (**FN072_w2_2**) Yuan 元，equals to annual payment 等同于缴费 ____ (**FN072_w2_3**) Yuan/Year 元/年 → Skip to FN074_w2 请跳至 FN074_w2
3. 参保时距离领取年龄不足 15 年，因此一次性补缴 ____ (**FN072_w2_4**) Yuan 元，再按年缴费 ____ (**FN072_w2_5**) Yuan/Year 元/年
4. I don’t need to pay myself. I am over 60 years old, and I am covered through my children’s participation in the New Rural Social Pension Insurance. 自己超过 60 岁不用缴费，但符合条件的子女已纳入新型农村社会养老保险 → Skip to FN075_w2 请跳至 FN075_w2

FN073_w2 How many years do you need to pay to receive benefits? 您需要缴几年保险费?（补缴的保险费也要折算进去）____ Years 年

FN074_w2 Who pays for your participation in New Rural Social Pension Insurance? 谁为您的新农保缴费的?
1. Myself 我自己
2. My children 我的孩子
3. Other family member or relative 其他家人或亲戚
4. Others 其他人

FN075_w2 Did you start to receive pension benefit? 您是否已经领取了新型农村社会养老保险（新农保）发放的养老金？
1. Yes 是
2. No 否 → Skip to FN078_w2 请跳至 FN078_w2

FN076_w2 In what month and year did you start to receive pension benefits? 您是从哪年哪月开始领取领取新型农村社会养老保险待遇的？____ 2008...2013 (**FN076_w2_1**) year 年 __0...12 (**FN076_w2_2**) month 月

[IWER: Mark the year using four digits. Take down the month as its actual number. For example, write January as "1" not "01", December as "12". If do not remember month, fill '0'. 访员注意：用 4 位数表示年，按照实际的月份填写月。例：1 月写作"1"，而不是"01"，12 月写作"12"。如果记不住月份，请填入'0'。]

FN077_w2 How much do you receive every month? 您每月能领取多少养老金？____ Yuan/month 元/月

FN077_w2_bracket [IWER: If R is unwilling to answer or does not remember, ask unfolding bracket questions here. 访员注意：如果受访者不愿回答或者忘记了，在此处分级展开提问]: 500 /1,000 /2,000 /3,500 /5,000 yuan 元

PROCEDURE 程序:
Skip to FN080_w2 跳至 FN080_w2

FN078_w2 When do you expect to receive pension benefit? 您预期在多大年龄领取新型农村社会养老保险的保险金？At age 在 ____ (**FN078_w2_1**) 45...120 岁 or in 或在 ____ (**FN078_w2_2**) years 年之后。

FN079_w2 About how much do you expect your benefits to be? 您预期自己能领多少钱？____ Yuan per month 元/月

FN079_w2_bracket [IWER: If R is unwilling to answer or does not remember, ask unfolding bracket questions here. 访员注意：如果受访者不愿回答或者忘记了，在此处分级展开提问]: 500 /1,000 /2,000 /3,500 /5,000 yuan 元

Part 7 征地养老保险（失地养老保险/被征地农民养老保险）

FN079_w2_1 Whether your land was acquired? 您家的土地是否被征用过？
1. Yes 是
2. No 否 → Skip to FN080_w2 请跳至FN080_w2

FN079_w2_2 When was your land acquired? 您家的土地是哪一年被征用的？____ 1900...2013 Year 年

FN079_w2_3 Did you participate in land expropriation pension insurance, or did you receive the pension? 您是否参加了/正在领取征地养老保险？
1. Yes, but I don't receive it 参加了保险，还未领取养老保险金
2. Yes, I receive it 正在领取征地养老保险金 → Skip to FN079_w2_10 请跳至FN079_w2_10
3. No 没有参加或领取征地养老保险 → Skip to FN080_w2 请跳至FN080_w2

FN079_w2_4 Do you need to pay the premium by yourself? 您的征地养老保险是否需要个人缴费？
1. Yes 是

2. No 否 → Skip to FN079_w2_7 请跳至 FN079_w2_7

FN079_w2_5 Who paid for this pension insurance? 谁给您的征地养老保险缴费?

1. Myself 我自己
2. My children 我的孩子
3. Other family member or relative 其他家人或亲戚
4. Others 其他人

FN079_w2_6 How much did you need to pay? 要缴纳多少钱? ____ (**FN079_w2_6_1**) Yuan per month 元/月
Or 或 ____ (**FN079_w2_6_2**) Yuan per year 元/年

FN079_w2_6_bracket [IWER: If R is unwilling to answer or does not remember, ask unfolding bracket questions here. 访员注意: 如果受访者不愿回答或者忘记了, 在此处分级展开提问]: 10 /50 /100 /500 /1,000 yuan 元/月

FN079_w2_7 How much did government and community subsidize? 您的征地养老保险, 政府和集体补助多少钱? Government paid 政府出资 ____ (**FN079_w2_7_1**) Yuan per month 元/月 Or 或 ____ (**FN079_w2_7_2**) Yuan per year 元/年 Community paid 集体出资 ____ (**FN079_w2_7_3**) Yuan per month 元/月 Or 或 ____ (**FN079_w2_7_4**) Yuan per year 元/年

FN079_w2_7_bracket1 [IWER: If R is unwilling to answer or does not remember, ask unfolding bracket questions here. 访员注意: 如果受访者不愿回答或者忘记了, 在此处分级展开提问]: 政府出资 10 /50 /100 /500 /1,000 yuan 元/月

FN079_w2_7_bracket2 [IWER: If R is unwilling to answer or does not remember, ask unfolding bracket questions here. 访员注意: 如果受访者不愿回答或者忘记了, 在此处分级展开提问]: 集体出资 10 /50 /100 /500 /1,000 yuan 元/月

FN079_w2_8 When do you expect to receive this pension? 您预期在什么时候享用该保险? At age 在 ____ 45...120 (**FN079_w2_8_1**) years old 岁 or in 或在 ____ 0.00...100.00 (**FN079_w2_8_2**) years 年之后

FN079_w2_9 About how much do you expect your benefits to be? (as an amount per month or year or a lump sum?) 您预期自己能领多少钱? ____ (**FN079_w2_9_1**) Yuan per month 元/月 Or 或 ____ (**FN079_w2_9_2**) Yuan 元 (Lump sum amount 一次结清)

FN079_w2_9_bracket [IWER: If R is unwilling to answer or does not remember, ask unfolding bracket questions here. 访员注意: 如果受访者不愿回答或者忘记了, 在此处分级展开提问]: 10 /50 /100 /500 /1,000 yuan 元/月

PROCEDURE 程序:
If FN079_w2_3 = 2 ask FN079_w2_10-FFN079_w2_11_bracket.
Otherwise skip to FN080_w2. 跳至 FN080_w2

FN079_w2_10 In what month and year did you start to receive this pension? 您是从哪年哪月开始领取征地养老保险金的? ____ 1990...2013 (**FN079_w2_10_1**) year 年 __0...12 (**FN079_w2_10_2**) month 月
[IWER: Mark the year using four digits. Take down the month as its actual number. For example, write January as “1” not “01”, December as “12”. If do not remember month, fill ‘0’. 访员注意: 用 4 位数表示年, 按照实际的月份填写月。例: 1 月写作 “1”, 而不是 “01”, 12 月写作 “12”。如果记不住月份, 请填入’ 0’。]

FN079_w2_11 How much do you receive now? (as an amount per month?) 您现在每月领多少钱? ____ Yuan per month 元/月

FN079_w2_11_bracket [IWER: If R is unwilling to answer or does not remember, ask unfolding bracket questions here. 访员注意: 如果受访者不愿回答或者忘记了, 在此处分级展开提问]: 10 /50 /100 /500 /1,000 yuan 元/月

Part 8 高龄老人补贴

FN080_w2 Did you receive old age pension allowance? 您是否在领取高龄老人补贴?

1. Yes 是
2. No 否 → Skip to FN083_w2 请跳至 FN083_w2

FN081_w2 In what month and year did you start to receive the pension subsidy for the oldest old? 您是从哪年哪月开始领取高龄老人养老补贴的? ____ 1990...2013 (**FN081_w2_1**) year 年 __0...12 (**FN081_w2_1**) month 月
F1: “高龄老人养老补助 (补贴)” 指 60 岁以上老人享有政策性的优惠, 如乘车免费、挂号费免费、参观等免费、部分社区活动免费, 如注射流感疫苗等, 百岁以上老人才享有月补助金。
[IWER: Mark the year using four digits. Take down the month as its actual number. For example, write January as “1” not “01”, December as “12”. If do not remember month, fill ‘0’. 访员注意: 用 4 位数表示年, 按照实际的月份填写月。例: 1 月写作 “1”, 而不是 “01”, 12 月写作 “12”。如果记不住月份, 请填入’ 0’。]

FN082_w2 How much do you receive now? 您现在每月领多少钱? ____ Yuan per month 元/月

FN082_w2_bracket [IWER: If R is unwilling to answer or does not remember, ask unfolding bracket questions here. 访员注意: 如果受访者不愿回答或者忘记了, 在此处分级展开提问]: 500 /1,000 /2,000 /3,500 /5,000 yuan 元

Part 9 其他养老保险

FN083_w2 You just told us you are receiving benefits from other pension program, what is the name of this pension program? 除了上述几种养老保险之外, 您是否还参加了其他养老保险项目/正在领取其他养老保险金?

1. Yes 是, 正在参加其他养老保险项目/正在缴费, 目前还未领取养老金

2. Yes 是，参加了其他养老保险项目，现在在领取养老金
3. No 否，没有参加其他养老保险项目 → Skip to FN097_w2 请跳至 FN097_w2

FN084_w2 What is the name of the program? 这个项目的名称是 ________

[IWER:. 访员注意：如果受访者回答正在参加/领取多份其他养老保险，请其选取自己认为最主要的一份回答。]

FN085_w2 where did you participate in the pension program? 您在哪儿参加的这份保险项目？

1. The same as permanent address 和目前常住地一样
2. Another village/neighborhood in permanent address' s county/city/district 常住地所在县/市/区的其他村/社区 ____ (**FN085_w2_1**) village/neighborhood 村/社区
3. Other 其它: ____ (**FN085_w2_2**) province_city_county/city/district 省_市_县/市/区, ____ (**FN085_w2_3**) village/neighborhood 村/社区
4. Abroad 国外

[IWER: 访员注意：如果记不清详细的地址名称，请在相应输入框中填入“其它”。]

**PROCEDURE 程序:**<br>Skip FN086_w2-FN094_w2_bracket if FN083_w2 = 2. 如果FN083_w2 = 2，跳过FN086_w2-FN094_w2_bracket

FN086_w2 When did you start to participate in this pension? 您什么时候开始参加这份养老保险的？ ____ 1990...2013(**FN086_w2_1**)year 年 __0...12 (**FN086_w2_2**)month 月

[IWER: Mark the year using four digits. Take down the month as its actual number. For example, write January as “1” not “01”, December as “12”. If do not remember month, fill ‘0’. 访员注意：用 4 位数表示年，按照实际的月份填写月。例：1 月写作“1”，而不是“01”,12 月写作“12”。如果记不住月份，请填入’0’。]

FN087_w2 Did you need to pay the premium? 您参加的这份养老保险需要缴费吗？

1. Yes 需要
2. No 不需要 → Skip to FN093_w2 请跳至 FN093_w2

FN088_w2 Who paid for the this pension insurance? 谁给您的这份养老保险缴费？

1. Myself 我自己
2. My employer 我的雇主
3. My family or relative 我的家人或亲戚
4. Other person 其他人

FN089_w2 How do you contribute to the commercial pension? 以什么样的形式交纳保险费?

1. Annual/Monthly payment 按年/按月
2. Lump sum amount 一次性缴费 → Skip to FN092_w2 请跳至 FN092_w2

FN090_w2 You contribute ____ Yuan/ year to the commercial insurance ? 按年算的话，您每年需要交纳多少保险费？ ____ Yuan/ year 元/年

FN090_w2_bracket [IWER: If R is unwilling to answer or does not remember, ask unfolding bracket questions here. 访员注意：如果受访者不愿回答或者忘记了，在此处分级展开提问]: 500 /1,000 /2,000 /3,500 /5,000 yuan 元

FN091_w2 How many years do you need to pay? 您需要缴几年保险费? ____ Years 年 [Soft Check: Prompt for verification if greater than a legal maximum]

**PROCEDURE 程序:**
Skip FN092_w2-FN092_w2_bracket if FN089_w2 = 1. 如果 FN089_w2 = 1，跳过 FN092_w2-FN092_w2_bracket

FN092_w2 How much premium do you need to pay in total? 您一共需要交纳多少保险费? ____Yuan 元

FN092_w2_bracket [IWER: If R is unwilling to answer or does not remember, ask unfolding bracket questions here. 访员注意：如果受访者不愿回答或者忘记了，在此处分级展开提问]: 500 /1,000 /2,000 /3,500 /5,000 yuan 元

FN093_w2 When do you expect to receive pension 您预期在多大年龄享用该保险? At age 在 ____ (**FN093_w2_1**) 45..120 岁 or in 或在 ____ (**FN093_w2_2**) years 年之后。

FN094_w2 About how much do you expect your benefits to be? (as an amount per month or year or a lump sum?) 您预期自己能领多少钱? ____ (**FN094_w2_1**) Yuan per month 元/月 Or 或 ____ (**FN094_w2_1**) Yuan 元（Lump sum amount 一次结清）

FN094_w2_bracket [IWER: If R is unwilling to answer or does not remember, ask unfolding bracket questions here. 访员注意：如果受访者不愿回答或者忘记了，在此处分级展开提问]: 500 /1,000 /2,000 /3,500 /5,000 yuan 元

**PROCEDURE 程序:**
If FN083_w2 = 2 ask FN095_w2 - FN096_w2_bracket . 如果 FN083_w2 = 2 询问FN095_w2 - FN096_w2_bracket
Otherwise skip to FN097_w2. 跳至 FN097_w2

FN095_w2 In what month and year did you start to receive this pension benefits? 您是从哪年哪月开始领取这份养老金（退休金）的? ____ 1990...2013 (**FN095_w2_1**) year 年 __0...12 (**FN095_w2_2**) month 月
[IWER: Mark the year using four digits. Take down the month as its actual number. For example, write January as “1” not “01”, December as “12”. If do not remember month, fill ‘0’. 访员注意：用 4 位数表示年，按照实际的月份填写月。例：1 月写作“1”，而不是“01”，12 月写作“12”。如果记不住月份，请填入’0’。]

FN096_w2 How much do you receive now? 您现在每月领多少钱? ____ Yuan per month 元/月

FN096_w2_bracket [IWER: If R is unwilling to answer or does not remember, ask unfolding bracket questions here. 访员注意：如果受访者不愿回答或者忘记了，在此处分级展开提问]: 500 /1,000 /2,000 /3,500 /5,000 yuan 元

Ask all R 所有人回答：

FN097_w2 Who do you think you can rely on financially for old-age support？如果您将来老了干不动工作了，您认为生活来源主要将是什么？

1. Children 子女 → Skip to FN098_w2 请跳至 FN098_w2
2. Savings 储蓄结束本部分
3. Pension or retirement salary 养老金或退休金结束本部分
4. Commercial pension insurance 商业养老保险结束本部分
5. Other 其他结束本部分

F1：“商业养老保险”是指受访者直接向商业保险公司投保，定期缴纳保险费，从合同约定年龄开始持续、定期地领取养老金的人寿保险，能有效地满足客户的养老需要。

FN098_w2 Which child(ren)? 是哪个/哪些子女？(choose all that apply 可多选) [加载子女列表，让受访者选择]

G&H INCOME, EXPENDITURES AND ASSETS 收入、支出与资产

PROCEDURE 程序:
For new R, please skip section G1. 新受访者请跳过 G1 部分。

G1 RELATIVE INCOME 相对收入

[INTRO: The following questions are on subjective feelings 下面是关于主观感受的一些问题:]

[Show Card 出示卡片 24]

G001_W2 Compared to the average living standard of your relatives, how would you rate your standard of living? 与您的亲戚们的平均生活水平相比，您觉得自己的生活水平是好很多、好一些、差不多、差一些，还是差很多？

1. Much better 好得多
2. a little better 好一些
3. about the same 差不多
4. a little worse 差一些
5. much worse 差很多
6. I do not know 不知道

G002_W2 Compared to the average living standard of your schoolmates who are at the same level of education with you, how would you rate your standard of living? 与和您教育程度一样的同学们的平均生活水平相比，您觉得自己的生活水平是好很多、好一些、差不多、差一些，还是差很多？

1. Much better 好得多
2. a little better 好一些
3. about the same 差不多
4. a little worse 差一些
5. much worse 差很多
6. I do not know 不知道
7. Not applicable 不适用

G003_W2 Compared to the average living standard of your colleagues, how would you rate your standard of living? 与您的同事们的平均生活水平相比，您觉得自己的生活水平是好很多、好一些、差不多、差一些，还是差很多？

1. Much better 好得多
2. a little better 好一些
3. about the same 差不多
4. a little worse 差一些
5. much worse 差很多

6. I do not know 不知道
7. Not applicable 不适用

G004_W2 Compared to the average living standard of your neighbors or others in your village or neighborhood, how would you rate your standard of living? 与您的邻居/村里人的平均生活水平相比，您觉得自己的生活水平得是好很多、好一些、差不多、差一些，还是差很多？
1. Much better 好得多
2. a little better 好一些
3. about the same 差不多
4. a little worse 差一些
5. much worse 差很多
6. I do not know 不知道

G005_W2 Compared to the average living standard of people in your city or county, how would you rate your standard of living? 与本县/市/区人们的平均生活水平相比，您觉得自己的生活水平是好很多、好一些、差不多、差一些，还是差很多？
1. Much better 好得多
2. a little better 好一些
3. about the same 差不多
4. a little worse 差一些
5. much worse 差很多
6. I do not know 不知道

G2 HOUSEHOLD INCOME AND EXPENDITURES 家户收入与支出

[IWER: Part 1_1 is asked of the main respondent and spouse respectively. Other parts in this section is asked of the family respondent. Do not allow a proxy respondent to answer the entire section. Part 1_1 由主要受访者及其配偶分别回答，其余部分由家庭受访者回答。本部分不允许完全请别人代答。]

PART 1 Household Wage Income and Individual-based transfers 家户工资收入和个人获得的转移收入

Part 1_1: Main Respondent and Spouse's Wage Income and Individual-based transfers 主要受访者及其配偶的工资收入和个人获得的转移收入

[IWER: Please conduct Part 1_1 when the main respondent and spouse are at home. Don't allow a proxy to complete the part. 访员注意：当主要受访者及其配偶在场时，提问 Part 1_1 问卷。这部分不允许请人完全代填。]

GA001 Did you receive any wage and bonus income in the past year? 过去一年，您有没有领工资（包括奖金、各种补贴，不包括退休工资）？
1. Yes 有

2. No 没有 → Skip to GA003 跳至 GA003

GA002 How much did you receive last year? 一共领了多少钱? _____ (**GA002_1**) yuan 元或每个月领多少钱? _____ (**GA002_2**) yuan/month 元
Wage income: yuan [soft check >240,000] or yuan/month [soft check>18,000]

GA002_bracket [IWER: If R is unwilling to answer or does not remember, ask unfolding bracket questions here. 访员注意：如果受访者不愿回答或者忘记了，在此处分级展开提问]
10,000 /30,000 /50,000 /100,000 /200,000 yuan 元

GA002_W2_1 Does the above mentioned wage exclude any insurance, income tax, public housing funds and other fees? 上面提到的工资有没有扣除各类保险、所得税、住房公积金或其他杂费?

1. Yes 有
2. No 没有

GA002_W2_2 What is the total amount of your insurance, income tax, public housing funds and other fees? 您被扣除的/上交的个人所得税、各类保险、住房公积金或其他杂费一共是多少元? _______ (**GA002_w2_2a**) Yuan/year 元/年, or _____ (**GA002_w2_2b**) Yuan/month 元/月, or about 或相当于工资的 ___ (**GA002_w2_2c**) % of wage。
[IWER: If R is unwilling to answer or does not remember, ask unfolding bracket questions here. 访员注意：如果受访者不愿回答或者忘记了，在此处分级展开提问:] 个人所得税、各类保险、住房公积金或其他杂费一共是多少元? 300 /500 /1,000 /2,000 /3,000 yuan/month 元/月。
Among it, 其中:

1. Income tax 个人所得税是 _______ (**GA002_w2_2_1a**) Yuan/Yea 元/年，或 _____ (**GA002_w2_2_1b**) Yuan/Month 元/月, or about 或相当于工资的 ___ (**GA002_w2_2_1c**) % of wage.
[IWER: If R is unwilling to answer or does not remember, ask unfolding bracket questions here. 访员注意：如果受访者不愿回答或者忘记了，在此处分级展开提问: 个人所得税 0 /50 /100 /500 /1,000 yuan 元/月
2. Various insurance (pension insurance, health insurance, unemployment insurance,worker’s injury insurance, maternity insurance) 各类保险（包括养老保险、医疗保险、失业保险、工伤保险、生育保险等）是 _______ (**GA002_w2_2_2a**) Yuan/Year 元/年，或 _____ (**GA002_w2_2_2b**) Yuan/Month 元/月, or about 或相当于工资的 ___ (**GA002_w2_2_2c**) % of wage.
[IWER: If R is unwilling to answer or does not remember, ask unfolding bracket questions here. 访员注意：如果受访者不愿回答或者忘记了，在此处分级展开提问:]
各类保险（包括养老保险、医疗保险、失业保险、工伤保险、生育保险等）0 /50 /100 /500 /1,000 yuan/month 元/月
3. Public housing fund 住房公积金是 _______ (**GA002_w2_2_3a**) Yuan/Year 元/年，或 _____ (**GA002_w2_2_3b**) Yuan/Month 元/月, or about 或相当于工资的 ___ (**GA002_w2_2_3c**)

% of wage。
[IWER: If R is unwilling to answer or does not remember, ask unfolding bracket questions here. 访员注意：如果受访者不愿回答或者忘记了，在此处分级展开提问:] 住房公积金 0 /50 /100 /500 /1,000 yuan/month 元/月

[Show Card 出示卡片 25]

GA003 Did you receive any of the following types of individual income in the past year? (check all that apply) 过去一年，您有没有领到下列收入？(可多选)

1. Pensions (including wages from government institutions and firms, supplemental pension of the firms,and income from such programs as rural pension insurance, Urban residents' pension and commercial pension insurance, new rural social pension insurance and pension subsidy for the oldest old) 退休金或养老金（包括政府机关和事业单位退休金，企业职工基本养老保险，企业补充养老保险，农村、城乡、城镇居民养老保险，商业养老保险，人寿保险，征地养老保险，高龄老人养老补助等）
2. unemployment compensation 失业补助
3. pension subsidy 无保障老人生活补贴
4. Workers' compensation from Industrial Accident Compensation Insurance includes wage-replacement benefits, disability benefits, and survivors' benefits 工伤保险金包括误工补贴、伤残补助、丧葬费等
5. elderly family planning subsidies 独生子女老年补助
6. medical aid 医疗救助
7. other government subsidies 政府给个人的其他补助
8. social assistance 社会捐助
9. other income sources 其他收入
10. None of the above 以上均没有 → Skip GA004 跳过 GA004

GA004 How much did you receive last year? 过去一年一共领了多少钱？____ (**GA004_1**) 元 Yuan 或每个月领多少钱？____ (**GA004_2**) 元/月 Yuan/Month

PROCEDURE 程序:

If GA003 = 1 and GA004 = DK, ask GA004_bracket. 如果领取了退休金或养老金，但是没回答多少钱，提问 GA004_bracket

GA004_bracket [IWER: If R is unwilling to answer or does not remember, ask unfolding bracket questions here. 访员注意：如果受访者不愿回答或者忘记了，在此处分级展开提问:] 加起来一共值多少钱？10,000 /30,000 /50,000 /100,000 /200,000 yuan 元

Part 1_2: Other Household Member's Wage Income and Individual-based transfers 其他家户成员的工资收入和个人获得的转移收入

[IWER reminder: make sure others are not present. 访员注意：请确保没有其他人在场。]

[Intro: We'd like to ask you some questions about the income and assets of OTHER members of your household. 下面我们想问问您家其他家户成员的收入情况。我们将会对您的回答严格保密，并且仅用于学术研究。]

PROCEDURE 程序:
The names of other household members are preloaded from the cover screen information. For each member (excluding main respondent and spouse)ask GA005 to GA008_bracket. 其他家户成员名单要从过滤问卷获得。对于每一个家户成员（不包括主要受访者及其配偶），分别询问 GA005 至 GA008_bracket。

GA005 Did [preload household member name] receive any wage and bonus income in the past year? 过去一年，[preload household member name] 有没有领工资（包括奖金，不包括退休工资）？

1. Yes 有
2. No 没有 → Skip to GA007 跳至 GA003

GA006 After tax and various social insurances, how much did he/she receive in the past year year? 扣除税和缴纳的社会保险，过去一年他/她一共领了多少钱？ ______ (**GA006_1**) yuan 元 or 或每个月领多少钱？ _____ (**GA006_2**) yuan/month 元
Wage income: yuan [soft check >200,000] or yuan/month [soft check>15,000]

GA006_bracket [IWER: If R is unwilling to answer or does not remember, ask unfolding bracket questions here. 访员注意：如果受访者不愿回答或者忘记了，在此处分级展开提问]
10,000 /30,000 /50,000 /100,000 /200,000 yuan 元

GA006_w2 过去一年，[preload household member name] 缴纳的税和社会保险一共有多少钱？ ______ (**GA006_w2_1**) yuan/month 元/年 or 或每个月缴多少钱？ _____ (**GA006_w2_2**) yuan 元/月。

GA006_w2_bracket [IWER: If R is unwilling to answer or does not remember, ask unfolding bracket questions here. 访员注意：如果受访者不愿回答或者忘记了，在此处分级展开提问]
300 /500 /1,000 /2,000 /3,000 yuan 元/月

[Show Card 出示卡片 25]

GA007 Did [preload household member name] receive any of the following types of individual income in the past year? (check all that apply) 过去一年，[preload household member name] 有没有领到下列收入？（可多选）

1. Pensions (including wages from government institutions and firms, supplemental pension of the firms,and income from such programs as rural pension insurance, Urban residents' pension and commercial pension insurance, new rural social pension insurance and pension subsidy for the oldest old) 退休金或养老金（包括政府机关和事业单位退休金，企业职工基本养老保险，企业补充养老保险，农村、城乡、城镇居民养老保险，商业养老保险，人寿保险，征地养老保险，高龄老人养老补助等）

2. unemployment compensation 失业补助
3. pension subsidy 无保障老人生活补贴
4. Workers' compensation from Industrial Accident Compensation Insurance includes wage-replacement benefits, disability benefits, and survivors' benefits 工伤保险金包括误工补贴、伤残补助、丧葬费等
5. elderly family planning subsidies 独生子女老年补助
6. medical aid 医疗救助
7. other government subsidies 政府发放给个人的其他补助
8. social assistance 社会捐助
9. other income sources 其他收入
10. None of the above 以上均没有 → Skip to GA008 跳至 GA008

[F1:"医疗救助"在部分区县试点，救助标准是由各区县根据自身经济情况制定，导致救助标准有高有低。主要三种，一是提供社会医疗救助金，给救助对象以经济补偿；二是给医疗机构一定的经济补贴，使后者直接减免救助对象的部分医疗费；三是由社会医疗救助机构举办专门医疗机构，免费为救助对象提供医疗服务。]

GA008 How much did he/she receive last year? 过去一年一共领了多少钱？____ (**GA008_1**) yuan 元 [soft check >10000] or 或每个月领多少钱？____ (**GA008_2**) yuan/month 元/月 [soft check >3000]

PROCEDURE 程序:
If GA007 = 1 and GA008 = DK, ask GA008_bracket. 如果领取了退休金或养老金，但是没回答多少钱，提问 GA008_bracket

GA008_bracket [IWER: If R is unwilling to answer or does not remember, ask unfolding bracket questions here. 访员注意：如果受访者不愿回答或者忘记了，在此处分级展开提问：] 10,000 /30,000 /50,000 /100,000 /200,000 yuan 元

PROCEDURE 程序:
Skip to next person. 请询问下一个人。

PART 2 HOUSEHOLD AGRICULTURAL INCOME AND EXPENDITURE 家户农业收入与支出

[Intro: Next we will ask some questions about your household agricultural income and expenditure. 下面，我们将问一些有关您家农业收入与支出的问题。]

GB001 Did your household engage in agricultural work (including cropping, forestry, livestock, and fish) last year? 过去一年，除了您和您的配偶，您家其他家庭成员有没有从事种地、管理果树、采集农林产品、养鱼、打鱼、养牲畜等农业活动，或者去市场销售自家生产的农产品？

1. Yes 有

2. No 没有 → Skip to GC001 跳至 GC001

GB002 [IWER: The names of other household members not including respondent and spouse are preloaded from the cover screen information: 访员注意：其他家户成员不包括主要受访者及其配偶，其他家户成员的名单从过滤问卷获得] [preload other household member name] Who engaged in agricultural work in the past year? 过去一年，从事这些农业活动的家户成员有哪些？(可多选)

Crops and forestry products 农林产品

GB003 Did your household engage in cropping or forestry last year? 您家过去一年是否从事了种植业或林业（包括花木、蔬菜和各类农作物的种植）?

1. Yes 是
2. No 否 → Skip to GB007 跳至 GB007

GB004 When was the most recent harvest? 最近的一次收成是在什么时候？____2009...2013 (**GB004_1**) year 年 ____0...12 (**GB004_2**) month 月
[IWER: Mark the year using four digits. Take down the month as its actual number. For example, write January as "1" not "01", December as "12". If do not remember month, fill '0'. 访员注意：用 4 位数表示年，按照实际的月份填写月。例：1 月写作“1”，而不是“01”，12 月写作“12”。如果记不住月份，请填入’0’。]

GB005 What is the total value of all crops and forestry products produced in the past year? 过去一年，您家生产的农产品和林产品加起来一共值多少钱？____ (**GB005_1**) Yuan 元 [soft check: 75000 yuan] Among it, what is the value of the crops and forestry products that is home consumed? 其中，自家消费的占多少？____ (**GB005_2**)Yuan 元或 __ (**GB005_3**) %

GB005_bracket [IWER: If R is unwilling to answer or does not remember, ask unfolding bracket questions here. 访员注意：如果受访者不愿回答或者忘记了，在此处分级展开提问:] 加起来一共值多少钱？1,000 /3,000 /5,000 /7,000 /10,000 yuan 元

GB005_w2_bracket [IWER: If R is unwilling to answer or does not remember, ask unfolding bracket questions here. 访员注意：如果受访者不愿回答或者忘记了，在此处分级展开提问:] 自家消费的占多少？1,000 /3,000 /5,000 /7,000 /10,000 yuan 元

GB006 What was the total cost of producing crops (including vegetables and Chinese herbs) and forestry products in the past year? (including Seeds (including home-used seeds), Fertilizer, Organic fertilizer, Pesticide, Plastic sheets, Hiring labor (including with machine or animals), Land rents, Rents (excluding land rents), Irrigation, Fuel, Transportation, Processing, Marketing (including packaging, management fee)) 过去一年，您家为了农业生产（包括种植粮食作物、蔬菜和中药材等）和林业生产（包括种植蘑菇、木耳等林下作物、种植茶叶），总共投入了多少钱？[包括种子（含自家留种的价值）、化肥、农家肥、农药、塑料薄膜、雇工费（包括其使用的机器和役畜）、土地租金、除地租以外的其他租金（例如：租用收割机）、灌溉费、燃料、运输费、加工费、市场费用（包括包装费，管理费等）] ____ Yuan 元 [soft check: 50,000 yuan]

GB006_bracket [IWER: If R is unwilling to answer or does not remember, ask unfolding bracket questions here. 访员注意：如果受访者不愿回答或者忘记了，在此处分级展开提问:] 3,00 /6,00 /1,000 /2000 /5,000 yuan 元

Livestock and fisheries 牲畜和水产品

GB007 Did your household grow any livestock or aquatic life last year? 您家过去一年养过牲畜（家禽、家畜如鸡、鸭、牛、猪、羊等都算）或者水产品吗？

1. Yes 是
2. No 否 → Skip to GC001 跳至 GC001

GB008 What is the current value of all livestock（including chicken, duck, cattle, pig, sheep, etc.）and aquatic life? 现在，您家所有的家禽、家畜（包括鸡、鸭、牛、猪、羊等），水产品加起来一共值多少钱？ ____ Yuan 元 [soft check: 100,000 yuan]

GB008_bracket [IWER: If R is unwilling to answer or does not remember, ask unfolding bracket questions here. 访员注意：如果受访者不愿回答或者忘记了，在此处分级展开提问:] 5,00 /1,500 /3,000 /4,500 /9,000 yuan 元

GB009 What was the value of all livestock and aquatic life at this time last year? 去年这个时候，您家拥有的牲畜及水产品一共值多少钱？ ____Yuan 元 [soft check: 100,000 yuan]

GB009_bracket [IWER: If R is unwilling to answer or does not remember, ask unfolding bracket questions here. 访员注意：如果受访者不愿回答或者忘记了，在此处分级展开提问:] 5,00 /1,500 /2,500 /4,000 /8,000 yuan 元

GB010 How much did you spend purchasing new livestock and aquatic life in the past year? 过去一年，你们家买牲畜及水产品一共花了多少钱？ ____ Yuan 元 [soft check: 50,000]

GB011 What was the value of all livestock and aquatic life that were sold or consumed in the past year? 过去一年，你们家卖出去的和自家消费的牲畜及水产品加起来一共值多少钱?____ (**GB011_1**) Yuan 元 [soft check: 100,000 yuan] Among it, what is the amount or percent consumed at your home? 其中，自家消费的占多少？ ____ (**GB011_2**) Yuan 元或 ___ (**GB011_3**) %

GB011_bracket [IWER: If R is unwilling to answer or does not remember, ask unfolding bracket questions here. 访员注意：如果受访者不愿回答或者忘记了，在此处分级展开提问:] 加起来一共值多少钱？ 2,00 /9,00 /1,500 /2,500 /5,000 yuan 元

GB011_w2_bracket [IWER: If R is unwilling to answer or does not remember, ask unfolding bracket questions here. 访员注意：如果受访者不愿回答或者忘记了，在此处分级展开提问:] 加起来一共值多少钱？ 2,00 /9,00 /1,500 /2,500 /5,000 yuan 元

GB012 What was the value of all livestock products produced (including the self consumption value) in the past year, including milk, wool (including cashmere, sheep or goat skin), and eggs? 过去一年，你们家养的牲畜生产出来的副产品（比如鸡蛋、牛奶、羊毛、羊绒、羊皮），加起来一共值多少钱（包括所有卖出去的和自家消费的）? ____ **(GB012_1)** Yuan 元 [soft check: 50,000 yuan] Among it, what is the amount or percent consumed at your home? 其中，自家消费的占多少? ____ **(GB012_2)** Yuan 元或 ___ **(GB012_3)** % [soft check: 50,000 yuan]

GB012_bracket [IWER: If R is unwilling to answer or does not remember, ask unfolding bracket questions here. 访员注意：如果受访者不愿回答或者忘记了，在此处分级展开提问:] 一共值多少钱（包括所有卖出去的和自家消费的）? 100 /200 /300 /500 /1000 yuan 元

GB012_bracket_2 [IWER: If R is unwilling to answer or does not remember, ask unfolding bracket questions here. 访员注意：如果受访者不愿回答或者忘记了，在此处分级展开提问:] 其中，自家消费的占多少? 100 /200 /300 /500 /1000 yuan 元

GB013 What was the cost of producing livestock and aquatic life in the past year, including the value of all feed, medicine, pasture fees, animal pens, wages, etc. 过去一年，养这些牲畜及水产品一共花了多少钱，包括喂养费、医药费、放牧费、畜舍栅栏费、雇工费等? ____ Yuan 元

[soft check: 50,000 yuan] [soft check: "reported raising livestock but no evidence of such activity" if GB008 = 0 and GB009 = 0 如果受访者饲养过牲畜或水产品，但 GB008 = 0 且 GB009 = 0，则进行检查]

PART 3 Self-employed Activities 个体经营或开办私营企业

GC001 Did your household members engage in any self-employed activities last year? 过去一年，您家是否有家户成员从事某些个体经营或开办私营企业?

1. Yes 是
2. No 否 → Skip to GD001 跳至 GD001

GC002 How many types of activities did your household members participate in the past year? ____ activities. 过去一年，您家家户成员从事几项个体经营活动或开办几家私营企业?

PROCEDURE 程序:
For each each self-employed activity in GC002, ask GC003 - GC005. 针对每一项个体经营活动或私营企业，询问 GC003 - GC005

GC003 Who engaged in this self-employment businesse in the past year? 过去一年，从事这项个体经营或私营企业的家户成员有哪些?

[IWER: All the names of household members are preloaded from the cover screen information 访员注意：所有家户成员名单（包括主要受访者及其配偶）要从过滤问卷获得] (可多选)

[Show Card 出示卡片 26]

GC004 Which types of activities? 这项活动属于什么行业？

1. Services (cooking, sewing, private clinic etc.) 服务业（烹饪、缝纫、私人诊所等）(**GC004_1**)
2. Transportation 交通运输 (**GC004_2**)
3. Construction 建筑业 (**GC004_3**)
4. Mining 采掘 (**GC004_4**)
5. Processing production 加工生产业 (**GC004_5**)
6. Business 商业 (**GC004_6**)
7. Others 其他 (**GC004_7**)

GC005 Not including fixed capital costs, what is your best estimate of the net income earned from this activity by your household members last year? [If the activity was conducted jointly with non-household members, report only the net income earned by household members. Remember to consider the following types of costs: energy, housing or equipment rental, raw materials, transportation, marketing, wages, taxes or fees.] 不包括固定资本成本，您能否精确估计一下您家过去一年从这项活动净赚多少钱？如果此项经营活动有非家户成员参与，只计算家户成员的净收入。别忘了考虑下列各种成本：能源、住房和设备租用费、原材料、交通费、营销、工资、税收和杂费。____ Yuan 元
[soft check: 500,000 yuan]

GC005_bracket [IWER: If R is unwilling to answer or does not remember, ask unfolding bracket questions here. 访员注意：如果受访者不愿回答或者忘记了，在此处分级展开提问:]
5,000 /10,000 /50,000 /100,000 /200,000 yuan 元

PART 4 HOUSEHOLD PUBLIC TRANSFER INCOME 家户政府转移支付收入

[We ask the public transfers received by the households (with household as the unit). Public transfers have characteristic of welfare, such as Wubaohu Subsidy and Tekunhu Subsidy gived by government. 这部分询问家户从政府所得的各种转移支付收入。政府转移支付具有福利支出的性质，如政府给五保户和特困户的补助金等。这里询问的是以家户为单位获得的转移支付。]

GD001 How much Dibao assistance did your household receive last year? (if not applicable, fill in 0 yuan). 您家过去一年得到低保了吗？得到多少？（如果没有，请填 0）____ Yuan 元

[Show Card 出示卡片 27]

GD002 Did your household receive any of the following government subsidies in the past year? (check all that apply) 您家过去一年有没有收到下列政府补助？(可多选)
[soft checks for each category: 20,000 yuan 对每项进行检查：20,000 元。]

1. Reforestation 退耕还林: how much? 有多少？ ____ (**GD002_1**) Yuan 元
2. Agricultural subsidies 农业补助: how much? 有多少？ ____ (**GD002_2**) Yuan 元

3. Wubaohu (targets low-income, blind, disabled, aged persons, and young persons that have no means to support themselves. 五保户补助金（用来补助那些无法自力更生的低收入者、盲人、身体残疾者、老人和小孩: how much? 有多少？ ____ (**GD002_3**) Yuan 元
4. Tekunhu 特困户补助: how much? 有多少？ ____ (**GD002_4**) Yuan 元
5. Work injury subsidies to the immediate family members 工伤人员供养直系亲属抚恤金 how much? 有多少？ ____ (**GD002_5**) Yuan 元
6. Emergency or disaster relief (jiujikuan, jiuzaikuan) last year? 突发事件或重大灾难之后的补助（即救济金、赈灾款）(包括实物形式）：how much? 加上实物救助的价值一共有多少？ ____ (**GD002_6**) Yuan 元
7. Other 其他补助: how much? 有多少？ ____ (**GD002_7**) Yuan 元
8. None 没有收到任何政府补助 → Skip to GD003 跳至 GD003

[Show Card 出示卡片 28]

GD003 Did your household receive any income from the following sources in the past year? (check all that apply) 您家过去一年有没有收到下列哪种捐助或补偿？(可多选)

1. Donations from the society (including cash, and items like food, clothing, etc.) 社会捐助（包括现金，食品、衣服等）：how much? 加上实物的价值一共有多少？ ____ (**GD003_1**) Yuan 元 [soft check: 20,000 yuan]
2. Compensation for land seizure last? 征地补偿金: how much? 有多少？ ____ (**GD003_2**) Yuan 元 [soft check: 100,000 yuan]
3. Compensation to pulling down your house or apartment last year? 住房拆迁补偿: how much? 有多少？ ____(**GD003_3**) Yuan 元 [soft check: 100,000 yuan]
4. None 没有收到任何捐助或补偿

PART 5 HOUSEHOLD LIVING EXPENDITURE 家户生活支出

GE001 We wish to know your family food expenditure for the last week. Are you the primary person who purchases food for the household? 我们想知道您家最近一周的食品支出。您负责为家里购买食品吗？

1. Yes 是 → skip to GE004 跳至 GE004
2. No 否

GE002 Who is the primary person purchasing food for the household? 谁负责为您家购买食品？

[CAPI: Preload all the HHmember list 列出所有家户成员的名单]

[IWER: If possible, the primary person who purchases food for the household should answer the questions about expenditures FE003-FE009] [访员注意：如果可能的话，由负责为该家庭购买食品的人来回答生活支出问题]

GE004 In the past week, how many people usually ate meals together in your household (not including guests)? 最近一周，您家里一般有几口人吃饭（不包括客人)？ __ Persons 人 [soft check: 10]

GE005 Last week how many meals did you provide to guests? 最近一周，您家的客人在您家吃了几顿饭（按人次计算）? ____ meals 人次? [soft check: 100]

[Intro: The next questions are about your household living expenditure, including your household members '(preloaded names of household members) living expenditure. If one attends school/work outside and comes home almost every week, GE006 –GE008 includes his/her expenditureon food and meals outside. If one attends school/work outside but not come home every week, GE006 –GE008 excludes his/her expenditureon food and meals outside.] [引语：下面问题是关于您家的生活支出的。这些支出包括所有家户成员的生活支出。如果有人在外上学/上班，但周末回家，他们在外上学/上班发生的开支算在内；如果有人在外打工等，周末不回家，他们在外的生活开支不算在内。]

GE006 In the past week, how much did your household spend on food (excluding eating out expenditure, alcohol, cigarettes, cigars and tobacco expenditure)? 最近一周，您家花了多少钱购买食品，不包括外出就餐、购买香烟、酒水等? ____ Yuan 元 [soft check: 6000 yuan]

GE006_W2 Does your household produce agricultural products yourself (including plants, meat, eggs, aquatic lives, oil, vegetables and fruits, cigaretts and wine, drinks and milk products, produced food, seasonings, etc.)? 您家自己生产农产品吗（包括粮食作物、肉类、蛋类、水产品、油、蔬菜水果、烟酒、饮料及乳制品、加工食品、调料等）?

1. Yes 生产
2. No 不生产 → skip to GE007 跳至 GE007

GE006_W2_1 In the past week, what was the market value of the food that members of the household consumed that you grew yourselves? 最近一周，您家里人消费的自家生产的农产品在市场上卖的话值多少钱? ____ Yuan 元

GE007 Among it, how much did your household spend on eating out? 最近一周，您家花了多少钱外出就餐等? ____ Yuan 元 [soft check: 3000 yuan]

GE008 Among it, how much did your household spend on alcohol, Cigarettes, cigars and tobacco? 最近一周，您家花了多少钱购买香烟、酒水等? ____ Yuan 元 [soft check: 3000 yuan]

[Show Card 出示卡片 29]

GE009 Please tell me the expenditure last month for your household for the following items. 下面我们想了解您家过去一个月在以下各项消费中的支出。
[soft check for each category: 5000 yuan] [IWER: fill in 0 if no corresponding expenditure; fill in -9999 if the respondent cannot recall the expenditure. 访员注意：没有相应项支出用 0 元表示，记不清则请受访者估计一个数字，如果实在无法估计，该支出用 -9999 表示]

1. Communication fees (including post, internet usage, telephone and cell phone usage) 邮电、通讯支出（包括电话、手机、上网、邮寄等） ____ **(GE009_1)** Yuan 元
2. Utilities: Water and electricity 水费、电费 ____ **(GE009_2)** Yuan 元
3. Fuels (including gas, coal, etc.) 燃料费（包括煤炭、煤制品、柴草、木炭、天然气、液化气等） ____ **(GE009_3)** Yuan 元
4. Fees for Matron, housekeepers and servants 保姆、小时工、佣人等的支出 __ **(GE009_4)** Yuan 元
5. Local Transportation 在当地的交通费 ____ **(GE009_5)** Yuan 元
6. Household items and personal toiletries that are used daily plus beauty treatments (e.g., detergent, soap, toothpaste, toothbrush, cosmetics, beauty salon, etc.) 日用品（包括洗漱用品、家居用品、厨卫用品、装饰用品等） ____ **(GE009_6)** Yuan 元
7. Entertainment (including fees to buy books, newspapers, VCCs, DVDs, going to cinema and bars) 文化娱乐支出（包括书报杂志、光盘、影剧票、歌舞厅和网吧等） ____ **(GE009_7)** Yuan 元

[Show Card 出示卡片 30]

GE010 In the last year how much did your household spend on the following items? 下面我们想了解您家过去一年在以下各项消费中的支出。

[IWER: fill in 0 if no corresponding expenditure; fill in -9999 if the respondent cannot recall the expenditure. 访员注意：没有相应项支出用 0 元表示，记不清则请受访者估计一个数字，如果实在无法估计，该支出用 -9999 表示。] [soft check: >=100,000 yuan]

1. Clothing and bedding 衣着消费 ____ **(GE010_1)** Yuan 元
2. Long distance traveling expenses 家庭的旅游支出 ____ **(GE010_2)** Yuan 元
3. Heating(centrally heated) 家庭的取暖费支出 (指集中供暖)____ **(GE010_3)** Yuan 元
4. Furniture, consumption of durable goods and electronics, includes refrigerator, washing machine, TV, computers and expensive instruments like pianio. 家具、耐用消费品及电器的支出（包括电冰箱、洗衣机、电视、电脑和高档乐器如钢琴等） ____ **(GE010_4)** Yuan 元
5. Education and training(including tuition, training fees, etc.) 教育和培训支出（包括学杂费、培训费等） ____ **(GE010_5)** Yuan 元
6. Medical expenditure 医疗支出（包括直接或间接。注：间接医疗支出，指因为医疗而产生的交通费、营养费、家人陪护花费等。） ____ **(GE010_6)** Yuan 元
7. Fitness expenditures 保健费用（包括健身锻炼及产品器械、保健品等） ____ **(GE010_7)** Yuan 元
8. Beauty (including make-ups, facials, massages, etc.) 美容支出（包括化妆品、美容护理、按摩等） ____ **(GE010_8)** Yuan 元
9. Automobiles 购买汽车 ____ **(GE010_9)** Yuan 元
10. Purchase, Maintenance and repair (of transportation vehicles, appliances, communication products, etc.) 各种交通工具（如自行车、电动自行车等，不包括汽车）、通讯工具（如电话、手机等）的购买、维修及配件费用 ____ **(GE010_10)** Yuan 元

11. Property management fees (including parking fee) 物业费 (包括车位费) ____ (**GE010_11**) Yuan 元
12. Taxes and fees turned over to the government 上交给政府相关部门的税费和杂费（不包括所得税）____ (**GE010_12**) Yuan 元
13. Donations to the society (including cash, and items like food, clothing, etc.) 社会捐助支出（包括现金，食品、衣服等）____ (**GE010_13**) Yuan 元

GE011 How often did the respondent receive assistance in answering section Household income and expenditure? 受访者填写该部分问卷时是否求助？

[IWER: If it is answered by a proxy, please record the respondent's reaction. 访员注意：如果是协助回答，请记录受访者的反应。]

1. Never 从未
2. A few times 偶尔几次
3. Most or all of the time 大多数时间

HA HOUSEHOLD ASSETS 家户资产

[IWER: This section is asked of the family respondent. Do not allow a proxy respondent to answer the entire section. 访员注意：这部分要问家庭受访者，不允许完全请别人代答。]

PART 1 Current Residence 第一部分：现有住宅

The following questions pertain to your current residence. 下面是关于您现住宅的问题。

HA000_W2 Where is your current house located? 您家现在住的房子的地址是 __________ (**HA000_1_PSU/HA000_1_PSU_OTHER**) Province/District 省市/区，Zip Code 邮编是 ______(**HA000_2**)，小区/自然村名称是 ___________ (**HA000_3**)。

HA000_W2_0 访员填写：受访者当前居住住宅类型是？

1. 一般居民住宅
2. 养老院/敬老院，名称 ____ (**HA000_w2_0_1**) → skip to HA026_W2 Branchpoint 跳至 HA026_W2 Branchpoint
3. 医院，名称 ____ (**HA000_w2_0_2**) → skip to HA026_W2 Branchpoint 跳至 HA026_W2 Branchpoint
4. 干休所，名称 ____ (**HA000_w2_0_3**) → skip to HA026_W2 Branchpoint 跳至 HA026_W2 Branchpoint
5. 其他机构，请注明机构类型和名称，____ (**HA000_w2_0_4**) → skip to HA026_W2 Branchpoint 跳至 HA026_W2 Branchpoint

HA000_W2_1 BRANCHPOINT :

IF THIS IS A NEW INTERVIEWED HOUSEHOLD, GO TO HA001 如果是新的受访户，请询问HA001.

IF THIS IS A REINTERVIEWED HOUSEHOLD, GO TO HA000_W2_1 如果是老受访户，请询问HA000_W2_1.

HA000_W2_1 这所房子是上次访问时 [ZIWTime]，您居住的房屋 [加载上一轮调查时现有住宅的特征] 吗？

1. Yes 是 → skip to HA001_W2 跳至 HA001_W2
2. No 否

HA001 When did your household start to live at your current residence? 您家何时开始住进现在的房子？ ____1900...2013 Year 年
[IWER: Mark the year using four digits. 访员注意：用 4 位数表示年。]

HA001_W2 What is the construction area of the house? 这房子的建筑面积是多大？ ____ m^2 平方米 [soft check<10 or >500]

HA002 Do you pay rent for your current residence? 您家现在住的房子要付租金吗？

1. Yes 要
2. No 不要 → skip to HA005 跳至 HA005

HA003 How much rent do you pay each month? 如果要付租金，一个月付多少钱？ _____ Yuan / month 元/月 [soft check: <100, >10,000 yuan]

HA004 Did you pay less than the market rental value? 您家支付的租金是否低于市场价？

1. Yes 是
2. No 否 → skip to HA006 跳至 HA006

HA005 If you rented the same housing unit from the market, what is the rent per month you would have to pay? 如果从市场上租同样的房子，每月的租金会是多少？ ____ Yuan/month 元/月 [soft check: <100, >10,000 yuan]

HA006 How much of the rent was paid by a housing subsidy from the employer of a household member? which household member? 您家户成员的单位提供多少住房补贴来支付租金？ _____ (**HA006_1**)yuan 元 [soft check >10,000 yuan] ，哪几个家户成员？ ____ (**HA006_2**) [preloaded list 从前面提取家户成员名单]

HA007 Who owns your current residence? 您家现在的房子归谁所有？

1. 完全归家户成员所有
2. 部分归家户成员所有
3. 完全不归家户成员所有 → skip to HA010 跳至 HA010

HA007_W2_1 [CAPI: Ask this question only when the earlier records show that ONE of the respondent (main respond OR the spouse passed away in the last two years 只有从前面问卷中，发现主要受访者与其配偶中，有一人去世的情况，才问此问] Has the owndership status changes following the death of [preload the name of the deceased main respondent or spouse] 在过去两年内，有没有因为 [加载去世的主要受访者或配偶的名字] 的去世，而导致现在这所房子的所有权状况发生改变？

1. Yes 有

2. No 没有 → skip to HA008 跳至 HA008

HA007_W2_2 在去世前，[加载去世的主要受访者或配偶的名字] 拥有该房产的比重是 __ %.

HA007_W2_3 [加载去世的主要受访者或配偶的名字] 拥有的那部分房产，是如何处置的？继承的比重分别是?

[注意，这里问的是去世受访者拥有的产权部分的继承比重分配。比如去世受访者拥有 60 他将自己产权的 5050 的继承情况，并未询问家庭成员目前拥有的产权情况。]

[Hard check: 以下各项总和不能超过 100]

1. 由配偶继承，___ **(HA007_w2_3_1[1])**
2. 子女、女婿、儿媳，___ **(HA007_w2_3_1[2])**
3. 兄弟姐妹，___ **(HA007_w2_3_1[3])**
4. 其他亲戚，___ **(HA007_w2_3_1[4])**
5. 孙子/女，___ **(HA007_w2_3_1[5])**
6. 朋友，___ **(HA007_w2_3_1[6])**
7. 慈善机构，___ **(HA007_w2_3_1[7])**
8. 其他，___ **(HA007_w2_3_1[8])**

PROCEDURE 程序:
如果继承人包含了子女（HA007_W2_3 = 2），提问 HA007_W2_4 和 HA007_W2_5

HA007_W2_4 是哪些子女？[Load 子女名称让 proxy 选择，可多选]。

HA007_W2_5 [加载 HA007_W2_4 中的子女姓名，逐个询问] 继承的产权，占 [加载去世的主要受访者或配偶的名字] 生前拥有产权比重的 ___ %?

PROCEDURE 程序:
如果继承人包含了孙子女（HA007_W2_3 = 5），提问 HA007_W2_6 和 HA007_W2_7

HA007_W2_6 孙子女是哪些子女的孩子？[Load 子女名称让 proxy 选择，可多选]。

HA007_W2_7 [加载 HA007_W2_6 中的子女姓名，逐个询问] 的孩子继承的产权，占 [加载去世的主要受访者或配偶的名字] 生前拥有产权比重的 __ %？

HA008 Which household member(s) own the house? (preloaded names of household members) 哪一个/些家户成员拥有这处房子？ ____ [从前面提取家户成员名单]

HA009 What share of the house is owned by [preloaded names of household members]?（加载 HA008 的答案）拥有产权的比重是？ ____ 0.00...100.00 % [hard check: range 0-100]

PROCEDURE 程序:
Skip to HA011. 跳至 HA011

HA010 Which non-household members own all or part of your current residence? (circle all that apply) 您家现在的房子是哪些非家户成员的？（可多选）

1. Working unit of household member, 家户成员单位的，which household member? ____ (**HA010_1**) 哪一个家户成员的单位 [preloaded list] [从前面提取家户成员名单]
2. Government indemnificatory housing 政府保障性住房
3. Child(non- household member) of main respondent or spouse 主要受访者（或其配偶）的非家户成员的孩子, which child? 哪个孩子？ ____ (**HA010_2**) [preloaded list] [从前面提取孩子的名单]
4. Parent(non- household member) of main respondent or spouse 主要受访者（或其配偶）的非家户成员的父母 ____ (**HA010_3**) [preload list] [从前面提取父母的名单]
5. Nonresident other relatives 没在一起居住的其他亲戚
6. Friends 朋友
7. Other 其他人

HA011 What is the present market value of your house? Or, what is the present market value of a similar housing unit within its neighborhood? 这所房子当前的市场价是多少？或者说，这房子附近类似的房子当前的市场价是多少？

Total price 总价格 ____ (**HA011_1**) 10000 Yuan 万元 [soft check < 10, > 500]

Or unit price 或单位价格 ____ (**HA011_2**) 1000 Yuan/m^2 千元/平方米

[IWER: Skip to HA013 if R answered HA011. If not, ask unfolding brackets. 如果答卷人回答了HA011，跳至HA013，否则，提问分级展开问题。]

HA012 [IWER: If R is unwilling to answer or does not remember, ask unfolding bracket questions here. 访员注意：如果受访者不愿回答或者忘记了，在此处分级展开提问]

20,000/50,000/100,000/200,000/500,000 yuan 元

PROCEDURE 程序:

If HA007 $= 2/3$, then skip to HA025. 如果 HA007 $= 2/3$，请跳至 HA025。

HA013 Do you or other household members take out a bank loan to finance the purchase, construction, or decoration of your house now? 您家是否正在用按揭贷款来购买、建造或装修您的房子？（已还清贷款不算）

1. Yes 是
2. No 否 → go back HA016 返回到 HA016

HA014 What is the outstanding amount of the loans? 连本金带利息，还有多少贷款没还清？ ____ 10,000 Yuan 万元 [soft check: >500,000 yuan]

HA014_W2 How much is the unpaid interest? 其中应付利息是 ____ 10,000 Yuan 万元

HA015 What is the monthly mortgage payment? 每月要还多少贷款？ ____ Yuan 元 [soft check: >20000]

HA016 How was this housing unit obtained? 房子的来源是什么？

1. Purchased from market 是从市场买来的

2. Purchased from working unit of household member(s) 从家户成员的单位买来的. 哪一个家户成员的单位 ____? **(HA016_1)** [preloaded list] [从前面提取家户成员名单]
3. Purchased by child (non-household member) of main respondent or spouse, which child? 由主要受访者（或其配偶）的非家户成员的孩子购得，哪个孩子？____ **(HA016_2)** [preloaded list] [从前面提取名单]
4. Purchased by Parents(non- household member), of who (main respondent or spouse) ? 由主要受访者（或其配偶）的非家户成员的父母购得，谁的（主要受访者还是其配偶的）? ____ **(HA016_3)**
5. Purchased from Other relatives 从其他亲戚那里购得
6. Self-built 自建
7. Inherited, bequeathed, or given 继承（如祖业）或受赠与
8. Received home as compensation for demolition of old home 拆迁换房
9. Other 其他 → skip to HA020 请跳至 HA020

HA017 When did you purchase it? 这房子什么时候买/建/换/继承/受赠的? ____ 1900...2013 Year 年 [IWER: Mark the year using four digits. 访员注意: 用 4 位数表示年。]

PROCEDURE 程序:
If HA016 = 7 , then skip to HA020. 如果 HA016 = 7， 请跳至 HA020.

HA018 How much of your own money did you spend on this housing? 买/建/换这房子您家自己付了多少钱? ____ 10,000 Yuan 万元 [soft check >500]

PROCEDURE 程序:
If HA016 = 6 , then skip to HA024. 如果 HA016 = 6， 请跳至 HA024.
If HA016 = 8 , then skip to HA022. 如果 HA016 = 8， 请跳至 HA022.

HA019 Was it purchased at market price, subsidized by working unit, or as purchased as economical housing? 这房子是以市场价购买、单位优惠价格买的、或者是经济适用房?
1. Market price 以市场价购买 → skip to HA024 请跳至 HA024
2. Subsidized by working unit 单位优惠价格买的
3. Economic housing 经济适用房
4. Other 其他

HA020 What would you have to pay if you had paid a market-set price for the same housing? 同样房子当时的市场价格是多少? Total price 总价格 ____ **(HA020_1)** 10,000 Yuan 万元 [soft check < 10, > 500] or unit price 或单位价格 ____ **(HA020_2)** 1,000 Yuan/m^2 千元/平方米 [soft check: < 1, > 25]

PROCEDURE 程序:
If HA020_1 >= 500, or HA020_1 < 10, or HA020_2 < 1, or HA020_2 > 25, ask HA020_check. 如果 HA020_1 >= 500， 或者HA020_1 < 10， 或者 HA020_2 < 1， 或者 HA020_2 > 25， 询问 HA020_check。

HA020_check I heard that the total price of your have paid a market-set price of [preload HA020_1] 10,000 Yuan, is this correct? [Or] I heard that the unit price of your house is [preload HA020_2] 1,000 Yuan/square meter, is this correct? 我听您说当时支付的市场价格是 [preload HA020_1] 万元 / [preload HA020_2] 千元每平米，对吗？

1. Yes 是
2. No 否 → go back HA020 返回到HA020

PROCEDURE 程序:

Ask HA022 – HA023_check only if HA016 chooses 8.

HA022 How much was the market value of the old house at that time? 您家老房子当时的市场价格是多少？Total price 总价格 ____ 10,000 Yuan 万元 [soft check: < 1, > 500]

PROCEDURE 程序:

If HA022 < 1, orHA022 $>= 500$, ask HA022_check. 如果 HA022 < 1，或者HA022 $>= 500$，询问 HA020_check。

HA022_check I heard that you paid a total price of [preload HA022] 10,000 Yuan, is this correct? 我听您说您当时支付的市场价格是 [加载HA022的答案] 万元，对吗？

1. Yes 是
2. No 否 → go back HA022 返回到HA022

HA023 How much was the market value of the new house at that time? 换的新房子当时的市场价格是多少？Total price 总价格 ____ (**HA023_1**) 10,000 Yuan 万元 [soft check: <10, >500] or unit price 或单位价格 ____ (**HA023_2**) 1,000 Yuan/m^2 千元/平方米 [soft check: <1, >25]

PROCEDURE 程序:

If HA023_1 ≥ 500, or HA023_1 < 10, or HA023_2 < 1, or HA023_2 > 25, ask HA023_check. 如果 HA023_1 ≥ 500，或者HA023_1 < 10，或者 HA023_2 < 1，或者HA023_2 > 25，询问 HA023_check。

HA023_check I heard that the market value of the new house is [preload HA023_1] 10,000 Yuan at that time, is this correct? 我听您说您换的新房当时的市场价格是 [加载 HA023_1 的答案] 万元，对吗？

1. Yes 是
2. No 否 → go back HA023 返回到HA023

HA024 Can you sell the house freely? 你家是不是可以自由卖房？

1. Yes 是的
2. No, restricted by work unit 不是，受到单位的限制

HA025 How much did you spend on decorating or renovating all of your houses (exclude expenditures on furniture) in the past year? 您家装修过房子吗？过去一年，您装修名下所有的房屋一共花了多少钱（不包括家具开销）？____ Yuan 元 [soft check: >500,000 yuan]

HA025_W2 How much did you spend on remodelling all of your houses (exclude expenditures on decorating) in the past year? 您家翻盖过房子吗？过去一年，您翻盖名下所有的房屋一共花了多少钱（不包括装修费用）？ ________ Yuan 元 [soft check: >500,000 yuan] [访员注意：翻盖指把老房子拆了，在原有的地皮或地基上建一个新房。多见于农村自建住房。]

**HA026_W2 BRANCHPOINT**:<br>新受访者，跳至 HA027。<br>老受访者，如果 HA000_W2_1 = 1，跳至 PART 2.

[我们的数据显示，您家现在居住的住宅与两年前不同。我们想询问一下上次访问时您家所居住住宅的处置情况（HA026_W2_1[25]-HA026_W2_14_bracket[25]）
HA026_W2_1-HA026_W2_14_bracket为一个循环]

HA026_W2_1 您上次访问时回答的 [下载上次访问时的相关住宅信息] 住宅是怎么处置的？

1. 现在自己住着 → 结束本循环
2. Empty 空着 → 结束本循环
3. Sold 出售
4. Rented out 出租 → 结束本循环
5. Being torn down 被拆迁 → skip to HA026_W2_6 跳至 HA026_W2_6
6. My child(ren) stay in it 子女住着 → skip to HA026_W2_9 跳至 HA026_W2_9
7. My parent(s) stay in it 父母住着 → skip to HA026_W2_9 跳至 HA026_W2_9
8. Others (relatives, friends) stay in it 给其他人 (亲戚，朋友) 居住 → skip to HA026_W2_9 跳至 HA026_W2_9
9. Give to others as gift 赠予他人 → skip to HA026_W2_12 跳至 HA026_W2_12
10. Last residence is rented, no longer rent it 上次住宅是租来的，现已不再租用 → 结束本循环
11. Other, please specify 其它，请注明 ____ (**HA026_W2_1a**) → 结束本循环
12. Last record is wrong, the mentioned residence is not ours 上次记录有误，提及的住宅不是我家的 → 结束本循环

HA026_W2_2 When did you sell that house? ____ Year __ Month 您何时出售的该住宅？ ____ (**HA026_W2_2_1**) 年 __ (**HA026_W2_2_2**) 月

HA026_W2_3 What is the net income you got from selling the house, excluding relative fees? 扣除相关费用，您出售该住宅后获得的净收入是多少？ Total price 总价格 ____ 10,000 Yuan 万元 [soft check < 0.1, > 500]

HA026_W2_4 [IWER: If R is unwilling to answer or does not remember, ask unfolding bracket questions here. 访员注意：如果受访者不愿回答或者忘记了，在此处分级展开提问] 20,000/50,000/100,000/200,000/500,000 yuan 元

HA026_W2_5 How did you distribute the income from selling this house? (Can be multiple choice, ask the respondent to fill in corresponding percentages.) 您如何处理卖房所得的收入？ [下列选项可以多选，有下列处置方式的请受访者回答相应比例]

1. Deposits in bank account 存入银行账户，占售房总收入的 ___ (**HA026_W2_5a**) % of total income
2. Everyday living expenses 用于日常生活支出，占售房总收入的 ___ (**HA026_W2_5b**) % of total income
3. Buying new houses 用于购买新房，占售房总收入的 ___ (**HA026_W2_5c**) % of total income
4. Medical expenditure 用于医疗支出，占售房总收入的 ___ (**HA026_W2_5d**) % of total income
5. Other important expenses 用于其他重大支出，占售房总收入的 ___ (**HA026_W2_5e**) % of total income
6. Trasfer to Children 赠予儿女，占售房总收入的 ___ (**HA026_W2_5f**) % of total income
7. Transfer to parents 赠予父母，占售房总收入的 ___ (**HA026_W2_5g**) % of total income
8. Transfer to relatives 赠予亲戚，占总收入的 ___ (**HA026_W2_5h**) % of total income
9. ther, please specify 其他，请注明 ___ (**HA026_W2_5i**)，占总收入的 ___ (**HA026_W2_5j**) % of total income

[CAPI: 结束本循环]

HA026_W2_6 Did you receive any compensation for tearing down the house? 这处房屋被拆迁，您家是否获得拆迁补偿款？

1. Yes 是
2. No 否 → 结束本循环

HA026_W2_7 When did you receive the compensation for tearing down the house? 您家何时获得了拆迁补偿款？ ____ Year 年 __ Month 月

HA026_W2_8 What is the total amount of the compensation? 您家获得的拆迁补偿款的总额是多少？ ____ 10,000 Yuan 万元

HA026_W2_8_bracket [IWER: If R is unwilling to answer or does not remember, ask unfolding bracket questions here. 访员注意：如果受访者不愿回答或者忘记了，在此处分级展开提问] 20,000/50,000/100,000/200,000/500,000 yuan 元

[CAPI: 结束本循环]

HA026_W2_9 Have those who live in your house pay you any cash, or provide you in-kind payment? 住您房屋的人，有没有给您现金或者实物的补偿？

1. Yes 是
2. No 否 → 结束本循环

HA026_W2_10 How did those who live in your house pay you the cash or the in-kind payment? 住您房屋的人是如何给您现金或者实物的补偿的？

1. One lump-sum payment 一次性补偿

2. Monthly payment 每月支付
3. Irregular payment over the year 不定期补偿

HA026_W2_11 What is the total amount did those who live in your house pay you in the past year? 过去一年，住您房屋的人给您的补偿是 ____ (**HA026_W2_11a**) Yuan 元？Among it, cash is 其中，现金 ____ (**HA026_W2_11b**) Yuan 元，in-kind payment is 实物值 ____ (**HA026_W2_11c**) Yuan 元？

HA026_W2_11_bracket [IWER: If R is unwilling to answer or does not remember, ask unfolding bracket questions here. 访员注意：如果受访者不愿回答或者忘记了，在此处分级展开提问] 20,000/50,000/100,000/200,000/500,000 yuan 元

[CAPI: 结束本循环]

HA026_W2_12 Have those who got your house as gifts pay you any cash, or provide you in-kind payment? 获得您赠予房产的人，有没有给您现金或者实物的补偿？

1. Yes 是
2. No 否 → 结束本循环

HA026_W2_13 How did those who got your house as gifts pay you the cash or the in-kind payment? 获得您赠予房产的人是如何给您现金或者实物的补偿的？

1. One lump-sum payment 一次性补偿
2. Monthly payment 每月支付
3. Irregular payment over the year 不定期补偿

HA026_W2_14 What is the total amount did those who got your house as a gift pay you in the past year? 过去一年，获得您赠予房产的人，给您的补偿是 ____ (**HA026_W2_14a**) Yuan 元？Among it, cash is 其中，现金 ____ (**HA026_W2_14b**) Yuan 元，in-kind payment is 实物值 ____ (**HA026_W2_14c**) Yuan 元？

HA026_W2_14_bracket [IWER: If R is unwilling to answer or does not remember, ask unfolding bracket questions here. 访员注意：如果受访者不愿回答或者忘记了，在此处分级展开提问] 20,000/50,000/100,000/200,000/500,000 yuan 元

[CAPI: 循环结束]

**PROCEDURE 程序:**
老受访者，请跳过 HA027 - HA028。

HA027 Excluding the house in which you live, do you or members of your household own any other residential properties? 除了您家现在的住房以外，您或其他家户成员是否拥有其他房产（是否拥有以您或其他家户成员是否是房主为准）？

1. Yes 是
2. No 否 → skip to HA054 跳至 HA054

HA028 How many other housing units do you or members of your household currently own? 您或其他家户成员还有几处其他的房产？ __ 0...10 处

PART 2 Other Residences 第二部分：其他房产

[For every other housing unit owned by house members, ask the following questions, 对所有家户成员拥有的其他的每处房产，询问以下的问题：]

**HA029_W2 BRANCHPOINT**:
IF THE HOUSEHOLD IS A NEW INTERVIEW HOUSEHOLD, THEN SKIP TO HA029. 如果是新家户，跳至 HA029
IF THE HOUSEHOLD IS A RE-INTERVIEW HOUSEHOLD, SKIP TO HA029_W2. 如果是老家户，回答 HA029_W2

HA029_W2 In the past two years since [preload the year of last wave] Year [preload the month of last wave] month, have your household obtained (purchased/built/inherited/received as gift/got new house due to tearing down) any new houses? 自从上次访问 [ZIWTime] 以来，您家有没有得到（购买/新建/继承/受赠/拆迁换房等）新的房产（前面回答过的现有住宅除外）？

1. Yes, obtained 是，得到了 __ 0...10 (**HA029_W2_1**) houses 处
2. No 否 → skip to HA051_W2_1 Branchpoint 跳至 HA051_W2_1 Branchpoint

[CAPI：HA029-HA051 为一个循环]

HA029 Now we want to know information about other housing unit(s) owned by your household members. 现在我们想了解有关你家第 n 处（对老受访者提示：两年来新增的）其他房产的情况 [CAPI: If this is new respondent, then n starts with 1, loop until the number recorded in HA028. 如果是新受访者，则 n 从 1 开始，到HA028 记录的房产数为止。If this is R from last wave and select 1 in HA029_W2, then n starts from 1, ends at the number given by HA029_w2_1. 如果是老受访者，且HA029_W2=1，则 n 从 1 开始，到 HA029_w2_1 记录的房产数为止。If this is R from last wave and select 2 in HA029_W2, then skip to HA051_W2_1. 如果是老受访者，且HA029_W2=2，则跳至 HA051_W2_1 前的程序控制]

Where is this house located? 该房的地址是 ___________ (**HA029_1_PSU/HA029_1_PSU_OTHER**) Province/District 省市/区，Zip Code 邮编是 ______ (**HA029_2**)，小区/自然村名称是 ___________ (**HA029_3**)。

HA030 Who owns this residence? 这所房子归谁所有？

1. Owned completely by your household member(s). 完全归家户成员所有
2. Owned partly by your household member(s). 部分归家户成员所有

HA030_W2_1 [CAPI: Ask this question only when the earlier records show that ONE of the respondent (main respond OR the spouse passed away in the last two years 只有从前面问卷中，发现主要受访者与其配偶中，有一人去世的情况，才问此问。] Has the owndership status changes following the death of [preload the name of the deceased main respondent or spouse]? 在过去两年内，有没有因为 [加载去世的主要受访者或配偶的名字] 的去世，而导致现在这所房子的所有权状况发生改变？

1. Yes 有
2. No 没有 → skip to HA031 跳至HA031

HA030_W2_2 在去世前，[加载去世的主要受访者或配偶的名字] 拥有该房产的比重是 __ %.

HA030_W2_3 [加载去世的主要受访者或配偶的名字] 拥有的那部分房产，是如何处置的？继承的比重分别是？[注意，这里问的是去世受访者拥有的产权部分的继承比重分配。比如去世受访者拥有 60% 的产权，他将自己产权的 50% 给了配偶，另外 50% 平均分配给两个儿女。那么这里要填的比重分别是配偶 50%，子女 50%。另外，回答完该问之后，家庭成员仍然需要回答 HA030，因为本问题只询问产权的继承情况，并未询问家庭成员目前拥有的产权情况。]
[Hard check: 以下各项总和不能超过 100]

1. 由配偶继承，___ (**HA030_w2_3_1[1]**)
2. 子女、女婿、儿媳，___ (**HA030_w2_3_1[2]**)
3. 兄弟姐妹，___ (**HA030_w2_3_1[3]**)
4. 其他亲戚，___ (**HA030_w2_3_1[4]**)
5. 孙子/女，___ (**HA030_w2_3_1[5]**)
6. 朋友，___ (**HA030_w2_3_1[6]**)
7. 慈善机构，___ (**HA030_w2_3_1[7]**)
8. 其他，___ (**HA030_w2_3_1[8]**)

PROCEDURE 程序:
如果 HA030_W2_3 中继承人选包含了子女，询问 HA030_W2_4 和 HA030_W2_5

HA030_W2_4 是哪些子女？[Load 子女名称让 proxy 选择，可多选]

HA030_W2_5 [加载 HA030_W2_4 中的子女姓名，逐个询问] 继承的产权，占 [加载去世的主要受访者或配偶的名字] 生前拥有产权比重的 __ % ?

PROCEDURE 程序:
如果继承人选包含了孙子女（HA030_W2_3 =5），询问 HA030_W2_6 和 HA030_W2_7

HA030_W2_6 孙子女是哪些子女的孩子？[Load 子女名称让 proxy 选择，可多选]。

HA030_W2_7 [加载 HA030_W2_6 中的子女姓名，逐个询问] 的孩子继承的产权，占 [加载去世的主要受访者或配偶的名字] 生前拥有产权比重的 __ % ?

HA031 Which household member(s) own the house? [preloaded names of household members] 哪一个/些家户成员拥有这处房子？____ [从前面提取家户成员名单]

HA032 What share of the house is owned by [preloaded names of household members]? [加载 HA031 的回答] 拥有产权的比重是？_____ %0.00...100.00% [hard check: range 0-100]

PROCEDURE 程序:
If HA030 = 1, skip HA033

HA033 What non-household members own part of your current residence? [circle all that apply] 您家现在的房子是哪些非家户成员的？(可多选)

1. Working unit of household member, 家户成员单位的，which household member? 哪一个家户成员的单位 [preloaded list][从前面提取家户成员名单] ____ (**HA033_1**)
2. Government indemnificatory housing 政府保障性住房
3. Child(non- household member) of main respondent or spouse 主要受访者（或其配偶）的非家户成员的孩子, which child? 哪个孩子？ ____ (**HA033_2**) [preloaded list] 从前面提取孩子的名单
4. Parent(non- household member) of main respondent or spouse 主要受访者（或其配偶）的非家户成员的父母 ____ (**HA033_3**) [preload list] 从前面提取父母的名单
5. Nonresident other relatives 没在一起居住的其他亲戚
6. Friends 朋友
7. Other 其他人

PROCEDURE 程序:

If HA031 does not include respondent or spouse, skip to Next house. 如果 HA031 答案不包括受访者及其配偶，请跳至下一处房产

HA034 What is the present market value of your house? Or, what is the present market value of a similar housing unit within its neighborhood? 当前的市场价是多少？或者说，这房子附近类似的房子当前的市场价是多少？Total price 总价格 ____ (**HA034_1**) 10,000 Yuan 万元 [soft check <10, >500] or unit price 或单位价格 ____ (**HA034_2**) 1,000Yuan/m^2 千元/平方米 [soft check<1, >25]

[CAPI: Skip to HA036 if R answered HA034. If not, ask unfolding brackets. 如果答卷人回答了HA034，跳至HA036，否则，提问分级展开问题]

PROCEDURE 程序:

If HA034_1 $>=$ 500 or HA034_1 $<$ 10, or HA034_2 $<$ 1 or HA034_2 $>$ 25, ask HA034_check. 如果 HA034_1 $>=$ 500，或者 HA034_1 $<$ 10，或者HA034_2 $<$ 1，或者HA034_2 $>$ 25，询问 HA034_check。

HA034_check I heard that the present market value of your house is [preload HA034_1] 10,000 Yuan, is this correct? 我听您说您房子的市场价是 [加载 HA034_1 的答案] 万元，对吗？

1. Yes 是
2. No 否 → go back HA034 返回到 HA034

HA035 [IWER: If R is unwilling to answer or does not remember, ask unfolding bracket questions here. 访员注意：如果受访者不愿回答或者忘记了，在此处分级展开提问] 20,000/50,000/100,000/200,000/500,000 yuan 元

HA036 Do your household members take out a bank loan to finance the purchase, construct, or decorate this house? 您家是否正在用按揭贷款来购买、建造或装修这所房屋？(如曾按揭贷款买房，但已还清不算)

1. Yes 是
2. No 否 → Skip to HA039 请跳至HA039

HA037 What is the outstanding amount of the loans? 还有多少贷款没还清？____ 10,000 Yuan 万元 [soft check >500]

HA038 What is the monthly mortgage payment? 每月要还多少贷款？____ Yuan 元 [soft check >20,000]

HA039 How was this housing unit obtained? 房子的来源是什么？
1. Purchased from market 是从市场买来的
2. Purchased from working unit of respondent or spouse 从受访者或其配偶的单位买来的，谁的单位 ____ (**HA039_1**)？[preloaded list] [从前面提取受访者或其配偶的名单]
3. Purchased by child of main respondent or spouse, which one? 由主要受访者（或其配偶）的孩子购得，哪一个 ____ (**HA039_2**) [preload list] [提取孩子名单]
4. Purchased by parents of main respondent or spouse, of who（main respondent or spouse）? 由主要受访者（或其配偶）的父母购得，谁的（主要受访者还是其配偶的）父母 ____ (**HA039_3**)
5. Purchased from Other relatives 从其他亲戚那里买的
6. Self-built 自建 → Skip to HA041 请跳至 HA041
7. Inherited, bequeathed, or given 继承（如祖业）或受赠与 → Skip to HA041 请跳至 HA041
8. Received home as compensation for demolition of old home, 拆迁换房 → Skip to HA041 请跳至HA041
9. Other 其他 → Skip to HA045 请跳至 HA045

HA040 Was it purchased at market price, subsidized by working unit, or as purchased as economical housing? 这房子是以市场价买、单位优惠价格买的、或者是经济适用房？
1. Market price 以市场价购买
2. Subsidized by working unit 单位优惠价格买的
3. Economic housing 经济适用房
4. Other 其他

HA041 When did you purchase/build/inherit it? 这所房子是什么时候买/建/换/继承/受赠的？____ 1900...2013 Year 年
[IWER: Mark the year using four digits. 访员注意：用 4 位数表示年。]

HA042 Can you sell the house freely if you want? 如果想卖的话，您家是不是可以自由卖房？
1. Yes 是的
2. No, restricted by work unit 不是，受到单位的限制

PROCEDURE 程序:
If HA039 = 7 , then skip to HA045. 如果 HA039 = 7， 请跳至 HA045。

HA043 How much of your own money did you spend on the house (including loan-financed)? 买/建/换这所房子你家花了多少钱? ____ 10,000Yuan 万元 [soft check<10, >500]
[IWER: Skip to HA045 if R answered HA043. If not, ask unfolding brackets. 如果答卷人回答了 HA043，跳至 HA045，否则，提问分级展开问题。]

PROCEDURE 程序:
IF HA043 >= 500 or HA043 < 10, ask HA043_check. 如果 HA043 >= 500，或者 HA043 < 10，询问HA043_check。

HA043_check I heard that you had spend [preload HA043] 10,000 Yuan on the house, is this correct? 我您说您买/建/换这所房子时花了 [加载 HA043 的答案] 万元，对吗?
1. Yes 是
2. No 否 → go back HA043 返回到 HA043

HA044 [IWER: If R is unwilling to answer or does not remember, ask unfolding bracket questions here. 访员注意：如果受访者不愿回答或者忘记了，在此处分级展开提问] 10,000/20,000/50,000/100,000/200,000 yuan 元

PROCEDURE 程序:
If HA040 = 1, then skip to HA051. 如果 HA040 = 1，请跳至HA051。
If HA039 = 6, then skip to HA051. 如果 HA039 = 6，请跳至HA051。

HA045 What would you have to pay if you had paid a market-set price for the same housing? 同样房子当时的市场价格是多少? Total price 总价格 ____ (**HA045_1**) 10,000 Yuan 万元 [soft check >500] or unit price 或单位价格 ____ (**HA045_2**) 1,000 Yuan/m^2 千元/平方米 [soft check >30]

PROCEDURE 程序:
If HA045_1 >= 500，ask HA045_check. 如果 HA045_1 >= 500，询问 HA045_check。

HA045_check I heard that you had paid a market-set price [preload HA045_1] 10,000 Yuan for the same housing, is this correct? 我听您说同样房子在当时的市场价是 [加载 HA045_1 的答案] 万元，对吗?
1. Yes 是
2. No 否 → go back HA045 返回到 HA045

HA051 What is the construction area of the house? 这房子的建筑面积是多大? ____m^2 平方米 [soft check<10 or >500]

[Skip to next house until the last house 跳至下一处房产，直至循环结束]

[CAPI：下载上一轮其他房产的信息，对于每一处，询问 HA026_W2_1-HA026_W2_14_bracket 的循环 (循环序号从 1 开始)。循环结束之后，跳至HA052。]

HA051_W2_1 BRANCHPOINT:
IF THE HOUSEHOLD IS A NEW INTERVIEW HOUSEHOLD, SKIP TO HA052 如果该家户是新家户，则跳至HA052

[现在我们想了解一下上次访问时您提到的其他房产的情况。]

HA052 What is the monthly rental income for all room or houses owned by main respondent or spouse, that you are currently leasing? 主要受访者（或其配偶）拥有的房屋，正在出租的，每月一共能收取多少房租？

1. ____ (**HA052_1**) Yuan/month 元/月 [soft check >20,000]
2. Not applicable 没有出租

HA053 What is the monthly rental income for all room or houses owned by other household members that are currently being leased? 其他家户成员拥有的房屋，正在出租的，每月一共能收取多少房租？

1. ____ (**HA053_1**) Yuan/month 元/月
2. Not applicable 没有出租

PART 3 Land 第三部分：土地

[The following questions pertain to your land. 下面是关于土地的问题]

HA054 Does your household have any collective distributing or rent cultivated land, forest land, pasture and/or pond? (Choose all that apply) 您家是否有集体分配的耕地、林地、牧场或水塘，或者从别人那里租用了耕地、林地、牧场或水塘？(可多选)

1. Cultivated land 耕地 (**HA054_1**)
2. Forest land 林地 (**HA054_2**)
3. Pasture 牧场 (**HA054_3**)
4. Pond 水塘 (**HA054_4**)
5. None 没有 (**HA054_5**) → Skip to HA064 请跳至HA064

PROCEDURE 程序:
According to all options choosed in HA054 , ask HA055 -HA063 in loop. 针对 HA054 的所有选择，循环询问 HA055 -HA063 题。

HA055 How many mu of [preload answer fromHA054] do you have? 您家从集体分配到的 [加载 HA054 的答案] 有多少亩？ ____ Mu 亩 [soft check > 50]

HA056 How many mu of them are irrigable? 其中可灌溉面积是多少亩？ ____Mu 亩 [hard check cannot be > HA055]

HA057 What is the rent per mu per year you would get if you rent out all your [preload answer from HA054]? 如果出租的话，每亩 [加载 HA054 的答案] 每年的租金会是多少？ ____ Yuan per mu per year 元每年每亩 [soft check < 10, > 4000]

HA058 Did you rent out any of your [preload answer from HA054] in the past year? 过去一年您家是否将 [加载 HA054 的答案] 出租给了其他人？

1. Yes 是
2. No 否 → Skip to HA061 请跳至 HA061

HA059 How much [preload answer from HA054] did you rent out the past year? 过去一年您家出租了多少亩 [加载 HA054 的答案]？ ____ Mu 亩 [hard check, cannot be > HA055]

HA060 How much rental income did you earn in the past year? 过去一年您家出租 [加载 HA054 的答案] 收到的租金是多少？ ____ Yuan 元

HA061 Did you rent in any [preload answer from HA054] from others (including the collective) in the past year? 过去一年您家是否从别人（包括集体）那租用了 [加载 HA054 的答案]？

1. Yes 是
2. No 否 → Skip toHA064 请跳至 HA064

HA062 How much did you rent in the past year? 过去一年您家租用了多少亩 [加载 HA054 的答案]？ ____ Mu 亩 [soft check > 100]

HA063 How much rent did you pay in the past year? 过去一年您家租用 [加载 HA054 的答案] 付出的租金是多少？ ____ Yuan 元 [soft check >20000]

HA064 How much rental income did you earn for any other household assets other than housing or land? (trees, use of fixed capital, durables, or livestock)? 除了出租房屋或者土地，过去一年您家从出租其他家庭资产（如树木、固定资本的使用、耐用品或者牲畜）收取了多少租金？

1. ____ (**HA064_1**) Yuan 元
2. Not applicable 没有出租

PART 4 Equipments, Consumption durables, and Valuables. 家用设备、耐用消费品和其他贵重物品

[Show Card 出示卡片 31]

HA065 Do members of your household own the following assets? (Choose all that apply) 您家有下列物品吗？（可多选）

For all categories, add [soft check <100 or >30,000] unless other check is written

[For each asset owned by the household] what is the asset's current value? (Yuan) 针对这家有的每一项物品提问：当前值多少？（元）

1. Automobile 汽车 [soft check <3000, >500,000] ____ (**HA065_1[1]**)
2. Electric Bicycle 电动自行车 ____ (**HA065_1[2]**)
3. Motorcycle 摩托车 ____ (**HA065_1[3]**)

4. Refrigerator 电冰箱、冰柜 ____ **(HA065_1[4])**
5. Washing machine 洗衣机 ____ **(HA065_1[5])**
6. TV 电视机 ____ **(HA065_1[6])**
7. Computer 家用电脑 ____ **(HA065_1[7])**
8. Stereo system 组合音响 ____ **(HA065_1[8])**
9. Video camera 摄像机 ____ **(HA065_1[9])**
10. Camera 照相机 ____ **(HA065_1[10])**
11. Air conditioner 空调 ____ **(HA065_1[11])**
12. Mobile phone 手机 ____ **(HA065_1[12])**
13. Furniture 值钱家具 ____ **(HA065_1[13])**
14. Music instrument 高档乐器 ____ **(HA065_1[14])**
15. Valuable decorations, ornaments 昂贵的装饰、物品 ____ **(HA065_1[15])**)
16. Treasures and precious metal (such as gold) 珠宝和贵重金属（如黄金等）__________ **(HA065_1[16])**
17. Antiques, valuable paintings and calligraphic work, and other artistic work 古董、字画及其他艺术品 ____ **(HA065_1[17])**
18. None 没有以上物品

HA065_w2_[1] You just told us that your household owns an automobile/ automobiles, do you or your spouse own the automobile(s)? 您刚刚提到家里有汽车，请问您家的汽车是属于您和您配偶的吗？

1. Yes 是
2. No 否

HA065_w2_[16] You just told us that your household own treasures and precious metal, what percentage among the total value is owned by you and/or your spouse? 您刚刚提到家里有珠宝和贵重金属，请问在总价值中，有多大比例是属于您和您配偶的？____ %
[IWER: 访员注意：此处询问的是，在家户拥有的珠宝和贵金属的总价值中，受访者及其配偶夫妻俩**合计**拥有的比例。例：如果家户拥有价值 10,000 元的黄金，夫妻俩及一个孩子共同拥有，三个人拥有比例相同，则应填写 67%；如果家户拥有价值 10000 元的黄金，夫妻俩共同拥有，两个人拥有比例相同，二人之外的其他家户成员没有所有权，则应填写 100%。]

HA065_w2_[17] You just told us that your household own antiques, valuable paintings and calligraphic work, and other artistic work, what percentage among the total value is owned by you and/or your spouse? 您刚刚提到家里有古董、字画和其他艺术品，请问在总价值中，有多大比例是属于您和您配偶的？____ %
[IWER: 访员注意：此处询问的是，在家户拥有的古董、字画和其他艺术品的总价值中，受访者及其配偶夫妻俩**合计**拥有的比例。例：如果家户拥有价值 10000 元的古董，夫妻俩及一个孩子共同拥有，三个人拥有比例相同，则应填写 67%；如果家户拥有价值 10000 元的黄金，夫妻俩共同拥有，两个人拥有比例相同，二人之外的其他家户成员没有所有权，则应填写 100%。]

HA066 Do members of your household own the following fixed capital assets? How much are the assets worth? (check all that apply) 您家有下列固定资产吗？现在值多少钱？（可多选）

1. Tractor, current value____ Yuan 拖拉机，现在值 ____ (**HA066_1**) 元 [soft check $< 1000, > 30,000$]
2. Thresher, current value____ Yuan 脱粒机 (包括打稻机)，现在值 ____ (**HA066_2**) 元 [soft check < 100 or $> 10,000$]
3. Tractor tools, current value____ Yuan 机引农具，现在值 ____ (**HA066_3**) 元 [soft check < 100 or $> 10,000$]
4. Water pump, current value____ Yuan 抽水机 (包括水泵)，现在值 ____ (**HA066_4**) 元 [soft check < 100 or $> 10,000$]
5. Processing equipment, current value____ Yuan 加工机械，现在值 ____ (**HA066_5**) 元 [soft check < 100 or $> 10,000$]
6. None 没有以上固定资产 ____ (**HA066_6**)

HA067 What is the current value of other fixed capital assets used in household production or self-employed activities? 用于家庭生产、个体经营或开办私营企业的其他固定资产现在值多少钱？ ____ Yuan 元

[IWER: Be sure to ask about fixed capital assets used in all self-employment activities, do not count assets already reported above. 访员注意：务必要询问用于所有家庭生产、个体经营或开办私营企业活动的固定资产，不包括前面问过了的固定资产。]

HA068 Does your household have any other durable or fixed assets worth 500 yuan or more? 您家有其他 500 元及以上的其他耐用品或固定资产吗？

1. Yes 有 How much are the assets worth? 现在值多少？ ____ (**HA068_1**) Yuan 元 [hard check > 500] [soft check $> 50,000$]
2. No 没有

HA069 Have you lent to other families or individuals and not been repaid by them? 现在还有没有人或单位欠您家钱没还的？

1. Yes 有
2. No 没有 → Skip to HA071 请跳至 HA071

HA070 What is the total amount of the loans? 他们一共还欠您家多少钱？ ______ Yuan 元 [soft check $> 500,000$]

HA071 How much interest income from what you lent to others in past year? 过去一年您家借出去的钱一共得到多少利息？ _____ Yuan 元 [soft check $> 50,000$]

HA072 What is the total amount of loans that you are still owing to other families, individuals, or your work unit? (not including mortgage loans) 您家还欠其他家庭、个人或单位的钱总数是多少？（不包括抵押贷款） ____ Yuan 元 [soft check $> 500,000$]

[IWER: Skip to HA074, if R answered HA072, If not, ask unfolding brackets HA073. 访员注意：如果受访者回答了 HA072，跳至 HA074，否则，提问分级展开问题 HA073]

HA073 [IWER: If R is unwilling to answer or does not remember, ask unfolding bracket questions here. 访员注意：如果受访者不愿回答或者忘记了，在此处分级展开提问] 5,000/10,000 /50,000 /100,000 /500,000 yuan 元

[Intro: Next we will ask your household members, other than the main respondent and spouse, some financial questions. 下面，我们将问除主要受访者及其配偶外的家户成员一些财产问题。]

[IWER: The names of other household members not including respondent and spouse are preloaded from the cover screen information. For each member, answer the following questions. 访员注意：其他家户成员不包括主要受访者及其配偶，其他家户成员的名单要从过滤问卷获得。对于每一个其他家户成员，分别问下面的问题。]

HA074 What is the value of all financial assets of [preload household member name] (includes cash, savings, stocks, funds)? [preload household member name] 拥有多少金融资产（包括现金、存款、股票、基金）？____ Yuan 元 [soft check $> 1,000,000$]

HA075 What is the value of all outstanding (unpaid) loans from banks or financial institutions (not including mortgages) of [name]? [name] 还欠银行或其他金融机构的大笔贷款（不包括住房抵押贷款）有多少？____ Yuan 元 [Soft check $> 500,000$]

HA076 How often did the respondent receive assistance in answering section Household assets? 受访者填写该部分问卷时是否求助？

[IWER: If it is answered by a proxy, please record the respondent's reaction. 访员注意：如果是协助回答，请记录受访者的反应。]

1. Never 从未
2. A few times 偶尔几次
3. Most or all of the time 大多数时间

HB INDIVIDUAL ASSETS 个人资产

[IWER: Please conduct sections HB and HC when the main respondent and his/her spouse are at home. Don't allow a proxy to complete the entire sections. 访员注意：当主要受访者和其配偶在场时，才提问 HB 和 HC 部分问卷。这部分不允许请人完全代填。]

PART 1 Financial Assets 第一部分：金融资产

[The following questions pertain to your financial asset. 下面是关于金融资产的问题。]

[IWER reminder: make sure others are not present, IWER read following instructions: the following questions pertain to your financial asset, the answers to these questions will be kept strictly confidential and will be used for research purposes only. 访员注意：请确保没有其他人在场。请调查员念"下面是关于您的金融资产问题。我们将会对您的回答严格保密，并且仅用于学术研究。"]

HC001 How much cash is held by you and your spouse at home? 您和您爱人现在在家里有多少现金（随身携带以及放在家里的）? _____ Yuan 元 [soft check $> 50,000$ or < 100]
[IWER: Skip to HC003 if R answered HC001 and HC001 $\neq 0$. If not, ask unfolding brackets. 访员注意：如果受访者回答了HC001且回答不为 0，跳至 HC003，否则，提问分级展开问题]

HC002 [IWER: If R is unwilling to answer or does not remember, ask unfolding bracket questions here. 访员注意：如果受访者不愿回答或者忘记了，在此处分级展开提问] 500 /1,000 /2,000 /5,000 /10,000 yuan 元

HC003 Of which, how much do you own (if jointly owned with spouse then count 50%)? 其中，有多少是您名下的（如果和爱人共有，那么算 50%）? ____ (**HC003_1**) Yuan 元 [soft check $> 50,000$] or 或者 __ (**HC003_2**) % [range $0 - 100$]

[CAPI: prompt for HC004 - HC019: IWER: for deposit, bonds, stocks, and funds, only include assets legally in his/her name. 对 HC004 - HC019 提示：访员注意：对于存款、政府债券、股票和基金，只计算法定意义上在受访者名下的财产]

HC005 What is the total amount of deposits you are currently holding in financial institutions（eg: bank）? 您现在在金融机构 (如银行等) 存了多少钱? ______ Yuan 元 [soft check $> 500,000$ or < 100]

**PROCEDURE 程序:**
Skip to HC007 if R answered HC005. If not, ask unfolding brackets. 访员注意：如果受访者回答了 HC005，跳至 HC007，否则，提问分级展开问题。]

HC006 [IWER: If R is unwilling to answer or does not remember, ask unfolding bracket questions here. 访员注意：如果受访者不愿回答或者忘记了，在此处分级展开提问] 2,000/10,000/50,000 /100,000/500,000 yuan 元

HC007 Do you have any government bonds (e.g. Treasury bills) in your name? 您有（记在您名下的）政府债券（如国库券）吗?

1. Yes 有
2. No 没有 → go back HC010 返回到 HC010

HC008 What is the total face value of government bonds that you are currently holding? 您现有的所有政府债券 (如国库券等) 总共面值多少?_____ Yuan 元 [soft check $> 50,000$]

**PROCEDURE 程序:**
Skip to HC0010 if R answered HC008. If not, ask unfolding brackets HC009. 访员注意：如果受访者回答了 HC008，跳至 HC0010，否则，提问分级展开问题HC009。

HC009 [IWER: If R is unwilling to answer or does not remember, ask unfolding bracket questions here. 访员注意：如果受访者不愿回答或者忘记了，在此处分级展开提问] 10,000 /50,000 /100,000 /200,000 /500,000 yuan 元

HC010 Have you held any stocks in the past year in your name, excluding the equity or stock of your work unit? 不包括您在单位持有的股份，过去一年，您持有过（记在您名下的）股票吗？

1. Yes 有
2. No 没有 → Skip to HC015 请跳至 HC015

HC013 What is the present market value of all the stocks you are currently holding? 您现在持有的股票当前价值多少？ ______ Yuan 元 [soft check $> 200,000$]

PROCEDURE 程序:

Skip to HC015 if R answered HC013. If not, ask unfolding brackets. 访员注意：如果受访者回答了 HC013，跳至HC015，否则，提问分级展开问题。

HC014 [IWER: If R is unwilling to answer or does not remember, ask unfolding bracket questions here. 访员注意：如果受访者不愿回答或者忘记了，在此处分级展开提问] 10,000 /50,000 /100,000 /200,000 /500,000 yuan 元

HC015 Have you held any funds in your name in the past year? 过去一年，您持有过（记在您名下的）基金吗？

1. Yes 有
2. No 没有 → Go to PROGRAM before HC020 请跳至 HC020 前面的程序控制

HC018 What is the present market value of all the mutual funds you are currently holding? 您现在持有的基金当前价值多少？ ______ Yuan 元 [soft check $> 200,000$]

[IWER: Skip to HC020 if R answered HC018. If not, ask unfolding brackets. 访员注意：如果受访者回答了 HC018，跳至HC020，否则，提问分级展开问题。]

HC019 [IWER: If R is unwilling to answer or does not remember, ask unfolding bracket questions here. 访员注意：如果受访者不愿回答或者忘记了，在此处分级展开提问] 10,000 /50,000 /100,000 /200,000 /500,000 yuan 元

PROCEDURE 程序:

if HC005 $= 0$ and HC007 $= 2$ and HC010 $= 2$ and HC015 $= 2$, then skip HC020. 如果HC005 $= 0$，且HC007，HC010，HC015 答案都为 2，那么请跳过 HC020。

HC020 What percentage of the deposits, bonds, stocks, and funds held in your name is fully controlled by you and not your spouse? (%) 您名下的存款、政府债券、股票和基金有百分之多少是完全由你支配的？ ____ 0...100 % [hard check $\geq 0, \leq 100$]

HC021 Do you have any other deposits, bonds, stocks, or funds that belong to you but which are held in a person's name other than you or your spouse? 您是否还有其他存款、政府债券、股票和基金没有记在您或您爱人的名下？

1. Yes 是
2. No 否 → Skip to HC023 请跳至 HC023

HC022 What is the value of such assets? 这些资产的总价值是多少? ______ yuan 元 [soft check $> 200,000$]

HC023 Other than income you have already told me about, did you receive any other income from other **investments** in past year? 除了上述提到的收入，过去一年您还有其他**投资收入**吗?

1. Yes 是
2. No 否 → Skip toHC027 请跳至 HC027

[IWER:. 访员注意: 投资收入，指通过资本市场或资金市场借助特定投资工具进行投资得到的收益总称，包括分红收入、资本利得、利息收益、海外投资收益等。]

HC024 How much did you receive altogether from other **investments** in the past year? 您过去一年从其他**投资**中总共获得多少钱? ____ Yuan 元 [soft check $> 200,000$]

PROCEDURE 程序:

Skip to HC027 if R answered HC024. If not, ask unfolding brackets HC025. 访员注意: 如果受访者回答了 HC024，跳至 HC027，否则，提问分级展开问题HC025。]

HC025 [IWER: If R is unwilling to answer or does not remember, ask unfolding bracket questions here. 访员注意: 如果受访者不愿回答或者忘记了，在此处分级展开提问] 1,000 /5,000 /10,000 /20,000 /50,000 yuan 元

HC026 What percentage of the other investments is owned jointly with your spouse? 其他投资，有百分之多少是与您爱人共有的? ____ 0...100% [hard check ≤ 0, ≥ 100]

HC027 Do you have public housing funding? 您有住房公积金吗?

1. Yes 有
2. No 没有 → Skip to HC030 请跳至HC030

HC028 What is the total amount of money in your public housing fund? 您现在住房公积金账户上一共有多少钱? ______ Yuan 元 [soft check $> 100,000$]

PROCEDURE 程序:

Skip to HC030 if R answered HC028. If not, ask unfolding brackets HC029. 访员注意: 如果受访者回答了 HC028，跳至 HC030，否则，提问分级展开问题HC029。

HC029 [IWER: If R is unwilling to answer or does not remember, ask unfolding bracket questions here. 访员注意: 如果受访者不愿回答或者忘记了，在此处分级展开提问] 5,000/10,000 /50,000 /100,000 /200,000 yuan 元

HC030 Do you have Jizikuan that your work unit or other work units have collected from you and are still holding (Jizikuan is fund individuals provided to the work unit for the purpose of investment, building apartments, etc.)? 您有没有交给自己单位或其他单位的集资款还未返还?

1. Yes 有
2. No 没有 → Skip to HC033 请跳至 HC033

HC031 What is the amount of your jizikuan? 您的集资款总金额是多少? ______Y uan 元 [soft check > 200, 000]

**PROCEDURE 程序:**
Skip to HC033 if R answered HC031. If not, ask unfolding brackets HC032. 访员注意:如果受访者回答了 HC031, 跳至 HC033, 否则, 提问分级展开问题 HC032。

HC032 [IWER: If R is unwilling to answer or does not remember, ask unfolding bracket questions here. 访员注意:如果受访者不愿回答或者忘记了,在此处分级展开提问] 5,000/10,000 /50,000 /100,000 /200,000 yuan 元

HC033 Do you have any unpaid salary that your work unit still owes you? 目前,有单位拖欠您的工资吗?
1. Yes 有
2. No 没有 → Skip to HC036 请跳至 HC036

HC034 What is the amount of your unpaid salary? 一共拖欠您多少工资? ______ Yuan 元 [soft check > 100, 000]

**PROCEDURE 程序:**
Skip to HC036 if R answered HC034. If not, ask unfolding brackets HC035. 访员注意:如果受访者回答了 HC034, 跳至 HC036, 否则, 提问分级展开问题 HC035。

HC035 [IWER: If R is unwilling to answer or does not remember, ask unfolding bracket questions here. 访员注意:如果受访者不愿回答或者忘记了,在此处分级展开提问] 5,000/10,000 /50,000 /100,000 /200,000 yuan 元

HC036 Have you participated in any rotating savings and credit association during the past year? 您在过去一年里有没有参加过标会?
1. Yes 有
2. No 没有 → Skip to HD001 请跳至 HD001

HC037 What is the total amount of funds that you are still obligated to pay to the rotating savings and credit association? 您还需要支付给标会多少钱? ______ yuan 元

PART 2 DEBTS 第二部分: 债务

[The following questions pertain to your debt. 下面是关于您债务的问题。]

HD001 What is the total amount of loan that you haven't repaid yet (not including loans for house)? 您尚未还清的贷款总额(不包括买房、建房、装修的贷款)是多少? ______ Yuan 元 [soft check > 500, 000]

HD002_W2_1 What is the total amount of personal loan that you haven't repaid yet? 尚未还清的个人间借款总额是多少? ______ Yuan 元 [soft check > 500,000]

**PROCEDURE 程序:**<br>Skip to HD003 if R answered HD001 and HD002_W2_1. If not, ask unfolding brackets HD002 and HD002_W2. 访员注意: 如果受访者回答了HD001和HD002_W2_1, 跳至HD003, 否则, 提问分级展开问题 HD002 和 HD002_W2。

HD002 [IWER: If R is unwilling to answer or does not remember, ask unfolding bracket questions here. 访员注意: 如果受访者不愿回答或者忘记了, 在此处分级展开提问] 5,000/10,000 /50,000 /100,000 /500,000 yuan 元

HD002_W2 [IWER: If R is unwilling to answer or does not remember, ask unfolding bracket questions here. 访员注意: 如果受访者不愿回答或者忘记了, 在此处分级展开提问] 5,000/10,000 /50,000 /100,000 /500,000 yuan 元

HD003 What is the amount of your credit card balance? 您有信用卡吗? 有的话现在您的信用卡所欠金额是多少? _____ Yuan 元 [soft check > 50,000] (如果没有, 请填 0)

**PROCEDURE 程序:**<br>Skip to HD005 if R answered HD003 . If not, ask unfolding brackets HD004. 访员注意: 如果受访者回答了 HD003, 跳至 HD005, 否则, 提问分级展开问题 HD004。

HD004 [IWER: If R is unwilling to answer or does not remember, ask unfolding bracket questions here. 访员注意: 如果受访者不愿回答或者忘记了, 在此处分级展开提问] 500/1,000 /5,000 /10,000 /50,000 yuan 元

HD005 Have you ever inherited anything? 您是否继承过遗产?

1. Yes 是
2. No 否 → Skip toHD012 请跳至HD012

HD006 How much in total have you inherited? 您继承的遗产总共值多少钱? ______ Yuan 元 (按继承时的价值来算) [soft check > 200,000]

**PROCEDURE 程序:**<br>Skip to HD008 if R answered HD006. If not, ask unfolding brackets HD007. 访员注意: 如果受访者回答了 HD006, 跳至 HD008, 否则, 提问分级展开问题 HD007]

HD007 [IWER: If R is unwilling to answer or does not remember, ask unfolding bracket questions here. 访员注意: 如果受访者不愿回答或者忘记了, 在此处分级展开提问] 5,000/10,000 /50,000 /100,000 /500,000 yuan 元

HD008 From whom you inherited? (Choose all that apply) 您从谁那里继承了遗产? (可多选)

1. Parents 父母
2. Parents-in-law 岳父母

3. Children 子女
4. Relatives 亲戚
5. Others 其它

HD009 When did the largest inheritance occur? 您继承遗产最多的一次发生在哪一年? ____ 1900...2013 Year 年
[IWER: Mark the year using four digits. 访员注意: 用四位数字表示年份。]

HD010 What was the value of that inheritance? 当时这笔遗产值多少钱? ______ Yuan 元 [soft check > 200, 000]

PROCEDURE 程序:
Skip to HD012 if R answered HD010. If not, ask unfolding brackets HD011. 访员注意: 如果受访者回答了 HD010, 跳至 HD012, 否则, 提问分级展开问题 HD011。

HD011 [IWER: If R is unwilling to answer or does not remember, ask unfolding bracket questions here. 访员注意: 如果受访者不愿回答或者忘记了, 在此处分级展开提问] 5,000/10,000 /50,000 /100,000 /500,000 yuan 元

HD012 How often did the respondent receive assistance in answering section H ASSETS? 受访者填写该部分问卷时是否求助?
[IWER: If it is answered by a proxy, please record the respondent's reaction. 访员注意: 如果是协助回答, 请记录受访者的反应。]
1. Never 从未
2. A few times 偶尔几次
3. Most or all of the time 大多数时间

I HOUSING CHARACTERISTICS 住房情况

PROCEDURE 程序: Only main respondent answer I001 - I026. 仅主要受访者回答 I001 - I026

I001 What is the construction area of your residence? 您现在住房的建筑面积是多大？____ m^2 平方米 [soft check: <10 or >500]

I002 What is the total housing land area? 宅基地的面积有多大?（宅基地是指农村集体经济组织为保障农户生活需要而拨给农户建造房屋及小庭院使用的土地）____ m^2 平方米 [soft check <10, >1000]
[访员注意：如果没有此项，请填写"0"。]

I003 Is your residence used for business as well? 这个住房兼作生产经营用房吗？

1. Yes 是
2. No 否

I004 What type of structure is this building? Is it concrete and steel, mixed structure, bricks and wood, wood, bamboo, grass or other? 本建筑是什么结构？

1. Concrete and steel 钢筋混凝土结构
2. Bricks and wood 砖木结构
3. Mixed structure 混合结构
4. Wood, bamboo, grass 木、竹、草结构
5. Woolen felt 毛毡
6. Sheet iron 简易铁皮房
7. Cave dwelling 窑洞
8. Tent 帐篷
9. Adobe 土坯房
10. Other 其它结构

I005 When was this house built? 这个房子是什么时候建成的？____ Year 年
If R is unclear about year, please choose among following items. 如果不清楚具体年份，从以下选项中选择：

1. 0 – 5 years 0 – 5 年
2. 5 – 10 years 5 – 10 年
3. 10 – 20 years 10 – 20 年
4. 20 – 30 years 20 – 30 年
5. 30 – 40 years 30 – 40 年
6. more than 40 years 40 年以上

I006 Is the building one story or multi-level building? 住房所在建筑是平房还是楼房？

1. One-story building 平房 → skip to I007
2. Multi-story building 楼房 → skip to I008

I007 Is the story independent or compound? 是独立的平房，还是大杂院？

1. Independent story 独立的平房
2. Compound 大杂院

I008 Which story is this building on? 该住房在第几层？ ___

**PROCEDURE 程序**: If I008 > 1, ask I009. 如果 I008 > 1，询问I009。

I009 Does it has elevator? 有电梯吗？

1. Yes 是
2. No 否

I010 Are there any handicapped facilities (e.g., non-stair ramp)? 住宅是否有无障碍通道？（例如：没有台阶的斜坡等）

1. Yes 是
2. No 否

**PROCEDURE 程序**: If I010 = 2, ask I011. 如果 I010 = 2，询问I011。

I011 How many steps had to be climbed to get to the main entrance of the household's flat? 从外面回来要爬多少个阶梯才能到家门口？
[IWER: Do not count steps if an elevator is available. 访员注意：可以坐电梯而不用爬的阶梯忽略不计。]

1. 0 0 个
2. 1 to 5 1 – 5 个阶梯
3. 6 to 15 6 – 15 个阶梯
4. 16 to 25 16 – 25 个阶梯
5. More than 25 25 个阶梯以上

I012 How many bedrooms, living rooms, bathrooms, and kitchens are there in your residence? 您家现在的房屋有 __ (**I012_1**) bedrooms 室 __ (**I012_2**) living rooms 厅 __ (**I012_3**) toilets 卫生间（厕所） __ (**I012_4**) kitchens 厨房 __ (**I012_5**) balcony 阳台 [soft check: > 20]

**PROCEDURE 程序**: Ask I013 if no toilets in the answer to I012. 如果I012答案中没有卫生间，则提问I013。

I013 How far is the nearest toilet to your house? 离您家最近的厕所多远？ ____ (**I013**) meters 米 [soft check: > 500]

I014 What is the type of toilet? 厕所是什么样的？是蹲坑式还是坐式？

1. Toilet without a seat 蹲坑式
2. Toilet with a seat 坐式

I015 Is the toilet flushable? 厕所能冲水吗?
1. Yes 是
2. No 否

I016 Does your residence have electricity? 您家的住房是否有电?
1. Yes 是
2. No 否

I017 Does your residence have running water? 是否有自来水?
1. Yes 是
2. No 否

I018 Is there in-house shower or bath facility? What type? 住房内有无洗澡设施? 是什么样的?
1. Hot water provided 统一供热水
2. Water heater installed by the household 家庭自装热水器
3. No 无

I019 Does your residence have coal gas or natural gas supply? 是否有管道煤气或天然气?
1. Yes 是
2. No 否

I020 Does your residence have heating? 是否带供暖设施（不包括土暖气和可制暖的空调）?
1. Yes 是 → skip to I022 请跳至I022
2. No 否

I021 What is the main heating energy source? 供暖所用的主要能源是什么?
1. Solar 太阳能
2. Coal 煤炭、蜂窝煤
3. Natural gas 管道天然气或煤气
4. Liquefied Petroleum Gas 液化石油气
5. Electric 电
6. Crop residue/Wood buring 秸秆、柴火
7. Other 其他

I022 What is the main source of cooking fuel? 做饭用的主要燃料是什么?
1. Coal 煤炭、蜂窝煤
2. Natural gas 管道天然气或煤气
3. Marsh gas 沼气
4. Liquefied Petroleum Gas 液化石油气
5. Electric 电
6. crop residue/Wood burning 秸秆、柴火
7. other 其他

I023 Does your residence have a telephone connection? 您家装电话了吗?

1. Yes 是
2. No 否

I024 Does your residence have broad-band internet connection? 您家可以宽带上网吗?
1. Yes 是
2. No 否

I025 [Interviewer records it] [访员自己记录] How clear and tity is in this household? 这户人家的室内整洁度如何?
1. Excellent 非常整洁
2. Very clear 很整洁
3. Clear 整洁
4. Fair 一般
5. Poor 不整洁
6. Not applicable 不适用

I026 [Interviewer records it] [访员自己记录] How is the temperature in this household? 这户人家的室内温度如何?
1. Very hot 很热
2. Hot 比较热
3. Bearable 还可以
4. Cold 比较冷
5. Very cold 很冷
6. Not applicable 不适用

J INTERVIEWER OBSERVATION 访问员观察

[IWER. This section is about your observations during the interview and should be filled out after each completed individual interview. 访员注意：这部分是您在调查时所做的观察，访问结束后，请填写这部分内容。]

J001 Were any third persons, except proxy respondents, present during (parts of) the interview with [Respondent Name]? (circle all that apply) 当您访问主要受访者时，除代理回答人外，是否还有其他人在场？(可多选)

1. Nobody 没有其他人 → Skip to J003 跳至 J003
2. Spouse or partner 受访者的配偶
3. Parent or parents 受访者的父母亲
4. Child or children 受访者的孩子
5. Other relatives 其他亲戚
6. Other persons present 还有其他的人在场

J002 Did these persons intervene during the interview? 调查时，这人/这些人有没有干预？

1. Yes, often 有，经常干预
2. Yes, occasionally 有，偶尔干预
3. No 没有

J003 How would you describe the willingness of [Respondent Name] to answer? 受访者回答问卷时配合程度如何？

1. Very good 非常配合 → Skip to J005 跳至 J005
2. Good 比较配合 → Skip to J005 跳至 J005
3. Fair 一般 → Skip to J005 跳至 J005
4. Bad 不太配合 → Skip to J005 跳至 J005
5. Good in the beginning, got worse during the interview 刚开始配合，后来就不太配合
6. Bad in the beginning, got better during the interview 刚开始不太配合，后来态度慢慢变好了 → Skip to J005 跳至 J005

J004 Why did the respondent's willingness to answer get worse during the interview? (Choose all that apply) 受访者为什么后来不大愿意回答问卷了？(可多选)

1. The respondent was losing interest 没有兴趣了
2. The respondent was losing concentration or was getting tired 受访者注意力不能集中了，有些厌倦
3. Other, please specify 其他，请注明 ______

J005 Did [Respondent Name] ask for clarification on any questions? 受访者回答问题时，需要您来解释吗？

1. Never 根本不需要
2. Almost never 基本上不需要
3. Now and then 偶尔需要

4. Often 经常需要
5. Very often 频繁需要
6. Always 一直需要

J006 Overall, did you feel that [Respondent Name] understood the questions? 总的来说，您觉得受访者能听懂这些题目吗？

1. Never 全部听不懂
2. Almost never 几乎听不懂
3. Now and then 偶尔能听懂
4. Often 大部分能听懂
5. Very often 绝大部分能听懂
6. Always 全部能听懂

J007 Did the respondent need any help reading the showcards during the interview? 出示卡片时，受访者要您帮忙读卡片？

1. Yes, due to sight problems 要，由于视力问题
2. Yes, due to literacy problems 要，因为不识字
3. No 不需要

J008_W2 During the visit, the respondents primarily use which of the following languages? 访问过程中，受访者主要使用以下哪种语言？

1. Mandarin 普通话 → 跳至 J009_W2
2. Dialect 方言 → 跳至 J010_W2

J009_W2 What about the level of mandarin of the respondent? 受访者回答问卷时，普通话标准程度如何？

1. Very Good 非常标准 → 跳过 J010_W2 - J011_W2
2. Good 比较标准 → 跳过 J010_W2 - J011_W2
3. Fair 一般 → 跳过 J010_W2 - J011_W2
4. Mixed with Some Local Accent 夹杂部分地方口音 → 跳至 J011_W2
5. Mixed with Lot of Local Accent 夹杂大量地方口音 → 跳至 J011_W2

J010_W2 During the visit, the respondents primarily use which dialect? ____ 受访者回答问卷时，使用哪种方言？ ____

[访员注意：填写方言城市名称（如苏州方言），或方言语系（如客家话）。]

J011_W2 During the visit, the respondents mixed with which local accent? ____ 受访者回答问卷时，夹杂哪种地方口音？ ____

[访员注意：填写方言城市名称（如苏州方言），或方言语系（如客家话）。]

PROCEDURE 程序: If Proxy, ask J012_W2 - J013_W2. 如果使用了代理，则询问 J012_W2 - J013_W2。

J012_W2 Name of Proxy 代理人的姓名 _____

J013_W2 Relationship between proxy and respondent 代理人与受访者的关系

1. Spouse 配偶
2. Mother 母亲
3. Father 父亲
4. Mother-in-law 岳母/婆婆
5. Father-in-law 岳父/公公
6. Sibling 兄弟姐妹
7. Brother-in-law, sister-in-law 姐夫妹夫/嫂子弟媳
8. Child 孩子
9. Spouse of child 孩子的配偶
10. Grandchild 孙子女
11. Other relative 其他亲戚
12. Helper or other non-relative 帮忙的人或者其他非亲属
